# Supplementary material for: The Use of Pro-Angiogenic and/or Pro-Hypoxic miRNAs as Tools to Monitor Patients with Diffuse Gliomas
Source: Int J Mol Sci. 2022 May 27;23(11):6042. doi: 10.3390/ijms23116042 (PMC9181142; doi:10.3390/ijms23116042)
Supplement: Supplementary file 1 [file ijms-23-06042-s001.zip › ijms-1728913-supplementary.pdf]

Table S1. Patients' data for each histology.

	isocitrate dehydrogenase 1/2 (IDH)-mutant and 1p19q-codeleted oligodendrogliomas	IDH-mutant astrocytomas	IDH-mutant glioblastomas	IDH-wild type glioblastomas
	n=27	n=25	n=8	n=37
**Main molecular alterations**	IDH1/2 mutation<br>1p19q deletion	IDH1/2 mutation<br>ATRX loss	IDH1/2 mutation<br>ATRX loss	
**Grading**	11 grade II<br>16 grade III	18 grade II<br>7 grade III	8 grade IV	37 grade IV
**Gender**	15 (55.5%) men<br>12 (44.5%) women	14 (56%) men<br>11 (44%) women	5 (62.5%) men<br>3 (37.5%) women	26 (70.3%) men<br>11 (29.3%) women
**Median age at diagnosis** ([range])	51.4 years<br>[26.7-63.9]	36.3 years<br>[23.6-66.9]	37.9 years<br>[22.3-73.5]	67.3 years<br>[50.2-79.3]
**Resection**				
Complete:	13 (48%)	5 (20%)	4 (50%)	19 (52%)
Subtotal:	3 (11%)	5 (20%)	0	5 (14%)
Partial:	11 (41%)	13 (52%)	4 (50%)	7 (19%)
Only biopsied:	0	1 (4%)	0	6 (16%)
Unknown :	0	1 (4%)	0	0
**Treatment following surgery**				
Stupp protocol:	2 (7%)	5 (20%)	4 (50%)	28 (76%)
Radiotherapy:	9 (33%)	2 (8%)	2 (25%)	3 (8%)
Chemotherapy:	5 (19%)	4 (16%)	2 (25%)	1 (2%)
None:	8 (29%)	14 (56%)	0	3 (8%)
Unknown :	3 (11%)	0	0	2 (2%)
**Median follow-up period** ([range])	72.64 months<br>[7.85-304.23]	62.82 months<br>[7.26-121.48]	39.34 months<br>[8.64-114.00]	8.67 months<br>[0.26-61.24]
**Recurrence (number / delay** [range])	15 patients<br>***median period***:<br>55.03 months<br>[7.79-130.07]	12 patients<br>***median period***:<br>22.19 months<br>[8.51-210.23]	4 patients<br>***median period***:<br>7.62 months<br>[6.54-40.77]	19 patients<br>***median period***:<br>7.03 months<br>[1.12-24.18]
**Median overall survival OS**	119 months	92.2 months	19.7 months	14.1 months

OS: overall survival; IDH: isocitrate dehydrogenase; ATRX: alpha-thalassemia retardation syndrome

Table S2. Main targets of the miRNA studied.

miRNA Name	Gene Symbol	Gene Description
hsa-miR-100-5p	TRIB2	tribbles pseudokinase 2
hsa-miR-100-5p	KBTBD8	kelch repeat and BTB domain containing 8
hsa-miR-100-5p	SMARCA5	SWI/SNF related, matrix associated, actin dependent regulator of chromatin, subfamily a, member 5
hsa-miR-100-5p	MTOR	mechanistic target of rapamycin kinase
hsa-miR-100-5p	HS3ST2	heparan sulfate-glucosamine 3-sulfotransferase 2
hsa-miR-100-5p	RAVER2	ribonucleoprotein, PTB binding 2
hsa-miR-100-5p	ZZEF1	zinc finger ZZ-type and EF-hand domain containing 1
hsa-miR-100-5p	BAZ2A	bromodomain adjacent to zinc finger domain 2A
hsa-miR-100-5p	NOX4	NADPH oxidase 4
hsa-miR-100-5p	HS3ST3B1	heparan sulfate-glucosamine 3-sulfotransferase 3B1
hsa-miR-100-5p	ETFDH	electron transfer flavoprotein dehydrogenase
hsa-miR-100-5p	TTC39A	tetratricopeptide repeat domain 39A
hsa-miR-100-5p	FZD8	frizzled class receptor 8
hsa-miR-100-5p	NR6A1	nuclear receptor subfamily 6 group A member 1
hsa-miR-100-5p	FGFR3	fibroblast growth factor receptor 3
hsa-miR-100-5p	PCSK9	proprotein convertase subtilisin/kexin type 9
hsa-miR-100-5p	ATP11C	ATPase phospholipid transporting 11C
hsa-miR-100-5p	CTDSPL	CTD small phosphatase like
hsa-miR-100-5p	TAOK1	TAO kinase 1
hsa-miR-100-5p	MBNL1	muscleblind like splicing regulator 1
hsa-miR-100-5p	PRDM1	PR/SET domain 1
hsa-miR-100-5p	ADCY1	adenylate cyclase 1
hsa-miR-100-5p	MTMR3	myotubularin related protein 3
hsa-miR-100-5p	AGO2	argonaute RISC catalytic component 2
hsa-miR-100-5p	THAP2	THAP domain containing 2
hsa-miR-100-5p	ZADH2	zinc binding alcohol dehydrogenase domain containing 2
hsa-miR-100-5p	ST6GALNAC4	ST6 N-acetylgalactosaminide alpha-2,6-sialyltransferase 4
hsa-miR-100-5p	ZNF197	zinc finger protein 197
hsa-miR-100-5p	RMND5A	required for meiotic nuclear division 5 homolog A
hsa-miR-100-5p	LRRC8B	leucine rich repeat containing 8 VRAC subunit B
hsa-miR-100-5p	PPP3CA	protein phosphatase 3 catalytic subunit alpha
hsa-miR-100-5p	EPDR1	ependymin related 1
hsa-miR-100-5p	HOXA1	homeobox A1
hsa-miR-100-5p	TRIM71	tripartite motif containing 71
hsa-miR-100-5p	AP1AR	adaptor related protein complex 1 associated regulatory protein
hsa-miR-100-5p	RAP1B	RAP1B, member of RAS oncogene family
hsa-miR-100-5p	RNF144B	ring finger protein 144B

hsa-miR-100-5p	PPFIA3	PTPRF interacting protein alpha 3
hsa-miR-100-5p	HES7	hes family bHLH transcription factor 7
hsa-miR-100-5p	TMPRSS13	transmembrane serine protease 13
hsa-miR-100-5p	DESI2	desumoylating isopeptidase 2
hsa-miR-100-5p	ZNRF2	zinc and ring finger 2
hsa-miR-100-5p	RASA2	RAS p21 protein activator 2
hsa-miR-100-5p	CAMTA1	calmodulin binding transcription activator 1
hsa-miR-100-5p	CDC25A	cell division cycle 25A
hsa-miR-100-5p	TRIB1	tribbles pseudokinase 1
hsa-miR-100-5p	CDYL2	chromodomain Y like 2
hsa-miR-126-5p	HSPB8	heat shock protein family B (small) member 8
hsa-miR-126-5p	PDE7B	phosphodiesterase 7B
hsa-miR-126-5p	FBXO6	F-box protein 6
hsa-miR-126-5p	TRMT9B	tRNA methyltransferase 9B (putative)
hsa-miR-126-5p	TNFAIP8L3	TNF alpha induced protein 8 like 3
hsa-miR-126-5p	HOXB6	homeobox B6
hsa-miR-126-5p	GNE	glucosamine (UDP-N-acetyl)-2-epimerase/N-acetylmannosamine kinase
hsa-miR-126-5p	NAT1	N-acetyltransferase 1
hsa-miR-126-5p	CASP9	caspase 9
hsa-miR-126-5p	TRIM8	tripartite motif containing 8
hsa-miR-126-5p	TMEM182	transmembrane protein 182
hsa-miR-126-5p	MR1	major histocompatibility complex, class I-related
hsa-miR-126-5p	ESRRG	estrogen related receptor gamma
hsa-miR-126-5p	S1PR3	sphingosine-1-phosphate receptor 3
hsa-miR-126-5p	A2ML1	alpha-2-macroglobulin like 1
hsa-miR-126-5p	MDM4	MDM4, p53 regulator
hsa-miR-126-5p	BRWD3	bromodomain and WD repeat domain containing 3
hsa-miR-126-5p	RFX4	regulatory factor X4
hsa-miR-126-5p	TRPS1	transcriptional repressor GATA binding 1
hsa-miR-126-5p	ARL11	ADP ribosylation factor like GTPase 11
hsa-miR-126-5p	FAM168A	family with sequence similarity 168 member A
hsa-miR-126-5p	CASK	calcium/calmodulin dependent serine protein kinase
hsa-miR-126-5p	DENND1B	DENN domain containing 1B
hsa-miR-126-5p	GRIK2	glutamate ionotropic receptor kainate type subunit 2
hsa-miR-126-5p	TBCA	tubulin folding cofactor A
hsa-miR-126-5p	HOXC8	homeobox C8
hsa-miR-126-5p	INPP5D	inositol polyphosphate-5-phosphatase D
hsa-miR-126-5p	CAMK2A	calcium/calmodulin dependent protein kinase II alpha
hsa-miR-126-5p	GABRA4	gamma-aminobutyric acid type A receptor alpha4 subunit
hsa-miR-126-5p	CD84	CD84 molecule
hsa-miR-126-5p	GGT6	gamma-glutamyltransferase 6
hsa-miR-126-5p	TSC22D4	TSC22 domain family member 4
hsa-miR-126-5p	MACC1	MET transcriptional regulator MACC1
hsa-miR-126-5p	GABRB2	gamma-aminobutyric acid type A receptor beta2 subunit

hsa-miR-126-5p	RORA	RAR related orphan receptor A
hsa-miR-126-5p	MAP3K2	mitogen-activated protein kinase kinase kinase 2
hsa-miR-126-5p	FGF7	fibroblast growth factor 7
hsa-miR-126-5p	CNTN1	contactin 1
hsa-miR-126-5p	SETD9	SET domain containing 9
hsa-miR-126-5p	CHST11	carbohydrate sulfotransferase 11
hsa-miR-126-5p	PI15	peptidase inhibitor 15
hsa-miR-126-5p	VPS13A	vacuolar protein sorting 13 homolog A
hsa-miR-126-5p	YIPF6	Yip1 domain family member 6
hsa-miR-126-5p	PNRC1	proline rich nuclear receptor coactivator 1
hsa-miR-126-5p	MYEF2	myelin expression factor 2
hsa-miR-126-5p	KCNB1	potassium voltage-gated channel subfamily B member 1
hsa-miR-126-5p	COMMD2	COMM domain containing 2
hsa-miR-126-5p	SHISAL1	shisa like 1
hsa-miR-126-5p	FXN	frataxin
hsa-miR-126-5p	TUBGCP4	tubulin gamma complex associated protein 4
hsa-miR-126-5p	MMRN1	multimerin 1
hsa-miR-126-5p	ZDHHC15	zinc finger DHHC-type containing 15
hsa-miR-126-5p	PKHD1	PKHD1, fibrocystin/polyductin
hsa-miR-126-5p	KIRREL1	kirre like nephrin family adhesion molecule 1
hsa-miR-126-5p	STEAP2	STEAP2 metalloreductase
hsa-miR-126-5p	RABL3	RAB, member of RAS oncogene family like 3
hsa-miR-126-5p	EBF1	EBF transcription factor 1
hsa-miR-126-5p	ZIC5	Zic family member 5
hsa-miR-126-5p	DBNDD2	dysbindin domain containing 2
hsa-miR-126-5p	C5orf47	chromosome 5 open reading frame 47
hsa-miR-126-5p	ZBTB41	zinc finger and BTB domain containing 41
hsa-miR-126-5p	SLC25A53	solute carrier family 25 member 53
hsa-miR-126-5p	TSHZ3	teashirt zinc finger homeobox 3
hsa-miR-126-5p	TTF2	transcription termination factor 2
hsa-miR-126-5p	AMFR	autocrine motility factor receptor
hsa-miR-126-5p	SRSF12	serine and arginine rich splicing factor 12
hsa-miR-126-5p	TECPR2	tectonin beta-propeller repeat containing 2
hsa-miR-126-5p	ACAN	aggrecan
hsa-miR-126-5p	SYT10	synaptotagmin 10
hsa-miR-126-5p	ADH4	alcohol dehydrogenase 4 (class II), pi polypeptide
hsa-miR-126-5p	VWC2	von Willebrand factor C domain containing 2
hsa-miR-126-5p	RINL	Ras and Rab interactor like
hsa-miR-126-5p	NDUFS1	NADH:ubiquinone oxidoreductase core subunit S1
hsa-miR-126-5p	NWD1	NACHT and WD repeat domain containing 1
hsa-miR-126-5p	CHRNA5	cholinergic receptor nicotinic alpha 5 subunit
hsa-miR-126-5p	EREG	epiregulin
hsa-miR-126-5p	CCDC32	coiled-coil domain containing 32
hsa-miR-126-5p	TMEM41B	transmembrane protein 41B
hsa-miR-126-5p	GUCY1A2	guanylate cyclase 1 soluble subunit alpha 2

hsa-miR-126-5p	RAB30	RAB30, member RAS oncogene family
hsa-miR-126-5p	WFDC13	WAP four-disulfide core domain 13
hsa-miR-126-5p	L2HGDH	L-2-hydroxyglutarate dehydrogenase
hsa-miR-126-5p	LDLRAD2	low density lipoprotein receptor class A domain containing 2
hsa-miR-126-5p	DLG1	discs large MAGUK scaffold protein 1
hsa-miR-126-5p	TBC1D15	TBC1 domain family member 15
hsa-miR-126-5p	TMEM56	transmembrane protein 56
hsa-miR-126-5p	SULT6B1	sulfotransferase family 6B member 1
hsa-miR-126-5p	PPFIA2	PTPRF interacting protein alpha 2
hsa-miR-126-5p	MEX3A	mex-3 RNA binding family member A
hsa-miR-126-5p	METTL17	methyltransferase like 17
hsa-miR-126-5p	FAM111A	family with sequence similarity 111 member A
hsa-miR-126-5p	NCOA7	nuclear receptor coactivator 7
hsa-miR-126-5p	C2CD4A	C2 calcium dependent domain containing 4A
hsa-miR-126-5p	SAMSN1	SAM domain, SH3 domain and nuclear localization signals 1
hsa-miR-126-5p	MFAP4	microfibril associated protein 4
hsa-miR-126-5p	TRIP6	thyroid hormone receptor interactor 6
hsa-miR-126-5p	THAP3	THAP domain containing 3
hsa-miR-126-5p	CHMP5	charged multivesicular body protein 5
hsa-miR-126-5p	SENP7	SUMO specific peptidase 7
hsa-miR-126-5p	NFIA	nuclear factor I A
hsa-miR-126-5p	CCP110	centriolar coiled-coil protein 110
hsa-miR-126-5p	ELAVL4	ELAV like RNA binding protein 4
hsa-miR-126-5p	SOX6	SRY-box 6
hsa-miR-126-5p	ADAM9	ADAM metallopeptidase domain 9
hsa-miR-126-5p	ZNF135	zinc finger protein 135
hsa-miR-126-5p	CHST7	carbohydrate sulfotransferase 7
hsa-miR-126-5p	SRD5A3	steroid 5 alpha-reductase 3
hsa-miR-126-5p	KL	klotho
hsa-miR-126-5p	VCPIP1	valosin containing protein interacting protein 1
hsa-miR-126-5p	MAT2B	methionine adenosyltransferase 2B
hsa-miR-126-5p	NFAT5	nuclear factor of activated T cells 5
hsa-miR-126-5p	F9	coagulation factor IX
hsa-miR-126-5p	JPH1	junctophilin 1
hsa-miR-126-5p	BICD2	BICD cargo adaptor 2
hsa-miR-126-5p	FGD2	FYVE, RhoGEF and PH domain containing 2
hsa-miR-126-5p	LPP	LIM domain containing preferred translocation partner in lipoma
hsa-miR-126-5p	PCBD2	pterin-4 alpha-carbinolamine dehydratase 2
hsa-miR-126-5p	PTPN20	protein tyrosine phosphatase, non-receptor type 20
hsa-miR-126-5p	ATP8B1	ATPase phospholipid transporting 8B1
hsa-miR-126-5p	MMP16	matrix metallopeptidase 16
hsa-miR-126-5p	HECTD2	HECT domain E3 ubiquitin protein ligase 2
hsa-miR-126-5p	SYNPO2	synaptopodin 2
hsa-miR-126-5p	CCL28	C-C motif chemokine ligand 28
hsa-miR-126-5p	CDCA7	cell division cycle associated 7

hsa-miR-126-5p	NOVA1	NOVA alternative splicing regulator 1
hsa-miR-126-5p	C8orf44-SGK3	C8orf44-SGK3 readthrough
hsa-miR-126-5p	SGK3	serum/glucocorticoid regulated kinase family member 3
hsa-miR-126-5p	CAMK4	calcium/calmodulin dependent protein kinase IV
hsa-miR-126-5p	FRYL	FRY like transcription coactivator
hsa-miR-126-5p	CCR1	C-C motif chemokine receptor 1
hsa-miR-126-5p	NUP43	nucleoporin 43
hsa-miR-126-5p	FUT9	fucosyltransferase 9
hsa-miR-126-5p	SPAST	spastin
hsa-miR-126-5p	CALHM1	calcium homeostasis modulator 1
hsa-miR-126-5p	ARSK	arylsulfatase family member K
hsa-miR-126-5p	MCTP1	multiple C2 and transmembrane domain containing 1
hsa-miR-126-5p	UBE2W	ubiquitin conjugating enzyme E2 W
hsa-miR-126-5p	KCNN3	potassium calcium-activated channel subfamily N member 3
hsa-miR-126-5p	HNMT	histamine N-methyltransferase
hsa-miR-126-5p	RFTN2	raftlin family member 2
hsa-miR-126-5p	PREX2	phosphatidylinositol-3,4,5-trisphosphate dependent Rac exchange factor 2
hsa-miR-126-5p	HOXB5	homeobox B5
hsa-miR-126-5p	MAPK10	mitogen-activated protein kinase 10
hsa-miR-126-5p	PROCR	protein C receptor
hsa-miR-126-5p	C5orf34	chromosome 5 open reading frame 34
hsa-miR-126-5p	PDZD8	PDZ domain containing 8
hsa-miR-126-5p	ZNF695	zinc finger protein 695
hsa-miR-126-5p	ETV1	ETS variant 1
hsa-miR-126-5p	IDS	iduronate 2-sulfatase
hsa-miR-126-5p	SPATA2	spermatogenesis associated 2
hsa-miR-126-5p	MBNL3	muscleblind like splicing regulator 3
hsa-miR-126-5p	TET2	tet methylcytosine dioxygenase 2
hsa-miR-126-5p	LONRF3	LON peptidase N-terminal domain and ring finger 3
hsa-miR-126-5p	HPS5	HPS5, biogenesis of lysosomal organelles complex 2 subunit 2
hsa-miR-126-5p	CYP7B1	cytochrome P450 family 7 subfamily B member 1
hsa-miR-126-5p	ZNF703	zinc finger protein 703
hsa-miR-126-5p	NCAN	neurocan
hsa-miR-126-5p	EDA2R	ectodysplasin A2 receptor
hsa-miR-126-5p	SLC49A4	solute carrier family 49 member 4
hsa-miR-126-5p	DTD1	D-tyrosyl-tRNA deacylase 1
hsa-miR-126-5p	DHX35	DEAH-box helicase 35
hsa-miR-126-5p	AMACR	alpha-methylacyl-CoA racemase
hsa-miR-126-5p	MDH1	malate dehydrogenase 1
hsa-miR-126-5p	SETBP1	SET binding protein 1
hsa-miR-126-5p	CCDC13	coiled-coil domain containing 13
hsa-miR-126-5p	USP14	ubiquitin specific peptidase 14
hsa-miR-126-5p	CLEC1A	C-type lectin domain family 1 member A
hsa-miR-126-5p	PLCB1	phospholipase C beta 1

hsa-miR-126-5p	RGR	retinal G protein coupled receptor
hsa-miR-126-5p	GLYAT	glycine-N-acyltransferase
hsa-miR-126-5p	MGAT3	mannosyl (beta-1,4-)-glycoprotein beta-1,4-N-acetylglucosaminyltransferase
hsa-miR-126-5p	TRPC5	transient receptor potential cation channel subfamily C member 5
hsa-miR-126-5p	JARID2	jumonji and AT-rich interaction domain containing 2
hsa-miR-126-5p	DYRK2	dual specificity tyrosine phosphorylation regulated kinase 2
hsa-miR-126-5p	ERGIC2	ERGIC and golgi 2
hsa-miR-126-5p	PSMA1	proteasome subunit alpha 1
hsa-miR-126-5p	ADGRB3	adhesion G protein-coupled receptor B3
hsa-miR-126-5p	TERB2	telomere repeat binding bouquet formation protein 2
hsa-miR-126-5p	ARL5A	ADP ribosylation factor like GTPase 5A
hsa-miR-126-5p	ZNF687	zinc finger protein 687
hsa-miR-126-5p	GRIK1	glutamate ionotropic receptor kainate type subunit 1
hsa-miR-126-5p	RWDD4	RWD domain containing 4
hsa-miR-126-5p	CELF2	CUGBP Elav-like family member 2
hsa-miR-126-5p	SH3BGR	SH3 domain binding glutamate rich protein
hsa-miR-126-5p	AP3B1	adaptor related protein complex 3 subunit beta 1
hsa-miR-126-5p	SLC7A14	solute carrier family 7 member 14
hsa-miR-126-5p	GABRA1	gamma-aminobutyric acid type A receptor alpha1 subunit
hsa-miR-126-5p	CCDC186	coiled-coil domain containing 186
hsa-miR-126-5p	HOXB2	homeobox B2
hsa-miR-126-5p	BLZF1	basic leucine zipper nuclear factor 1
hsa-miR-126-5p	HOXA13	homeobox A13
hsa-miR-126-5p	MCHR2	melanin concentrating hormone receptor 2
hsa-miR-126-5p	SCEL	sciellin
hsa-miR-126-5p	WRB-SH3BGR	WRB-SH3BGR readthrough
hsa-miR-126-5p	QPRT	quinolinate phosphoribosyltransferase
hsa-miR-126-5p	NFXL1	nuclear transcription factor, X-box binding like 1
hsa-miR-126-5p	CNOT4	CCR4-NOT transcription complex subunit 4
hsa-miR-126-5p	KMT5A	lysine methyltransferase 5A
hsa-miR-126-5p	QRSL1	QRSL1, glutaminyl-tRNA amidotransferase subunit A
hsa-miR-126-5p	GASK1B	golgi associated kinase 1B
hsa-miR-126-5p	RECK	reversion inducing cysteine rich protein with kazal motifs
hsa-miR-126-5p	WDHD1	WD repeat and HMG-box DNA binding protein 1
hsa-miR-126-5p	PDLIM5	PDZ and LIM domain 5
hsa-miR-126-5p	RASA2	RAS p21 protein activator 2
hsa-miR-126-5p	PIAS2	protein inhibitor of activated STAT 2
hsa-miR-126-5p	CDC14B	cell division cycle 14B
hsa-miR-126-5p	AK2	adenylate kinase 2
hsa-miR-126-5p	ERICH4	glutamate rich 4
hsa-miR-126-5p	CHIC1	cysteine rich hydrophobic domain 1
hsa-miR-126-5p	KRAS	KRAS proto-oncogene, GTPase
hsa-miR-126-5p	KLHL32	kelch like family member 32
hsa-miR-126-5p	ABCA5	ATP binding cassette subfamily A member 5

hsa-miR-126-5p	SHISA3	shisa family member 3
hsa-miR-126-5p	SLC2A11	solute carrier family 2 member 11
hsa-miR-126-5p	SRFBP1	serum response factor binding protein 1
hsa-miR-126-5p	BDH2	3-hydroxybutyrate dehydrogenase 2
hsa-miR-126-5p	EGFL6	EGF like domain multiple 6
hsa-miR-126-5p	GRM8	glutamate metabotropic receptor 8
hsa-miR-126-5p	PTPN12	protein tyrosine phosphatase, non-receptor type 12
hsa-miR-126-5p	RP2	RP2, ARL3 GTPase activating protein
hsa-miR-126-5p	KAT2B	lysine acetyltransferase 2B
hsa-miR-126-5p	EIF4A2	eukaryotic translation initiation factor 4A2
hsa-miR-126-5p	SF3A1	splicing factor 3a subunit 1
hsa-miR-126-5p	CSF1	colony stimulating factor 1
hsa-miR-126-5p	DGKI	diacylglycerol kinase iota
hsa-miR-126-5p	PWWP2A	PWWP domain containing 2A
hsa-miR-126-5p	IKZF2	IKAROS family zinc finger 2
hsa-miR-126-5p	RBM41	RNA binding motif protein 41
hsa-miR-126-5p	AHR	aryl hydrocarbon receptor
hsa-miR-126-5p	LRRTM3	leucine rich repeat transmembrane neuronal 3
hsa-miR-126-5p	PHKB	phosphorylase kinase regulatory subunit beta
hsa-miR-126-5p	HSD11B1	hydroxysteroid 11-beta dehydrogenase 1
hsa-miR-126-5p	FPGT	fucose-1-phosphate guanylyltransferase
hsa-miR-126-5p	DCAF13	DDB1 and CUL4 associated factor 13
hsa-miR-126-5p	ZNF33B	zinc finger protein 33B
hsa-miR-126-5p	FABP3	fatty acid binding protein 3
hsa-miR-126-5p	WASHC4	WASH complex subunit 4
hsa-miR-126-5p	GOSR1	golgi SNAP receptor complex member 1
hsa-miR-126-5p	REPS2	RALBP1 associated Eps domain containing 2
hsa-miR-126-5p	SP5	Sp5 transcription factor
hsa-miR-126-5p	MAATS1	MYCBP associated and testis expressed 1
hsa-miR-126-5p	PAX2	paired box 2
hsa-miR-126-5p	ABCD3	ATP binding cassette subfamily D member 3
hsa-miR-126-5p	PATE1	prostate and testis expressed 1
hsa-miR-126-5p	CHURC1	churchill domain containing 1
hsa-miR-126-5p	TMEM33	transmembrane protein 33
hsa-miR-126-5p	SLC41A2	solute carrier family 41 member 2
hsa-miR-126-5p	PEX13	peroxisomal biogenesis factor 13
hsa-miR-126-5p	HYDIN	HYDIN, axonemal central pair apparatus protein
hsa-miR-126-5p	PPP1R10	protein phosphatase 1 regulatory subunit 10
hsa-miR-126-5p	FGG	fibrinogen gamma chain
hsa-miR-126-5p	PMS1	PMS1 homolog 1, mismatch repair system component
hsa-miR-126-5p	ARID2	AT-rich interaction domain 2
hsa-miR-126-5p	TCHH	trichohyalin
hsa-miR-126-5p	FMC1-LUC7L2	FMC1-LUC7L2 readthrough
hsa-miR-126-5p	LUC7L2	LUC7 like 2, pre-mRNA splicing factor
hsa-miR-126-5p	DLL4	delta like canonical Notch ligand 4

hsa-miR-126-5p	SSBP3	single stranded DNA binding protein 3
hsa-miR-126-5p	C22orf39	chromosome 22 open reading frame 39
hsa-miR-126-5p	TRAF6	TNF receptor associated factor 6
hsa-miR-126-5p	TRDN	triadin
hsa-miR-126-5p	UTRN	utrophin
hsa-miR-126-5p	VWA8	von Willebrand factor A domain containing 8
hsa-miR-126-5p	PRPF8	pre-mRNA processing factor 8
hsa-miR-126-5p	SEMA6D	semaphorin 6D
hsa-miR-126-5p	CSF2RA	colony stimulating factor 2 receptor alpha subunit
hsa-miR-126-5p	JPT2	Jupiter microtubule associated homolog 2
hsa-miR-126-5p	EPOR	erythropoietin receptor
hsa-miR-126-5p	C21orf91	chromosome 21 open reading frame 91
hsa-miR-126-5p	ZIC2	Zic family member 2
hsa-miR-126-5p	PF4	platelet factor 4
hsa-miR-126-5p	GMPPB	GDP-mannose pyrophosphorylase B
hsa-miR-126-5p	FNDC9	fibronectin type III domain containing 9
hsa-miR-126-5p	TEP1	telomerase associated protein 1
hsa-miR-126-5p	APOPT1	apoptogenic 1, mitochondrial
hsa-miR-126-5p	THRB	thyroid hormone receptor beta
hsa-miR-126-5p	PTGS2	prostaglandin-endoperoxide synthase 2
hsa-miR-126-5p	HERC3	HECT and RLD domain containing E3 ubiquitin protein ligase 3
hsa-miR-126-5p	EFR3A	EFR3 homolog A
hsa-miR-126-5p	GINS1	GINS complex subunit 1
hsa-miR-126-5p	PRAMEF2	PRAME family member 2
hsa-miR-126-5p	AIMP1	aminoacyl tRNA synthetase complex interacting multifunctional protein 1
hsa-miR-126-5p	BCAP29	B cell receptor associated protein 29
hsa-miR-126-5p	DENND6A	DENN domain containing 6A
hsa-miR-126-5p	KIF14	kinesin family member 14
hsa-miR-126-5p	NRG1	neuregulin 1
hsa-miR-126-5p	ICK	intestinal cell kinase
hsa-miR-126-5p	VPS13C	vacuolar protein sorting 13 homolog C
hsa-miR-126-5p	CTNNA3	catenin alpha 3
hsa-miR-126-5p	KLHL7	kelch like family member 7
hsa-miR-126-5p	LRRC74B	leucine rich repeat containing 74B
hsa-miR-126-5p	UBA3	ubiquitin like modifier activating enzyme 3
hsa-miR-126-5p	MBLAC2	metallo-beta-lactamase domain containing 2
hsa-miR-126-5p	MED11	mediator complex subunit 11
hsa-miR-126-5p	ANTXR2	ANTXR cell adhesion molecule 2
hsa-miR-126-5p	ODR4	odr-4 GPCR localization factor homolog
hsa-miR-126-5p	RGS5	regulator of G protein signaling 5
hsa-miR-126-5p	PGAM4	phosphoglycerate mutase family member 4
hsa-miR-126-5p	CACNB4	calcium voltage-gated channel auxiliary subunit beta 4
hsa-miR-126-5p	P2RY12	purinergic receptor P2Y12
hsa-miR-126-5p	ZBTB20	zinc finger and BTB domain containing 20

hsa-miR-126-5p	ZNF333	zinc finger protein 333
hsa-miR-126-5p	CALHM3	calcium homeostasis modulator 3
hsa-miR-126-5p	SLC16A7	solute carrier family 16 member 7
hsa-miR-126-5p	KBTBD6	kelch repeat and BTB domain containing 6
hsa-miR-126-5p	IL7	interleukin 7
hsa-miR-126-5p	PLAG1	PLAG1 zinc finger
hsa-miR-126-5p	LTN1	listerin E3 ubiquitin protein ligase 1
hsa-miR-126-5p	PRKCA	protein kinase C alpha
hsa-miR-126-5p	FSIP1	fibrous sheath interacting protein 1
hsa-miR-126-5p	GPBP1	GC-rich promoter binding protein 1
hsa-miR-126-5p	PHF23	PHD finger protein 23
hsa-miR-126-5p	SLIT2	slit guidance ligand 2
hsa-miR-126-5p	ARID1A	AT-rich interaction domain 1A
hsa-miR-126-5p	ANTXR1	ANTXR cell adhesion molecule 1
hsa-miR-126-5p	FAM8A1	family with sequence similarity 8 member A1
hsa-miR-126-5p	DLK1	delta like non-canonical Notch ligand 1
hsa-miR-126-5p	FAR2	fatty acyl-CoA reductase 2
hsa-miR-126-5p	TMEM65	transmembrane protein 65
hsa-miR-126-5p	EXD2	exonuclease 3'-5' domain containing 2
hsa-miR-126-5p	SLC26A7	solute carrier family 26 member 7
hsa-miR-126-5p	ELK4	ELK4, ETS transcription factor
hsa-miR-126-5p	ELAVL3	ELAV like RNA binding protein 3
hsa-miR-126-5p	EPGN	epithelial mitogen
hsa-miR-126-5p	ZBTB7C	zinc finger and BTB domain containing 7C
hsa-miR-126-5p	FAXC	failed axon connections homolog
hsa-miR-126-5p	UBN2	ubinuclein 2
hsa-miR-126-5p	PTPN4	protein tyrosine phosphatase, non-receptor type 4
hsa-miR-126-5p	B4GALT4	beta-1,4-galactosyltransferase 4
hsa-miR-126-5p	PPP6R3	protein phosphatase 6 regulatory subunit 3
hsa-miR-126-5p	GABRA5	gamma-aminobutyric acid type A receptor alpha5 subunit
hsa-miR-126-5p	ZIC1	Zic family member 1
hsa-miR-126-5p	MEF2D	myocyte enhancer factor 2D
hsa-miR-126-5p	ZKSCAN8	zinc finger with KRAB and SCAN domains 8
hsa-miR-126-5p	HMCN1	hemicentin 1
hsa-miR-126-5p	TCP10L2	t-complex 10 like 2
hsa-miR-126-5p	ST8SIA3	ST8 alpha-N-acetyl-neuraminide alpha-2,8-sialyltransferase 3
hsa-miR-126-5p	CYS1	cystin 1
hsa-miR-126-5p	GRIN2A	glutamate ionotropic receptor NMDA type subunit 2A
hsa-miR-126-5p	TGFBI	transforming growth factor beta induced
hsa-miR-126-5p	CPEB4	cytoplasmic polyadenylation element binding protein 4
hsa-miR-126-5p	L3MBTL4	L3MBTL4, histone methyl-lysine binding protein
hsa-miR-126-5p	SGPP1	sphingosine-1-phosphate phosphatase 1
hsa-miR-126-5p	IAH1	isoamyl acetate hydrolyzing esterase 1 (putative)
hsa-miR-126-5p	ARL13B	ADP ribosylation factor like GTPase 13B
hsa-miR-126-5p	PPIF	peptidylprolyl isomerase F

hsa-miR-126-5p	CD2AP	CD2 associated protein
hsa-miR-126-5p	TRMT10A	tRNA methyltransferase 10A
hsa-miR-126-5p	COLEC12	collectin subfamily member 12
hsa-miR-126-5p	C17orf75	chromosome 17 open reading frame 75
hsa-miR-126-5p	DCDC2	doublecortin domain containing 2
hsa-miR-126-5p	PNLIPRP3	pancreatic lipase related protein 3
hsa-miR-126-5p	FAM199X	family with sequence similarity 199, X-linked
hsa-miR-126-5p	IFI44	interferon induced protein 44
hsa-miR-126-5p	LARP4	La ribonucleoprotein domain family member 4
hsa-miR-126-5p	ZNF10	zinc finger protein 10
hsa-miR-126-5p	MOG	myelin oligodendrocyte glycoprotein
hsa-miR-126-5p	PGC	progastricsin
hsa-miR-126-5p	CUBN	cubilin
hsa-miR-126-5p	OSTF1	osteoclast stimulating factor 1
hsa-miR-126-5p	MSR1	macrophage scavenger receptor 1
hsa-miR-126-5p	PPARGC1A	PPARG coactivator 1 alpha
hsa-miR-126-5p	COL11A1	collagen type XI alpha 1 chain
hsa-miR-126-5p	DAPP1	dual adaptor of phosphotyrosine and 3-phosphoinositides 1
hsa-miR-126-5p	NEGR1	neuronal growth regulator 1
hsa-miR-126-5p	KRT78	keratin 78
hsa-miR-126-5p	SMCHD1	structural maintenance of chromosomes flexible hinge domain containing 1
hsa-miR-126-5p	WNT3	Wnt family member 3
hsa-miR-126-5p	USP12	ubiquitin specific peptidase 12
hsa-miR-126-5p	NACC2	NACC family member 2
hsa-miR-126-5p	LETM2	leucine zipper and EF-hand containing transmembrane protein 2
hsa-miR-126-5p	GSK3B	glycogen synthase kinase 3 beta
hsa-miR-126-5p	STX17	syntaxin 17
hsa-miR-126-5p	CDK19	cyclin dependent kinase 19
hsa-miR-126-5p	FAM129A	family with sequence similarity 129 member A
hsa-miR-126-5p	PEX5L	peroxisomal biogenesis factor 5 like
hsa-miR-126-5p	TGFBR1	transforming growth factor beta receptor 1
hsa-miR-126-5p	RSPO3	R-spondin 3
hsa-miR-126-5p	DNAJB4	DnaJ heat shock protein family (Hsp40) member B4
hsa-miR-126-5p	LRRC3B	leucine rich repeat containing 3B
hsa-miR-126-5p	TANK	TRAF family member associated NFKB activator
hsa-miR-126-5p	PAN3	poly(A) specific ribonuclease subunit PAN3
hsa-miR-126-5p	LIN9	lin-9 DREAM MuvB core complex component
hsa-miR-126-5p	GRB2	growth factor receptor bound protein 2
hsa-miR-126-5p	DKK1	dickkopf WNT signaling pathway inhibitor 1
hsa-miR-126-5p	CTBS	chitobiase
hsa-miR-126-5p	SPRY4	sprouty RTK signaling antagonist 4
hsa-miR-126-5p	INAFM2	InaF motif containing 2
hsa-miR-126-5p	MAP3K13	mitogen-activated protein kinase kinase kinase 13
hsa-miR-126-5p	WASHC3	WASH complex subunit 3

hsa-miR-126-5p	EIF3J	eukaryotic translation initiation factor 3 subunit J
hsa-miR-126-5p	LRCH2	leucine rich repeats and calponin homology domain containing 2
hsa-miR-126-5p	CALCB	calcitonin related polypeptide beta
hsa-miR-126-5p	KPNA4	karyopherin subunit alpha 4
hsa-miR-126-5p	UNC5D	unc-5 netrin receptor D
hsa-miR-126-5p	DMBX1	diencephalon/mesencephalon homeobox 1
hsa-miR-126-5p	KCNV1	potassium voltage-gated channel modifier subfamily V member 1
hsa-miR-126-5p	UGT2B17	UDP glucuronosyltransferase family 2 member B17
hsa-miR-126-5p	MAN1A1	mannosidase alpha class 1A member 1
hsa-miR-126-5p	NXT2	nuclear transport factor 2 like export factor 2
hsa-miR-126-5p	KIAA1549L	KIAA1549 like
hsa-miR-126-5p	GPSM1	G protein signaling modulator 1
hsa-miR-126-5p	SLITRK4	SLIT and NTRK like family member 4
hsa-miR-126-5p	ODAPH	odontogenesis associated phosphoprotein
hsa-miR-126-5p	RGS4	regulator of G protein signaling 4
hsa-miR-126-5p	ITGB6	integrin subunit beta 6
hsa-miR-126-5p	TAOK3	TAO kinase 3
hsa-miR-126-5p	NLK	nemo like kinase
hsa-miR-126-5p	ZBTB18	zinc finger and BTB domain containing 18
hsa-miR-126-5p	ACVR2B	activin A receptor type 2B
hsa-miR-126-5p	CREB1	cAMP responsive element binding protein 1
hsa-miR-126-5p	ENOX2	ecto-NOX disulfide-thiol exchanger 2
hsa-miR-126-5p	ADCYAP1	adenylate cyclase activating polypeptide 1
hsa-miR-126-5p	PRAMEF13	PRAME family member 13
hsa-miR-126-5p	GLE1	GLE1, RNA export mediator
hsa-miR-126-5p	TMX4	thioredoxin related transmembrane protein 4
hsa-miR-126-5p	FAM78A	family with sequence similarity 78 member A
hsa-miR-126-5p	RAB31	RAB31, member RAS oncogene family
hsa-miR-126-5p	WDR64	WD repeat domain 64
hsa-miR-126-5p	PPP1R1C	protein phosphatase 1 regulatory inhibitor subunit 1C
hsa-miR-126-5p	PRAMEF14	PRAME family member 14
hsa-miR-126-5p	RSBN1	round spermatid basic protein 1
hsa-miR-126-5p	NHLRC2	NHL repeat containing 2
hsa-miR-126-5p	PRAMEF1	PRAME family member 1
hsa-miR-126-5p	ADGRG2	adhesion G protein-coupled receptor G2
hsa-miR-126-5p	ZDHHC20	zinc finger DHHC-type containing 20
hsa-miR-126-5p	CAVIN2	caveolae associated protein 2
hsa-miR-126-5p	DPY19L2	dpy-19 like 2
hsa-miR-126-5p	KMT2A	lysine methyltransferase 2A
hsa-miR-126-5p	NDUFAF3	NADH:ubiquinone oxidoreductase complex assembly factor 3
hsa-miR-126-5p	LRRC8D	leucine rich repeat containing 8 VRAC subunit D
hsa-miR-126-5p	HPSE	heparanase
hsa-miR-126-5p	DICER1	dicer 1, ribonuclease III
hsa-miR-126-5p	OCLN	occludin
hsa-miR-126-5p	IMPAD1	inositol monophosphatase domain containing 1

hsa-miR-126-5p	MLPH	melanophilin
hsa-miR-126-5p	EPS15	epidermal growth factor receptor pathway substrate 15
hsa-miR-126-5p	UHRF1BP1L	UHRF1 binding protein 1 like
hsa-miR-126-5p	CA1	carbonic anhydrase 1
hsa-miR-126-5p	RXFP1	relaxin family peptide receptor 1
hsa-miR-126-5p	XIRP2	xin actin binding repeat containing 2
hsa-miR-126-5p	PLIN1	perilipin 1
hsa-miR-126-5p	RTN4RL1	reticulon 4 receptor like 1
hsa-miR-126-5p	FRMPD4	FERM and PDZ domain containing 4
hsa-miR-126-5p	USP10	ubiquitin specific peptidase 10
hsa-miR-126-5p	SLC19A2	solute carrier family 19 member 2
hsa-miR-126-5p	PRXL2A	peroxiredoxin like 2A
hsa-miR-126-5p	ZNF33A	zinc finger protein 33A
hsa-miR-126-5p	MORC1	MORC family CW-type zinc finger 1
hsa-miR-126-5p	MMS22L	MMS22 like, DNA repair protein
hsa-miR-126-5p	PCDH7	protocadherin 7
hsa-miR-126-5p	HPN	hepsin
hsa-miR-126-5p	UBE3C	ubiquitin protein ligase E3C
hsa-miR-126-5p	C6orf136	chromosome 6 open reading frame 136
hsa-miR-126-5p	AHCTF1	AT-hook containing transcription factor 1
hsa-miR-126-5p	COMMD10	COMM domain containing 10
hsa-miR-126-5p	WDR5B	WD repeat domain 5B
hsa-miR-126-5p	BRAT1	BRCA1 associated ATM activator 1
hsa-miR-126-5p	CHMP3	charged multivesicular body protein 3
hsa-miR-126-5p	MSRB3	methionine sulfoxide reductase B3
hsa-miR-126-5p	TAF9	TATA-box binding protein associated factor 9
hsa-miR-126-5p	GTF2F1	general transcription factor IIF subunit 1
hsa-miR-126-5p	SLC6A15	solute carrier family 6 member 15
hsa-miR-126-5p	ANKRD20A1	ankyrin repeat domain 20 family member A1
hsa-miR-126-5p	HMGXB4	HMG-box containing 4
hsa-miR-126-5p	SNRPN	small nuclear ribonucleoprotein polypeptide N
hsa-miR-126-5p	LONRF2	LON peptidase N-terminal domain and ring finger 2
hsa-miR-126-5p	CCDC149	coiled-coil domain containing 149
hsa-miR-126-5p	TNFSF4	TNF superfamily member 4
hsa-miR-126-5p	SOGA1	suppressor of glucose, autophagy associated 1
hsa-miR-126-5p	CCNT2	cyclin T2
hsa-miR-126-5p	NF1	neurofibromin 1
hsa-miR-126-5p	AASDHPPT	aminoadipate-semialdehyde dehydrogenase-phosphopantetheinyl transferase
hsa-miR-126-5p	ANKRD20A4	ankyrin repeat domain 20 family member A4
hsa-miR-126-5p	RNF103-CHMP3	RNF103-CHMP3 readthrough
hsa-miR-126-5p	POFUT1	protein O-fucosyltransferase 1
hsa-miR-126-5p	MINDY2	MINDY lysine 48 deubiquitinase 2
hsa-miR-126-5p	DUSP28	dual specificity phosphatase 28
hsa-miR-126-5p	MAP3K7	mitogen-activated protein kinase kinase kinase 7

hsa-miR-126-5p	ANKRD20A2	ankyrin repeat domain 20 family member A2
hsa-miR-126-5p	C21orf62	chromosome 21 open reading frame 62
hsa-miR-126-5p	RASEF	RAS and EF-hand domain containing
hsa-miR-126-5p	NXT1	nuclear transport factor 2 like export factor 1
hsa-miR-126-5p	SMS	spermine synthase
hsa-miR-126-5p	REG3G	regenerating family member 3 gamma
hsa-miR-126-5p	MINAR1	membrane integral NOTCH2 associated receptor 1
hsa-miR-126-5p	CHUK	conserved helix-loop-helix ubiquitous kinase
hsa-miR-126-5p	STARD4	StAR related lipid transfer domain containing 4
hsa-miR-126-5p	CLEC12B	C-type lectin domain family 12 member B
hsa-miR-126-5p	VGLL1	vestigial like family member 1
hsa-miR-126-5p	CCDC14	coiled-coil domain containing 14
hsa-miR-126-5p	CDK13	cyclin dependent kinase 13
hsa-miR-126-5p	IFFO1	intermediate filament family orphan 1
hsa-miR-126-5p	STRN	striatin
hsa-miR-126-5p	AKAP4	A-kinase anchoring protein 4
hsa-miR-126-5p	GATA3	GATA binding protein 3
hsa-miR-126-5p	ZFAND5	zinc finger AN1-type containing 5
hsa-miR-126-5p	CASP3	caspase 3
hsa-miR-126-5p	STK17B	serine/threonine kinase 17b
hsa-miR-126-5p	PHC3	polyhomeotic homolog 3
hsa-miR-126-5p	TMEM266	transmembrane protein 266
hsa-miR-126-5p	LMO4	LIM domain only 4
hsa-miR-126-5p	C2orf69	chromosome 2 open reading frame 69
hsa-miR-126-5p	TOB1	transducer of ERBB2, 1
hsa-miR-126-5p	NR1D2	nuclear receptor subfamily 1 group D member 2
hsa-miR-126-5p	SFRP4	secreted frizzled related protein 4
hsa-miR-126-5p	NUDT12	nudix hydrolase 12
hsa-miR-126-5p	PHLPP1	PH domain and leucine rich repeat protein phosphatase 1
hsa-miR-126-5p	NHSL1	NHS like 1
hsa-miR-126-5p	IL17D	interleukin 17D
hsa-miR-126-5p	ASCC3	activating signal cointegrator 1 complex subunit 3
hsa-miR-126-5p	BAIAP2L1	BAI1 associated protein 2 like 1
hsa-miR-126-5p	FGF2	fibroblast growth factor 2
hsa-miR-126-5p	SELENOT	selenoprotein T
hsa-miR-126-5p	LACC1	laccase domain containing 1
hsa-miR-126-5p	LOC100130451	uncharacterized LOC100130451
hsa-miR-126-5p	TFEC	transcription factor EC
hsa-miR-126-5p	POLR2K	RNA polymerase II subunit K
hsa-miR-126-5p	ASTN1	astrotactin 1
hsa-miR-126-5p	GAS2L3	growth arrest specific 2 like 3
hsa-miR-126-5p	TMEM209	transmembrane protein 209
hsa-miR-126-5p	GALNT4	polypeptide N-acetylgalactosaminyltransferase 4
hsa-miR-126-5p	MED14	mediator complex subunit 14
hsa-miR-126-5p	FKTN	fukutin

hsa-miR-126-5p	KLF17	Kruppel like factor 17
hsa-miR-126-5p	AFG1L	AFG1 like ATPase
hsa-miR-126-5p	GRIA2	glutamate ionotropic receptor AMPA type subunit 2
hsa-miR-126-5p	USP47	ubiquitin specific peptidase 47
hsa-miR-126-5p	ZRANB1	zinc finger RANBP2-type containing 1
hsa-miR-126-5p	SSX2IP	SSX family member 2 interacting protein
hsa-miR-126-5p	POC1B-GALNT4	POC1B-GALNT4 readthrough
hsa-miR-126-5p	SYS1	SYS1, golgi trafficking protein
hsa-miR-126-5p	DAP3	death associated protein 3
hsa-miR-126-5p	NSL1	NSL1, MIS12 kinetochore complex component
hsa-miR-126-5p	NCAPH	non-SMC condensin I complex subunit H
hsa-miR-126-5p	CADM2	cell adhesion molecule 2
hsa-miR-126-5p	C16orf72	chromosome 16 open reading frame 72
hsa-miR-126-5p	CEP126	centrosomal protein 126
hsa-miR-126-5p	SLC35A3	solute carrier family 35 member A3
hsa-miR-126-5p	BMP3	bone morphogenetic protein 3
hsa-miR-126-5p	FCHO2	FCH domain only 2
hsa-miR-126-5p	TMEM59	transmembrane protein 59
hsa-miR-126-5p	CRBN	cereblon
hsa-miR-126-5p	CADM1	cell adhesion molecule 1
hsa-miR-126-5p	DBR1	debranching RNA lariats 1
hsa-miR-126-5p	C11orf87	chromosome 11 open reading frame 87
hsa-miR-126-5p	ST8SIA4	ST8 alpha-N-acetyl-neuraminide alpha-2,8-sialyltransferase 4
hsa-miR-126-5p	NAPG	NSF attachment protein gamma
hsa-miR-126-5p	UPRT	uracil phosphoribosyltransferase homolog
hsa-miR-126-5p	CACNA1A	calcium voltage-gated channel subunit alpha1 A
hsa-miR-126-5p	SH3BGRL2	SH3 domain binding glutamate rich protein like 2
hsa-miR-126-5p	ACER3	alkaline ceramidase 3
hsa-miR-126-5p	EYA4	EYA transcriptional coactivator and phosphatase 4
hsa-miR-126-5p	SLC25A15	solute carrier family 25 member 15
hsa-miR-126-5p	LRCH1	leucine rich repeats and calponin homology domain containing 1
hsa-miR-126-5p	CEP170	centrosomal protein 170
hsa-miR-126-5p	GPATCH2L	G-patch domain containing 2 like
hsa-miR-126-5p	PLEKHH2	pleckstrin homology, MyTH4 and FERM domain containing H2
hsa-miR-126-5p	BTC	betacellulin
hsa-miR-126-5p	RASSF6	Ras association domain family member 6
hsa-miR-126-5p	MAP2	microtubule associated protein 2
hsa-miR-126-5p	PTPN22	protein tyrosine phosphatase, non-receptor type 22
hsa-miR-126-5p	SMIM11B	small integral membrane protein 11B
hsa-miR-126-5p	C10orf126	chromosome 10 open reading frame 126
hsa-miR-126-5p	HOMEZ	homeobox and leucine zipper encoding
hsa-miR-126-5p	FBN2	fibrillin 2
hsa-miR-126-5p	GLIPR1	GLI pathogenesis related 1
hsa-miR-126-5p	RO60	Ro60, Y RNA binding protein
hsa-miR-126-5p	ATP1A2	ATPase Na+/K+ transporting subunit alpha 2

hsa-miR-126-5p	CERS6	ceramide synthase 6
hsa-miR-126-5p	SOX13	SRY-box 13
hsa-miR-126-5p	C9orf72	chromosome 9 open reading frame 72
hsa-miR-126-5p	SDHAF3	succinate dehydrogenase complex assembly factor 3
hsa-miR-126-5p	ZKSCAN4	zinc finger with KRAB and SCAN domains 4
hsa-miR-126-5p	IL36G	interleukin 36 gamma
hsa-miR-126-5p	GFRAL	GDNF family receptor alpha like
hsa-miR-126-5p	TRIL	TLR4 interactor with leucine rich repeats
hsa-miR-126-5p	GRM7	glutamate metabotropic receptor 7
hsa-miR-126-5p	SMIM11A	small integral membrane protein 11A
hsa-miR-126-5p	ZNF148	zinc finger protein 148
hsa-miR-126-5p	MYBL1	MYB proto-oncogene like 1
hsa-miR-126-5p	CRLS1	cardiolipin synthase 1
hsa-miR-126-5p	EIF4A3	eukaryotic translation initiation factor 4A3
hsa-miR-126-5p	PLEKHB2	pleckstrin homology domain containing B2
hsa-miR-126-5p	HOOK3	hook microtubule tethering protein 3
hsa-miR-126-5p	IREB2	iron responsive element binding protein 2
hsa-miR-126-5p	CIAO2A	cytosolic iron-sulfur assembly component 2A
hsa-miR-126-5p	TNS2	tensin 2
hsa-miR-126-5p	TMTC1	transmembrane and tetratricopeptide repeat containing 1
hsa-miR-126-5p	SS18L1	SS18L1, nBAF chromatin remodeling complex subunit
hsa-miR-126-5p	EPHA5	EPH receptor A5
hsa-miR-126-5p	CPED1	cadherin like and PC-esterase domain containing 1
hsa-miR-126-5p	CDH20	cadherin 20
hsa-miR-126-5p	MSH4	mutS homolog 4
hsa-miR-126-5p	MDGA2	MAM domain containing glycosylphosphatidylinositol anchor 2
hsa-miR-126-5p	FXR1	FMR1 autosomal homolog 1
hsa-miR-126-5p	GNL1	G protein nucleolar 1 (putative)
hsa-miR-126-5p	NCK1	NCK adaptor protein 1
hsa-miR-126-5p	GUCY1A1	guanylate cyclase 1 soluble subunit alpha 1
hsa-miR-126-5p	HSPA4L	heat shock protein family A (Hsp70) member 4 like
hsa-miR-126-5p	MARCH3	membrane associated ring-CH-type finger 3
hsa-miR-126-5p	PPP1R12B	protein phosphatase 1 regulatory subunit 12B
hsa-miR-126-5p	PDZRN4	PDZ domain containing ring finger 4
hsa-miR-126-5p	IFT57	intraflagellar transport 57
hsa-miR-126-5p	GRM5	glutamate metabotropic receptor 5
hsa-miR-126-5p	KIRREL2	kirre like nephrin family adhesion molecule 2
hsa-miR-126-5p	ZBTB1	zinc finger and BTB domain containing 1
hsa-miR-126-5p	POLK	DNA polymerase kappa
hsa-miR-126-5p	ZNF777	zinc finger protein 777
hsa-miR-126-5p	TCTN1	tectonic family member 1
hsa-miR-126-5p	RABGEF1	RAB guanine nucleotide exchange factor 1
hsa-miR-126-5p	FEM1B	fem-1 homolog B
hsa-miR-126-5p	PTPN14	protein tyrosine phosphatase, non-receptor type 14
hsa-miR-126-5p	NUDT7	nudix hydrolase 7

hsa-miR-126-5p	TTC7B	tetratricopeptide repeat domain 7B
hsa-miR-126-5p	HELZ	helicase with zinc finger
hsa-miR-126-5p	MYOZ3	myozenin 3
hsa-miR-126-5p	SETD5	SET domain containing 5
hsa-miR-126-5p	GHR	growth hormone receptor
hsa-miR-126-5p	SMIM8	small integral membrane protein 8
hsa-miR-126-5p	RBM19	RNA binding motif protein 19
hsa-miR-126-5p	RPRD1A	regulation of nuclear pre-mRNA domain containing 1A
hsa-miR-126-5p	FYB1	FYN binding protein 1
hsa-miR-126-5p	EFCAB7	EF-hand calcium binding domain 7
hsa-miR-126-5p	PANK3	pantothenate kinase 3
hsa-miR-126-5p	TSHZ1	teashirt zinc finger homeobox 1
hsa-miR-126-5p	ZNF197	zinc finger protein 197
hsa-miR-126-5p	RCN2	reticulocalbin 2
hsa-miR-126-5p	DIP2B	disco interacting protein 2 homolog B
hsa-miR-126-5p	ESM1	endothelial cell specific molecule 1
hsa-miR-126-5p	UBIAD1	UbiA prenyltransferase domain containing 1
hsa-miR-126-5p	ABHD18	abhydrolase domain containing 18
hsa-miR-126-5p	E2F7	E2F transcription factor 7
hsa-miR-126-5p	WIPF2	WAS/WASL interacting protein family member 2
hsa-miR-126-5p	ZNF208	zinc finger protein 208
hsa-miR-126-5p	TNFAIP2	TNF alpha induced protein 2
hsa-miR-126-5p	CHAMP1	chromosome alignment maintaining phosphoprotein 1
hsa-miR-126-5p	PCDH10	protocadherin 10
hsa-miR-126-5p	VAPA	VAMP associated protein A
hsa-miR-126-5p	PF4V1	platelet factor 4 variant 1
hsa-miR-126-5p	ZNF624	zinc finger protein 624
hsa-miR-126-5p	ADAMTSL1	ADAMTS like 1
hsa-miR-126-5p	DDIAS	DNA damage induced apoptosis suppressor
hsa-miR-126-5p	MYD88	MYD88, innate immune signal transduction adaptor
hsa-miR-126-5p	ASAH2B	N-acylsphingosine amidohydrolase 2B
hsa-miR-126-5p	DBF4	DBF4 zinc finger
hsa-miR-126-5p	PHACTR2	phosphatase and actin regulator 2
hsa-miR-126-5p	ARHGAP20	Rho GTPase activating protein 20
hsa-miR-126-5p	DCAF10	DDB1 and CUL4 associated factor 10
hsa-miR-126-5p	SYT14	synaptotagmin 14
hsa-miR-126-5p	MRTFB	myocardin related transcription factor B
hsa-miR-126-5p	WWC1	WW and C2 domain containing 1
hsa-miR-126-5p	NECAB1	N-terminal EF-hand calcium binding protein 1
hsa-miR-126-5p	EFCAB11	EF-hand calcium binding domain 11
hsa-miR-126-5p	EVI2A	ecotropic viral integration site 2A
hsa-miR-126-5p	EXOC5	exocyst complex component 5
hsa-miR-126-5p	ASPH	aspartate beta-hydroxylase
hsa-miR-126-5p	SLC25A30	solute carrier family 25 member 30
hsa-miR-126-5p	SNAI2	snail family transcriptional repressor 2

hsa-miR-126-5p	SRGAP2B	SLIT-ROBO Rho GTPase activating protein 2B
hsa-miR-126-5p	ANO5	anoctamin 5
hsa-miR-126-5p	METTL15	methyltransferase like 15
hsa-miR-126-5p	DNAJC3	DnaJ heat shock protein family (Hsp40) member C3
hsa-miR-126-5p	MBOAT1	membrane bound O-acyltransferase domain containing 1
hsa-miR-126-5p	PUS10	pseudouridine synthase 10
hsa-miR-126-5p	ANGPTL1	angiopoietin like 1
hsa-miR-126-5p	JAG1	jagged 1
hsa-miR-126-5p	LRRC55	leucine rich repeat containing 55
hsa-miR-126-5p	ZNF224	zinc finger protein 224
hsa-miR-126-5p	SNURF	SNRPN upstream reading frame
hsa-miR-126-5p	ZNF680	zinc finger protein 680
hsa-miR-126-5p	PPP2R2B	protein phosphatase 2 regulatory subunit Bbeta
hsa-miR-126-5p	C3orf80	chromosome 3 open reading frame 80
hsa-miR-126-5p	LAMTOR3	late endosomal/lysosomal adaptor, MAPK and MTOR activator 3
hsa-miR-126-5p	DPH6	diphthamine biosynthesis 6
hsa-miR-126-5p	GCLM	glutamate-cysteine ligase modifier subunit
hsa-miR-126-5p	NLGN4Y	neuroligin 4 Y-linked
hsa-miR-126-5p	GJB2	gap junction protein beta 2
hsa-miR-126-5p	SMIM25	small integral membrane protein 25
hsa-miR-126-5p	ITGB3BP	integrin subunit beta 3 binding protein
hsa-miR-126-5p	PTPRB	protein tyrosine phosphatase, receptor type B
hsa-miR-126-5p	TGS1	trimethylguanosine synthase 1
hsa-miR-126-5p	SNX14	sorting nexin 14
hsa-miR-126-5p	KCNMA1	potassium calcium-activated channel subfamily M alpha 1
hsa-miR-126-5p	OGT	O-linked N-acetylglucosamine (GlcNAc) transferase
hsa-miR-126-5p	MPC1	mitochondrial pyruvate carrier 1
hsa-miR-126-5p	COL12A1	collagen type XII alpha 1 chain
hsa-miR-126-5p	RUNX1T1	RUNX1 translocation partner 1
hsa-miR-126-5p	DAB2IP	DAB2 interacting protein
hsa-miR-126-5p	FMO2	flavin containing monooxygenase 2
hsa-miR-126-5p	AP1G1	adaptor related protein complex 1 subunit gamma 1
hsa-miR-126-5p	TMCO4	transmembrane and coiled-coil domains 4
hsa-miR-126-5p	BAG3	BCL2 associated athanogene 3
hsa-miR-126-5p	GPR88	G protein-coupled receptor 88
hsa-miR-126-5p	COBLL1	cordon-bleu WH2 repeat protein like 1
hsa-miR-126-5p	BARD1	BRCA1 associated RING domain 1
hsa-miR-126-5p	ABRA	actin binding Rho activating protein
hsa-miR-126-5p	CHCHD3	coiled-coil-helix-coiled-coil-helix domain containing 3
hsa-miR-126-5p	HRH4	histamine receptor H4
hsa-miR-126-5p	ADAMTS6	ADAM metallopeptidase with thrombospondin type 1 motif 6
hsa-miR-126-5p	ZNF354C	zinc finger protein 354C
hsa-miR-126-5p	ZNF407	zinc finger protein 407
hsa-miR-126-5p	ANK3	ankyrin 3
hsa-miR-126-5p	EBPL	EBP like

hsa-miR-126-5p	LDB1	LIM domain binding 1
hsa-miR-126-5p	C2	complement C2
hsa-miR-126-5p	RBM26	RNA binding motif protein 26
hsa-miR-126-5p	SREK1	splicing regulatory glutamic acid and lysine rich protein 1
hsa-miR-126-5p	AGTR2	angiotensin II receptor type 2
hsa-miR-126-5p	STC1	stanniocalcin 1
hsa-miR-126-5p	CHD1L	chromodomain helicase DNA binding protein 1 like
hsa-miR-126-5p	TSG101	tumor susceptibility 101
hsa-miR-126-5p	SPOUT1	SPOUT domain containing methyltransferase 1
hsa-miR-126-5p	MIPOL1	mirror-image polydactyly 1
hsa-miR-126-5p	ATG3	autophagy related 3
hsa-miR-126-5p	SPATA6	spermatogenesis associated 6
hsa-miR-126-5p	RAB11FIP2	RAB11 family interacting protein 2
hsa-miR-126-5p	DOCK7	dedicator of cytokinesis 7
hsa-miR-126-5p	BRAF	B-Raf proto-oncogene, serine/threonine kinase
hsa-miR-126-5p	MCTS1	MCTS1, re-initiation and release factor
hsa-miR-126-5p	TRAPPC13	trafficking protein particle complex 13
hsa-miR-126-5p	PCSK2	proprotein convertase subtilisin/kexin type 2
hsa-miR-126-5p	PCLO	piccolo presynaptic cytomatrix protein
hsa-miR-126-5p	SCAP	SREBF chaperone
hsa-miR-126-5p	SLC7A4	solute carrier family 7 member 4
hsa-miR-126-5p	PALM2	paralemmin 2
hsa-miR-126-5p	ALDH1L2	aldehyde dehydrogenase 1 family member L2
hsa-miR-126-5p	AKIRIN1	akirin 1
hsa-miR-126-5p	MRPL57	mitochondrial ribosomal protein L57
hsa-miR-126-5p	POU2F1	POU class 2 homeobox 1
hsa-miR-126-5p	AASS	aminoadipate-semialdehyde synthase
hsa-miR-126-5p	CALCRL	calcitonin receptor like receptor
hsa-miR-126-5p	CNEP1R1	CTD nuclear envelope phosphatase 1 regulatory subunit 1
hsa-miR-126-5p	RAB22A	RAB22A, member RAS oncogene family
hsa-miR-126-5p	DNAH5	dynein axonemal heavy chain 5
hsa-miR-126-5p	PCSK5	proprotein convertase subtilisin/kexin type 5
hsa-miR-126-5p	PPAT	phosphoribosyl pyrophosphate amidotransferase
hsa-miR-126-5p	NR3C1	nuclear receptor subfamily 3 group C member 1
hsa-miR-126-5p	GLCE	glucuronic acid epimerase
hsa-miR-126-5p	NEUROG2	neurogenin 2
hsa-miR-126-5p	RC3H1	ring finger and CCCH-type domains 1
hsa-miR-126-5p	PTPN11	protein tyrosine phosphatase, non-receptor type 11
hsa-miR-126-5p	MCM5	minichromosome maintenance complex component 5
hsa-miR-126-5p	CCDC179	coiled-coil domain containing 179
hsa-miR-126-5p	TTC9	tetratricopeptide repeat domain 9
hsa-miR-126-5p	GPATCH8	G-patch domain containing 8
hsa-miR-126-5p	CDYL2	chromodomain Y like 2
hsa-miR-126-5p	CLEC19A	C-type lectin domain containing 19A
hsa-miR-126-5p	SLC33A1	solute carrier family 33 member 1

hsa-miR-126-5p	KIAA0355	KIAA0355
hsa-miR-126-5p	EEF1AKMT2	EEF1A lysine methyltransferase 2
hsa-miR-126-5p	TCEANC2	transcription elongation factor A N-terminal and central domain containing 2
hsa-miR-126-5p	TET1	tet methylcytosine dioxygenase 1
hsa-miR-126-5p	NDUFA5	NADH:ubiquinone oxidoreductase subunit A5
hsa-miR-126-5p	DYNLT3	dynein light chain Tctex-type 3
hsa-miR-126-5p	LRP1	LDL receptor related protein 1
hsa-miR-126-5p	ZC3H12B	zinc finger CCCH-type containing 12B
hsa-miR-126-5p	ZFC3H1	zinc finger C3H1-type containing
hsa-miR-126-5p	USP45	ubiquitin specific peptidase 45
hsa-miR-126-5p	TNIK	TRAF2 and NCK interacting kinase
hsa-miR-126-5p	RHOBTB3	Rho related BTB domain containing 3
hsa-miR-126-5p	DISC1	DISC1 scaffold protein
hsa-miR-126-5p	PLA2G12A	phospholipase A2 group XIIA
hsa-miR-126-5p	ANKIB1	ankyrin repeat and IBR domain containing 1
hsa-miR-126-5p	FZD3	frizzled class receptor 3
hsa-miR-126-5p	KIF13A	kinesin family member 13A
hsa-miR-126-5p	NOX1	NADPH oxidase 1
hsa-miR-126-5p	HNRNPU	heterogeneous nuclear ribonucleoprotein U
hsa-miR-126-5p	CRIPT	CXXC repeat containing interactor of PDZ3 domain
hsa-miR-126-5p	DTWD2	DTW domain containing 2
hsa-miR-126-5p	LOC100144595	uncharacterized LOC100144595
hsa-miR-126-5p	SELENOI	selenoprotein I
hsa-miR-126-5p	IL1F10	interleukin 1 family member 10
hsa-miR-126-5p	TMEM64	transmembrane protein 64
hsa-miR-126-5p	NFIB	nuclear factor I B
hsa-miR-126-5p	CR1	complement C3b/C4b receptor 1 (Knops blood group)
hsa-miR-126-5p	LPIN2	lipin 2
hsa-miR-126-5p	CCDC88A	coiled-coil domain containing 88A
hsa-miR-126-5p	ELOVL2	ELOVL fatty acid elongase 2
hsa-miR-126-5p	ZBTB7B	zinc finger and BTB domain containing 7B
hsa-miR-126-5p	TIMM29	translocase of inner mitochondrial membrane 29
hsa-miR-126-5p	ZNF280D	zinc finger protein 280D
hsa-miR-126-5p	FLT1	fms related tyrosine kinase 1
hsa-miR-126-5p	EPB41L4B	erythrocyte membrane protein band 4.1 like 4B
hsa-miR-126-5p	PPIL6	peptidylprolyl isomerase like 6
hsa-miR-126-5p	FGF1	fibroblast growth factor 1
hsa-miR-126-5p	SLC25A12	solute carrier family 25 member 12
hsa-miR-126-5p	RAPGEF3	Rap guanine nucleotide exchange factor 3
hsa-miR-126-5p	FMN2	formin 2
hsa-miR-126-5p	REEP3	receptor accessory protein 3
hsa-miR-126-5p	SLCO6A1	solute carrier organic anion transporter family member 6A1
hsa-miR-126-5p	ST6GALNAC5	ST6 N-acetylgalactosaminide alpha-2,6-sialyltransferase 5
hsa-miR-126-5p	TMEM19	transmembrane protein 19

hsa-miR-126-5p	ODF2L	outer dense fiber of sperm tails 2 like
hsa-miR-126-5p	SPCS3	signal peptidase complex subunit 3
hsa-miR-126-5p	RGPD4	RANBP2-like and GRIP domain containing 4
hsa-miR-126-5p	HBP1	HMG-box transcription factor 1
hsa-miR-126-5p	UTY	ubiquitously transcribed tetratricopeptide repeat containing, Y-linked
hsa-miR-126-5p	MED4	mediator complex subunit 4
hsa-miR-126-5p	PHF14	PHD finger protein 14
hsa-miR-126-5p	MIGA1	mitoguardin 1
hsa-miR-126-5p	SLC4A10	solute carrier family 4 member 10
hsa-miR-126-5p	SNX2	sorting nexin 2
hsa-miR-126-5p	ARPC3	actin related protein 2/3 complex subunit 3
hsa-miR-126-5p	PRTG	protogenin
hsa-miR-126-5p	TMX1	thioredoxin related transmembrane protein 1
hsa-miR-126-5p	SGIP1	SH3 domain GRB2 like endophilin interacting protein 1
hsa-miR-126-5p	RAB33B	RAB33B, member RAS oncogene family
hsa-miR-126-5p	UMPS	uridine monophosphate synthetase
hsa-miR-126-5p	SLC24A2	solute carrier family 24 member 2
hsa-miR-126-5p	LYPLAL1	lysophospholipase like 1
hsa-miR-126-5p	IPMK	inositol polyphosphate multikinase
hsa-miR-126-5p	ADAMTS4	ADAM metallopeptidase with thrombospondin type 1 motif 4
hsa-miR-126-5p	PITPNC1	phosphatidylinositol transfer protein cytoplasmic 1
hsa-miR-126-5p	DCLK1	doublecortin like kinase 1
hsa-miR-126-5p	ZNF326	zinc finger protein 326
hsa-miR-126-5p	UBE2V2	ubiquitin conjugating enzyme E2 V2
hsa-miR-126-5p	TEAD1	TEA domain transcription factor 1
hsa-miR-126-5p	RDH11	retinol dehydrogenase 11
hsa-miR-126-5p	PSD3	pleckstrin and Sec7 domain containing 3
hsa-miR-126-5p	OTULIN	OTU deubiquitinase with linear linkage specificity
hsa-miR-126-5p	PDE9A	phosphodiesterase 9A
hsa-miR-126-5p	LZTFL1	leucine zipper transcription factor like 1
hsa-miR-126-5p	PANX1	pannexin 1
hsa-miR-126-5p	CYP4F2	cytochrome P450 family 4 subfamily F member 2
hsa-miR-126-5p	AFF4	AF4/FMR2 family member 4
hsa-miR-126-5p	RGPD8	RANBP2-like and GRIP domain containing 8
hsa-miR-126-5p	TMEM170B	transmembrane protein 170B
hsa-miR-126-5p	METTL25	methyltransferase like 25
hsa-miR-126-5p	PSAT1	phosphoserine aminotransferase 1
hsa-miR-126-5p	UGT8	UDP glycosyltransferase 8
hsa-miR-126-5p	ANKRD20A3	ankyrin repeat domain 20 family member A3
hsa-miR-126-5p	SMC2	structural maintenance of chromosomes 2
hsa-miR-126-5p	RGPD6	RANBP2-like and GRIP domain containing 6
hsa-miR-126-5p	TACO1	translational activator of cytochrome c oxidase I
hsa-miR-126-5p	ENPP5	ectonucleotide pyrophosphatase/phosphodiesterase 5 (putative)
hsa-miR-126-5p	OTUD6B	OTU domain containing 6B
hsa-miR-126-5p	SLC35G1	solute carrier family 35 member G1

hsa-miR-126-5p	SH3TC2	SH3 domain and tetratricopeptide repeats 2
hsa-miR-126-5p	NCOR1	nuclear receptor corepressor 1
hsa-miR-126-5p	DTX3L	deltex E3 ubiquitin ligase 3L
hsa-miR-126-5p	STAM2	signal transducing adaptor molecule 2
hsa-miR-126-5p	RGPD5	RANBP2-like and GRIP domain containing 5
hsa-miR-126-5p	MITF	melanocyte inducing transcription factor
hsa-miR-126-5p	FAM221A	family with sequence similarity 221 member A
hsa-miR-126-5p	TNFAIP3	TNF alpha induced protein 3
hsa-miR-126-5p	SOCS4	suppressor of cytokine signaling 4
hsa-miR-126-5p	LSAMP	limbic system associated membrane protein
hsa-miR-126-5p	PDGFD	platelet derived growth factor D
hsa-miR-126-5p	GK	glycerol kinase
hsa-miR-126-5p	TBC1D20	TBC1 domain family member 20
hsa-miR-126-5p	CLHC1	clathrin heavy chain linker domain containing 1
hsa-miR-126-5p	PALLD	palladin, cytoskeletal associated protein
hsa-miR-126-5p	RAB3GAP2	RAB3 GTPase activating non-catalytic protein subunit 2
hsa-miR-126-5p	ATP6V1A	ATPase H+ transporting V1 subunit A
hsa-miR-126-5p	RAD51B	RAD51 paralog B
hsa-miR-126-5p	REV3L	REV3 like, DNA directed polymerase zeta catalytic subunit
hsa-miR-126-5p	FBXO33	F-box protein 33
hsa-miR-126-5p	CSNK1D	casein kinase 1 delta
hsa-miR-126-5p	ERBIN	erbb2 interacting protein
hsa-miR-126-5p	TMEM26	transmembrane protein 26
hsa-miR-126-5p	ITGB8	integrin subunit beta 8
hsa-miR-126-5p	TNFRSF19	TNF receptor superfamily member 19
hsa-miR-126-5p	FHL5	four and a half LIM domains 5
hsa-miR-126-5p	NLGN4X	neuroligin 4 X-linked
hsa-miR-126-5p	ATXN1L	ataxin 1 like
hsa-miR-126-5p	HSFX3	heat shock transcription factor family, X-linked member 3
hsa-miR-126-5p	ATG16L1	autophagy related 16 like 1
hsa-miR-126-5p	XRN1	5'-3' exoribonuclease 1
hsa-miR-126-5p	TENT4B	terminal nucleotidyltransferase 4B
hsa-miR-126-5p	RAB3IP	RAB3A interacting protein
hsa-miR-126-5p	TLL2	tolloid like 2
hsa-miR-126-5p	STAU2	staufen double-stranded RNA binding protein 2
hsa-miR-126-5p	CTCFL	CCCTC-binding factor like
hsa-miR-126-5p	GCG	glucagon
hsa-miR-126-5p	UAP1	UDP-N-acetylglucosamine pyrophosphorylase 1
hsa-miR-126-5p	TBC1D19	TBC1 domain family member 19
hsa-miR-126-5p	FBXO36	F-box protein 36
hsa-miR-126-5p	RNASEL	ribonuclease L
hsa-miR-126-5p	TMEM70	transmembrane protein 70
hsa-miR-126-5p	COLEC10	collectin subfamily member 10
hsa-miR-126-5p	DGKH	diacylglycerol kinase eta
hsa-miR-126-5p	SLC25A24	solute carrier family 25 member 24

hsa-miR-126-5p	IL15	interleukin 15
hsa-miR-126-5p	VGLL3	vestigial like family member 3
hsa-miR-126-5p	ZNF260	zinc finger protein 260
hsa-miR-126-5p	SPATA18	spermatogenesis associated 18
hsa-miR-126-5p	GRIA4	glutamate ionotropic receptor AMPA type subunit 4
hsa-miR-126-5p	MBTD1	mbt domain containing 1
hsa-miR-126-5p	ARL15	ADP ribosylation factor like GTPase 15
hsa-miR-126-5p	RAB27B	RAB27B, member RAS oncogene family
hsa-miR-126-5p	GNB4	G protein subunit beta 4
hsa-miR-126-5p	HBEGF	heparin binding EGF like growth factor
hsa-miR-126-5p	DNAH14	dynein axonemal heavy chain 14
hsa-miR-126-5p	MPP7	membrane palmitoylated protein 7
hsa-miR-126-5p	C11orf45	chromosome 11 open reading frame 45
hsa-miR-126-5p	EPHB1	EPH receptor B1
hsa-miR-126-5p	SYAP1	synapse associated protein 1
hsa-miR-126-5p	LACTB	lactamase beta
hsa-miR-126-5p	ALCAM	activated leukocyte cell adhesion molecule
hsa-miR-126-5p	TMEM47	transmembrane protein 47
hsa-miR-126-5p	TMEM30A	transmembrane protein 30A
hsa-miR-126-5p	RGS10	regulator of G protein signaling 10
hsa-miR-126-5p	SAA2	serum amyloid A2
hsa-miR-126-5p	NRXN3	neurexin 3
hsa-miR-126-5p	ZBTB6	zinc finger and BTB domain containing 6
hsa-miR-126-5p	NSUN7	NOP2/Sun RNA methyltransferase family member 7
hsa-miR-126-5p	MMGT1	membrane magnesium transporter 1
hsa-miR-126-5p	ZBTB8B	zinc finger and BTB domain containing 8B
hsa-miR-126-5p	C15orf41	chromosome 15 open reading frame 41
hsa-miR-126-5p	ANKRD33B	ankyrin repeat domain 33B
hsa-miR-126-5p	RBM46	RNA binding motif protein 46
hsa-miR-126-5p	TMEM100	transmembrane protein 100
hsa-miR-126-5p	RPS6KA6	ribosomal protein S6 kinase A6
hsa-miR-126-5p	ZMYM6	zinc finger MYM-type containing 6
hsa-miR-126-5p	UBE2H	ubiquitin conjugating enzyme E2 H
hsa-miR-126-5p	ARHGAP32	Rho GTPase activating protein 32
hsa-miR-126-5p	IL17F	interleukin 17F
hsa-miR-126-5p	UTS2B	urotensin 2B
hsa-miR-126-5p	MAN2A1	mannosidase alpha class 2A member 1
hsa-miR-126-5p	BZW1	basic leucine zipper and W2 domains 1
hsa-miR-126-5p	DDR1	discoidin domain receptor tyrosine kinase 1
hsa-miR-126-5p	SLMAP	sarcolemma associated protein
hsa-miR-126-5p	ADHFE1	alcohol dehydrogenase, iron containing 1
hsa-miR-126-5p	PRRX1	paired related homeobox 1
hsa-miR-126-5p	HMBOX1	homeobox containing 1
hsa-miR-126-5p	MRS2	magnesium transporter MRS2
hsa-miR-126-5p	CDC5L	cell division cycle 5 like

hsa-miR-126-5p	PSMB2	proteasome subunit beta 2
hsa-miR-126-5p	UFSP1	UFM1 specific peptidase 1 (inactive)
hsa-miR-126-5p	PRKD1	protein kinase D1
hsa-miR-126-5p	MOB1B	MOB kinase activator 1B
hsa-miR-126-5p	PIP4K2C	phosphatidylinositol-5-phosphate 4-kinase type 2 gamma
hsa-miR-126-5p	RASAL2	RAS protein activator like 2
hsa-miR-126-5p	MST1L	macrophage stimulating 1 like
hsa-miR-126-5p	ERO1A	endoplasmic reticulum oxidoreductase 1 alpha
hsa-miR-126-5p	TMEM135	transmembrane protein 135
hsa-miR-126-5p	ACADL	acyl-CoA dehydrogenase long chain
hsa-miR-126-5p	SOCS6	suppressor of cytokine signaling 6
hsa-miR-126-5p	DMXL1	Dmx like 1
hsa-miR-126-5p	FLRT2	fibronectin leucine rich transmembrane protein 2
hsa-miR-126-5p	SAE1	SUMO1 activating enzyme subunit 1
hsa-miR-126-5p	MBP	myelin basic protein
hsa-miR-126-5p	PRKX	protein kinase X-linked
hsa-miR-126-5p	MAP3K20	mitogen-activated protein kinase kinase kinase 20
hsa-miR-126-5p	DNAJB9	DnaJ heat shock protein family (Hsp40) member B9
hsa-miR-126-5p	RMDN1	regulator of microtubule dynamics 1
hsa-miR-126-5p	ZNF41	zinc finger protein 41
hsa-miR-126-5p	SRGAP2C	SLIT-ROBO Rho GTPase activating protein 2C
hsa-miR-126-5p	FAM216B	family with sequence similarity 216 member B
hsa-miR-126-5p	MAPKAPK5	mitogen-activated protein kinase-activated protein kinase 5
hsa-miR-126-5p	TMPRSS3	transmembrane serine protease 3
hsa-miR-126-5p	C9orf3	chromosome 9 open reading frame 3
hsa-miR-126-5p	ZSCAN9	zinc finger and SCAN domain containing 9
hsa-miR-126-5p	CCDC148	coiled-coil domain containing 148
hsa-miR-126-5p	LCORL	ligand dependent nuclear receptor corepressor like
hsa-miR-126-5p	MAPK13	mitogen-activated protein kinase 13
hsa-miR-126-5p	FZD2	frizzled class receptor 2
hsa-miR-126-5p	CFL2	cofilin 2
hsa-miR-126-5p	FRZB	frizzled related protein
hsa-miR-126-5p	NAA50	N(alpha)-acetyltransferase 50, NatE catalytic subunit
hsa-miR-126-5p	STS	steroid sulfatase
hsa-miR-126-5p	GOLGA7B	golgin A7 family member B
hsa-miR-126-5p	MMAA	metabolism of cobalamin associated A
hsa-miR-126-5p	PGM2L1	phosphoglucomutase 2 like 1
hsa-miR-126-5p	CSRNP3	cysteine and serine rich nuclear protein 3
hsa-miR-126-5p	CRNKL1	crooked neck pre-mRNA splicing factor 1
hsa-miR-126-5p	ELAVL2	ELAV like RNA binding protein 2
hsa-miR-126-5p	CTR9	CTR9 homolog, Paf1/RNA polymerase II complex component
hsa-miR-126-5p	MGP	matrix Gla protein
hsa-miR-126-5p	ABRAXAS2	abraxas 2, BRISC complex subunit
hsa-miR-126-5p	PIK3CA	phosphatidylinositol-4,5-bisphosphate 3-kinase catalytic subunit alpha
hsa-miR-126-5p	CYTIP	cytohesin 1 interacting protein

hsa-miR-126-5p	TTC3	tetratricopeptide repeat domain 3
hsa-miR-126-5p	FARP1	FERM, ARH/RhoGEF and pleckstrin domain protein 1
hsa-miR-126-5p	DCAF8L1	DDB1 and CUL4 associated factor 8 like 1
hsa-miR-126-5p	VAMP4	vesicle associated membrane protein 4
hsa-miR-126-5p	MFSD11	major facilitator superfamily domain containing 11
hsa-miR-126-5p	ARPC5L	actin related protein 2/3 complex subunit 5 like
hsa-miR-126-5p	CCDC36	coiled-coil domain containing 36
hsa-miR-126-5p	INO80D	INO80 complex subunit D
hsa-miR-126-5p	OGFRL1	opioid growth factor receptor like 1
hsa-miR-126-5p	GIMAP8	GTPase, IMAP family member 8
hsa-miR-126-5p	AMER2	APC membrane recruitment protein 2
hsa-miR-126-5p	C18orf25	chromosome 18 open reading frame 25
hsa-miR-126-5p	TMEM71	transmembrane protein 71
hsa-miR-126-5p	PELI2	pellino E3 ubiquitin protein ligase family member 2
hsa-miR-126-5p	RILPL1	Rab interacting lysosomal protein like 1
hsa-miR-126-5p	BRD1	bromodomain containing 1
hsa-miR-126-5p	DPPA4	developmental pluripotency associated 4
hsa-miR-126-5p	TMEM63B	transmembrane protein 63B
hsa-miR-126-5p	FSD1L	fibronectin type III and SPRY domain containing 1 like
hsa-miR-126-5p	TMEM9B	TMEM9 domain family member B
hsa-miR-126-5p	DOCK5	dedicator of cytokinesis 5
hsa-miR-126-5p	APLF	aprataxin and PNKP like factor
hsa-miR-126-5p	MED17	mediator complex subunit 17
hsa-miR-126-5p	BTLA	B and T lymphocyte associated
hsa-miR-126-5p	BTAF1	B-TFIID TATA-box binding protein associated factor 1
hsa-miR-126-5p	SH3RF1	SH3 domain containing ring finger 1
hsa-miR-126-5p	EPSTI1	epithelial stromal interaction 1
hsa-miR-126-5p	LOX	lysyl oxidase
hsa-miR-126-5p	FAM227A	family with sequence similarity 227 member A
hsa-miR-126-5p	DHX33	DEAH-box helicase 33
hsa-miR-126-5p	FNDC3A	fibronectin type III domain containing 3A
hsa-miR-126-5p	SRRM4	serine/arginine repetitive matrix 4
hsa-miR-126-5p	NCAPG	non-SMC condensin I complex subunit G
hsa-miR-126-5p	C12orf71	chromosome 12 open reading frame 71
hsa-miR-126-5p	SNCA	synuclein alpha
hsa-miR-126-5p	STON2	stonin 2
hsa-miR-126-5p	DCLK2	doublecortin like kinase 2
hsa-miR-126-5p	FABP7	fatty acid binding protein 7
hsa-miR-126-5p	PDGFRA	platelet derived growth factor receptor alpha
hsa-miR-126-5p	PNN	pinin, desmosome associated protein
hsa-miR-126-5p	YPEL2	yippee like 2
hsa-miR-126-5p	C8orf34	chromosome 8 open reading frame 34
hsa-miR-126-5p	DSEL	dermatan sulfate epimerase like
hsa-miR-126-5p	ERAP1	endoplasmic reticulum aminopeptidase 1
hsa-miR-126-5p	IVD	isovaleryl-CoA dehydrogenase

hsa-miR-126-5p	C1RL	complement C1r subcomponent like
hsa-miR-126-5p	KIAA1109	KIAA1109
hsa-miR-126-5p	MS4A1	membrane spanning 4-domains A1
hsa-miR-126-5p	HNRNPD	heterogeneous nuclear ribonucleoprotein D
hsa-miR-126-5p	ZNF627	zinc finger protein 627
hsa-miR-126-5p	HBS1L	HBS1 like translational GTPase
hsa-miR-126-5p	PHAX	phosphorylated adaptor for RNA export
hsa-miR-126-5p	SLC25A21	solute carrier family 25 member 21
hsa-miR-126-5p	C8orf37	chromosome 8 open reading frame 37
hsa-miR-126-5p	METTL2A	methyltransferase like 2A
hsa-miR-126-5p	NADK2	NAD kinase 2, mitochondrial
hsa-miR-126-5p	CEP41	centrosomal protein 41
hsa-miR-126-5p	RXRG	retinoid X receptor gamma
hsa-miR-126-5p	ANKRD42	ankyrin repeat domain 42
hsa-miR-126-5p	SECISBP2L	SECIS binding protein 2 like
hsa-miR-126-5p	CNTN4	contactin 4
hsa-miR-126-5p	POF1B	POF1B, actin binding protein
hsa-miR-126-5p	CLDN11	claudin 11
hsa-miR-126-5p	MARK1	microtubule affinity regulating kinase 1
hsa-miR-126-5p	SAMD8	sterile alpha motif domain containing 8
hsa-miR-126-5p	ATF6	activating transcription factor 6
hsa-miR-126-5p	FAM76B	family with sequence similarity 76 member B
hsa-miR-126-5p	ADAT2	adenosine deaminase, tRNA specific 2
hsa-miR-126-5p	GRAMD2B	GRAM domain containing 2B
hsa-miR-126-5p	PTCHD4	patched domain containing 4
hsa-miR-126-5p	C12orf40	chromosome 12 open reading frame 40
hsa-miR-126-5p	ACSM5	acyl-CoA synthetase medium chain family member 5
hsa-miR-126-5p	ZNF264	zinc finger protein 264
hsa-miR-126-5p	TBC1D9B	TBC1 domain family member 9B
hsa-miR-126-5p	DIO3	iodothyronine deiodinase 3
hsa-miR-126-5p	TMEM251	transmembrane protein 251
hsa-miR-126-5p	AGPAT5	1-acylglycerol-3-phosphate O-acyltransferase 5
hsa-miR-126-5p	CYP39A1	cytochrome P450 family 39 subfamily A member 1
hsa-miR-126-5p	TBX18	T-box 18
hsa-miR-126-5p	CNOT6	CCR4-NOT transcription complex subunit 6
hsa-miR-126-5p	EYS	eyes shut homolog
hsa-miR-126-5p	BCL11B	BCL11B, BAF complex component
hsa-miR-126-5p	FAM84B	family with sequence similarity 84 member B
hsa-miR-126-5p	TFPI2	tissue factor pathway inhibitor 2
hsa-miR-126-5p	AAK1	AP2 associated kinase 1
hsa-miR-126-5p	OPRK1	opioid receptor kappa 1
hsa-miR-126-5p	ANGPTL7	angiopoietin like 7
hsa-miR-126-5p	CLCN3	chloride voltage-gated channel 3
hsa-miR-126-5p	ZC3H6	zinc finger CCCH-type containing 6
hsa-miR-126-5p	TEX12	testis expressed 12

hsa-miR-126-5p	TCF12	transcription factor 12
hsa-miR-126-5p	HPCA	hippocalcin
hsa-miR-126-5p	SLC6A4	solute carrier family 6 member 4
hsa-miR-126-5p	NEUROD6	neuronal differentiation 6
hsa-miR-126-5p	TMPO	thymopoietin
hsa-miR-126-5p	RRP15	ribosomal RNA processing 15 homolog
hsa-miR-126-5p	SFT2D1	SFT2 domain containing 1
hsa-miR-126-5p	EMB	embigin
hsa-miR-126-5p	SPRED1	sprouty related EVH1 domain containing 1
hsa-miR-126-5p	SPICE1	spindle and centriole associated protein 1
hsa-miR-126-5p	ERCC6	ERCC excision repair 6, chromatin remodeling factor
hsa-miR-126-5p	TBL1XR1	transducin beta like 1 X-linked receptor 1
hsa-miR-126-5p	DTD2	D-tyrosyl-tRNA deacylase 2 (putative)
hsa-miR-126-5p	GRHL2	grainyhead like transcription factor 2
hsa-miR-126-5p	SLFN13	schlafen family member 13
hsa-miR-126-5p	ABCC9	ATP binding cassette subfamily C member 9
hsa-miR-126-5p	ZNF81	zinc finger protein 81
hsa-miR-126-5p	SBSPON	somatomedin B and thrombospondin type 1 domain containing
hsa-miR-126-5p	EXPH5	exophilin 5
hsa-miR-126-5p	sept-07	septin 7
hsa-miR-126-5p	BHLHE41	basic helix-loop-helix family member e41
hsa-miR-126-5p	C9orf153	chromosome 9 open reading frame 153
hsa-miR-126-5p	PTPN21	protein tyrosine phosphatase, non-receptor type 21
hsa-miR-126-5p	MMP20	matrix metallopeptidase 20
hsa-miR-126-5p	ZADH2	zinc binding alcohol dehydrogenase domain containing 2
hsa-miR-126-5p	DYNC1I2	dynein cytoplasmic 1 intermediate chain 2
hsa-miR-126-5p	SLC17A8	solute carrier family 17 member 8
hsa-miR-126-5p	PIH1D3	PIH1 domain containing 3
hsa-miR-126-5p	TRIM61	tripartite motif containing 61
hsa-miR-126-5p	HMGB1	high mobility group box 1
hsa-miR-126-5p	SMC1A	structural maintenance of chromosomes 1A
hsa-miR-126-5p	JRKL	JRK like
hsa-miR-126-5p	LOC102724951	uncharacterized LOC102724951
hsa-miR-126-5p	CEPT1	choline/ethanolamine phosphotransferase 1
hsa-miR-126-5p	TMEM168	transmembrane protein 168
hsa-miR-126-5p	ZDHHC21	zinc finger DHHC-type containing 21
hsa-miR-126-5p	MAGEF1	MAGE family member F1
hsa-miR-126-5p	HIPK3	homeodomain interacting protein kinase 3
hsa-miR-126-5p	GCC1	GRIP and coiled-coil domain containing 1
hsa-miR-126-5p	CPNE9	copine family member 9
hsa-miR-126-5p	DLG5	discs large MAGUK scaffold protein 5
hsa-miR-126-5p	AMMECR1L	AMMECR1 like
hsa-miR-126-5p	CDC37L1	cell division cycle 37 like 1
hsa-miR-126-5p	MELK	maternal embryonic leucine zipper kinase
hsa-miR-126-5p	CNTNAP2	contactin associated protein like 2

hsa-miR-126-5p	TSPO2	translocator protein 2
hsa-miR-126-5p	CRKL	CRK like proto-oncogene, adaptor protein
hsa-miR-126-5p	LOC102723360	uncharacterized LOC102723360
hsa-miR-126-5p	LOC102724219	uncharacterized LOC102724219
hsa-miR-126-5p	PGBD1	piggyBac transposable element derived 1
hsa-miR-126-5p	ATN1	atrophin 1
hsa-miR-126-5p	ZBTB38	zinc finger and BTB domain containing 38
hsa-miR-126-5p	ENO4	enolase 4
hsa-miR-126-5p	FBN1	fibrillin 1
hsa-miR-126-5p	PPIL1	peptidylprolyl isomerase like 1
hsa-miR-126-5p	CLDN22	claudin 22
hsa-miR-126-5p	LOC102724843	uncharacterized LOC102724843
hsa-miR-126-5p	PRUNE2	prune homolog 2 with BCH domain
hsa-miR-126-5p	FNTB	farnesyltransferase, CAAX box, beta
hsa-miR-126-5p	BRD3	bromodomain containing 3
hsa-miR-126-5p	TPD52L3	TPD52 like 3
hsa-miR-126-5p	TMEM245	transmembrane protein 245
hsa-miR-126-5p	GLUD1	glutamate dehydrogenase 1
hsa-miR-126-5p	CA13	carbonic anhydrase 13
hsa-miR-126-5p	VXN	vexin
hsa-miR-126-5p	TNC	tenascin C
hsa-miR-126-5p	GOLGA6D	golgin A6 family member D
hsa-miR-126-5p	CPNE8	copine 8
hsa-miR-126-5p	SNX4	sorting nexin 4
hsa-miR-126-5p	C7orf57	chromosome 7 open reading frame 57
hsa-miR-126-5p	TPD52L1	TPD52 like 1
hsa-miR-126-5p	MARF1	meiosis regulator and mRNA stability factor 1
hsa-miR-126-5p	HMGCR	3-hydroxy-3-methylglutaryl-CoA reductase
hsa-miR-126-5p	CHD9	chromodomain helicase DNA binding protein 9
hsa-miR-126-5p	IRX3	iroquois homeobox 3
hsa-miR-126-5p	sept-02	septin 2
hsa-miR-126-5p	SEC14L1	SEC14 like lipid binding 1
hsa-miR-126-5p	CDH19	cadherin 19
hsa-miR-126-5p	IGF2BP2	insulin like growth factor 2 mRNA binding protein 2
hsa-miR-126-5p	TM4SF18	transmembrane 4 L six family member 18
hsa-miR-126-5p	GLRB	glycine receptor beta
hsa-miR-126-5p	GTF2A1	general transcription factor IIA subunit 1
hsa-miR-126-5p	KLHL41	kelch like family member 41
hsa-miR-126-5p	FAT4	FAT atypical cadherin 4
hsa-miR-126-5p	ETS2	ETS proto-oncogene 2, transcription factor
hsa-miR-126-5p	ZNF607	zinc finger protein 607
hsa-miR-126-5p	TRAF3	TNF receptor associated factor 3
hsa-miR-126-5p	TNKS2	tankyrase 2
hsa-miR-126-5p	HECW2	HECT, C2 and WW domain containing E3 ubiquitin protein ligase 2
hsa-miR-126-5p	F5	coagulation factor V

hsa-miR-126-5p	TMEM189	transmembrane protein 189
hsa-miR-126-5p	BTBD1	BTB domain containing 1
hsa-miR-126-5p	LRRK2	leucine rich repeat kinase 2
hsa-miR-126-5p	CXADR	CXADR, Ig-like cell adhesion molecule
hsa-miR-126-5p	PLSCR4	phospholipid scramblase 4
hsa-miR-126-5p	TFPI	tissue factor pathway inhibitor
hsa-miR-126-5p	NCALD	neurocalcin delta
hsa-miR-126-5p	SGCB	sarcoglycan beta
hsa-miR-126-5p	PPFIA1	PTPRF interacting protein alpha 1
hsa-miR-126-5p	ANKRD29	ankyrin repeat domain 29
hsa-miR-126-5p	MLANA	melan-A
hsa-miR-126-5p	LYPLA1	lysophospholipase 1
hsa-miR-126-5p	GJC1	gap junction protein gamma 1
hsa-miR-126-5p	HCN1	hyperpolarization activated cyclic nucleotide gated potassium channel 1
hsa-miR-126-5p	FAM155A	family with sequence similarity 155 member A
hsa-miR-126-5p	MIA2	MIA SH3 domain ER export factor 2
hsa-miR-126-5p	AZIN1	antizyme inhibitor 1
hsa-miR-126-5p	MEX3B	mex-3 RNA binding family member B
hsa-miR-126-5p	C18orf32	chromosome 18 open reading frame 32
hsa-miR-126-5p	FSHR	follicle stimulating hormone receptor
hsa-miR-126-5p	SMIM10L1	small integral membrane protein 10 like 1
hsa-miR-126-5p	ZNF74	zinc finger protein 74
hsa-miR-126-5p	PTPRC	protein tyrosine phosphatase, receptor type C
hsa-miR-126-5p	FABP4	fatty acid binding protein 4
hsa-miR-126-5p	UBTD2	ubiquitin domain containing 2
hsa-miR-126-5p	HDAC4	histone deacetylase 4
hsa-miR-126-5p	KSR1	kinase suppressor of ras 1
hsa-miR-126-5p	FAT3	FAT atypical cadherin 3
hsa-miR-126-5p	VPREB3	V-set pre-B cell surrogate light chain 3
hsa-miR-126-5p	DDX43	DEAD-box helicase 43
hsa-miR-126-5p	NR2F1	nuclear receptor subfamily 2 group F member 1
hsa-miR-126-5p	RPS6KC1	ribosomal protein S6 kinase C1
hsa-miR-126-5p	BCL2L2	BCL2 like 2
hsa-miR-126-5p	SLAIN2	SLAIN motif family member 2
hsa-miR-126-5p	DTL	denticleless E3 ubiquitin protein ligase homolog
hsa-miR-126-5p	RALGAPA1	Ral GTPase activating protein catalytic alpha subunit 1
hsa-miR-126-5p	NETO1	neuropilin and tolloid like 1
hsa-miR-126-5p	NUAK1	NUAK family kinase 1
hsa-miR-126-5p	PPP2R5E	protein phosphatase 2 regulatory subunit B'epsilon
hsa-miR-126-5p	RPL17-C18orf32	RPL17-C18orf32 readthrough
hsa-miR-126-5p	ENTPD7	ectonucleoside triphosphate diphosphohydrolase 7
hsa-miR-126-5p	RFT1	RFT1 homolog
hsa-miR-126-5p	RCOR3	REST corepressor 3
hsa-miR-126-5p	PAM	peptidylglycine alpha-amidating monooxygenase

hsa-miR-126-5p	KCNJ16	potassium voltage-gated channel subfamily J member 16
hsa-miR-126-5p	BEND3	BEN domain containing 3
hsa-miR-126-5p	ZEB1	zinc finger E-box binding homeobox 1
hsa-miR-126-5p	RHOBTB1	Rho related BTB domain containing 1
hsa-miR-126-5p	MCU	mitochondrial calcium uniporter
hsa-miR-126-5p	ZNF697	zinc finger protein 697
hsa-miR-126-5p	UBR5	ubiquitin protein ligase E3 component n-recognin 5
hsa-miR-126-5p	LINGO1	leucine rich repeat and Ig domain containing 1
hsa-miR-126-5p	KCNT2	potassium sodium-activated channel subfamily T member 2
hsa-miR-126-5p	ZNF212	zinc finger protein 212
hsa-miR-126-5p	VPS4B	vacuolar protein sorting 4 homolog B
hsa-miR-126-5p	PIP4K2A	phosphatidylinositol-5-phosphate 4-kinase type 2 alpha
hsa-miR-126-5p	EVI5	ecotropic viral integration site 5
hsa-miR-126-5p	HCN4	hyperpolarization activated cyclic nucleotide gated potassium channel 4
hsa-miR-126-5p	SDE2	SDE2 telomere maintenance homolog
hsa-miR-126-5p	ACYP2	acylphosphatase 2
hsa-miR-126-5p	CTSS	cathepsin S
hsa-miR-126-5p	KCTD4	potassium channel tetramerization domain containing 4
hsa-miR-126-5p	CFHR3	complement factor H related 3
hsa-miR-126-5p	MARCKS	myristoylated alanine rich protein kinase C substrate
hsa-miR-126-5p	TNFRSF11A	TNF receptor superfamily member 11a
hsa-miR-126-5p	SLC2A13	solute carrier family 2 member 13
hsa-miR-126-5p	OGN	osteoglycin
hsa-miR-126-5p	INHBE	inhibin subunit beta E
hsa-miR-126-5p	C14orf39	chromosome 14 open reading frame 39
hsa-miR-126-5p	NRAP	nebulin related anchoring protein
hsa-miR-126-5p	DCBLD2	discoidin, CUB and LCCL domain containing 2
hsa-miR-126-5p	NUP88	nucleoporin 88
hsa-miR-126-5p	NUP88	nucleoporin 88
hsa-miR-126-5p	FAM151B	family with sequence similarity 151 member B
hsa-miR-126-5p	MBL2	mannose binding lectin 2
hsa-miR-126-5p	RFX3	regulatory factor X3
hsa-miR-126-5p	SPRY3	sprouty RTK signaling antagonist 3
hsa-miR-126-5p	TMEFF2	transmembrane protein with EGF like and two follistatin like domains 2
hsa-miR-126-5p	SCAI	suppressor of cancer cell invasion
hsa-miR-126-5p	TOR1AIP2	torsin 1A interacting protein 2
hsa-miR-126-5p	ARHGAP9	Rho GTPase activating protein 9
hsa-miR-126-5p	FGFR2	fibroblast growth factor receptor 2
hsa-miR-126-5p	TLL1	tolloid like 1
hsa-miR-126-5p	CDKN2D	cyclin dependent kinase inhibitor 2D
hsa-miR-126-5p	RANBP3L	RAN binding protein 3 like
hsa-miR-126-5p	CAST	calpastatin
hsa-miR-126-5p	ROCK1	Rho associated coiled-coil containing protein kinase 1

hsa-miR-126-5p	PRKCH	protein kinase C eta
hsa-miR-126-5p	CDC14A	cell division cycle 14A
hsa-miR-126-5p	PDZD2	PDZ domain containing 2
hsa-miR-126-5p	BCAT1	branched chain amino acid transaminase 1
hsa-miR-126-5p	ZSCAN30	zinc finger and SCAN domain containing 30
hsa-miR-126-5p	MAPK1IP1L	mitogen-activated protein kinase 1 interacting protein 1 like
hsa-miR-126-5p	MTFR1	mitochondrial fission regulator 1
hsa-miR-126-5p	AP1AR	adaptor related protein complex 1 associated regulatory protein
hsa-miR-126-5p	OR51E2	olfactory receptor family 51 subfamily E member 2
hsa-miR-126-5p	RGS18	regulator of G protein signaling 18
hsa-miR-126-5p	ADAM22	ADAM metallopeptidase domain 22
hsa-miR-126-5p	AKNA	AT-hook transcription factor
hsa-miR-126-5p	PTGFR	prostaglandin F receptor
hsa-miR-126-5p	CCDC82	coiled-coil domain containing 82
hsa-miR-126-5p	ZFYVE16	zinc finger FYVE-type containing 16
hsa-miR-126-5p	SEC22A	SEC22 homolog A, vesicle trafficking protein
hsa-miR-126-5p	GLO1	glyoxalase I
hsa-miR-126-5p	STXBP4	syntaxin binding protein 4
hsa-miR-126-5p	ARF6	ADP ribosylation factor 6
hsa-miR-126-5p	ANAPC7	anaphase promoting complex subunit 7
hsa-miR-126-5p	ZNF793	zinc finger protein 793
hsa-miR-126-5p	CLASP1	cytoplasmic linker associated protein 1
hsa-miR-126-5p	PTPRD	protein tyrosine phosphatase, receptor type D
hsa-miR-126-5p	RBBP4	RB binding protein 4, chromatin remodeling factor
hsa-miR-126-5p	SRCAP	Snf2 related CREBBP activator protein
hsa-miR-126-5p	KCNH2	potassium voltage-gated channel subfamily H member 2
hsa-miR-126-5p	REL	REL proto-oncogene, NF-kB subunit
hsa-miR-126-5p	CPNE3	copine 3
hsa-miR-126-5p	MYT1	myelin transcription factor 1
hsa-miR-126-5p	DVL3	dishevelled segment polarity protein 3
hsa-miR-126-5p	COL4A1	collagen type IV alpha 1 chain
hsa-miR-126-5p	ST8SIA1	ST8 alpha-N-acetyl-neuraminide alpha-2,8-sialyltransferase 1
hsa-miR-126-5p	RNMT	RNA guanine-7 methyltransferase
hsa-miR-126-5p	CA5B	carbonic anhydrase 5B
hsa-miR-126-5p	DSC2	desmocollin 2
hsa-miR-126-5p	RALGPS2	Ral GEF with PH domain and SH3 binding motif 2
hsa-miR-126-5p	PIH1D2	PIH1 domain containing 2
hsa-miR-126-5p	RAPGEF6	Rap guanine nucleotide exchange factor 6
hsa-miR-126-5p	CCKBR	cholecystokinin B receptor
hsa-miR-126-5p	DDX31	DEAD-box helicase 31
hsa-miR-126-5p	TCAIM	T cell activation inhibitor, mitochondrial
hsa-miR-126-5p	MICU3	mitochondrial calcium uptake family member 3
hsa-miR-126-5p	ST6GALNAC1	ST6 N-acetylgalactosaminide alpha-2,6-sialyltransferase 1
hsa-miR-126-5p	SERF1B	small EDRK-rich factor 1B
hsa-miR-126-5p	UPP2	uridine phosphorylase 2

hsa-miR-126-5p	LHFPL3	LHFPL tetraspan subfamily member 3
hsa-miR-126-5p	NAALAD2	N-acetylated alpha-linked acidic dipeptidase 2
hsa-miR-126-5p	OLFM3	olfactomedin 3
hsa-miR-126-5p	ZNF254	zinc finger protein 254
hsa-miR-126-5p	TCFL5	transcription factor like 5
hsa-miR-126-5p	A1CF	APOBEC1 complementation factor
hsa-miR-126-5p	RIMS1	regulating synaptic membrane exocytosis 1
hsa-miR-126-5p	THSD7A	thrombospondin type 1 domain containing 7A
hsa-miR-126-5p	ZNF546	zinc finger protein 546
hsa-miR-126-5p	DPP8	dipeptidyl peptidase 8
hsa-miR-126-5p	MAP9	microtubule associated protein 9
hsa-miR-126-5p	C5orf30	chromosome 5 open reading frame 30
hsa-miR-126-5p	USP15	ubiquitin specific peptidase 15
hsa-miR-126-5p	XPA	XPA, DNA damage recognition and repair factor
hsa-miR-126-5p	PKIA	cAMP-dependent protein kinase inhibitor alpha
hsa-miR-126-5p	EIF2A	eukaryotic translation initiation factor 2A
hsa-miR-126-5p	JAKMIP3	Janus kinase and microtubule interacting protein 3
hsa-miR-126-5p	FAF1	Fas associated factor 1
hsa-miR-126-5p	QKI	QKI, KH domain containing RNA binding
hsa-miR-126-5p	CPEB2	cytoplasmic polyadenylation element binding protein 2
hsa-miR-126-5p	PLOD2	procollagen-lysine,2-oxoglutarate 5-dioxygenase 2
hsa-miR-126-5p	H3F3A	H3 histone family member 3A
hsa-miR-126-5p	BMPR1B	bone morphogenetic protein receptor type 1B
hsa-miR-126-5p	ILDR2	immunoglobulin like domain containing receptor 2
hsa-miR-126-5p	GPR155	G protein-coupled receptor 155
hsa-miR-126-5p	COPA	coatomer protein complex subunit alpha
hsa-miR-126-5p	FLT4	fms related tyrosine kinase 4
hsa-miR-126-5p	LIN54	lin-54 DREAM MuvB core complex component
hsa-miR-126-5p	FOXN3	forkhead box N3
hsa-miR-126-5p	FOLH1B	folate hydrolase 1B
hsa-miR-126-5p	ZSCAN32	zinc finger and SCAN domain containing 32
hsa-miR-126-5p	ADCY7	adenylate cyclase 7
hsa-miR-126-5p	DDHD1	DDHD domain containing 1
hsa-miR-126-5p	JMY	junction mediating and regulatory protein, p53 cofactor
hsa-miR-126-5p	CACNA1E	calcium voltage-gated channel subunit alpha1 E
hsa-miR-126-5p	CFAP299	cilia and flagella associated protein 299
hsa-miR-126-5p	HIF1A	hypoxia inducible factor 1 subunit alpha
hsa-miR-126-5p	CLCC1	chloride channel CLIC like 1
hsa-miR-126-5p	POLR2F	RNA polymerase II subunit F
hsa-miR-126-5p	GPA33	glycoprotein A33
hsa-miR-126-5p	MED13L	mediator complex subunit 13 like
hsa-miR-126-5p	DOK6	docking protein 6
hsa-miR-126-5p	OSBPL8	oxysterol binding protein like 8
hsa-miR-126-5p	HECA	hdc homolog, cell cycle regulator
hsa-miR-126-5p	TTC33	tetratricopeptide repeat domain 33

hsa-miR-126-5p	AEBP2	AE binding protein 2
hsa-miR-126-5p	ZBTB2	zinc finger and BTB domain containing 2
hsa-miR-126-5p	PLXNA4	plexin A4
hsa-miR-126-5p	DDX21	DExD-box helicase 21
hsa-miR-126-5p	M1AP	meiosis 1 associated protein
hsa-miR-126-5p	SPAG16	sperm associated antigen 16
hsa-miR-126-5p	KIF5B	kinesin family member 5B
hsa-miR-126-5p	DCUN1D5	defective in cullin neddylation 1 domain containing 5
hsa-miR-126-5p	NRXN1	neurexin 1
hsa-miR-126-5p	NAF1	nuclear assembly factor 1 ribonucleoprotein
hsa-miR-126-5p	CD44	CD44 molecule (Indian blood group)
hsa-miR-126-5p	SERF1A	small EDRK-rich factor 1A
hsa-miR-126-5p	CPLX3	complexin 3
hsa-miR-126-5p	HDAC9	histone deacetylase 9
hsa-miR-126-5p	LRP1B	LDL receptor related protein 1B
hsa-miR-126-5p	EPHA7	EPH receptor A7
hsa-miR-126-5p	ME2	malic enzyme 2
hsa-miR-126-5p	CLIC2	chloride intracellular channel 2
hsa-miR-126-5p	NAB1	NGFI-A binding protein 1
hsa-miR-126-5p	TOB2	transducer of ERBB2, 2
hsa-miR-126-5p	GABRA6	gamma-aminobutyric acid type A receptor alpha6 subunit
hsa-miR-126-5p	PTER	phosphotriesterase related
hsa-miR-126-5p	PNPLA8	patatin like phospholipase domain containing 8
hsa-miR-126-5p	SKAP2	src kinase associated phosphoprotein 2
hsa-miR-126-5p	C8orf88	chromosome 8 open reading frame 88
hsa-miR-126-5p	ANAPC16	anaphase promoting complex subunit 16
hsa-miR-126-5p	TMPRSS11D	transmembrane serine protease 11D
hsa-miR-126-5p	LRRC19	leucine rich repeat containing 19
hsa-miR-126-5p	PLEKHF1	pleckstrin homology and FYVE domain containing 1
hsa-miR-126-5p	SLC17A9	solute carrier family 17 member 9
hsa-miR-126-5p	CRCP	CGRP receptor component
hsa-miR-126-5p	PTH2R	parathyroid hormone 2 receptor
hsa-miR-126-5p	BCO1	beta-carotene oxygenase 1
hsa-miR-128-3p	AFF4	AF4/FMR2 family member 4
hsa-miR-128-3p	SZRD1	SUZ RNA binding domain containing 1
hsa-miR-128-3p	KDM7A	lysine demethylase 7A
hsa-miR-128-3p	SEC22A	SEC22 homolog A, vesicle trafficking protein
hsa-miR-128-3p	GPAM	glycerol-3-phosphate acyltransferase, mitochondrial
hsa-miR-128-3p	FRMPD3	FERM and PDZ domain containing 3
hsa-miR-128-3p	RPS6KA5	ribosomal protein S6 kinase A5
hsa-miR-128-3p	PAIP2	poly(A) binding protein interacting protein 2
hsa-miR-128-3p	PHF6	PHD finger protein 6
hsa-miR-128-3p	DCUN1D4	defective in cullin neddylation 1 domain containing 4
hsa-miR-128-3p	SMAP1	small ArfGAP 1
hsa-miR-128-3p	IGLON5	IgLON family member 5

hsa-miR-128-3p	UBE2E2	ubiquitin conjugating enzyme E2 E2
hsa-miR-128-3p	KCNK2	potassium two pore domain channel subfamily K member 2
hsa-miR-128-3p	POGLUT1	protein O-glucosyltransferase 1
hsa-miR-128-3p	ARRDC4	arrestin domain containing 4
hsa-miR-128-3p	MOSMO	modulator of smoothened
hsa-miR-128-3p	KCNK10	potassium two pore domain channel subfamily K member 10
hsa-miR-128-3p	CDS1	CDP-diacylglycerol synthase 1
hsa-miR-128-3p	PLK2	polo like kinase 2
hsa-miR-128-3p	TNPO1	transportin 1
hsa-miR-128-3p	TRPV3	transient receptor potential cation channel subfamily V member 3
hsa-miR-128-3p	TMTC2	transmembrane and tetratricopeptide repeat containing 2
hsa-miR-128-3p	GRIA3	glutamate ionotropic receptor AMPA type subunit 3
hsa-miR-128-3p	SRGAP2	SLIT-ROBO Rho GTPase activating protein 2
hsa-miR-128-3p	OTULIN	OTU deubiquitinase with linear linkage specificity
hsa-miR-128-3p	MSI2	musashi RNA binding protein 2
hsa-miR-128-3p	DTX4	deltex E3 ubiquitin ligase 4
hsa-miR-128-3p	CSF1	colony stimulating factor 1
hsa-miR-128-3p	BEND4	BEN domain containing 4
hsa-miR-128-3p	NGFR	nerve growth factor receptor
hsa-miR-128-3p	SS18	SS18, nBAF chromatin remodeling complex subunit
hsa-miR-128-3p	POGZ	pogo transposable element derived with ZNF domain
hsa-miR-128-3p	SOCS5	suppressor of cytokine signaling 5
hsa-miR-128-3p	ECE2	endothelin converting enzyme 2
hsa-miR-128-3p	CACNG2	calcium voltage-gated channel auxiliary subunit gamma 2
hsa-miR-128-3p	RGL2	ral guanine nucleotide dissociation stimulator like 2
hsa-miR-128-3p	UBR5	ubiquitin protein ligase E3 component n-recognin 5
hsa-miR-128-3p	PRKX	protein kinase X-linked
hsa-miR-128-3p	MPP6	membrane palmitoylated protein 6
hsa-miR-128-3p	FAM184A	family with sequence similarity 184 member A
hsa-miR-128-3p	PCNX1	pecanex 1
hsa-miR-128-3p	EEF1AKMT4-ECE2	EEF1AKMT4-ECE2 readthrough
hsa-miR-128-3p	SLC22A23	solute carrier family 22 member 23
hsa-miR-128-3p	NRK	Nik related kinase
hsa-miR-128-3p	GAB1	GRB2 associated binding protein 1
hsa-miR-128-3p	TUB	tubby bipartite transcription factor
hsa-miR-128-3p	TEAD1	TEA domain transcription factor 1
hsa-miR-128-3p	DOT1L	DOT1 like histone lysine methyltransferase
hsa-miR-128-3p	NFIL3	nuclear factor, interleukin 3 regulated
hsa-miR-128-3p	PDIA5	protein disulfide isomerase family A member 5
hsa-miR-128-3p	SLC6A1	solute carrier family 6 member 1
hsa-miR-128-3p	GCC1	GRIP and coiled-coil domain containing 1
hsa-miR-128-3p	AKIRIN1	akirin 1
hsa-miR-128-3p	UNC13C	unc-13 homolog C
hsa-miR-128-3p	CCDC88A	coiled-coil domain containing 88A

hsa-miR-128-3p	RO60	Ro60, Y RNA binding protein
hsa-miR-128-3p	MED13L	mediator complex subunit 13 like
hsa-miR-128-3p	RETREG3	reticulophagy regulator family member 3
hsa-miR-128-3p	SOS1	SOS Ras/Rac guanine nucleotide exchange factor 1
hsa-miR-128-3p	FAM126A	family with sequence similarity 126 member A
hsa-miR-128-3p	IRS1	insulin receptor substrate 1
hsa-miR-128-3p	TTC39A	tetratricopeptide repeat domain 39A
hsa-miR-128-3p	CEP76	centrosomal protein 76
hsa-miR-128-3p	PHB	prohibitin
hsa-miR-128-3p	RNF182	ring finger protein 182
hsa-miR-128-3p	GATA6	GATA binding protein 6
hsa-miR-128-3p	AK2	adenylate kinase 2
hsa-miR-128-3p	PTPRB	protein tyrosine phosphatase, receptor type B
hsa-miR-128-3p	AK4	adenylate kinase 4
hsa-miR-128-3p	GXYLT1	glucoside xylosyltransferase 1
hsa-miR-128-3p	UGT8	UDP glycosyltransferase 8
hsa-miR-128-3p	NEO1	neogenin 1
hsa-miR-128-3p	PDE3A	phosphodiesterase 3A
hsa-miR-128-3p	GRIN2D	glutamate ionotropic receptor NMDA type subunit 2D
hsa-miR-128-3p	ADCY2	adenylate cyclase 2
hsa-miR-128-3p	H3F3B	H3 histone family member 3B
hsa-miR-128-3p	ZNF704	zinc finger protein 704
hsa-miR-128-3p	TGFBR1	transforming growth factor beta receptor 1
hsa-miR-128-3p	XPR1	xenotropic and polytropic retrovirus receptor 1
hsa-miR-128-3p	CNOT6	CCR4-NOT transcription complex subunit 6
hsa-miR-128-3p	NF1	neurofibromin 1
hsa-miR-128-3p	VEGFC	vascular endothelial growth factor C
hsa-miR-128-3p	CXADR	CXADR, Ig-like cell adhesion molecule
hsa-miR-128-3p	TRIL	TLR4 interactor with leucine rich repeats
hsa-miR-128-3p	ARMC8	armadillo repeat containing 8
hsa-miR-128-3p	CABLES2	Cdk5 and Abl enzyme substrate 2
hsa-miR-128-3p	SETD7	SET domain containing 7, histone lysine methyltransferase
hsa-miR-128-3p	SPTY2D1	SPT2 chromatin protein domain containing 1
hsa-miR-128-3p	TTC9	tetratricopeptide repeat domain 9
hsa-miR-128-3p	RCOR3	REST corepressor 3
hsa-miR-128-3p	PTPN9	protein tyrosine phosphatase, non-receptor type 9
hsa-miR-128-3p	USP42	ubiquitin specific peptidase 42
hsa-miR-128-3p	EIF2S2	eukaryotic translation initiation factor 2 subunit beta
hsa-miR-128-3p	ABL2	ABL proto-oncogene 2, non-receptor tyrosine kinase
hsa-miR-128-3p	GALNT7	polypeptide N-acetylgalactosaminyltransferase 7
hsa-miR-128-3p	ROR1	receptor tyrosine kinase like orphan receptor 1
hsa-miR-128-3p	MIEF1	mitochondrial elongation factor 1
hsa-miR-128-3p	USH2A	usherin
hsa-miR-128-3p	YWHAB	tyrosine 3-monooxygenase/tryptophan 5-monooxygenase activation protein beta

hsa-miR-128-3p	ERC2	ELKS/RAB6-interacting/CAST family member 2
hsa-miR-128-3p	ARHGEF38	Rho guanine nucleotide exchange factor 38
hsa-miR-128-3p	PDHX	pyruvate dehydrogenase complex component X
hsa-miR-128-3p	NEK2	NIMA related kinase 2
hsa-miR-128-3p	ATP8A1	ATPase phospholipid transporting 8A1
hsa-miR-128-3p	MAPK14	mitogen-activated protein kinase 14
hsa-miR-128-3p	NXT2	nuclear transport factor 2 like export factor 2
hsa-miR-128-3p	NABP1	nucleic acid binding protein 1
hsa-miR-128-3p	VANGL2	VANGL planar cell polarity protein 2
hsa-miR-128-3p	STOX2	storkhead box 2
hsa-miR-128-3p	GRIK3	glutamate ionotropic receptor kainate type subunit 3
hsa-miR-128-3p	RNGTT	RNA guanylyltransferase and 5'-phosphatase
hsa-miR-128-3p	NFX1	nuclear transcription factor, X-box binding 1
hsa-miR-128-3p	RYBP	RING1 and YY1 binding protein
hsa-miR-128-3p	COMMD3-BMI1	COMMD3-BMI1 readthrough
hsa-miR-128-3p	FBLN5	fibulin 5
hsa-miR-128-3p	SLC39A7	solute carrier family 39 member 7
hsa-miR-128-3p	FOXA3	forkhead box A3
hsa-miR-128-3p	BAG2	BCL2 associated athanogene 2
hsa-miR-128-3p	FRYL	FRY like transcription coactivator
hsa-miR-128-3p	STK32A	serine/threonine kinase 32A
hsa-miR-128-3p	PPM1E	protein phosphatase, Mg2+/Mn2+ dependent 1E
hsa-miR-128-3p	STRN4	striatin 4
hsa-miR-128-3p	PLCH1	phospholipase C eta 1
hsa-miR-128-3p	ARHGAP12	Rho GTPase activating protein 12
hsa-miR-128-3p	HIC1	HIC ZBTB transcriptional repressor 1
hsa-miR-128-3p	SNAP25	synaptosome associated protein 25
hsa-miR-128-3p	CYP39A1	cytochrome P450 family 39 subfamily A member 1
hsa-miR-128-3p	EPHB2	EPH receptor B2
hsa-miR-128-3p	COL27A1	collagen type XXVII alpha 1 chain
hsa-miR-128-3p	KDM3A	lysine demethylase 3A
hsa-miR-128-3p	AMER2	APC membrane recruitment protein 2
hsa-miR-128-3p	PHF24	PHD finger protein 24
hsa-miR-128-3p	FAM126B	family with sequence similarity 126 member B
hsa-miR-128-3p	MDN1	midasin AAA ATPase 1
hsa-miR-128-3p	RAB20	RAB20, member RAS oncogene family
hsa-miR-128-3p	EML1	EMAP like 1
hsa-miR-128-3p	ISL1	ISL LIM homeobox 1
hsa-miR-128-3p	RNF38	ring finger protein 38
hsa-miR-128-3p	USP49	ubiquitin specific peptidase 49
hsa-miR-128-3p	TMEM64	transmembrane protein 64
hsa-miR-128-3p	PPFIA2	PTPRF interacting protein alpha 2
hsa-miR-128-3p	SCAI	suppressor of cancer cell invasion
hsa-miR-128-3p	SFXN2	sideroflexin 2
hsa-miR-128-3p	SORL1	sortilin related receptor 1

hsa-miR-128-3p	TMEM167A	transmembrane protein 167A
hsa-miR-128-3p	ZNF652	zinc finger protein 652
hsa-miR-128-3p	UGCG	UDP-glucose ceramide glucosyltransferase
hsa-miR-128-3p	MET	MET proto-oncogene, receptor tyrosine kinase
hsa-miR-128-3p	ARHGAP32	Rho GTPase activating protein 32
hsa-miR-128-3p	RET	ret proto-oncogene
hsa-miR-128-3p	CASC3	CASC3, exon junction complex subunit
hsa-miR-128-3p	SLC7A11	solute carrier family 7 member 11
hsa-miR-128-3p	BAZ2B	bromodomain adjacent to zinc finger domain 2B
hsa-miR-128-3p	PLAGL2	PLAG1 like zinc finger 2
hsa-miR-128-3p	RECK	reversion inducing cysteine rich protein with kazal motifs
hsa-miR-128-3p	NAA50	N(alpha)-acetyltransferase 50, NatE catalytic subunit
hsa-miR-128-3p	MED12L	mediator complex subunit 12 like
hsa-miR-128-3p	SH2D3C	SH2 domain containing 3C
hsa-miR-128-3p	FAM155A	family with sequence similarity 155 member A
hsa-miR-128-3p	GRIA4	glutamate ionotropic receptor AMPA type subunit 4
hsa-miR-128-3p	C20orf194	chromosome 20 open reading frame 194
hsa-miR-128-3p	WNK1	WNK lysine deficient protein kinase 1
hsa-miR-128-3p	PLCL2	phospholipase C like 2
hsa-miR-128-3p	MAPK8IP3	mitogen-activated protein kinase 8 interacting protein 3
hsa-miR-128-3p	CECR2	CECR2, histone acetyl-lysine reader
hsa-miR-128-3p	PDPK1	3-phosphoinositide dependent protein kinase 1
hsa-miR-128-3p	ZFP36L1	ZFP36 ring finger protein like 1
hsa-miR-128-3p	IRF4	interferon regulatory factor 4
hsa-miR-128-3p	LTBP1	latent transforming growth factor beta binding protein 1
hsa-miR-128-3p	CLPP	caseinolytic mitochondrial matrix peptidase proteolytic subunit
hsa-miR-128-3p	ZFHX3	zinc finger homeobox 3
hsa-miR-128-3p	VPS4B	vacuolar protein sorting 4 homolog B
hsa-miR-128-3p	SNX12	sorting nexin 12
hsa-miR-128-3p	GREM1	gremlin 1, DAN family BMP antagonist
hsa-miR-128-3p	NRBF2	nuclear receptor binding factor 2
hsa-miR-128-3p	NUS1	NUS1, dehydrodolichyl diphosphate synthase subunit
hsa-miR-128-3p	PDE10A	phosphodiesterase 10A
hsa-miR-128-3p	RASGEF1B	RasGEF domain family member 1B
hsa-miR-128-3p	RHOT2	ras homolog family member T2
hsa-miR-128-3p	CDIP1	cell death inducing p53 target 1
hsa-miR-128-3p	SH3BGRL2	SH3 domain binding glutamate rich protein like 2
hsa-miR-128-3p	FEM1B	fem-1 homolog B
hsa-miR-128-3p	UNC80	unc-80 homolog, NALCN channel complex subunit
hsa-miR-128-3p	RELN	reelin
hsa-miR-128-3p	ABCA12	ATP binding cassette subfamily A member 12
hsa-miR-128-3p	IGSF3	immunoglobulin superfamily member 3
hsa-miR-128-3p	DIRAS1	DIRAS family GTPase 1
hsa-miR-128-3p	CDH24	cadherin 24
hsa-miR-128-3p	ZNF800	zinc finger protein 800

hsa-miR-128-3p	PDE7B	phosphodiesterase 7B
hsa-miR-128-3p	PTPRT	protein tyrosine phosphatase, receptor type T
hsa-miR-128-3p	SP1	Sp1 transcription factor
hsa-miR-128-3p	RNF144A	ring finger protein 144A
hsa-miR-128-3p	SAMD10	sterile alpha motif domain containing 10
hsa-miR-128-3p	MAPKAPK3	mitogen-activated protein kinase-activated protein kinase 3
hsa-miR-128-3p	ARHGAP21	Rho GTPase activating protein 21
hsa-miR-128-3p	SMARCA2	SWI/SNF related, matrix associated, actin dependent regulator of chromatin, subfamily a, member 2
hsa-miR-128-3p	FAM155B	family with sequence similarity 155 member B
hsa-miR-128-3p	SEC24A	SEC24 homolog A, COPII coat complex component
hsa-miR-128-3p	CREB1	cAMP responsive element binding protein 1
hsa-miR-128-3p	NSD1	nuclear receptor binding SET domain protein 1
hsa-miR-128-3p	RSBN1L	round spermatid basic protein 1 like
hsa-miR-128-3p	CCNC	cyclin C
hsa-miR-128-3p	IL13RA1	interleukin 13 receptor subunit alpha 1
hsa-miR-128-3p	ABCB9	ATP binding cassette subfamily B member 9
hsa-miR-128-3p	PGAP1	post-GPI attachment to proteins 1
hsa-miR-128-3p	KBTBD11	kelch repeat and BTB domain containing 11
hsa-miR-128-3p	UBR1	ubiquitin protein ligase E3 component n-recognin 1
hsa-miR-128-3p	LITAF	lipopolysaccharide induced TNF factor
hsa-miR-128-3p	H3F3C	H3 histone family member 3C
hsa-miR-128-3p	UBE2W	ubiquitin conjugating enzyme E2 W
hsa-miR-128-3p	PIH1D3	PIH1 domain containing 3
hsa-miR-128-3p	COL21A1	collagen type XXI alpha 1 chain
hsa-miR-128-3p	MEGF11	multiple EGF like domains 11
hsa-miR-128-3p	C20orf202	chromosome 20 open reading frame 202
hsa-miR-128-3p	NREP	neuronal regeneration related protein
hsa-miR-128-3p	MSL1	MSL complex subunit 1
hsa-miR-128-3p	CA10	carbonic anhydrase 10
hsa-miR-128-3p	DCC	DCC netrin 1 receptor
hsa-miR-128-3p	PPP4C	protein phosphatase 4 catalytic subunit
hsa-miR-128-3p	MTMR4	myotubularin related protein 4
hsa-miR-128-3p	UBE2N	ubiquitin conjugating enzyme E2 N
hsa-miR-128-3p	GLTP	glycolipid transfer protein
hsa-miR-128-3p	PPME1	protein phosphatase methylesterase 1
hsa-miR-128-3p	SYT1	synaptotagmin 1
hsa-miR-128-3p	TMEM30A	transmembrane protein 30A
hsa-miR-128-3p	WDR7	WD repeat domain 7
hsa-miR-128-3p	STK24	serine/threonine kinase 24
hsa-miR-128-3p	ATXN10	ataxin 10
hsa-miR-128-3p	GCC2	GRIP and coiled-coil domain containing 2
hsa-miR-128-3p	FBXW7	F-box and WD repeat domain containing 7
hsa-miR-128-3p	CCNT2	cyclin T2
hsa-miR-128-3p	DCP1A	decapping mRNA 1A

hsa-miR-128-3p	FAM177A1	family with sequence similarity 177 member A1
hsa-miR-128-3p	PNKD	PNKD, MBL domain containing
hsa-miR-128-3p	AMD1	adenosylmethionine decarboxylase 1
hsa-miR-128-3p	CTDSP2	CTD small phosphatase 2
hsa-miR-128-3p	MBTD1	mbt domain containing 1
hsa-miR-128-3p	OSBPL10	oxysterol binding protein like 10
hsa-miR-128-3p	BCORL1	BCL6 corepressor like 1
hsa-miR-128-3p	VANGL1	VANGL planar cell polarity protein 1
hsa-miR-128-3p	TXNIP	thioredoxin interacting protein
hsa-miR-128-3p	SREK1	splicing regulatory glutamic acid and lysine rich protein 1
hsa-miR-128-3p	FNDC4	fibronectin type III domain containing 4
hsa-miR-128-3p	SOX7	SRY-box 7
hsa-miR-128-3p	C1QTNF7	C1q and TNF related 7
hsa-miR-128-3p	KCNN3	potassium calcium-activated channel subfamily N member 3
hsa-miR-128-3p	RPS6KB1	ribosomal protein S6 kinase B1
hsa-miR-128-3p	TRIM23	tripartite motif containing 23
hsa-miR-128-3p	FBLN2	fibulin 2
hsa-miR-128-3p	DISC1	DISC1 scaffold protein
hsa-miR-128-3p	SEC61A1	Sec61 translocon alpha 1 subunit
hsa-miR-128-3p	SGMS1	sphingomyelin synthase 1
hsa-miR-128-3p	MDH1B	malate dehydrogenase 1B
hsa-miR-128-3p	SLC35F3	solute carrier family 35 member F3
hsa-miR-128-3p	ANKRD40	ankyrin repeat domain 40
hsa-miR-128-3p	ABHD17C	abhydrolase domain containing 17C
hsa-miR-128-3p	SIRT1	sirtuin 1
hsa-miR-128-3p	GSPT1	G1 to S phase transition 1
hsa-miR-128-3p	NAV2	neuron navigator 2
hsa-miR-128-3p	NEMP2	nuclear envelope integral membrane protein 2
hsa-miR-128-3p	CKAP4	cytoskeleton associated protein 4
hsa-miR-128-3p	ITGA5	integrin subunit alpha 5
hsa-miR-128-3p	AKR7A2	aldo-keto reductase family 7 member A2
hsa-miR-128-3p	ADAMTS5	ADAM metallopeptidase with thrombospondin type 1 motif 5
hsa-miR-128-3p	GALNT3	polypeptide N-acetylgalactosaminyltransferase 3
hsa-miR-128-3p	PROSER2	proline and serine rich 2
hsa-miR-128-3p	SP2	Sp2 transcription factor
hsa-miR-128-3p	GRM5	glutamate metabotropic receptor 5
hsa-miR-128-3p	SPG21	SPG21, maspardin
hsa-miR-128-3p	ZNF618	zinc finger protein 618
hsa-miR-128-3p	HECTD1	HECT domain E3 ubiquitin protein ligase 1
hsa-miR-128-3p	SSH1	slingshot protein phosphatase 1
hsa-miR-128-3p	RAP1B	RAP1B, member of RAS oncogene family
hsa-miR-128-3p	SAMD12	sterile alpha motif domain containing 12
hsa-miR-128-3p	UBA6	ubiquitin like modifier activating enzyme 6
hsa-miR-128-3p	HOXA5	homeobox A5
hsa-miR-128-3p	JAG1	jagged 1

hsa-miR-128-3p	HAPLN1	hyaluronan and proteoglycan link protein 1
hsa-miR-128-3p	RND3	Rho family GTPase 3
hsa-miR-128-3p	EFR3A	EFR3 homolog A
hsa-miR-128-3p	MATN3	matrilin 3
hsa-miR-128-3p	N4BP1	NEDD4 binding protein 1
hsa-miR-128-3p	MLLT10	MLLT10, histone lysine methyltransferase DOT1L cofactor
hsa-miR-128-3p	SH3RF1	SH3 domain containing ring finger 1
hsa-miR-128-3p	ZKSCAN2	zinc finger with KRAB and SCAN domains 2
hsa-miR-128-3p	CEMIP	cell migration inducing hyaluronidase 1
hsa-miR-128-3p	RERE	arginine-glutamic acid dipeptide repeats
hsa-miR-128-3p	ERLEC1	endoplasmic reticulum lectin 1
hsa-miR-128-3p	GABBR2	gamma-aminobutyric acid type B receptor subunit 2
hsa-miR-128-3p	ZFP82	ZFP82 zinc finger protein
hsa-miR-128-3p	AFF3	AF4/FMR2 family member 3
hsa-miR-128-3p	GSK3B	glycogen synthase kinase 3 beta
hsa-miR-128-3p	MYT1	myelin transcription factor 1
hsa-miR-128-3p	BMI1	BMI1 proto-oncogene, polycomb ring finger
hsa-miR-128-3p	LYPD3	LY6/PLAUR domain containing 3
hsa-miR-128-3p	TMC7	transmembrane channel like 7
hsa-miR-128-3p	TMEM25	transmembrane protein 25
hsa-miR-128-3p	INO80D	INO80 complex subunit D
hsa-miR-128-3p	CACNA2D3	calcium voltage-gated channel auxiliary subunit alpha2delta 3
hsa-miR-128-3p	ITPKC	inositol-trisphosphate 3-kinase C
hsa-miR-128-3p	MMD	monocyte to macrophage differentiation associated
hsa-miR-128-3p	ST6GALNAC3	ST6 N-acetylgalactosaminide alpha-2,6-sialyltransferase 3
hsa-miR-128-3p	MAP2K7	mitogen-activated protein kinase kinase 7
hsa-miR-128-3p	SLC35F1	solute carrier family 35 member F1
hsa-miR-128-3p	WEE1	WEE1 G2 checkpoint kinase
hsa-miR-128-3p	LBH	limb bud and heart development
hsa-miR-128-3p	PAX9	paired box 9
hsa-miR-128-3p	FBXO30	F-box protein 30
hsa-miR-128-3p	PDS5B	PDS5 cohesin associated factor B
hsa-miR-128-3p	NEUROD6	neuronal differentiation 6
hsa-miR-128-3p	APOLD1	apolipoprotein L domain containing 1
hsa-miR-128-3p	EPB41L1	erythrocyte membrane protein band 4.1 like 1
hsa-miR-128-3p	COPB2	coatomer protein complex subunit beta 2
hsa-miR-128-3p	STX7	syntaxin 7
hsa-miR-128-3p	MINPP1	multiple inositol-polyphosphate phosphatase 1
hsa-miR-128-3p	PLPPR1	phospholipid phosphatase related 1
hsa-miR-128-3p	MNT	MAX network transcriptional repressor
hsa-miR-128-3p	ALDH4A1	aldehyde dehydrogenase 4 family member A1
hsa-miR-128-3p	CA7	carbonic anhydrase 7
hsa-miR-128-3p	FAM78A	family with sequence similarity 78 member A
hsa-miR-128-3p	HCN4	hyperpolarization activated cyclic nucleotide gated potassium channel 4

hsa-miR-128-3p	DCX	doublecortin
hsa-miR-128-3p	EYA4	EYA transcriptional coactivator and phosphatase 4
hsa-miR-128-3p	GNS	glucosamine (N-acetyl)-6-sulfatase
hsa-miR-128-3p	NPTXR	neuronal pentraxin receptor
hsa-miR-128-3p	HMBOX1	homeobox containing 1
hsa-miR-128-3p	IFITM10	interferon induced transmembrane protein 10
hsa-miR-128-3p	GPATCH2L	G-patch domain containing 2 like
hsa-miR-128-3p	MOSPD3	motile sperm domain containing 3
hsa-miR-128-3p	LHFPL3	LHFPL tetraspan subfamily member 3
hsa-miR-128-3p	ING5	inhibitor of growth family member 5
hsa-miR-128-3p	RETREG1	reticulophagy regulator 1
hsa-miR-128-3p	MTSS1L	MTSS1L, I-BAR domain containing
hsa-miR-128-3p	C1orf21	chromosome 1 open reading frame 21
hsa-miR-128-3p	ST14	suppression of tumorigenicity 14
hsa-miR-128-3p	ARF3	ADP ribosylation factor 3
hsa-miR-128-3p	SOCS6	suppressor of cytokine signaling 6
hsa-miR-128-3p	OPA1	OPA1, mitochondrial dynamin like GTPase
hsa-miR-128-3p	ZC3H12D	zinc finger CCCH-type containing 12D
hsa-miR-128-3p	DPY19L3	dpy-19 like C-mannosyltransferase 3
hsa-miR-128-3p	CHTF8	chromosome transmission fidelity factor 8
hsa-miR-128-3p	CCDC92	coiled-coil domain containing 92
hsa-miR-128-3p	MIPOL1	mirror-image polydactyly 1
hsa-miR-128-3p	COLGALT2	collagen beta(1-O)galactosyltransferase 2
hsa-miR-128-3p	CRKL	CRK like proto-oncogene, adaptor protein
hsa-miR-128-3p	MED14	mediator complex subunit 14
hsa-miR-128-3p	WBP1L	WW domain binding protein 1 like
hsa-miR-128-3p	GIGYF2	GRB10 interacting GYF protein 2
hsa-miR-128-3p	SOGA1	suppressor of glucose, autophagy associated 1
hsa-miR-128-3p	KIRREL1	kirre like nephrin family adhesion molecule 1
hsa-miR-128-3p	KLHDC8A	kelch domain containing 8A
hsa-miR-128-3p	KAT7	lysine acetyltransferase 7
hsa-miR-128-3p	ARID1B	AT-rich interaction domain 1B
hsa-miR-128-3p	CLDN18	claudin 18
hsa-miR-128-3p	TCEA1	transcription elongation factor A1
hsa-miR-128-3p	ZBTB20	zinc finger and BTB domain containing 20
hsa-miR-128-3p	SLC39A11	solute carrier family 39 member 11
hsa-miR-128-3p	STIM2	stromal interaction molecule 2
hsa-miR-128-3p	SCAF11	SR-related CTD associated factor 11
hsa-miR-128-3p	LPAR6	lysophosphatidic acid receptor 6
hsa-miR-128-3p	SMAD9	SMAD family member 9
hsa-miR-128-3p	NDST1	N-deacetylase and N-sulfotransferase 1
hsa-miR-128-3p	DHTKD1	dehydrogenase E1 and transketolase domain containing 1
hsa-miR-128-3p	B3GNT7	UDP-GlcNAc:betaGal beta-1,3-N-acetylglucosaminyltransferase 7
hsa-miR-128-3p	SLIT2	slit guidance ligand 2
hsa-miR-128-3p	TMEM87A	transmembrane protein 87A

hsa-miR-128-3p	REPS1	RALBP1 associated Eps domain containing 1
hsa-miR-128-3p	F3	coagulation factor III, tissue factor
hsa-miR-128-3p	EPB41L4A	erythrocyte membrane protein band 4.1 like 4A
hsa-miR-128-3p	HIP1	huntingtin interacting protein 1
hsa-miR-128-3p	USP46	ubiquitin specific peptidase 46
hsa-miR-128-3p	SHTN1	shootin 1
hsa-miR-128-3p	GTF2A2	general transcription factor IIA subunit 2
hsa-miR-128-3p	ASPH	aspartate beta-hydroxylase
hsa-miR-128-3p	GPD2	glycerol-3-phosphate dehydrogenase 2
hsa-miR-128-3p	MARK4	microtubule affinity regulating kinase 4
hsa-miR-128-3p	SASH1	SAM and SH3 domain containing 1
hsa-miR-128-3p	SLC26A11	solute carrier family 26 member 11
hsa-miR-128-3p	ITCH	itchy E3 ubiquitin protein ligase
hsa-miR-128-3p	KIAA1109	KIAA1109
hsa-miR-128-3p	SLC5A3	solute carrier family 5 member 3
hsa-miR-128-3p	NKX3-1	NK3 homeobox 1
hsa-miR-128-3p	DPY19L4	dpy-19 like 4
hsa-miR-128-3p	SNX18	sorting nexin 18
hsa-miR-128-3p	DPP10	dipeptidyl peptidase like 10
hsa-miR-128-3p	RCAN2	regulator of calcineurin 2
hsa-miR-128-3p	G6PC3	glucose-6-phosphatase catalytic subunit 3
hsa-miR-128-3p	TPPP	tubulin polymerization promoting protein
hsa-miR-128-3p	LIMK1	LIM domain kinase 1
hsa-miR-128-3p	CBLB	Cbl proto-oncogene B
hsa-miR-128-3p	ENAH	ENAH, actin regulator
hsa-miR-128-3p	KMT2A	lysine methyltransferase 2A
hsa-miR-128-3p	SKA3	spindle and kinetochore associated complex subunit 3
hsa-miR-128-3p	COL5A1	collagen type V alpha 1 chain
hsa-miR-128-3p	ONECUT2	one cut homeobox 2
hsa-miR-128-3p	MME	membrane metalloendopeptidase
hsa-miR-128-3p	FXR2	FMR1 autosomal homolog 2
hsa-miR-128-3p	SLC24A4	solute carrier family 24 member 4
hsa-miR-128-3p	SPATA17	spermatogenesis associated 17
hsa-miR-128-3p	FSD2	fibronectin type III and SPRY domain containing 2
hsa-miR-128-3p	CSRP2	cysteine and glycine rich protein 2
hsa-miR-128-3p	APBA2	amyloid beta precursor protein binding family A member 2
hsa-miR-128-3p	CNTLN	centlein
hsa-miR-128-3p	ELMO1	engulfment and cell motility 1
hsa-miR-128-3p	NRXN1	neurexin 1
hsa-miR-128-3p	MCF2L	MCF.2 cell line derived transforming sequence like
hsa-miR-128-3p	RAB11FIP1	RAB11 family interacting protein 1
hsa-miR-128-3p	UTP15	UTP15, small subunit processome component
hsa-miR-128-3p	GAPT	GRB2 binding adaptor protein, transmembrane
hsa-miR-128-3p	RAB39B	RAB39B, member RAS oncogene family
hsa-miR-128-3p	STK35	serine/threonine kinase 35

hsa-miR-128-3p	ECE1	endothelin converting enzyme 1
hsa-miR-128-3p	MSTO1	misato mitochondrial distribution and morphology regulator 1
hsa-miR-128-3p	TNFRSF10D	TNF receptor superfamily member 10d
hsa-miR-128-3p	BICC1	BicC family RNA binding protein 1
hsa-miR-128-3p	NEURL4	neuralized E3 ubiquitin protein ligase 4
hsa-miR-128-3p	RARA	retinoic acid receptor alpha
hsa-miR-128-3p	WNT3A	Wnt family member 3A
hsa-miR-128-3p	MBOAT2	membrane bound O-acyltransferase domain containing 2
hsa-miR-128-3p	PPP1CC	protein phosphatase 1 catalytic subunit gamma
hsa-miR-128-3p	NRP2	neuropilin 2
hsa-miR-128-3p	CA12	carbonic anhydrase 12
hsa-miR-128-3p	TTC39B	tetratricopeptide repeat domain 39B
hsa-miR-128-3p	SERTAD2	SERTA domain containing 2
hsa-miR-128-3p	MIER2	MIER family member 2
hsa-miR-128-3p	SAMD9L	sterile alpha motif domain containing 9 like
hsa-miR-128-3p	CCNK	cyclin K
hsa-miR-128-3p	E2F7	E2F transcription factor 7
hsa-miR-128-3p	MTDH	metadherin
hsa-miR-128-3p	OPHN1	oligophrenin 1
hsa-miR-128-3p	GLRA2	glycine receptor alpha 2
hsa-miR-128-3p	STAG1	stromal antigen 1
hsa-miR-128-3p	ZDHHC17	zinc finger DHHC-type containing 17
hsa-miR-128-3p	SLC39A13	solute carrier family 39 member 13
hsa-miR-128-3p	UBN2	ubinuclein 2
hsa-miR-128-3p	PITPNM2	phosphatidylinositol transfer protein membrane associated 2
hsa-miR-128-3p	GEM	GTP binding protein overexpressed in skeletal muscle
hsa-miR-128-3p	SMAD2	SMAD family member 2
hsa-miR-128-3p	TSR1	TSR1, ribosome maturation factor
hsa-miR-128-3p	PANK1	pantothenate kinase 1
hsa-miR-128-3p	ZNF705E	zinc finger protein 705E
hsa-miR-128-3p	KCNA1	potassium voltage-gated channel subfamily A member 1
hsa-miR-128-3p	TET1	tet methylcytosine dioxygenase 1
hsa-miR-128-3p	UBE2V1	ubiquitin conjugating enzyme E2 V1
hsa-miR-128-3p	YAF2	YY1 associated factor 2
hsa-miR-128-3p	DYNLL2	dynein light chain LC8-type 2
hsa-miR-128-3p	PLD6	phospholipase D family member 6
hsa-miR-128-3p	DBF4B	DBF4 zinc finger B
hsa-miR-128-3p	AARD	alanine and arginine rich domain containing protein
hsa-miR-128-3p	NCOA7	nuclear receptor coactivator 7
hsa-miR-128-3p	FAM102A	family with sequence similarity 102 member A
hsa-miR-128-3p	RIC3	RIC3 acetylcholine receptor chaperone
hsa-miR-128-3p	MTA3	metastasis associated 1 family member 3
hsa-miR-128-3p	PTPRQ	protein tyrosine phosphatase, receptor type Q
hsa-miR-128-3p	PTAR1	protein prenyltransferase alpha subunit repeat containing 1
hsa-miR-128-3p	GCNT2	glucosaminyl (N-acetyl) transferase 2 (I blood group)

hsa-miR-128-3p	DZIP1	DAZ interacting zinc finger protein 1
hsa-miR-128-3p	ZNF84	zinc finger protein 84
hsa-miR-128-3p	DENND1B	DENN domain containing 1B
hsa-miR-128-3p	ATP6V1C1	ATPase H+ transporting V1 subunit C1
hsa-miR-128-3p	KCTD4	potassium channel tetramerization domain containing 4
hsa-miR-128-3p	CCN4	cellular communication network factor 4
hsa-miR-128-3p	SBF2	SET binding factor 2
hsa-miR-128-3p	SETD5	SET domain containing 5
hsa-miR-128-3p	TMEM132E	transmembrane protein 132E
hsa-miR-128-3p	FBRSL1	fibrosin like 1
hsa-miR-128-3p	CLCN3	chloride voltage-gated channel 3
hsa-miR-128-3p	SLITRK1	SLIT and NTRK like family member 1
hsa-miR-128-3p	ZNF737	zinc finger protein 737
hsa-miR-128-3p	CIPC	CLOCK interacting pacemaker
hsa-miR-128-3p	SRP72	signal recognition particle 72
hsa-miR-128-3p	TBC1D22B	TBC1 domain family member 22B
hsa-miR-128-3p	YPEL3	yippee like 3
hsa-miR-128-3p	MED13	mediator complex subunit 13
hsa-miR-128-3p	C17orf102	chromosome 17 open reading frame 102
hsa-miR-128-3p	IFFO2	intermediate filament family orphan 2
hsa-miR-128-3p	ADGRG6	adhesion G protein-coupled receptor G6
hsa-miR-128-3p	PITHD1	PITH domain containing 1
hsa-miR-128-3p	SLC6A17	solute carrier family 6 member 17
hsa-miR-128-3p	RGS6	regulator of G protein signaling 6
hsa-miR-128-3p	PCM1	pericentriolar material 1
hsa-miR-128-3p	WNK3	WNK lysine deficient protein kinase 3
hsa-miR-128-3p	PFKL	phosphofructokinase, liver type
hsa-miR-128-3p	MDFI	MyoD family inhibitor
hsa-miR-128-3p	RBPMS2	RNA binding protein, mRNA processing factor 2
hsa-miR-128-3p	EBI3	Epstein-Barr virus induced 3
hsa-miR-128-3p	NXF1	nuclear RNA export factor 1
hsa-miR-128-3p	WASHC4	WASH complex subunit 4
hsa-miR-128-3p	SEMA6A	semaphorin 6A
hsa-miR-128-3p	PHF20	PHD finger protein 20
hsa-miR-128-3p	ZHX1	zinc fingers and homeoboxes 1
hsa-miR-128-3p	PLXNC1	plexin C1
hsa-miR-128-3p	ABHD17B	abhydrolase domain containing 17B
hsa-miR-128-3p	VAV3	vav guanine nucleotide exchange factor 3
hsa-miR-128-3p	EDRF1	erythroid differentiation regulatory factor 1
hsa-miR-128-3p	TCEANC2	transcription elongation factor A N-terminal and central domain containing 2
hsa-miR-128-3p	ZCCHC24	zinc finger CCHC-type containing 24
hsa-miR-128-3p	STARD4	StAR related lipid transfer domain containing 4
hsa-miR-128-3p	EYA1	EYA transcriptional coactivator and phosphatase 1
hsa-miR-128-3p	PDE12	phosphodiesterase 12

hsa-miR-128-3p	NR2F6	nuclear receptor subfamily 2 group F member 6
hsa-miR-128-3p	SEMA6D	semaphorin 6D
hsa-miR-128-3p	ARHGAP28	Rho GTPase activating protein 28
hsa-miR-128-3p	MPL	MPL proto-oncogene, thrombopoietin receptor
hsa-miR-128-3p	CDCP1	CUB domain containing protein 1
hsa-miR-128-3p	PIK3R1	phosphoinositide-3-kinase regulatory subunit 1
hsa-miR-128-3p	TMEM266	transmembrane protein 266
hsa-miR-128-3p	FOXO1	forkhead box O1
hsa-miR-128-3p	LRAT	lecithin retinol acyltransferase
hsa-miR-128-3p	OGFRL1	opioid growth factor receptor like 1
hsa-miR-128-3p	LMTK2	lemur tyrosine kinase 2
hsa-miR-128-3p	FAM172A	family with sequence similarity 172 member A
hsa-miR-128-3p	NCBP3	nuclear cap binding subunit 3
hsa-miR-128-3p	ANK1	ankyrin 1
hsa-miR-128-3p	CPEB3	cytoplasmic polyadenylation element binding protein 3
hsa-miR-128-3p	TMBIM6	transmembrane BAX inhibitor motif containing 6
hsa-miR-128-3p	RASL12	RAS like family 12
hsa-miR-128-3p	CYLD	CYLD lysine 63 deubiquitinase
hsa-miR-128-3p	MOB1B	MOB kinase activator 1B
hsa-miR-128-3p	SYNDIG1	synapse differentiation inducing 1
hsa-miR-128-3p	RGS8	regulator of G protein signaling 8
hsa-miR-128-3p	AMMECR1L	AMMECR1 like
hsa-miR-128-3p	ATL3	atlastin GTPase 3
hsa-miR-128-3p	CCR9	C-C motif chemokine receptor 9
hsa-miR-128-3p	EHF	ETS homologous factor
hsa-miR-128-3p	LSM1	LSM1 homolog, mRNA degradation associated
hsa-miR-128-3p	NPTX1	neuronal pentraxin 1
hsa-miR-128-3p	YPEL2	yippee like 2
hsa-miR-128-3p	IBSP	integrin binding sialoprotein
hsa-miR-128-3p	NTRK3	neurotrophic receptor tyrosine kinase 3
hsa-miR-128-3p	TNRC18	trinucleotide repeat containing 18
hsa-miR-128-3p	CAPZA1	capping actin protein of muscle Z-line subunit alpha 1
hsa-miR-128-3p	CBFB	core-binding factor subunit beta
hsa-miR-128-3p	DLGAP3	DLG associated protein 3
hsa-miR-128-3p	AGO3	argonaute RISC catalytic component 3
hsa-miR-128-3p	UBE2E3	ubiquitin conjugating enzyme E2 E3
hsa-miR-128-3p	PLEKHJ1	pleckstrin homology domain containing J1
hsa-miR-128-3p	DUSP18	dual specificity phosphatase 18
hsa-miR-128-3p	CCT3	chaperonin containing TCP1 subunit 3
hsa-miR-128-3p	PFKFB4	6-phosphofructo-2-kinase/fructose-2,6-biphosphatase 4
hsa-miR-128-3p	SMURF2	SMAD specific E3 ubiquitin protein ligase 2
hsa-miR-128-3p	DCAF7	DDB1 and CUL4 associated factor 7
hsa-miR-128-3p	SPTBN1	spectrin beta, non-erythrocytic 1
hsa-miR-128-3p	ACVR2A	activin A receptor type 2A
hsa-miR-128-3p	LIX1	limb and CNS expressed 1

hsa-miR-128-3p	GNG12	G protein subunit gamma 12
hsa-miR-128-3p	LY6G5B	lymphocyte antigen 6 family member G5B
hsa-miR-128-3p	IKZF2	IKAROS family zinc finger 2
hsa-miR-128-3p	SLU7	SLU7 homolog, splicing factor
hsa-miR-128-3p	CNR1	cannabinoid receptor 1
hsa-miR-128-3p	CPD	carboxypeptidase D
hsa-miR-128-3p	PDGFRA	platelet derived growth factor receptor alpha
hsa-miR-128-3p	TAB3	TGF-beta activated kinase 1 (MAP3K7) binding protein 3
hsa-miR-128-3p	TRAF3	TNF receptor associated factor 3
hsa-miR-128-3p	TFEB	transcription factor EB
hsa-miR-128-3p	HEG1	heart development protein with EGF like domains 1
hsa-miR-128-3p	ADCY3	adenylate cyclase 3
hsa-miR-128-3p	GATAD2A	GATA zinc finger domain containing 2A
hsa-miR-128-3p	RBM4	RNA binding motif protein 4
hsa-miR-128-3p	ELOVL6	ELOVL fatty acid elongase 6
hsa-miR-128-3p	FUBP3	far upstream element binding protein 3
hsa-miR-128-3p	MIER3	MIER family member 3
hsa-miR-128-3p	PCTP	phosphatidylcholine transfer protein
hsa-miR-128-3p	MAGI3	membrane associated guanylate kinase, WW and PDZ domain containing 3
hsa-miR-128-3p	PTPN5	protein tyrosine phosphatase, non-receptor type 5
hsa-miR-128-3p	GOT2	glutamic-oxaloacetic transaminase 2
hsa-miR-128-3p	HIPK2	homeodomain interacting protein kinase 2
hsa-miR-128-3p	UNC45B	unc-45 myosin chaperone B
hsa-miR-128-3p	TMEM91	transmembrane protein 91
hsa-miR-128-3p	INSR	insulin receptor
hsa-miR-128-3p	SFRP1	secreted frizzled related protein 1
hsa-miR-128-3p	ZNF510	zinc finger protein 510
hsa-miR-128-3p	HOXA9	homeobox A9
hsa-miR-128-3p	DCP2	decapping mRNA 2
hsa-miR-128-3p	SCAMP3	secretory carrier membrane protein 3
hsa-miR-128-3p	C11orf87	chromosome 11 open reading frame 87
hsa-miR-128-3p	PLPP3	phospholipid phosphatase 3
hsa-miR-128-3p	IPMK	inositol polyphosphate multikinase
hsa-miR-128-3p	TM9SF2	transmembrane 9 superfamily member 2
hsa-miR-128-3p	LGALS3	galectin 3
hsa-miR-128-3p	BAHD1	bromo adjacent homology domain containing 1
hsa-miR-128-3p	RIPOR2	RHO family interacting cell polarization regulator 2
hsa-miR-128-3p	NPAS3	neuronal PAS domain protein 3
hsa-miR-128-3p	HOXA10	homeobox A10
hsa-miR-128-3p	TCAIM	T cell activation inhibitor, mitochondrial
hsa-miR-128-3p	PTPRJ	protein tyrosine phosphatase, receptor type J
hsa-miR-128-3p	BEX3	brain expressed X-linked 3
hsa-miR-128-3p	KMT2C	lysine methyltransferase 2C
hsa-miR-128-3p	TLK2	tousled like kinase 2

hsa-miR-128-3p	RESF1	retroelement silencing factor 1
hsa-miR-128-3p	SEMA7A	semaphorin 7A (John Milton Hagen blood group)
hsa-miR-128-3p	H2AFY	H2A histone family member Y
hsa-miR-128-3p	UNC5D	unc-5 netrin receptor D
hsa-miR-128-3p	ZBTB39	zinc finger and BTB domain containing 39
hsa-miR-128-3p	GRIP1	glutamate receptor interacting protein 1
hsa-miR-128-3p	ZNF696	zinc finger protein 696
hsa-miR-128-3p	NUDT6	nudix hydrolase 6
hsa-miR-128-3p	GFPT2	glutamine-fructose-6-phosphate transaminase 2
hsa-miR-128-3p	CCDC50	coiled-coil domain containing 50
hsa-miR-128-3p	PPIL4	peptidylprolyl isomerase like 4
hsa-miR-128-3p	PTPN4	protein tyrosine phosphatase, non-receptor type 4
hsa-miR-128-3p	COL3A1	collagen type III alpha 1 chain
hsa-miR-128-3p	DCK	deoxycytidine kinase
hsa-miR-128-3p	DDX6	DEAD-box helicase 6
hsa-miR-128-3p	BMPR2	bone morphogenetic protein receptor type 2
hsa-miR-128-3p	CCDC71	coiled-coil domain containing 71
hsa-miR-128-3p	UNKL	unk like zinc finger
hsa-miR-128-3p	CASC10	cancer susceptibility 10
hsa-miR-128-3p	FBXO33	F-box protein 33
hsa-miR-128-3p	PRKAG2	protein kinase AMP-activated non-catalytic subunit gamma 2
hsa-miR-128-3p	COX6C	cytochrome c oxidase subunit 6C
hsa-miR-128-3p	ULK1	unc-51 like autophagy activating kinase 1
hsa-miR-128-3p	ABCB10	ATP binding cassette subfamily B member 10
hsa-miR-128-3p	TRIM32	tripartite motif containing 32
hsa-miR-128-3p	TPH2	tryptophan hydroxylase 2
hsa-miR-128-3p	MAGEA10	MAGE family member A10
hsa-miR-128-3p	USP15	ubiquitin specific peptidase 15
hsa-miR-128-3p	CDKN2A	cyclin dependent kinase inhibitor 2A
hsa-miR-128-3p	PTGER3	prostaglandin E receptor 3
hsa-miR-128-3p	MEGF6	multiple EGF like domains 6
hsa-miR-128-3p	ADGRA3	adhesion G protein-coupled receptor A3
hsa-miR-128-3p	ACTA2	actin, alpha 2, smooth muscle, aorta
hsa-miR-128-3p	SLC25A25	solute carrier family 25 member 25
hsa-miR-128-3p	ID2	inhibitor of DNA binding 2
hsa-miR-128-3p	DEF8	differentially expressed in FDCP 8 homolog
hsa-miR-128-3p	FGD6	FYVE, RhoGEF and PH domain containing 6
hsa-miR-128-3p	LRRN4CL	LRRN4 C-terminal like
hsa-miR-128-3p	ACTR8	ARP8 actin related protein 8 homolog
hsa-miR-128-3p	ARHGAP19	Rho GTPase activating protein 19
hsa-miR-128-3p	RAB3B	RAB3B, member RAS oncogene family
hsa-miR-128-3p	NAA15	N(alpha)-acetyltransferase 15, NatA auxiliary subunit
hsa-miR-128-3p	RAP2A	RAP2A, member of RAS oncogene family
hsa-miR-128-3p	STK40	serine/threonine kinase 40
hsa-miR-128-3p	TMCC1	transmembrane and coiled-coil domain family 1

hsa-miR-128-3p	RALGAPA2	Ral GTPase activating protein catalytic alpha subunit 2
hsa-miR-128-3p	ST13	ST13, Hsp70 interacting protein
hsa-miR-128-3p	ZBTB34	zinc finger and BTB domain containing 34
hsa-miR-128-3p	NHS	NHS actin remodeling regulator
hsa-miR-128-3p	NETO2	neuropilin and tolloid like 2
hsa-miR-128-3p	TRMT2A	tRNA methyltransferase 2 homolog A
hsa-miR-128-3p	PELI2	pellino E3 ubiquitin protein ligase family member 2
hsa-miR-128-3p	UBE2K	ubiquitin conjugating enzyme E2 K
hsa-miR-128-3p	CDYL2	chromodomain Y like 2
hsa-miR-128-3p	VSIG10	V-set and immunoglobulin domain containing 10
hsa-miR-128-3p	FAM122B	family with sequence similarity 122B
hsa-miR-128-3p	PARK7	Parkinsonism associated deglycase
hsa-miR-128-3p	KPNA3	karyopherin subunit alpha 3
hsa-miR-128-3p	CPA4	carboxypeptidase A4
hsa-miR-128-3p	SBNO1	strawberry notch homolog 1
hsa-miR-128-3p	SDF2	stromal cell derived factor 2
hsa-miR-128-3p	NOVA1	NOVA alternative splicing regulator 1
hsa-miR-128-3p	NIPAL4	NIPA like domain containing 4
hsa-miR-128-3p	CAB39	calcium binding protein 39
hsa-miR-128-3p	CHKA	choline kinase alpha
hsa-miR-128-3p	PLAGL1	PLAG1 like zinc finger 1
hsa-miR-128-3p	PSPC1	paraspeckle component 1
hsa-miR-128-3p	PROZ	protein Z, vitamin K dependent plasma glycoprotein
hsa-miR-128-3p	GOLGA6C	golgin A6 family member C
hsa-miR-128-3p	RUNDC3A	RUN domain containing 3A
hsa-miR-128-3p	MDM4	MDM4, p53 regulator
hsa-miR-128-3p	NRARP	NOTCH regulated ankyrin repeat protein
hsa-miR-128-3p	ORMDL3	ORMDL sphingolipid biosynthesis regulator 3
hsa-miR-128-3p	TM4SF20	transmembrane 4 L six family member 20
hsa-miR-128-3p	ANKS1A	ankyrin repeat and sterile alpha motif domain containing 1A
hsa-miR-128-3p	TMX1	thioredoxin related transmembrane protein 1
hsa-miR-128-3p	TYW1	tRNA-yW synthesizing protein 1 homolog
hsa-miR-128-3p	USP25	ubiquitin specific peptidase 25
hsa-miR-128-3p	TMEM170A	transmembrane protein 170A
hsa-miR-128-3p	IKZF1	IKAROS family zinc finger 1
hsa-miR-128-3p	DOCK11	dedicator of cytokinesis 11
hsa-miR-128-3p	DNAJC13	DnaJ heat shock protein family (Hsp40) member C13
hsa-miR-128-3p	LPGAT1	lysophosphatidylglycerol acyltransferase 1
hsa-miR-128-3p	ZEB1	zinc finger E-box binding homeobox 1
hsa-miR-128-3p	ZNF569	zinc finger protein 569
hsa-miR-128-3p	NKX3-2	NK3 homeobox 2
hsa-miR-128-3p	DVL2	dishevelled segment polarity protein 2
hsa-miR-128-3p	NDUFS4	NADH:ubiquinone oxidoreductase subunit S4
hsa-miR-128-3p	KCNA4	potassium voltage-gated channel subfamily A member 4
hsa-miR-128-3p	CBFA2T3	CBFA2/RUNX1 translocation partner 3

hsa-miR-128-3p	NTNG1	netrin G1
hsa-miR-128-3p	TTC28	tetratricopeptide repeat domain 28
hsa-miR-128-3p	GATA2	GATA binding protein 2
hsa-miR-128-3p	BRSK2	BR serine/threonine kinase 2
hsa-miR-128-3p	DMTF1	cyclin D binding myb like transcription factor 1
hsa-miR-128-3p	FN1	fibronectin 1
hsa-miR-128-3p	CLP1	cleavage and polyadenylation factor I subunit 1
hsa-miR-128-3p	KMT5A	lysine methyltransferase 5A
hsa-miR-128-3p	EIF5	eukaryotic translation initiation factor 5
hsa-miR-128-3p	INPP5J	inositol polyphosphate-5-phosphatase J
hsa-miR-128-3p	PIAS2	protein inhibitor of activated STAT 2
hsa-miR-128-3p	INPP1	inositol polyphosphate-1-phosphatase
hsa-miR-128-3p	POU2F1	POU class 2 homeobox 1
hsa-miR-128-3p	NEK6	NIMA related kinase 6
hsa-miR-128-3p	TBC1D9B	TBC1 domain family member 9B
hsa-miR-128-3p	JCHAIN	joining chain of multimeric IgA and IgM
hsa-miR-128-3p	ARFGEF1	ADP ribosylation factor guanine nucleotide exchange factor 1
hsa-miR-128-3p	FAM102B	family with sequence similarity 102 member B
hsa-miR-128-3p	CDH6	cadherin 6
hsa-miR-128-3p	SUZ12	SUZ12, polycomb repressive complex 2 subunit
hsa-miR-128-3p	KCNJ3	potassium voltage-gated channel subfamily J member 3
hsa-miR-128-3p	SHISA6	shisa family member 6
hsa-miR-128-3p	ALG9	ALG9, alpha-1,2-mannosyltransferase
hsa-miR-128-3p	TENT4A	terminal nucleotidyltransferase 4A
hsa-miR-128-3p	PRDM16	PR/SET domain 16
hsa-miR-128-3p	ETV3L	ETS variant 3 like
hsa-miR-128-3p	HAND2	heart and neural crest derivatives expressed 2
hsa-miR-128-3p	LRRFIP1	LRR binding FLII interacting protein 1
hsa-miR-128-3p	UNC5C	unc-5 netrin receptor C
hsa-miR-128-3p	DMKN	dermokine
hsa-miR-128-3p	ADH7	alcohol dehydrogenase 7 (class IV), mu or sigma polypeptide
hsa-miR-128-3p	SPATA2	spermatogenesis associated 2
hsa-miR-128-3p	ATOH8	atonal bHLH transcription factor 8
hsa-miR-128-3p	ARL8B	ADP ribosylation factor like GTPase 8B
hsa-miR-128-3p	SHE	Src homology 2 domain containing E
hsa-miR-128-3p	YTHDC1	YTH domain containing 1
hsa-miR-128-3p	OGA	O-GlcNAcase
hsa-miR-128-3p	ORC5	origin recognition complex subunit 5
hsa-miR-128-3p	ELFN2	extracellular leucine rich repeat and fibronectin type III domain containing 2
hsa-miR-128-3p	PKIA	cAMP-dependent protein kinase inhibitor alpha
hsa-miR-128-3p	LIN7C	lin-7 homolog C, crumbs cell polarity complex component
hsa-miR-128-3p	KIAA1549L	KIAA1549 like
hsa-miR-128-3p	ILDR2	immunoglobulin like domain containing receptor 2
hsa-miR-128-3p	SESN2	sestrin 2

hsa-miR-128-3p	TDRP	testis development related protein
hsa-miR-128-3p	NECTIN3	nectin cell adhesion molecule 3
hsa-miR-128-3p	S100A7A	S100 calcium binding protein A7A
hsa-miR-128-3p	TCIM	transcriptional and immune response regulator
hsa-miR-128-3p	DCAF17	DDB1 and CUL4 associated factor 17
hsa-miR-128-3p	UHRF2	ubiquitin like with PHD and ring finger domains 2
hsa-miR-128-3p	FOXN3	forkhead box N3
hsa-miR-128-3p	TAF4	TATA-box binding protein associated factor 4
hsa-miR-128-3p	SSX2B	SSX family member 2B
hsa-miR-128-3p	NEMP1	nuclear envelope integral membrane protein 1
hsa-miR-128-3p	DDHD1	DDHD domain containing 1
hsa-miR-128-3p	PDE3B	phosphodiesterase 3B
hsa-miR-128-3p	RMND5A	required for meiotic nuclear division 5 homolog A
hsa-miR-128-3p	CRB2	crumbs cell polarity complex component 2
hsa-miR-128-3p	TMEM229A	transmembrane protein 229A
hsa-miR-128-3p	TRIM6-TRIM34	TRIM6-TRIM34 readthrough
hsa-miR-128-3p	PNISR	PNN interacting serine and arginine rich protein
hsa-miR-128-3p	MAST4	microtubule associated serine/threonine kinase family member 4
hsa-miR-128-3p	TRIM34	tripartite motif containing 34
hsa-miR-128-3p	SREBF2	sterol regulatory element binding transcription factor 2
hsa-miR-128-3p	NLRP2B	NLR family pyrin domain containing 2B
hsa-miR-128-3p	RXRA	retinoid X receptor alpha
hsa-miR-128-3p	LIN28B	lin-28 homolog B
hsa-miR-128-3p	TPM1	tropomyosin 1
hsa-miR-128-3p	ERG	ETS transcription factor ERG
hsa-miR-128-3p	CPLX3	complexin 3
hsa-miR-128-3p	DKK2	dickkopf WNT signaling pathway inhibitor 2
hsa-miR-128-3p	SSX2	SSX family member 2
hsa-miR-128-3p	FAM206A	family with sequence similarity 206 member A
hsa-miR-128-3p	ZNF792	zinc finger protein 792
hsa-miR-128-3p	PLAG1	PLAG1 zinc finger
hsa-miR-128-3p	C1orf52	chromosome 1 open reading frame 52
hsa-miR-128-3p	TBC1D1	TBC1 domain family member 1
hsa-miR-128-3p	KIAA1211L	KIAA1211 like
hsa-miR-128-3p	ADAMTS10	ADAM metallopeptidase with thrombospondin type 1 motif 10
hsa-miR-128-3p	TMUB1	transmembrane and ubiquitin like domain containing 1
hsa-miR-128-3p	ARHGEF26	Rho guanine nucleotide exchange factor 26
hsa-miR-128-3p	HBEGF	heparin binding EGF like growth factor
hsa-miR-128-3p	RASAL2	RAS protein activator like 2
hsa-miR-128-3p	IL12RB2	interleukin 12 receptor subunit beta 2
hsa-miR-128-3p	CRTC1	CREB regulated transcription coactivator 1
hsa-miR-128-3p	RFX3	regulatory factor X3
hsa-miR-128-3p	ELAVL2	ELAV like RNA binding protein 2
hsa-miR-128-3p	AIF1L	allograft inflammatory factor 1 like
hsa-miR-128-3p	BET1L	Bet1 golgi vesicular membrane trafficking protein like

hsa-miR-128-3p	ALDH5A1	aldehyde dehydrogenase 5 family member A1
hsa-miR-128-3p	WDFY1	WD repeat and FYVE domain containing 1
hsa-miR-128-3p	SAR1A	secretion associated Ras related GTPase 1A
hsa-miR-128-3p	TRIO	trio Rho guanine nucleotide exchange factor
hsa-miR-128-3p	TBX3	T-box 3
hsa-miR-128-3p	WIPF2	WAS/WASL interacting protein family member 2
hsa-miR-128-3p	MS4A2	membrane spanning 4-domains A2
hsa-miR-128-3p	CCDC85C	coiled-coil domain containing 85C
hsa-miR-128-3p	RBM33	RNA binding motif protein 33
hsa-miR-128-3p	MTMR10	myotubularin related protein 10
hsa-miR-128-3p	POM121	POM121 transmembrane nucleoporin
hsa-miR-128-3p	PDK1	pyruvate dehydrogenase kinase 1
hsa-miR-128-3p	GALNT13	polypeptide N-acetylgalactosaminyltransferase 13
hsa-miR-128-3p	ZFP36L2	ZFP36 ring finger protein like 2
hsa-miR-128-3p	KIAA1210	KIAA1210
hsa-miR-128-3p	RREB1	ras responsive element binding protein 1
hsa-miR-128-3p	ELL2	elongation factor for RNA polymerase II 2
hsa-miR-128-3p	TMCC3	transmembrane and coiled-coil domain family 3
hsa-miR-128-3p	ZNF385A	zinc finger protein 385A
hsa-miR-128-3p	YY1AP1	YY1 associated protein 1
hsa-miR-128-3p	KCNAB2	potassium voltage-gated channel subfamily A regulatory beta subunit 2
hsa-miR-128-3p	FAM13B	family with sequence similarity 13 member B
hsa-miR-128-3p	GNG2	G protein subunit gamma 2
hsa-miR-128-3p	PBXIP1	PBX homeobox interacting protein 1
hsa-miR-128-3p	SPOUT1	SPOUT domain containing methyltransferase 1
hsa-miR-128-3p	NR5A2	nuclear receptor subfamily 5 group A member 2
hsa-miR-128-3p	TBC1D8B	TBC1 domain family member 8B
hsa-miR-128-3p	PLXND1	plexin D1
hsa-miR-128-3p	RAB3IP	RAB3A interacting protein
hsa-miR-128-3p	PPIF	peptidylprolyl isomerase F
hsa-miR-128-3p	CITED2	Cbp/p300 interacting transactivator with Glu/Asp rich carboxy-terminal domain 2
hsa-miR-128-3p	JMJD1C	jumonji domain containing 1C
hsa-miR-128-3p	E2F6	E2F transcription factor 6
hsa-miR-128-3p	PDE8B	phosphodiesterase 8B
hsa-miR-128-3p	FRS3	fibroblast growth factor receptor substrate 3
hsa-miR-128-3p	GRK6	G protein-coupled receptor kinase 6
hsa-miR-128-3p	COL11A2	collagen type XI alpha 2 chain
hsa-miR-128-3p	CLCN5	chloride voltage-gated channel 5
hsa-miR-128-3p	TMED5	transmembrane p24 trafficking protein 5
hsa-miR-128-3p	MYBL1	MYB proto-oncogene like 1
hsa-miR-128-3p	KPNB1	karyopherin subunit beta 1
hsa-miR-128-3p	FANCA	FA complementation group A
hsa-miR-128-3p	LRRC57	leucine rich repeat containing 57
hsa-miR-128-3p	DPH6	diphthamine biosynthesis 6

hsa-miR-128-3p	USP51	ubiquitin specific peptidase 51
hsa-miR-128-3p	LDLRAD3	low density lipoprotein receptor class A domain containing 3
hsa-miR-128-3p	NOD1	nucleotide binding oligomerization domain containing 1
hsa-miR-128-3p	CRB1	crumbs cell polarity complex component 1
hsa-miR-128-3p	SS18L1	SS18L1, nBAF chromatin remodeling complex subunit
hsa-miR-128-3p	GOLGA8A	golgin A8 family member A
hsa-miR-128-3p	FURIN	furin, paired basic amino acid cleaving enzyme
hsa-miR-128-3p	ZNF140	zinc finger protein 140
hsa-miR-128-3p	ZNF329	zinc finger protein 329
hsa-miR-128-3p	STK39	serine/threonine kinase 39
hsa-miR-128-3p	NOL4	nucleolar protein 4
hsa-miR-128-3p	ZNF577	zinc finger protein 577
hsa-miR-128-3p	ZNF426	zinc finger protein 426
hsa-miR-128-3p	POM121C	POM121 transmembrane nucleoporin C
hsa-miR-128-3p	TMEM87B	transmembrane protein 87B
hsa-miR-128-3p	STAB2	stabilin 2
hsa-miR-128-3p	MCTS1	MCTS1, re-initiation and release factor
hsa-miR-128-3p	KITLG	KIT ligand
hsa-miR-128-3p	EBF4	EBF family member 4
hsa-miR-128-3p	MVB12B	multivesicular body subunit 12B
hsa-miR-128-3p	DLL4	delta like canonical Notch ligand 4
hsa-miR-128-3p	SLC5A9	solute carrier family 5 member 9
hsa-miR-128-3p	EPB41	erythrocyte membrane protein band 4.1
hsa-miR-128-3p	FAM222B	family with sequence similarity 222 member B
hsa-miR-128-3p	ADCY6	adenylate cyclase 6
hsa-miR-128-3p	USF3	upstream transcription factor family member 3
hsa-miR-128-3p	CDK18	cyclin dependent kinase 18
hsa-miR-128-3p	CREBRF	CREB3 regulatory factor
hsa-miR-128-3p	BLOC1S5	biogenesis of lysosomal organelles complex 1 subunit 5
hsa-miR-128-3p	TIAL1	TIA1 cytotoxic granule associated RNA binding protein like 1
hsa-miR-128-3p	FOXO4	forkhead box O4
hsa-miR-128-3p	IGF2BP3	insulin like growth factor 2 mRNA binding protein 3
hsa-miR-128-3p	PRKCB	protein kinase C beta
hsa-miR-128-3p	PGM2L1	phosphoglucomutase 2 like 1
hsa-miR-128-3p	TSC1	TSC complex subunit 1
hsa-miR-128-3p	ZNF286A	zinc finger protein 286A
hsa-miR-128-3p	ITSN2	intersectin 2
hsa-miR-128-3p	FOXP4	forkhead box P4
hsa-miR-128-3p	PSMA1	proteasome subunit alpha 1
hsa-miR-128-3p	GOLGA6A	golgin A6 family member A
hsa-miR-128-3p	CADM1	cell adhesion molecule 1
hsa-miR-128-3p	COX18	cytochrome c oxidase assembly factor COX18
hsa-miR-128-3p	ZNF500	zinc finger protein 500
hsa-miR-128-3p	COX8A	cytochrome c oxidase subunit 8A
hsa-miR-128-3p	GOLGA6B	golgin A6 family member B

hsa-miR-128-3p	ZNF860	zinc finger protein 860
hsa-miR-128-3p	GOLGA6D	golgin A6 family member D
hsa-miR-128-3p	BAX	BCL2 associated X, apoptosis regulator
hsa-miR-128-3p	NUP210	nucleoporin 210
hsa-miR-128-3p	PROCR	protein C receptor
hsa-miR-128-3p	CISD2	CDGSH iron sulfur domain 2
hsa-miR-128-3p	NAB1	NGFI-A binding protein 1
hsa-miR-128-3p	ANKDD1A	ankyrin repeat and death domain containing 1A
hsa-miR-128-3p	LSM12	LSM12 homolog
hsa-miR-128-3p	GPATCH11	G-patch domain containing 11
hsa-miR-128-3p	ADGRF2	adhesion G protein-coupled receptor F2
hsa-miR-128-3p	GRAMD1B	GRAM domain containing 1B
hsa-miR-128-3p	INSM1	INSM transcriptional repressor 1
hsa-miR-128-3p	SGPP1	sphingosine-1-phosphate phosphatase 1
hsa-miR-128-3p	GOLM1	golgi membrane protein 1
hsa-miR-128-3p	SHANK3	SH3 and multiple ankyrin repeat domains 3
hsa-miR-128-3p	ZBED6CL	ZBED6 C-terminal like
hsa-miR-128-3p	NCOA1	nuclear receptor coactivator 1
hsa-miR-128-3p	ARMC3	armadillo repeat containing 3
hsa-miR-128-3p	IGFBPL1	insulin like growth factor binding protein like 1
hsa-miR-128-3p	SATB2	SATB homeobox 2
hsa-miR-128-3p	PLEKHM1	pleckstrin homology and RUN domain containing M1
hsa-miR-128-3p	E2F3	E2F transcription factor 3
hsa-miR-128-3p	RAB3C	RAB3C, member RAS oncogene family
hsa-miR-128-3p	CABP1	calcium binding protein 1
hsa-miR-128-3p	HYOU1	hypoxia up-regulated 1
hsa-miR-128-3p	BMP3	bone morphogenetic protein 3
hsa-miR-128-3p	FUT9	fucosyltransferase 9
hsa-miR-128-3p	FAM120C	family with sequence similarity 120C
hsa-miR-128-3p	RUVBL2	RuvB like AAA ATPase 2
hsa-miR-128-3p	EN1	engrailed homeobox 1
hsa-miR-128-3p	DNAJC3	DnaJ heat shock protein family (Hsp40) member C3
hsa-miR-128-3p	PLPPR5	phospholipid phosphatase related 5
hsa-miR-128-3p	SLC49A4	solute carrier family 49 member 4
hsa-miR-128-3p	ZNF37A	zinc finger protein 37A
hsa-miR-128-3p	OSER1	oxidative stress responsive serine rich 1
hsa-miR-128-3p	FBLN1	fibulin 1
hsa-miR-128-3p	MAP3K19	mitogen-activated protein kinase kinase kinase 19
hsa-miR-128-3p	RNF139	ring finger protein 139
hsa-miR-128-3p	IQSEC1	IQ motif and Sec7 domain 1
hsa-miR-128-3p	MYOCD	myocardin
hsa-miR-128-3p	DAP3	death associated protein 3
hsa-miR-128-3p	PPP1R3B	protein phosphatase 1 regulatory subunit 3B
hsa-miR-128-3p	BEAN1	brain expressed associated with NEDD4 1
hsa-miR-128-3p	PDXK	pyridoxal kinase

hsa-miR-128-3p	ZNF34	zinc finger protein 34
hsa-miR-128-3p	ALDH1L2	aldehyde dehydrogenase 1 family member L2
hsa-miR-128-3p	LPCAT1	lysophosphatidylcholine acyltransferase 1
hsa-miR-128-3p	KIAA1191	KIAA1191
hsa-miR-128-3p	NKAIN1	sodium/potassium transporting ATPase interacting 1
hsa-miR-128-3p	SYT4	synaptotagmin 4
hsa-miR-128-3p	TTC3	tetratricopeptide repeat domain 3
hsa-miR-128-3p	MICAL3	microtubule associated monooxygenase, calponin and LIM domain containing 3
hsa-miR-128-3p	C14orf93	chromosome 14 open reading frame 93
hsa-miR-128-3p	GNA13	G protein subunit alpha 13
hsa-miR-128-3p	C1orf229	chromosome 1 open reading frame 229
hsa-miR-128-3p	OTX2	orthodenticle homeobox 2
hsa-miR-128-3p	SMAD5	SMAD family member 5
hsa-miR-128-3p	LAPTM4B	lysosomal protein transmembrane 4 beta
hsa-miR-128-3p	GPATCH2	G-patch domain containing 2
hsa-miR-128-3p	G6PC2	glucose-6-phosphatase catalytic subunit 2
hsa-miR-128-3p	FAM84B	family with sequence similarity 84 member B
hsa-miR-128-3p	G0S2	G0/G1 switch 2
hsa-miR-128-3p	CCNJ	cyclin J
hsa-miR-128-3p	JADE2	jade family PHD finger 2
hsa-miR-128-3p	NOL9	nucleolar protein 9
hsa-miR-128-3p	ZIC5	Zic family member 5
hsa-miR-128-3p	GATC	glutamyl-tRNA amidotransferase subunit C
hsa-miR-128-3p	NEURL1B	neuralized E3 ubiquitin protein ligase 1B
hsa-miR-128-3p	TMEM9B	TMEM9 domain family member B
hsa-miR-128-3p	CREB3L2	cAMP responsive element binding protein 3 like 2
hsa-miR-128-3p	HMGB3	high mobility group box 3
hsa-miR-128-3p	LONRF1	LON peptidase N-terminal domain and ring finger 1
hsa-miR-128-3p	GPR161	G protein-coupled receptor 161
hsa-miR-128-3p	BTBD3	BTB domain containing 3
hsa-miR-128-3p	RELT	RELT, TNF receptor
hsa-miR-128-3p	SNIP1	Smad nuclear interacting protein 1
hsa-miR-128-3p	NMB	neuromedin B
hsa-miR-128-3p	ASAH1	N-acylsphingosine amidohydrolase 1
hsa-miR-128-3p	PRICKLE2	prickle planar cell polarity protein 2
hsa-miR-128-3p	FAM133B	family with sequence similarity 133 member B
hsa-miR-128-3p	APPBP2	amyloid beta precursor protein binding protein 2
hsa-miR-128-3p	ZNF470	zinc finger protein 470
hsa-miR-128-3p	TM2D3	TM2 domain containing 3
hsa-miR-128-3p	UBE2F	ubiquitin conjugating enzyme E2 F (putative)
hsa-miR-128-3p	SLFN13	schlafen family member 13
hsa-miR-128-3p	RGPD6	RANBP2-like and GRIP domain containing 6
hsa-miR-128-3p	RNASEH2C	ribonuclease H2 subunit C
hsa-miR-128-3p	GOLGA6L4	golgin A6 family-like 4

hsa-miR-128-3p	ARID2	AT-rich interaction domain 2
hsa-miR-128-3p	FADD	Fas associated via death domain
hsa-miR-128-3p	GOLGA6L10	golgin A6 family-like 10
hsa-miR-128-3p	SUSD1	sushi domain containing 1
hsa-miR-128-3p	TRAPPC8	trafficking protein particle complex 8
hsa-miR-128-3p	FOXQ1	forkhead box Q1
hsa-miR-128-3p	ZKSCAN4	zinc finger with KRAB and SCAN domains 4
hsa-miR-128-3p	RGPD5	RANBP2-like and GRIP domain containing 5
hsa-miR-128-3p	LDLRAP1	low density lipoprotein receptor adaptor protein 1
hsa-miR-128-3p	MAP4K5	mitogen-activated protein kinase kinase kinase kinase 5
hsa-miR-128-3p	ELOVL4	ELOVL fatty acid elongase 4
hsa-miR-128-3p	SLC7A1	solute carrier family 7 member 1
hsa-miR-128-3p	FAM13A	family with sequence similarity 13 member A
hsa-miR-128-3p	RCOR1	REST corepressor 1
hsa-miR-128-3p	CDR2L	cerebellar degeneration related protein 2 like
hsa-miR-128-3p	FAAP100	FA core complex associated protein 100
hsa-miR-128-3p	PPP2R2D	protein phosphatase 2 regulatory subunit Bdelta
hsa-miR-128-3p	NME4	NME/NM23 nucleoside diphosphate kinase 4
hsa-miR-128-3p	SPOPL	speckle type BTB/POZ protein like
hsa-miR-128-3p	CAPN7	calpain 7
hsa-miR-128-3p	ID4	inhibitor of DNA binding 4, HLH protein
hsa-miR-128-3p	LRP2BP	LRP2 binding protein
hsa-miR-128-3p	SLC10A7	solute carrier family 10 member 7
hsa-miR-128-3p	CTPS2	CTP synthase 2
hsa-miR-128-3p	SLC6A6	solute carrier family 6 member 6
hsa-miR-128-3p	IKZF5	IKAROS family zinc finger 5
hsa-miR-128-3p	TMOD2	tropomodulin 2
hsa-miR-128-3p	CASP1	caspase 1
hsa-miR-128-3p	NEBL	nebulette
hsa-miR-128-3p	INAVA	innate immunity activator
hsa-miR-128-3p	EFR3B	EFR3 homolog B
hsa-miR-128-3p	VIP	vasoactive intestinal peptide
hsa-miR-128-3p	GYS1	glycogen synthase 1
hsa-miR-128-3p	PPP2R3A	protein phosphatase 2 regulatory subunit B''alpha
hsa-miR-128-3p	SLC9A4	solute carrier family 9 member A4
hsa-miR-128-3p	TRABD2B	TraB domain containing 2B
hsa-miR-128-3p	CTU1	cytosolic thiouridylase subunit 1
hsa-miR-128-3p	SMPD4	sphingomyelin phosphodiesterase 4
hsa-miR-128-3p	ZNF783	zinc finger family member 783
hsa-miR-128-3p	SNRPE	small nuclear ribonucleoprotein polypeptide E
hsa-miR-128-3p	FCHSD2	FCH and double SH3 domains 2
hsa-miR-128-3p	KCNMB4	potassium calcium-activated channel subfamily M regulatory beta subunit 4
hsa-miR-128-3p	OR11A1	olfactory receptor family 11 subfamily A member 1
hsa-miR-128-3p	CFC1	cripto, FRL-1, cryptic family 1

hsa-miR-128-3p	APOF	apolipoprotein F
hsa-miR-128-3p	AGAP1	ArfGAP with GTPase domain, ankyrin repeat and PH domain 1
hsa-miR-128-3p	ARL15	ADP ribosylation factor like GTPase 15
hsa-miR-128-3p	MBNL2	muscleblind like splicing regulator 2
hsa-miR-128-3p	FAM71F2	family with sequence similarity 71 member F2
hsa-miR-128-3p	BTG2	BTG anti-proliferation factor 2
hsa-miR-128-3p	KCNK6	potassium two pore domain channel subfamily K member 6
hsa-miR-128-3p	EDNRA	endothelin receptor type A
hsa-miR-128-3p	CCNG1	cyclin G1
hsa-miR-128-3p	TWISTNB	TWIST neighbor
hsa-miR-128-3p	SIX1	SIX homeobox 1
hsa-miR-128-3p	LINGO4	leucine rich repeat and Ig domain containing 4
hsa-miR-128-3p	CCDC28B	coiled-coil domain containing 28B
hsa-miR-128-3p	FAM222A	family with sequence similarity 222 member A
hsa-miR-128-3p	BRAP	BRCA1 associated protein
hsa-miR-128-3p	DTNA	dystrobrevin alpha
hsa-miR-128-3p	SLC39A10	solute carrier family 39 member 10
hsa-miR-128-3p	HOXA13	homeobox A13
hsa-miR-128-3p	SH3GL3	SH3 domain containing GRB2 like 3, endophilin A3
hsa-miR-128-3p	ZBTB14	zinc finger and BTB domain containing 14
hsa-miR-128-3p	SLC9A7	solute carrier family 9 member A7
hsa-miR-128-3p	MECP2	methyl-CpG binding protein 2
hsa-miR-128-3p	DUSP5	dual specificity phosphatase 5
hsa-miR-128-3p	CREG2	cellular repressor of E1A stimulated genes 2
hsa-miR-128-3p	CSDC2	cold shock domain containing C2
hsa-miR-128-3p	NTRK2	neurotrophic receptor tyrosine kinase 2
hsa-miR-128-3p	TRAF1	TNF receptor associated factor 1
hsa-miR-128-3p	PRLR	prolactin receptor
hsa-miR-128-3p	RIMS3	regulating synaptic membrane exocytosis 3
hsa-miR-128-3p	SDK2	sidekick cell adhesion molecule 2
hsa-miR-128-3p	ACOT13	acyl-CoA thioesterase 13
hsa-miR-128-3p	WTAP	WT1 associated protein
hsa-miR-128-3p	WDTC1	WD and tetratricopeptide repeats 1
hsa-miR-128-3p	APCDD1L	APC down-regulated 1 like
hsa-miR-128-3p	BHLHB9	basic helix-loop-helix family member b9
hsa-miR-128-3p	CDH11	cadherin 11
hsa-miR-128-3p	C17orf51	chromosome 17 open reading frame 51
hsa-miR-128-3p	PWWP3A	PWWP domain containing 3A, DNA repair factor
hsa-miR-128-3p	CCM2	CCM2 scaffold protein
hsa-miR-128-3p	DHCR24	24-dehydrocholesterol reductase
hsa-miR-128-3p	PIGZ	phosphatidylinositol glycan anchor biosynthesis class Z
hsa-miR-128-3p	MBD3	methyl-CpG binding domain protein 3
hsa-miR-128-3p	PALM2	paralemmin 2
hsa-miR-128-3p	PLEKHH1	pleckstrin homology, MyTH4 and FERM domain containing H1

hsa-miR-128-3p	MGAT1	mannosyl (alpha-1,3-)-glycoprotein beta-1,2-N-acetylglucosaminyltransferase
hsa-miR-128-3p	ADRA1A	adrenoceptor alpha 1A
hsa-miR-128-3p	MR1	major histocompatibility complex, class I-related
hsa-miR-128-3p	TRAPPC4	trafficking protein particle complex 4
hsa-miR-128-3p	NECAB1	N-terminal EF-hand calcium binding protein 1
hsa-miR-128-3p	IBA57	IBA57, iron-sulfur cluster assembly
hsa-miR-128-3p	XIRP1	xin actin binding repeat containing 1
hsa-miR-128-3p	STOX1	storkhead box 1
hsa-miR-128-3p	TMEM121B	transmembrane protein 121B
hsa-miR-128-3p	KLHL7	kelch like family member 7
hsa-miR-128-3p	TSEN34	tRNA splicing endonuclease subunit 34
hsa-miR-128-3p	ZNF546	zinc finger protein 546
hsa-miR-128-3p	C2CD2	C2 calcium dependent domain containing 2
hsa-miR-128-3p	TACR1	tachykinin receptor 1
hsa-miR-128-3p	HTT	huntingtin
hsa-miR-128-3p	SLX4IP	SLX4 interacting protein
hsa-miR-128-3p	ZNF627	zinc finger protein 627
hsa-miR-128-3p	EGFR	epidermal growth factor receptor
hsa-miR-128-3p	HERC3	HECT and RLD domain containing E3 ubiquitin protein ligase 3
hsa-miR-128-3p	PON2	paraoxonase 2
hsa-miR-128-3p	THAP11	THAP domain containing 11
hsa-miR-128-3p	DAD1	defender against cell death 1
hsa-miR-128-3p	ATG2A	autophagy related 2A
hsa-miR-128-3p	AGFG1	ArfGAP with FG repeats 1
hsa-miR-128-3p	AEN	apoptosis enhancing nuclease
hsa-miR-128-3p	PNRC2	proline rich nuclear receptor coactivator 2
hsa-miR-128-3p	POU2F3	POU class 2 homeobox 3
hsa-miR-128-3p	PHLDA2	pleckstrin homology like domain family A member 2
hsa-miR-128-3p	KSR2	kinase suppressor of ras 2
hsa-miR-128-3p	PEAK1	pseudopodium enriched atypical kinase 1
hsa-miR-128-3p	SEMA4D	semaphorin 4D
hsa-miR-128-3p	CHST11	carbohydrate sulfotransferase 11
hsa-miR-128-3p	RGPD4	RANBP2-like and GRIP domain containing 4
hsa-miR-128-3p	ARID5B	AT-rich interaction domain 5B
hsa-miR-128-3p	NAP1L2	nucleosome assembly protein 1 like 2
hsa-miR-128-3p	PPARG	peroxisome proliferator activated receptor gamma
hsa-miR-128-3p	PAPPA	pappalysin 1
hsa-miR-128-3p	PIK3CA	phosphatidylinositol-4,5-bisphosphate 3-kinase catalytic subunit alpha
hsa-miR-128-3p	TMEM170B	transmembrane protein 170B
hsa-miR-128-3p	BCL3	BCL3, transcription coactivator
hsa-miR-128-3p	OIT3	oncoprotein induced transcript 3
hsa-miR-128-3p	ERO1B	endoplasmic reticulum oxidoreductase 1 beta
hsa-miR-128-3p	ABCB5	ATP binding cassette subfamily B member 5
hsa-miR-128-3p	MFSD6	major facilitator superfamily domain containing 6

hsa-miR-128-3p	CAMTA1	calmodulin binding transcription activator 1
hsa-miR-128-3p	TYRP1	tyrosinase related protein 1
hsa-miR-128-3p	EARS2	glutamyl-tRNA synthetase 2, mitochondrial
hsa-miR-128-3p	TTC17	tetratricopeptide repeat domain 17
hsa-miR-128-3p	FCRL2	Fc receptor like 2
hsa-miR-128-3p	IL17RA	interleukin 17 receptor A
hsa-miR-128-3p	NR2F2	nuclear receptor subfamily 2 group F member 2
hsa-miR-128-3p	FOSB	FosB proto-oncogene, AP-1 transcription factor subunit
hsa-miR-128-3p	ZNF20	zinc finger protein 20
hsa-miR-128-3p	PTCH1	patched 1
hsa-miR-128-3p	PRMT8	protein arginine methyltransferase 8
hsa-miR-128-3p	COG7	component of oligomeric golgi complex 7
hsa-miR-128-3p	MKNK2	MAP kinase interacting serine/threonine kinase 2
hsa-miR-128-3p	EDEM3	ER degradation enhancing alpha-mannosidase like protein 3
hsa-miR-128-3p	LDLR	low density lipoprotein receptor
hsa-miR-128-3p	NRIP2	nuclear receptor interacting protein 2
hsa-miR-128-3p	MAVS	mitochondrial antiviral signaling protein
hsa-miR-128-3p	GJD3	gap junction protein delta 3
hsa-miR-128-3p	WDR36	WD repeat domain 36
hsa-miR-128-3p	TMEM237	transmembrane protein 237
hsa-miR-128-3p	SPRY2	sprouty RTK signaling antagonist 2
hsa-miR-128-3p	CD276	CD276 molecule
hsa-miR-128-3p	KCTD8	potassium channel tetramerization domain containing 8
hsa-miR-128-3p	LRRTM2	leucine rich repeat transmembrane neuronal 2
hsa-miR-128-3p	CAMK1D	calcium/calmodulin dependent protein kinase ID
hsa-miR-128-3p	AGRN	agrin
hsa-miR-128-3p	RHOT1	ras homolog family member T1
hsa-miR-128-3p	CAND1	cullin associated and neddylation dissociated 1
hsa-miR-128-3p	SOWAHA	sosondowah ankyrin repeat domain family member A
hsa-miR-128-3p	ENPP1	ectonucleotide pyrophosphatase/phosphodiesterase 1
hsa-miR-128-3p	ADAMTS16	ADAM metallopeptidase with thrombospondin type 1 motif 16
hsa-miR-128-3p	PSEN1	presenilin 1
hsa-miR-128-3p	NPTX2	neuronal pentraxin 2
hsa-miR-128-3p	ERP27	endoplasmic reticulum protein 27
hsa-miR-128-3p	FSTL3	follistatin like 3
hsa-miR-128-3p	CLOCK	clock circadian regulator
hsa-miR-128-3p	ANHX	anomalous homeobox
hsa-miR-128-3p	MOCS3	molybdenum cofactor synthesis 3
hsa-miR-128-3p	E4F1	E4F transcription factor 1
hsa-miR-128-3p	ARHGAP23	Rho GTPase activating protein 23
hsa-miR-128-3p	POLM	DNA polymerase mu
hsa-miR-128-3p	RAD54L2	RAD54 like 2
hsa-miR-128-3p	FKBP3	FKBP prolyl isomerase 3
hsa-miR-128-3p	CLDN22	claudin 22
hsa-miR-128-3p	NR1D2	nuclear receptor subfamily 1 group D member 2

hsa-miR-128-3p	PDE6A	phosphodiesterase 6A
hsa-miR-128-3p	STAC	SH3 and cysteine rich domain
hsa-miR-128-3p	LMBR1L	limb development membrane protein 1 like
hsa-miR-128-3p	ARID3B	AT-rich interaction domain 3B
hsa-miR-128-3p	ZC3H12C	zinc finger CCCH-type containing 12C
hsa-miR-128-3p	EPHA10	EPH receptor A10
hsa-miR-128-3p	DDX3Y	DEAD-box helicase 3 Y-linked
hsa-miR-128-3p	MYH10	myosin heavy chain 10
hsa-miR-128-3p	VASH2	vasohibin 2
hsa-miR-128-3p	TGIF2	TGFB induced factor homeobox 2
hsa-miR-128-3p	CACNA1C	calcium voltage-gated channel subunit alpha1 C
hsa-miR-128-3p	ANKRD24	ankyrin repeat domain 24
hsa-miR-128-3p	ADH5	alcohol dehydrogenase 5 (class III), chi polypeptide
hsa-miR-128-3p	SLC36A1	solute carrier family 36 member 1
hsa-miR-128-3p	ABCA1	ATP binding cassette subfamily A member 1
hsa-miR-128-3p	HMOX1	heme oxygenase 1
hsa-miR-128-3p	RAPGEF2	Rap guanine nucleotide exchange factor 2
hsa-miR-128-3p	HOXC6	homeobox C6
hsa-miR-128-3p	PVR	poliovirus receptor
hsa-miR-128-3p	MLPH	melanophilin
hsa-miR-128-3p	MAGEB16	MAGE family member B16
hsa-miR-128-3p	CHD2	chromodomain helicase DNA binding protein 2
hsa-miR-128-3p	SPTLC2	serine palmitoyltransferase long chain base subunit 2
hsa-miR-128-3p	C18orf65	chromosome 18 open reading frame 65
hsa-miR-128-3p	ARX	aristaless related homeobox
hsa-miR-128-3p	TSPAN13	tetraspanin 13
hsa-miR-128-3p	STYK1	serine/threonine/tyrosine kinase 1
hsa-miR-128-3p	KCNA6	potassium voltage-gated channel subfamily A member 6
hsa-miR-128-3p	RSPO3	R-spondin 3
hsa-miR-128-3p	KDM4C	lysine demethylase 4C
hsa-miR-128-3p	WSB1	WD repeat and SOCS box containing 1
hsa-miR-128-3p	SPX	spexin hormone
hsa-miR-128-3p	RGS1	regulator of G protein signaling 1
hsa-miR-128-3p	CBX5	chromobox 5
hsa-miR-128-3p	MAN2A1	mannosidase alpha class 2A member 1
hsa-miR-128-3p	RARB	retinoic acid receptor beta
hsa-miR-128-3p	TSTD2	thiosulfate sulfurtransferase like domain containing 2
hsa-miR-128-3p	HSPD1	heat shock protein family D (Hsp60) member 1
hsa-miR-128-3p	TNRC6B	trinucleotide repeat containing 6B
hsa-miR-128-3p	ZNF124	zinc finger protein 124
hsa-miR-128-3p	DEPDC4	DEP domain containing 4
hsa-miR-128-3p	SCN2B	sodium voltage-gated channel beta subunit 2
hsa-miR-128-3p	SLCO1A2	solute carrier organic anion transporter family member 1A2
hsa-miR-128-3p	KIAA1211	KIAA1211
hsa-miR-128-3p	DLG2	discs large MAGUK scaffold protein 2

hsa-miR-128-3p	NKTR	natural killer cell triggering receptor
hsa-miR-128-3p	TET2	tet methylcytosine dioxygenase 2
hsa-miR-128-3p	LOC101928841	collagen alpha-1(II) chain-like
hsa-miR-128-3p	DEPDC1B	DEP domain containing 1B
hsa-miR-128-3p	CEP135	centrosomal protein 135
hsa-miR-128-3p	FAM118A	family with sequence similarity 118 member A
hsa-miR-128-3p	LMNB1	lamin B1
hsa-miR-128-3p	NCAN	neurocan
hsa-miR-128-3p	KAT2A	lysine acetyltransferase 2A
hsa-miR-128-3p	PPP4R2	protein phosphatase 4 regulatory subunit 2
hsa-miR-128-3p	SUCO	SUN domain containing ossification factor
hsa-miR-128-3p	RAB33B	RAB33B, member RAS oncogene family
hsa-miR-128-3p	PAQR9	progestin and adipoQ receptor family member 9
hsa-miR-128-3p	TNFSF8	TNF superfamily member 8
hsa-miR-128-3p	RLIM	ring finger protein, LIM domain interacting
hsa-miR-128-3p	SLFN11	schlafen family member 11
hsa-miR-128-3p	ZC3H13	zinc finger CCCH-type containing 13
hsa-miR-128-3p	PTGDR	prostaglandin D2 receptor
hsa-miR-128-3p	EXD1	exonuclease 3'-5' domain containing 1
hsa-miR-128-3p	ZNF385C	zinc finger protein 385C
hsa-miR-128-3p	GLRX	glutaredoxin
hsa-miR-128-3p	METTL8	methyltransferase like 8
hsa-miR-128-3p	ARFGEF3	ARFGEF family member 3
hsa-miR-128-3p	TOPBP1	DNA topoisomerase II binding protein 1
hsa-miR-128-3p	SMCR8	Smith-Magenis syndrome chromosome region, candidate 8
hsa-miR-128-3p	TMEM39A	transmembrane protein 39A
hsa-miR-128-3p	MSRB3	methionine sulfoxide reductase B3
hsa-miR-128-3p	LHX1	LIM homeobox 1
hsa-miR-128-3p	ERI1	exoribonuclease 1
hsa-miR-128-3p	KBTBD8	kelch repeat and BTB domain containing 8
hsa-miR-128-3p	CYP4F2	cytochrome P450 family 4 subfamily F member 2
hsa-miR-128-3p	CEBPE	CCAAT enhancer binding protein epsilon
hsa-miR-128-3p	IL16	interleukin 16
hsa-miR-128-3p	RCAN3	RCAN family member 3
hsa-miR-128-3p	PTCD2	pentatricopeptide repeat domain 2
hsa-miR-128-3p	DIRAS2	DIRAS family GTPase 2
hsa-miR-128-3p	SLC26A2	solute carrier family 26 member 2
hsa-miR-128-3p	RIMS1	regulating synaptic membrane exocytosis 1
hsa-miR-128-3p	C16orf54	chromosome 16 open reading frame 54
hsa-miR-128-3p	CIART	circadian associated repressor of transcription
hsa-miR-128-3p	HDHD2	haloacid dehalogenase like hydrolase domain containing 2
hsa-miR-128-3p	PTGER4	prostaglandin E receptor 4
hsa-miR-128-3p	NEK1	NIMA related kinase 1
hsa-miR-128-3p	APAF1	apoptotic peptidase activating factor 1
hsa-miR-128-3p	STK3	serine/threonine kinase 3

hsa-miR-128-3p	EIF4EBP2	eukaryotic translation initiation factor 4E binding protein 2
hsa-miR-128-3p	ZNF587B	zinc finger protein 587B
hsa-miR-128-3p	CDS2	CDP-diacylglycerol synthase 2
hsa-miR-128-3p	ADAM19	ADAM metallopeptidase domain 19
hsa-miR-128-3p	GPR35	G protein-coupled receptor 35
hsa-miR-128-3p	THRB	thyroid hormone receptor beta
hsa-miR-128-3p	MPP2	membrane palmitoylated protein 2
hsa-miR-128-3p	MLYCD	malonyl-CoA decarboxylase
hsa-miR-128-3p	TNPO3	transportin 3
hsa-miR-128-3p	MARK1	microtubule affinity regulating kinase 1
hsa-miR-128-3p	EPB41L2	erythrocyte membrane protein band 4.1 like 2
hsa-miR-128-3p	UMAD1	UBAP1-MVB12-associated (UMA) domain containing 1
hsa-miR-128-3p	PTAFR	platelet activating factor receptor
hsa-miR-128-3p	IFNA1	interferon alpha 1
hsa-miR-128-3p	TBP	TATA-box binding protein
hsa-miR-128-3p	TRIM50	tripartite motif containing 50
hsa-miR-128-3p	LETMD1	LETM1 domain containing 1
hsa-miR-128-3p	PIGT	phosphatidylinositol glycan anchor biosynthesis class T
hsa-miR-128-3p	ZSCAN20	zinc finger and SCAN domain containing 20
hsa-miR-128-3p	CD93	CD93 molecule
hsa-miR-128-3p	SP100	SP100 nuclear antigen
hsa-miR-128-3p	XKRY2	XK related, Y-linked 2
hsa-miR-128-3p	GVQW2	GVQW motif containing 2
hsa-miR-128-3p	CYB561D1	cytochrome b561 family member D1
hsa-miR-128-3p	TBCC	tubulin folding cofactor C
hsa-miR-128-3p	EFNB2	ephrin B2
hsa-miR-128-3p	HIVEP3	human immunodeficiency virus type I enhancer binding protein 3
hsa-miR-128-3p	RPGRIP1L	RPGRIP1 like
hsa-miR-128-3p	TTC39C	tetratricopeptide repeat domain 39C
hsa-miR-128-3p	NCOA5	nuclear receptor coactivator 5
hsa-miR-128-3p	HSPA1L	heat shock protein family A (Hsp70) member 1 like
hsa-miR-128-3p	ARHGEF11	Rho guanine nucleotide exchange factor 11
hsa-miR-128-3p	XKRY	XK related, Y-linked
hsa-miR-128-3p	VEGFB	vascular endothelial growth factor B
hsa-miR-128-3p	STAM	signal transducing adaptor molecule
hsa-miR-128-3p	TNFAIP3	TNF alpha induced protein 3
hsa-miR-128-3p	DPYS	dihydropyrimidinase
hsa-miR-128-3p	PAXBP1	PAX3 and PAX7 binding protein 1
hsa-miR-128-3p	NRIP1	nuclear receptor interacting protein 1
hsa-miR-132-3p	CDK19	cyclin dependent kinase 19
hsa-miR-132-3p	TIMM9	translocase of inner mitochondrial membrane 9
hsa-miR-132-3p	LEMD3	LEM domain containing 3
hsa-miR-132-3p	RGS7BP	regulator of G protein signaling 7 binding protein
hsa-miR-132-3p	MIA3	MIA SH3 domain ER export factor 3
hsa-miR-132-3p	MIER1	MIER1 transcriptional regulator

hsa-miR-132-3p	SOX5	SRY-box 5
hsa-miR-132-3p	CBLL1	Cbl proto-oncogene like 1
hsa-miR-132-3p	METTL25	methyltransferase like 25
hsa-miR-132-3p	GTF2H1	general transcription factor IIH subunit 1
hsa-miR-132-3p	MEX3C	mex-3 RNA binding family member C
hsa-miR-132-3p	MAPK1	mitogen-activated protein kinase 1
hsa-miR-132-3p	RASA1	RAS p21 protein activator 1
hsa-miR-132-3p	CHD1	chromodomain helicase DNA binding protein 1
hsa-miR-132-3p	MELK	maternal embryonic leucine zipper kinase
hsa-miR-132-3p	CSDE1	cold shock domain containing E1
hsa-miR-132-3p	TUT4	terminal uridylyl transferase 4
hsa-miR-132-3p	ZBTB20	zinc finger and BTB domain containing 20
hsa-miR-132-3p	BRWD1	bromodomain and WD repeat domain containing 1
hsa-miR-132-3p	SSH2	slingshot protein phosphatase 2
hsa-miR-132-3p	FEM1C	fem-1 homolog C
hsa-miR-132-3p	SGK3	serum/glucocorticoid regulated kinase family member 3
hsa-miR-132-3p	USP38	ubiquitin specific peptidase 38
hsa-miR-132-3p	DAZAP2	DAZ associated protein 2
hsa-miR-132-3p	VDAC2	voltage dependent anion channel 2
hsa-miR-132-3p	CCDC88A	coiled-coil domain containing 88A
hsa-miR-132-3p	MTF2	metal response element binding transcription factor 2
hsa-miR-132-3p	ZNF521	zinc finger protein 521
hsa-miR-132-3p	SOD2	superoxide dismutase 2
hsa-miR-132-3p	MYCBP2	MYC binding protein 2, E3 ubiquitin protein ligase
hsa-miR-132-3p	PTBP2	polypyrimidine tract binding protein 2
hsa-miR-132-3p	HBEGF	heparin binding EGF like growth factor
hsa-miR-132-3p	ZNF652	zinc finger protein 652
hsa-miR-132-3p	PCDH10	protocadherin 10
hsa-miR-132-3p	ETNK1	ethanolamine kinase 1
hsa-miR-132-3p	TJAP1	tight junction associated protein 1
hsa-miR-132-3p	SERP1	stress associated endoplasmic reticulum protein 1
hsa-miR-132-3p	SETD5	SET domain containing 5
hsa-miR-132-3p	NFE2L2	nuclear factor, erythroid 2 like 2
hsa-miR-132-3p	KLF7	Kruppel like factor 7
hsa-miR-132-3p	OSBPL8	oxysterol binding protein like 8
hsa-miR-132-3p	ZNF516	zinc finger protein 516
hsa-miR-132-3p	SPPL3	signal peptide peptidase like 3
hsa-miR-132-3p	SLC6A1	solute carrier family 6 member 1
hsa-miR-132-3p	CCN2	cellular communication network factor 2
hsa-miR-132-3p	DAAM1	dishevelled associated activator of morphogenesis 1
hsa-miR-132-3p	HHIP	hedgehog interacting protein
hsa-miR-132-3p	NMNAT2	nicotinamide nucleotide adenylyltransferase 2
hsa-miR-132-3p	HNRNPH1	heterogeneous nuclear ribonucleoprotein H1
hsa-miR-132-3p	NOVA1	NOVA alternative splicing regulator 1
hsa-miR-132-3p	SKAP2	src kinase associated phosphoprotein 2

hsa-miR-132-3p	SAP30L	SAP30 like
hsa-miR-132-3p	TLN2	talin 2
hsa-miR-132-3p	FBXL20	F-box and leucine rich repeat protein 20
hsa-miR-132-3p	GMFB	glia maturation factor beta
hsa-miR-132-3p	PPM1G	protein phosphatase, Mg2+/Mn2+ dependent 1G
hsa-miR-132-3p	NVL	nuclear VCP-like
hsa-miR-132-3p	RUFY3	RUN and FYVE domain containing 3
hsa-miR-132-3p	RPP14	ribonuclease P/MRP subunit p14
hsa-miR-132-3p	ELMSAN1	ELM2 and Myb/SANT domain containing 1
hsa-miR-132-3p	SLC25A28	solute carrier family 25 member 28
hsa-miR-132-3p	DCC	DCC netrin 1 receptor
hsa-miR-132-3p	FAM227A	family with sequence similarity 227 member A
hsa-miR-132-3p	ASF1A	anti-silencing function 1A histone chaperone
hsa-miR-132-3p	RB1	RB transcriptional corepressor 1
hsa-miR-132-3p	NACC2	NACC family member 2
hsa-miR-132-3p	L3MBTL3	L3MBTL3, histone methyl-lysine binding protein
hsa-miR-132-3p	MEF2A	myocyte enhancer factor 2A
hsa-miR-132-3p	ACVR2B	activin A receptor type 2B
hsa-miR-132-3p	KCMF1	potassium channel modulatory factor 1
hsa-miR-132-3p	STX16	syntaxin 16
hsa-miR-132-3p	USP9X	ubiquitin specific peptidase 9 X-linked
hsa-miR-132-3p	TMEM164	transmembrane protein 164
hsa-miR-132-3p	PHF12	PHD finger protein 12
hsa-miR-132-3p	PAM	peptidylglycine alpha-amidating monooxygenase
hsa-miR-132-3p	CAMSAP2	calmodulin regulated spectrin associated protein family member 2
hsa-miR-132-3p	sept-08	septin 8
hsa-miR-132-3p	ENPP4	ectonucleotide pyrophosphatase/phosphodiesterase 4
hsa-miR-132-3p	ATXN1	ataxin 1
hsa-miR-132-3p	FAM167A	family with sequence similarity 167 member A
hsa-miR-132-3p	BRI3	brain protein I3
hsa-miR-132-3p	MIS12	MIS12, kinetochore complex component
hsa-miR-132-3p	SIRT1	sirtuin 1
hsa-miR-132-3p	C8orf44-SGK3	C8orf44-SGK3 readthrough
hsa-miR-132-3p	KDM5A	lysine demethylase 5A
hsa-miR-132-3p	ALKAL1	ALK and LTK ligand 1
hsa-miR-132-3p	SOX11	SRY-box 11
hsa-miR-132-3p	GRM3	glutamate metabotropic receptor 3
hsa-miR-132-3p	CELSR3	cadherin EGF LAG seven-pass G-type receptor 3
hsa-miR-132-3p	SLC26A7	solute carrier family 26 member 7
hsa-miR-132-3p	SYN2	synapsin II
hsa-miR-132-3p	LIN28B	lin-28 homolog B
hsa-miR-132-3p	NLK	nemo like kinase
hsa-miR-132-3p	NREP	neuronal regeneration related protein
hsa-miR-132-3p	SEC16A	SEC16 homolog A, endoplasmic reticulum export factor

hsa-miR-132-3p	KCNK2	potassium two pore domain channel subfamily K member 2
hsa-miR-132-3p	FBXO9	F-box protein 9
hsa-miR-132-3p	CLMN	calmin
hsa-miR-132-3p	FAM91A1	family with sequence similarity 91 member A1
hsa-miR-132-3p	COL11A1	collagen type XI alpha 1 chain
hsa-miR-132-3p	DPYSL3	dihydropyrimidinase like 3
hsa-miR-132-3p	HNRNPM	heterogeneous nuclear ribonucleoprotein M
hsa-miR-132-3p	SEMA6A	semaphorin 6A
hsa-miR-132-3p	SCN3A	sodium voltage-gated channel alpha subunit 3
hsa-miR-132-3p	SALL4	spalt like transcription factor 4
hsa-miR-132-3p	BOLL	boule homolog, RNA binding protein
hsa-miR-132-3p	SALL1	spalt like transcription factor 1
hsa-miR-132-3p	ARID2	AT-rich interaction domain 2
hsa-miR-132-3p	ARMC8	armadillo repeat containing 8
hsa-miR-132-3p	RAD54L2	RAD54 like 2
hsa-miR-132-3p	INPP5K	inositol polyphosphate-5-phosphatase K
hsa-miR-132-3p	SPRED1	sprouty related EVH1 domain containing 1
hsa-miR-132-3p	ZHX1	zinc fingers and homeoboxes 1
hsa-miR-132-3p	CCDC71L	coiled-coil domain containing 71 like
hsa-miR-132-3p	NFATC2	nuclear factor of activated T cells 2
hsa-miR-132-3p	LRRC58	leucine rich repeat containing 58
hsa-miR-132-3p	SRGAP1	SLIT-ROBO Rho GTPase activating protein 1
hsa-miR-132-3p	ADAMTS5	ADAM metallopeptidase with thrombospondin type 1 motif 5
hsa-miR-132-3p	GPD2	glycerol-3-phosphate dehydrogenase 2
hsa-miR-132-3p	CACNG2	calcium voltage-gated channel auxiliary subunit gamma 2
hsa-miR-132-3p	CALU	calumenin
hsa-miR-132-3p	TMEM64	transmembrane protein 64
hsa-miR-132-3p	PGM5	phosphoglucomutase 5
hsa-miR-132-3p	ARID1B	AT-rich interaction domain 1B
hsa-miR-132-3p	PSMD12	proteasome 26S subunit, non-ATPase 12
hsa-miR-132-3p	KRTAP4-1	keratin associated protein 4-1
hsa-miR-132-3p	WDR5B	WD repeat domain 5B
hsa-miR-132-3p	RICTOR	RPTOR independent companion of MTOR complex 2
hsa-miR-132-3p	ZNF451	zinc finger protein 451
hsa-miR-132-3p	EP300	E1A binding protein p300
hsa-miR-132-3p	CD300LF	CD300 molecule like family member f
hsa-miR-132-3p	MED9	mediator complex subunit 9
hsa-miR-132-3p	SPATA13	spermatogenesis associated 13
hsa-miR-132-3p	CCDC34	coiled-coil domain containing 34
hsa-miR-132-3p	AZIN1	antizyme inhibitor 1
hsa-miR-132-3p	POM121	POM121 transmembrane nucleoporin
hsa-miR-132-3p	TSPAN6	tetraspanin 6
hsa-miR-132-3p	SLC1A3	solute carrier family 1 member 3

hsa-miR-132-3p	AMD1	adenosylmethionine decarboxylase 1
hsa-miR-132-3p	GPATCH2L	G-patch domain containing 2 like
hsa-miR-132-3p	CACNB1	calcium voltage-gated channel auxiliary subunit beta 1
hsa-miR-132-3p	E2F5	E2F transcription factor 5
hsa-miR-132-3p	GDF5	growth differentiation factor 5
hsa-miR-132-3p	FOXO3	forkhead box O3
hsa-miR-132-3p	AGO1	argonaute RISC catalytic component 1
hsa-miR-132-3p	KCNN3	potassium calcium-activated channel subfamily N member 3
hsa-miR-132-3p	MUC13	mucin 13, cell surface associated
hsa-miR-132-3p	G3BP2	G3BP stress granule assembly factor 2
hsa-miR-132-3p	SS18	SS18, nBAF chromatin remodeling complex subunit
hsa-miR-132-3p	CBWD1	COBW domain containing 1
hsa-miR-132-3p	NTNG1	netrin G1
hsa-miR-132-3p	KCNA6	potassium voltage-gated channel subfamily A member 6
hsa-miR-132-3p	TMEM136	transmembrane protein 136
hsa-miR-132-3p	CBWD3	COBW domain containing 3
hsa-miR-132-3p	RPL31	ribosomal protein L31
hsa-miR-132-3p	SIX4	SIX homeobox 4
hsa-miR-132-3p	DYRK2	dual specificity tyrosine phosphorylation regulated kinase 2
hsa-miR-132-3p	SLC30A6	solute carrier family 30 member 6
hsa-miR-132-3p	TMEM178B	transmembrane protein 178B
hsa-miR-132-3p	TRNAU1AP	tRNA selenocysteine 1 associated protein 1
hsa-miR-132-3p	ZYG11A	zyg-11 family member A, cell cycle regulator
hsa-miR-132-3p	TRDN	triadin
hsa-miR-132-3p	H2AFZ	H2A histone family member Z
hsa-miR-132-3p	DDX5	DEAD-box helicase 5
hsa-miR-132-3p	RHOQ	ras homolog family member Q
hsa-miR-132-3p	DUSP9	dual specificity phosphatase 9
hsa-miR-132-3p	SIPA1L2	signal induced proliferation associated 1 like 2
hsa-miR-132-3p	DIPK2A	divergent protein kinase domain 2A
hsa-miR-132-3p	AEBP2	AE binding protein 2
hsa-miR-132-3p	LSM11	LSM11, U7 small nuclear RNA associated
hsa-miR-132-3p	CNR1	cannabinoid receptor 1
hsa-miR-132-3p	DCUN1D4	defective in cullin neddylation 1 domain containing 4
hsa-miR-132-3p	CBWD2	COBW domain containing 2
hsa-miR-132-3p	KIAA2026	KIAA2026
hsa-miR-132-3p	FAM76B	family with sequence similarity 76 member B
hsa-miR-132-3p	GVQW2	GVQW motif containing 2
hsa-miR-132-3p	MTMR10	myotubularin related protein 10
hsa-miR-132-3p	ADCY3	adenylate cyclase 3
hsa-miR-132-3p	FRS2	fibroblast growth factor receptor substrate 2
hsa-miR-132-3p	ATP10D	ATPase phospholipid transporting 10D (putative)
hsa-miR-132-3p	AMER2	APC membrane recruitment protein 2

hsa-miR-132-3p	EFCAB9	EF-hand calcium binding domain 9
hsa-miR-132-3p	CC2D1B	coiled-coil and C2 domain containing 1B
hsa-miR-132-3p	STAG1	stromal antigen 1
hsa-miR-132-3p	MAP3K3	mitogen-activated protein kinase kinase kinase 3
hsa-miR-132-3p	ARNT	aryl hydrocarbon receptor nuclear translocator
hsa-miR-132-3p	LRRFIP1	LRR binding FLII interacting protein 1
hsa-miR-132-3p	CASP7	caspase 7
hsa-miR-132-3p	PYURF	PIGY upstream reading frame
hsa-miR-132-3p	MAOA	monoamine oxidase A
hsa-miR-132-3p	EPB41L5	erythrocyte membrane protein band 4.1 like 5
hsa-miR-132-3p	ZBTB18	zinc finger and BTB domain containing 18
hsa-miR-132-3p	ZFP3	ZFP3 zinc finger protein
hsa-miR-132-3p	TGS1	trimethylguanosine synthase 1
hsa-miR-132-3p	PBOV1	prostate and breast cancer overexpressed 1
hsa-miR-132-3p	TMEM106B	transmembrane protein 106B
hsa-miR-132-3p	INAFM2	InaF motif containing 2
hsa-miR-132-3p	MTMR1	myotubularin related protein 1
hsa-miR-132-3p	FOXA1	forkhead box A1
hsa-miR-132-3p	TTK	TTK protein kinase
hsa-miR-132-3p	ELOC	elongin C
hsa-miR-132-3p	HIC2	HIC ZBTB transcriptional repressor 2
hsa-miR-132-3p	FAM184A	family with sequence similarity 184 member A
hsa-miR-132-3p	RFX3	regulatory factor X3
hsa-miR-132-3p	FMN1	formin 1
hsa-miR-132-3p	USP9Y	ubiquitin specific peptidase 9 Y-linked
hsa-miR-132-3p	NDUFAF6	NADH:ubiquinone oxidoreductase complex assembly factor 6
hsa-miR-132-3p	MAPKAP1	mitogen-activated protein kinase associated protein 1
hsa-miR-132-3p	ISL1	ISL LIM homeobox 1
hsa-miR-132-3p	CNIH1	cornichon family AMPA receptor auxiliary protein 1
hsa-miR-132-3p	CXorf40A	chromosome X open reading frame 40A
hsa-miR-132-3p	HYDIN	HYDIN, axonemal central pair apparatus protein
hsa-miR-132-3p	ATP6AP1L	ATPase H+ transporting accessory protein 1 like
hsa-miR-132-3p	MCOLN3	mucolipin 3
hsa-miR-132-3p	PIK3R1	phosphoinositide-3-kinase regulatory subunit 1
hsa-miR-132-3p	PPP2CB	protein phosphatase 2 catalytic subunit beta
hsa-miR-132-3p	EPM2AIP1	EPM2A interacting protein 1
hsa-miR-132-3p	SMAD2	SMAD family member 2
hsa-miR-132-3p	RTN4	reticulon 4
hsa-miR-132-3p	CERS2	ceramide synthase 2
hsa-miR-132-3p	MISP3	MISP family member 3
hsa-miR-132-3p	PNKD	PNKD, MBL domain containing
hsa-miR-132-3p	DYNC1LI2	dynein cytoplasmic 1 light intermediate chain 2
hsa-miR-132-3p	HECTD1	HECT domain E3 ubiquitin protein ligase 1
hsa-miR-132-3p	MRM2	mitochondrial rRNA methyltransferase 2

hsa-miR-132-3p	TMEM47	transmembrane protein 47
hsa-miR-132-3p	DNAJC10	DnaJ heat shock protein family (Hsp40) member C10
hsa-miR-132-3p	GRIP1	glutamate receptor interacting protein 1
hsa-miR-132-3p	ARID4B	AT-rich interaction domain 4B
hsa-miR-132-3p	RPL13A	ribosomal protein L13a
hsa-miR-132-3p	APLP2	amyloid beta precursor like protein 2
hsa-miR-132-3p	SEC62	SEC62 homolog, preprotein translocation factor
hsa-miR-132-3p	PPAT	phosphoribosyl pyrophosphate amidotransferase
hsa-miR-132-3p	PSMA2	proteasome subunit alpha 2
hsa-miR-132-3p	ACSL4	acyl-CoA synthetase long chain family member 4
hsa-miR-132-3p	HAPLN1	hyaluronan and proteoglycan link protein 1
hsa-miR-132-3p	TRIM2	tripartite motif containing 2
hsa-miR-132-3p	MINDY3	MINDY lysine 48 deubiquitinase 3
hsa-miR-132-3p	SDF2	stromal cell derived factor 2
hsa-miR-132-3p	SLK	STE20 like kinase
hsa-miR-132-3p	TC2N	tandem C2 domains, nuclear
hsa-miR-132-3p	C9orf72	chromosome 9 open reading frame 72
hsa-miR-132-3p	HAO1	hydroxyacid oxidase 1
hsa-miR-132-3p	TMEFF1	transmembrane protein with EGF like and two follistatin like domains 1
hsa-miR-132-3p	LZTS3	leucine zipper tumor suppressor family member 3
hsa-miR-132-3p	BCAN	brevican
hsa-miR-132-3p	ANP32A	acidic nuclear phosphoprotein 32 family member A
hsa-miR-132-3p	SRP72	signal recognition particle 72
hsa-miR-132-3p	ABHD5	abhydrolase domain containing 5
hsa-miR-132-3p	SLBP	stem-loop binding protein
hsa-miR-132-3p	KLHL11	kelch like family member 11
hsa-miR-132-3p	KIAA1211L	KIAA1211 like
hsa-miR-132-3p	HRNR	hornerin
hsa-miR-132-3p	C22orf39	chromosome 22 open reading frame 39
hsa-miR-132-3p	ATXN7	ataxin 7
hsa-miR-132-3p	NFAT5	nuclear factor of activated T cells 5
hsa-miR-132-3p	PHF20L1	PHD finger protein 20 like 1
hsa-miR-132-3p	PRDM15	PR/SET domain 15
hsa-miR-132-3p	HEXB	hexosaminidase subunit beta
hsa-miR-132-3p	KCNH5	potassium voltage-gated channel subfamily H member 5
hsa-miR-132-3p	MEX3A	mex-3 RNA binding family member A
hsa-miR-132-3p	BICD2	BICD cargo adaptor 2
hsa-miR-132-3p	WDCP	WD repeat and coiled coil containing
hsa-miR-132-3p	SCN1A	sodium voltage-gated channel alpha subunit 1
hsa-miR-132-3p	SOS1	SOS Ras/Rac guanine nucleotide exchange factor 1
hsa-miR-132-3p	PRPF4B	pre-mRNA processing factor 4B
hsa-miR-132-3p	COL5A2	collagen type V alpha 2 chain
hsa-miR-132-3p	HS2ST1	heparan sulfate 2-O-sulfotransferase 1
hsa-miR-132-3p	CNTNAP3B	contactin associated protein like 3B

hsa-miR-132-3p	PRDM6	PR/SET domain 6
hsa-miR-132-3p	PDE7A	phosphodiesterase 7A
hsa-miR-132-3p	FANK1	fibronectin type III and ankyrin repeat domains 1
hsa-miR-132-3p	DENND1B	DENN domain containing 1B
hsa-miR-132-3p	TMEM41B	transmembrane protein 41B
hsa-miR-132-3p	CNTNAP3	contactin associated protein like 3
hsa-miR-132-3p	PRKD3	protein kinase D3
hsa-miR-132-3p	MAP3K20	mitogen-activated protein kinase kinase kinase 20
hsa-miR-132-3p	MECP2	methyl-CpG binding protein 2
hsa-miR-132-3p	ZNF236	zinc finger protein 236
hsa-miR-132-3p	EPC1	enhancer of polycomb homolog 1
hsa-miR-132-3p	SLC38A10	solute carrier family 38 member 10
hsa-miR-132-3p	PBDC1	polysaccharide biosynthesis domain containing 1
hsa-miR-132-3p	GNA12	G protein subunit alpha 12
hsa-miR-132-3p	MFSD11	major facilitator superfamily domain containing 11
hsa-miR-132-3p	DNAJA2	DnaJ heat shock protein family (Hsp40) member A2
hsa-miR-132-3p	BCL2L11	BCL2 like 11
hsa-miR-132-3p	PPM1E	protein phosphatase, Mg2+/Mn2+ dependent 1E
hsa-miR-132-3p	EGR1	early growth response 1
hsa-miR-132-3p	SOWAHA	sosondowah ankyrin repeat domain family member A
hsa-miR-132-3p	SFMBT1	Scm like with four mbt domains 1
hsa-miR-132-3p	SLC20A1	solute carrier family 20 member 1
hsa-miR-132-3p	RAD21	RAD21 cohesin complex component
hsa-miR-132-3p	EIF4A2	eukaryotic translation initiation factor 4A2
hsa-miR-132-3p	PTAR1	protein prenyltransferase alpha subunit repeat containing 1
hsa-miR-132-3p	SMAD5	SMAD family member 5
hsa-miR-132-3p	PAIP2	poly(A) binding protein interacting protein 2
hsa-miR-132-3p	NCKAP5	NCK associated protein 5
hsa-miR-132-3p	TSPYL5	TSPY like 5
hsa-miR-132-3p	YIPF5	Yip1 domain family member 5
hsa-miR-132-3p	TET2	tet methylcytosine dioxygenase 2
hsa-miR-132-3p	RAB6B	RAB6B, member RAS oncogene family
hsa-miR-132-3p	NEUROD4	neuronal differentiation 4
hsa-miR-132-3p	AMOT	angiomotin
hsa-miR-132-3p	OPRM1	opioid receptor mu 1
hsa-miR-132-3p	SEPHS1	selenophosphate synthetase 1
hsa-miR-132-3p	CAPRIN1	cell cycle associated protein 1
hsa-miR-132-3p	FBXO28	F-box protein 28
hsa-miR-132-3p	PKD1L1	polycystin 1 like 1, transient receptor potential channel interacting
hsa-miR-132-3p	SEC14L2	SEC14 like lipid binding 2
hsa-miR-132-3p	RNASEH2B	ribonuclease H2 subunit B
hsa-miR-132-3p	KITLG	KIT ligand
hsa-miR-132-3p	UBE2D3	ubiquitin conjugating enzyme E2 D3

hsa-miR-132-3p	MTPN	myotrophin
hsa-miR-132-3p	CENPQ	centromere protein Q
hsa-miR-132-3p	SEMA4G	semaphorin 4G
hsa-miR-132-3p	PTGS2	prostaglandin-endoperoxide synthase 2
hsa-miR-132-3p	WT1	Wilms tumor 1
hsa-miR-132-3p	REEP3	receptor accessory protein 3
hsa-miR-132-3p	SRGAP2B	SLIT-ROBO Rho GTPase activating protein 2B
hsa-miR-132-3p	RAB28	RAB28, member RAS oncogene family
hsa-miR-132-3p	ATL3	atlastin GTPase 3
hsa-miR-132-3p	ADCYAP1	adenylate cyclase activating polypeptide 1
hsa-miR-132-3p	ANKRD29	ankyrin repeat domain 29
hsa-miR-132-3p	CTCFL	CCCTC-binding factor like
hsa-miR-132-3p	GABRG1	gamma-aminobutyric acid type A receptor gamma1 subunit
hsa-miR-132-3p	C18orf25	chromosome 18 open reading frame 25
hsa-miR-132-3p	FZD6	frizzled class receptor 6
hsa-miR-132-3p	GPBP1	GC-rich promoter binding protein 1
hsa-miR-132-3p	CCDC169	coiled-coil domain containing 169
hsa-miR-132-3p	MTCL1	microtubule crosslinking factor 1
hsa-miR-132-3p	UHMK1	U2AF homology motif kinase 1
hsa-miR-132-3p	ARHGAP32	Rho GTPase activating protein 32
hsa-miR-132-3p	ZNF644	zinc finger protein 644
hsa-miR-132-3p	BTBD1	BTB domain containing 1
hsa-miR-132-3p	UNC13A	unc-13 homolog A
hsa-miR-132-3p	CLDN16	claudin 16
hsa-miR-132-3p	PTPRD	protein tyrosine phosphatase, receptor type D
hsa-miR-132-3p	ARHGEF40	Rho guanine nucleotide exchange factor 40
hsa-miR-132-3p	BRCA1	BRCA1, DNA repair associated
hsa-miR-132-3p	MAN1A2	mannosidase alpha class 1A member 2
hsa-miR-132-3p	ARL1	ADP ribosylation factor like GTPase 1
hsa-miR-132-3p	APAF1	apoptotic peptidase activating factor 1
hsa-miR-132-3p	PGR	progesterone receptor
hsa-miR-132-3p	SLC1A4	solute carrier family 1 member 4
hsa-miR-132-3p	BBX	BBX, HMG-box containing
hsa-miR-132-3p	ARGFX	arginine-fifty homeobox
hsa-miR-132-3p	MEIS2	Meis homeobox 2
hsa-miR-132-3p	HOMER1	homer scaffold protein 1
hsa-miR-132-3p	OLFM1	olfactomedin 1
hsa-miR-132-3p	CACUL1	CDK2 associated cullin domain 1
hsa-miR-132-3p	SLC7A1	solute carrier family 7 member 1
hsa-miR-132-3p	FGF7	fibroblast growth factor 7
hsa-miR-132-3p	DNAJC27	DnaJ heat shock protein family (Hsp40) member C27
hsa-miR-132-3p	FAM222B	family with sequence similarity 222 member B
hsa-miR-132-3p	VAPA	VAMP associated protein A
hsa-miR-132-3p	NIPSNAP3B	nipsnap homolog 3B

hsa-miR-132-3p	MECOM	MDS1 and EVI1 complex locus
hsa-miR-132-3p	POM121C	POM121 transmembrane nucleoporin C
hsa-miR-132-3p	VPS13A	vacuolar protein sorting 13 homolog A
hsa-miR-132-3p	MX2	MX dynamin like GTPase 2
hsa-miR-132-3p	ZNF133	zinc finger protein 133
hsa-miR-132-3p	PIK3CA	phosphatidylinositol-4,5-bisphosphate 3-kinase catalytic subunit alpha
hsa-miR-132-3p	ADRA1A	adrenoceptor alpha 1A
hsa-miR-132-3p	IL17RD	interleukin 17 receptor D
hsa-miR-132-3p	OTOGL	otogelin like
hsa-miR-132-3p	ZNF229	zinc finger protein 229
hsa-miR-132-3p	HIP1R	huntingtin interacting protein 1 related
hsa-miR-132-3p	DCUN1D1	defective in cullin neddylation 1 domain containing 1
hsa-miR-132-3p	TGFBI	transforming growth factor beta induced
hsa-miR-132-3p	NOXRED1	NADP dependent oxidoreductase domain containing 1
hsa-miR-132-3p	SPTY2D1	SPT2 chromatin protein domain containing 1
hsa-miR-132-3p	PLPP5	phospholipid phosphatase 5
hsa-miR-132-3p	SPTSSA	serine palmitoyltransferase small subunit A
hsa-miR-132-3p	ZNF678	zinc finger protein 678
hsa-miR-132-3p	FAM126B	family with sequence similarity 126 member B
hsa-miR-132-3p	MMP16	matrix metallopeptidase 16
hsa-miR-132-3p	EBF3	EBF transcription factor 3
hsa-miR-132-3p	BARD1	BRCA1 associated RING domain 1
hsa-miR-132-3p	BTAF1	B-TFIID TATA-box binding protein associated factor 1
hsa-miR-132-3p	EMSY	EMSY, BRCA2 interacting transcriptional repressor
hsa-miR-132-3p	GNB1	G protein subunit beta 1
hsa-miR-132-3p	SPATS1	spermatogenesis associated serine rich 1
hsa-miR-132-3p	SLC31A1	solute carrier family 31 member 1
hsa-miR-132-3p	B4GALT6	beta-1,4-galactosyltransferase 6
hsa-miR-132-3p	RPGR	retinitis pigmentosa GTPase regulator
hsa-miR-132-3p	PDE5A	phosphodiesterase 5A
hsa-miR-132-3p	KHDRBS2	KH RNA binding domain containing, signal transduction associated 2
hsa-miR-132-3p	KIF21B	kinesin family member 21B
hsa-miR-132-3p	MANEA	mannosidase endo-alpha
hsa-miR-132-3p	BNC2	basonuclin 2
hsa-miR-132-3p	PPP2R5C	protein phosphatase 2 regulatory subunit B'gamma
hsa-miR-132-3p	ZEB2	zinc finger E-box binding homeobox 2
hsa-miR-132-3p	UBAC1	UBA domain containing 1
hsa-miR-132-3p	LYN	LYN proto-oncogene, Src family tyrosine kinase
hsa-miR-132-3p	ERMP1	endoplasmic reticulum metallopeptidase 1
hsa-miR-132-3p	RC3H1	ring finger and CCCH-type domains 1
hsa-miR-132-3p	ZNF507	zinc finger protein 507
hsa-miR-132-3p	TGFB2	transforming growth factor beta 2
hsa-miR-132-3p	DNAJB14	DnaJ heat shock protein family (Hsp40) member B14

hsa-miR-132-3p	AAK1	AP2 associated kinase 1
hsa-miR-132-3p	FXR1	FMR1 autosomal homolog 1
hsa-miR-132-3p	TUT7	terminal uridylyl transferase 7
hsa-miR-132-3p	CDK14	cyclin dependent kinase 14
hsa-miR-132-3p	PFN2	profilin 2
hsa-miR-132-3p	ZSWIM6	zinc finger SWIM-type containing 6
hsa-miR-132-3p	ACAD9	acyl-CoA dehydrogenase family member 9
hsa-miR-132-3p	PDE3A	phosphodiesterase 3A
hsa-miR-132-3p	GAPVD1	GTPase activating protein and VPS9 domains 1
hsa-miR-132-3p	YWHAG	tyrosine 3-monooxygenase/tryptophan 5-monooxygenase activation protein gamma
hsa-miR-132-3p	FRMD6	FERM domain containing 6
hsa-miR-132-3p	BLCAP	BLCAP, apoptosis inducing factor
hsa-miR-132-3p	ABHD13	abhydrolase domain containing 13
hsa-miR-132-3p	RND3	Rho family GTPase 3
hsa-miR-132-3p	MTFR2	mitochondrial fission regulator 2
hsa-miR-132-3p	LRRC39	leucine rich repeat containing 39
hsa-miR-132-3p	ZNF362	zinc finger protein 362
hsa-miR-132-3p	KDM7A	lysine demethylase 7A
hsa-miR-132-3p	C1QL1	complement C1q like 1
hsa-miR-132-3p	PANX1	pannexin 1
hsa-miR-132-3p	BMPR1A	bone morphogenetic protein receptor type 1A
hsa-miR-132-3p	RBMS1	RNA binding motif single stranded interacting protein 1
hsa-miR-132-3p	EIF2S3	eukaryotic translation initiation factor 2 subunit gamma
hsa-miR-132-3p	MAGI3	membrane associated guanylate kinase, WW and PDZ domain containing 3
hsa-miR-132-3p	PLCG2	phospholipase C gamma 2
hsa-miR-132-3p	SNURF	SNRPN upstream reading frame
hsa-miR-132-3p	DYRK4	dual specificity tyrosine phosphorylation regulated kinase 4
hsa-miR-132-3p	NAP1L1	nucleosome assembly protein 1 like 1
hsa-miR-132-3p	RPL37	ribosomal protein L37
hsa-miR-132-3p	RASSF3	Ras association domain family member 3
hsa-miR-132-3p	COA5	cytochrome c oxidase assembly factor 5
hsa-miR-132-3p	CDRT1	CMT1A duplicated region transcript 1
hsa-miR-132-3p	RAP2B	RAP2B, member of RAS oncogene family
hsa-miR-132-3p	FOXP2	forkhead box P2
hsa-miR-132-3p	SLC25A20	solute carrier family 25 member 20
hsa-miR-132-3p	LY75	lymphocyte antigen 75
hsa-miR-132-3p	RAD50	RAD50 double strand break repair protein
hsa-miR-132-3p	MPEG1	macrophage expressed 1
hsa-miR-132-3p	TMEM68	transmembrane protein 68
hsa-miR-132-3p	MAPK3	mitogen-activated protein kinase 3
hsa-miR-132-3p	SRGAP3	SLIT-ROBO Rho GTPase activating protein 3
hsa-miR-132-3p	MLLT3	MLLT3, super elongation complex subunit
hsa-miR-132-3p	PNISR	PNN interacting serine and arginine rich protein

hsa-miR-132-3p	EDIL3	EGF like repeats and discoidin domains 3
hsa-miR-132-3p	PIGG	phosphatidylinositol glycan anchor biosynthesis class G
hsa-miR-132-3p	ACTR2	ARP2 actin related protein 2 homolog
hsa-miR-132-3p	COMMD10	COMM domain containing 10
hsa-miR-132-3p	ZNF695	zinc finger protein 695
hsa-miR-132-3p	TBCA	tubulin folding cofactor A
hsa-miR-132-3p	OTUD3	OTU deubiquitinase 3
hsa-miR-132-3p	MRS2	magnesium transporter MRS2
hsa-miR-132-3p	PRR15L	proline rich 15 like
hsa-miR-132-3p	NIPSNAP2	nipsnap homolog 2
hsa-miR-132-3p	PHIP	pleckstrin homology domain interacting protein
hsa-miR-132-3p	ABCG4	ATP binding cassette subfamily G member 4
hsa-miR-132-3p	C8A	complement C8 alpha chain
hsa-miR-132-3p	ZNF704	zinc finger protein 704
hsa-miR-132-3p	QKI	QKI, KH domain containing RNA binding
hsa-miR-132-3p	RLIM	ring finger protein, LIM domain interacting
hsa-miR-132-3p	DNAJC9	DnaJ heat shock protein family (Hsp40) member C9
hsa-miR-132-3p	SETMAR	SET domain and mariner transposase fusion gene
hsa-miR-132-3p	CUL4B	cullin 4B
hsa-miR-132-3p	ZBTB17	zinc finger and BTB domain containing 17
hsa-miR-132-3p	MSH2	mutS homolog 2
hsa-miR-132-3p	CFL2	cofilin 2
hsa-miR-132-3p	UGT2A3	UDP glucuronosyltransferase family 2 member A3
hsa-miR-132-3p	GABRA4	gamma-aminobutyric acid type A receptor alpha4 subunit
hsa-miR-132-3p	HIPK3	homeodomain interacting protein kinase 3
hsa-miR-132-3p	SGCZ	sarcoglycan zeta
hsa-miR-132-3p	GRIA2	glutamate ionotropic receptor AMPA type subunit 2
hsa-miR-132-3p	INSM2	INSM transcriptional repressor 2
hsa-miR-132-3p	CEMIP2	cell migration inducing hyaluronidase 2
hsa-miR-132-3p	DGKH	diacylglycerol kinase eta
hsa-miR-132-3p	CYP20A1	cytochrome P450 family 20 subfamily A member 1
hsa-miR-132-3p	NR2C2	nuclear receptor subfamily 2 group C member 2
hsa-miR-132-3p	POU3F3	POU class 3 homeobox 3
hsa-miR-132-3p	MCTS1	MCTS1, re-initiation and release factor
hsa-miR-132-3p	STON2	stonin 2
hsa-miR-132-3p	ARHGAP21	Rho GTPase activating protein 21
hsa-miR-132-3p	CEPT1	choline/ethanolamine phosphotransferase 1
hsa-miR-132-3p	SHANK2	SH3 and multiple ankyrin repeat domains 2
hsa-miR-132-3p	ELAVL4	ELAV like RNA binding protein 4
hsa-miR-132-3p	GPR89A	G protein-coupled receptor 89A
hsa-miR-132-3p	PRUNE1	prune exopolyphosphatase 1
hsa-miR-132-3p	TMEM51	transmembrane protein 51
hsa-miR-132-3p	EML4	EMAP like 4
hsa-miR-132-3p	EMCN	endomucin

hsa-miR-132-3p	NDRG4	NDRG family member 4
hsa-miR-132-3p	NAXE	NAD(P)HX epimerase
hsa-miR-132-3p	NET1	neuroepithelial cell transforming 1
hsa-miR-132-3p	SLC23A2	solute carrier family 23 member 2
hsa-miR-132-3p	MOCS3	molybdenum cofactor synthesis 3
hsa-miR-132-3p	L3HYPDH	trans-L-3-hydroxyproline dehydratase
hsa-miR-132-3p	SRGAP2	SLIT-ROBO Rho GTPase activating protein 2
hsa-miR-132-3p	DCBLD2	discoidin, CUB and LCCL domain containing 2
hsa-miR-132-3p	ZDHHC15	zinc finger DHHC-type containing 15
hsa-miR-132-3p	GPR89B	G protein-coupled receptor 89B
hsa-miR-132-3p	PRICKLE2	prickle planar cell polarity protein 2
hsa-miR-132-3p	ZDHHC23	zinc finger DHHC-type containing 23
hsa-miR-132-3p	TRMT13	tRNA methyltransferase 13 homolog
hsa-miR-132-3p	SPTLC2	serine palmitoyltransferase long chain base subunit 2
hsa-miR-132-3p	LOC102723360	uncharacterized LOC102723360
hsa-miR-132-3p	ATF2	activating transcription factor 2
hsa-miR-132-3p	TAF4	TATA-box binding protein associated factor 4
hsa-miR-132-3p	ORC4	origin recognition complex subunit 4
hsa-miR-132-3p	LOC102724219	uncharacterized LOC102724219
hsa-miR-132-3p	SPAST	spastin
hsa-miR-132-3p	UNC13C	unc-13 homolog C
hsa-miR-132-3p	BTBD7	BTB domain containing 7
hsa-miR-132-3p	CHP1	calcineurin like EF-hand protein 1
hsa-miR-132-3p	CDC40	cell division cycle 40
hsa-miR-132-3p	DDX47	DEAD-box helicase 47
hsa-miR-132-3p	C11orf87	chromosome 11 open reading frame 87
hsa-miR-132-3p	LOC102724951	uncharacterized LOC102724951
hsa-miR-132-3p	LHX9	LIM homeobox 9
hsa-miR-132-3p	SHOX	short stature homeobox
hsa-miR-132-3p	LOC102724843	uncharacterized LOC102724843
hsa-miR-132-3p	LARP4	La ribonucleoprotein domain family member 4
hsa-miR-132-3p	PNPLA3	patatin like phospholipase domain containing 3
hsa-miR-132-3p	AADAC	arylacetamide deacetylase
hsa-miR-132-3p	ADAMTS6	ADAM metallopeptidase with thrombospondin type 1 motif 6
hsa-miR-132-3p	TENT4B	terminal nucleotidyltransferase 4B
hsa-miR-132-3p	CDON	cell adhesion associated, oncogene regulated
hsa-miR-132-3p	ZBTB34	zinc finger and BTB domain containing 34
hsa-miR-132-3p	ZFX	zinc finger protein X-linked
hsa-miR-132-3p	COLQ	collagen like tail subunit of asymmetric acetylcholinesterase
hsa-miR-132-3p	FBXO42	F-box protein 42
hsa-miR-132-3p	LMLN	leishmanolysin like peptidase
hsa-miR-132-3p	GABPB1	GA binding protein transcription factor subunit beta 1
hsa-miR-132-3p	SLC12A6	solute carrier family 12 member 6

hsa-miR-132-3p	MSANTD3-TMEFF1	MSANTD3-TMEFF1 readthrough
hsa-miR-132-3p	SEC14L5	SEC14 like lipid binding 5
hsa-miR-132-3p	SUDS3	SDS3 homolog, SIN3A corepressor complex component
hsa-miR-132-3p	ETFBKMT	electron transfer flavoprotein subunit beta lysine methyltransferase
hsa-miR-132-3p	CPSF6	cleavage and polyadenylation specific factor 6
hsa-miR-132-3p	CAPSL	calcyphosine like
hsa-miR-132-3p	SLC6A3	solute carrier family 6 member 3
hsa-miR-132-3p	FAM19A2	family with sequence similarity 19 member A2, C-C motif chemokine like
hsa-miR-132-3p	FAM71F2	family with sequence similarity 71 member F2
hsa-miR-132-3p	CLOCK	clock circadian regulator
hsa-miR-132-3p	TMEM215	transmembrane protein 215
hsa-miR-132-3p	ZNF264	zinc finger protein 264
hsa-miR-132-3p	DGKE	diacylglycerol kinase epsilon
hsa-miR-132-3p	REL	REL proto-oncogene, NF-kB subunit
hsa-miR-132-3p	SV2B	synaptic vesicle glycoprotein 2B
hsa-miR-132-3p	MSTN	myostatin
hsa-miR-132-3p	FBXW2	F-box and WD repeat domain containing 2
hsa-miR-132-3p	FOXN3	forkhead box N3
hsa-miR-132-3p	PRKD1	protein kinase D1
hsa-miR-132-3p	GNL3L	G protein nucleolar 3 like
hsa-miR-132-3p	WASHC5	WASH complex subunit 5
hsa-miR-132-3p	SETDB2	SET domain bifurcated histone lysine methyltransferase 2
hsa-miR-132-3p	CAMTA1	calmodulin binding transcription activator 1
hsa-miR-132-3p	TP53AIP1	tumor protein p53 regulated apoptosis inducing protein 1
hsa-miR-132-3p	RHOU	ras homolog family member U
hsa-miR-132-3p	ZNF333	zinc finger protein 333
hsa-miR-132-3p	WDR93	WD repeat domain 93
hsa-miR-132-3p	ARMC1	armadillo repeat containing 1
hsa-miR-132-3p	TMEM50B	transmembrane protein 50B
hsa-miR-132-3p	TMEM87A	transmembrane protein 87A
hsa-miR-132-3p	GRK3	G protein-coupled receptor kinase 3
hsa-miR-132-3p	DNMBP	dynamin binding protein
hsa-miR-132-3p	DIPK2B	divergent protein kinase domain 2B
hsa-miR-132-3p	LOC100287896	uncharacterized LOC100287896
hsa-miR-132-3p	PPM1L	protein phosphatase, Mg2+/Mn2+ dependent 1L
hsa-miR-132-3p	KCNMA1	potassium calcium-activated channel subfamily M alpha 1
hsa-miR-132-3p	SCCPDH	saccharopine dehydrogenase (putative)
hsa-miR-132-3p	SNIP1	Smad nuclear interacting protein 1
hsa-miR-132-3p	NEIL2	nei like DNA glycosylase 2
hsa-miR-132-3p	NAB1	NGFI-A binding protein 1

hsa-miR-132-3p	BTRC	beta-transducin repeat containing E3 ubiquitin protein ligase
hsa-miR-132-3p	CC2D2A	coiled-coil and C2 domain containing 2A
hsa-miR-132-3p	AFF4	AF4/FMR2 family member 4
hsa-miR-132-3p	GRSF1	G-rich RNA sequence binding factor 1
hsa-miR-132-3p	EDN1	endothelin 1
hsa-miR-132-3p	WTAP	WT1 associated protein
hsa-miR-132-3p	KLRG1	killer cell lectin like receptor G1
hsa-miR-132-3p	RIT1	Ras like without CAAX 1
hsa-miR-132-3p	KCNS1	potassium voltage-gated channel modifier subfamily S member 1
hsa-miR-132-3p	POLR1A	RNA polymerase I subunit A
hsa-miR-132-3p	NUCKS1	nuclear casein kinase and cyclin dependent kinase substrate 1
hsa-miR-132-3p	ZNF90	zinc finger protein 90
hsa-miR-132-3p	ELOVL2	ELOVL fatty acid elongase 2
hsa-miR-132-3p	PIK3IP1	phosphoinositide-3-kinase interacting protein 1
hsa-miR-132-3p	ACHE	acetylcholinesterase (Cartwright blood group)
hsa-miR-132-3p	PTPN4	protein tyrosine phosphatase, non-receptor type 4
hsa-miR-132-3p	ESYT2	extended synaptotagmin 2
hsa-miR-132-3p	TSHZ2	teashirt zinc finger homeobox 2
hsa-miR-132-3p	C19orf47	chromosome 19 open reading frame 47
hsa-miR-132-3p	EXOC2	exocyst complex component 2
hsa-miR-132-3p	PRR5L	proline rich 5 like
hsa-miR-132-3p	CLVS2	clavesin 2
hsa-miR-132-3p	TRIB2	tribbles pseudokinase 2
hsa-miR-132-3p	SLC10A4	solute carrier family 10 member 4
hsa-miR-132-3p	PNN	pinin, desmosome associated protein
hsa-miR-132-3p	MEF2C	myocyte enhancer factor 2C
hsa-miR-132-3p	sept-11	septin 11
hsa-miR-132-3p	CST3	cystatin C
hsa-miR-132-3p	MAPK8	mitogen-activated protein kinase 8
hsa-miR-132-3p	WWOX	WW domain containing oxidoreductase
hsa-miR-132-3p	CSNK1G3	casein kinase 1 gamma 3
hsa-miR-132-3p	BNIP2	BCL2 interacting protein 2
hsa-miR-132-3p	ARID1A	AT-rich interaction domain 1A
hsa-miR-132-3p	ZNF287	zinc finger protein 287
hsa-miR-132-3p	C5orf51	chromosome 5 open reading frame 51
hsa-miR-132-3p	RRP7A	ribosomal RNA processing 7 homolog A
hsa-miR-132-3p	TPGS2	tubulin polyglutamylase complex subunit 2
hsa-miR-132-3p	CRTC1	CREB regulated transcription coactivator 1
hsa-miR-132-3p	ZNF280C	zinc finger protein 280C
hsa-miR-132-3p	CDK6	cyclin dependent kinase 6
hsa-miR-132-3p	TADA2B	transcriptional adaptor 2B
hsa-miR-132-3p	ATP6V1H	ATPase H+ transporting V1 subunit H
hsa-miR-132-3p	DOCK4	dedicator of cytokinesis 4

hsa-miR-132-3p	KMT5B	lysine methyltransferase 5B
hsa-miR-132-3p	VWA3B	von Willebrand factor A domain containing 3B
hsa-miR-132-3p	TTC14	tetratricopeptide repeat domain 14
hsa-miR-132-3p	MARCH6	membrane associated ring-CH-type finger 6
hsa-miR-132-3p	ZFY	zinc finger protein Y-linked
hsa-miR-132-3p	TMEM59	transmembrane protein 59
hsa-miR-132-3p	TCF7L1	transcription factor 7 like 1
hsa-miR-132-3p	PHKB	phosphorylase kinase regulatory subunit beta
hsa-miR-132-3p	DCAF10	DDB1 and CUL4 associated factor 10
hsa-miR-132-3p	CCDC189	coiled-coil domain containing 189
hsa-miR-132-3p	ALKBH8	alkB homolog 8, tRNA methyltransferase
hsa-miR-132-3p	ZBTB5	zinc finger and BTB domain containing 5
hsa-miR-132-3p	MEGF11	multiple EGF like domains 11
hsa-miR-132-3p	SEPSECS	Sep (O-phosphoserine) tRNA:Sec (selenocysteine) tRNA synthase
hsa-miR-132-3p	ABR	ABR, RhoGEF and GTPase activating protein
hsa-miR-132-3p	SREBF1	sterol regulatory element binding transcription factor 1
hsa-miR-132-3p	SEMA4A	semaphorin 4A
hsa-miR-132-3p	GRM4	glutamate metabotropic receptor 4
hsa-miR-132-3p	ZWINT	ZW10 interacting kinetochore protein
hsa-miR-132-3p	REPS2	RALBP1 associated Eps domain containing 2
hsa-miR-132-3p	ZNF248	zinc finger protein 248
hsa-miR-132-3p	UNC80	unc-80 homolog, NALCN channel complex subunit
hsa-miR-132-3p	ITCH	itchy E3 ubiquitin protein ligase
hsa-miR-132-3p	ZMAT3	zinc finger matrin-type 3
hsa-miR-132-3p	SLC30A10	solute carrier family 30 member 10
hsa-miR-132-3p	DIMT1	DIM1 dimethyladenosine transferase 1 homolog
hsa-miR-132-3p	UBR3	ubiquitin protein ligase E3 component n-recognin 3
hsa-miR-132-3p	ACP1	acid phosphatase 1
hsa-miR-132-3p	ZNF696	zinc finger protein 696
hsa-miR-132-3p	ENTPD1	ectonucleoside triphosphate diphosphohydrolase 1
hsa-miR-132-3p	FABP2	fatty acid binding protein 2
hsa-miR-132-3p	BRAP	BRCA1 associated protein
hsa-miR-132-3p	TFDP1	transcription factor Dp-1
hsa-miR-132-3p	ABCG1	ATP binding cassette subfamily G member 1
hsa-miR-132-3p	EDNRA	endothelin receptor type A
hsa-miR-132-3p	TPRG1	tumor protein p63 regulated 1
hsa-miR-132-3p	MDFIC	MyoD family inhibitor domain containing
hsa-miR-132-3p	PSD3	pleckstrin and Sec7 domain containing 3
hsa-miR-132-3p	HECTD2	HECT domain E3 ubiquitin protein ligase 2
hsa-miR-132-3p	SH3TC2	SH3 domain and tetratricopeptide repeats 2
hsa-miR-132-3p	PLXND1	plexin D1
hsa-miR-132-3p	NABP1	nucleic acid binding protein 1
hsa-miR-132-3p	GUCY1A1	guanylate cyclase 1 soluble subunit alpha 1
hsa-miR-132-3p	KLRC4-KLRK1	KLRC4-KLRK1 readthrough

hsa-miR-132-3p	ZNF280D	zinc finger protein 280D
hsa-miR-132-3p	TRIP12	thyroid hormone receptor interactor 12
hsa-miR-132-3p	APC	APC, WNT signaling pathway regulator
hsa-miR-132-3p	KLRK1	killer cell lectin like receptor K1
hsa-miR-132-3p	SUCNR1	succinate receptor 1
hsa-miR-132-3p	NOM1	nucleolar protein with MIF4G domain 1
hsa-miR-132-3p	FRK	fyn related Src family tyrosine kinase
hsa-miR-132-3p	FZD1	frizzled class receptor 1
hsa-miR-132-3p	NKTR	natural killer cell triggering receptor
hsa-miR-132-3p	CASD1	CAS1 domain containing 1
hsa-miR-132-3p	CFAP206	cilia and flagella associated protein 206
hsa-miR-132-3p	USP44	ubiquitin specific peptidase 44
hsa-miR-132-3p	VPS41	VPS41, HOPS complex subunit
hsa-miR-132-3p	EHF	ETS homologous factor
hsa-miR-132-3p	CNOT2	CCR4-NOT transcription complex subunit 2
hsa-miR-132-3p	GLIPR1	GLI pathogenesis related 1
hsa-miR-132-3p	DACH1	dachshund family transcription factor 1
hsa-miR-200b-3p	VASH2	vasohibin 2
hsa-miR-200b-3p	HIPK3	homeodomain interacting protein kinase 3
hsa-miR-200b-3p	MAP2	microtubule associated protein 2
hsa-miR-200b-3p	ERRFI1	ERBB receptor feedback inhibitor 1
hsa-miR-200b-3p	ZEB1	zinc finger E-box binding homeobox 1
hsa-miR-200b-3p	NR5A2	nuclear receptor subfamily 5 group A member 2
hsa-miR-200b-3p	ZEB2	zinc finger E-box binding homeobox 2
hsa-miR-200b-3p	RECK	reversion inducing cysteine rich protein with kazal motifs
hsa-miR-200b-3p	SLIT2	slit guidance ligand 2
hsa-miR-200b-3p	WIPF1	WAS/WASL interacting protein family member 1
hsa-miR-200b-3p	C11orf95	chromosome 11 open reading frame 95
hsa-miR-200b-3p	FAM8A1	family with sequence similarity 8 member A1
hsa-miR-200b-3p	LHFPL6	LHFPL tetraspan subfamily member 6
hsa-miR-200b-3p	AP1S2	adaptor related protein complex 1 subunit sigma 2
hsa-miR-200b-3p	SEC23A	Sec23 homolog A, coat complex II component
hsa-miR-200b-3p	TBX18	T-box 18
hsa-miR-200b-3p	PTPN21	protein tyrosine phosphatase, non-receptor type 21
hsa-miR-200b-3p	DNAJC3	DnaJ heat shock protein family (Hsp40) member C3
hsa-miR-200b-3p	QKI	QKI, KH domain containing RNA binding
hsa-miR-200b-3p	RIC1	RIC1 homolog, RAB6A GEF complex partner 1
hsa-miR-200b-3p	CCNJ	cyclin J
hsa-miR-200b-3p	PCMTD1	protein-L-isoaspartate (D-aspartate) O-methyltransferase domain containing 1
hsa-miR-200b-3p	CFL2	cofilin 2
hsa-miR-200b-3p	GPM6A	glycoprotein M6A
hsa-miR-200b-3p	ARHGAP6	Rho GTPase activating protein 6
hsa-miR-200b-3p	TFAP2A	transcription factor AP-2 alpha
hsa-miR-200b-3p	SLC35B4	solute carrier family 35 member B4

hsa-miR-200b-3p	BAP1	BRCA1 associated protein 1
hsa-miR-200b-3p	RAB11FIP2	RAB11 family interacting protein 2
hsa-miR-200b-3p	MSN	moesin
hsa-miR-200b-3p	FBXW7	F-box and WD repeat domain containing 7
hsa-miR-200b-3p	TRIM33	tripartite motif containing 33
hsa-miR-200b-3p	CNOT6	CCR4-NOT transcription complex subunit 6
hsa-miR-200b-3p	PRTG	protogenin
hsa-miR-200b-3p	RPS6KB1	ribosomal protein S6 kinase B1
hsa-miR-200b-3p	WASF3	WAS protein family member 3
hsa-miR-200b-3p	KDM7A	lysine demethylase 7A
hsa-miR-200b-3p	ELL2	elongation factor for RNA polymerase II 2
hsa-miR-200b-3p	ATXN1	ataxin 1
hsa-miR-200b-3p	SYNJ1	synaptojanin 1
hsa-miR-200b-3p	MIEF1	mitochondrial elongation factor 1
hsa-miR-200b-3p	MBNL3	muscleblind like splicing regulator 3
hsa-miR-200b-3p	CSNK1G3	casein kinase 1 gamma 3
hsa-miR-200b-3p	KHDRBS1	KH RNA binding domain containing, signal transduction associated 1
hsa-miR-200b-3p	ELMOD2	ELMO domain containing 2
hsa-miR-200b-3p	MCFD2	multiple coagulation factor deficiency 2
hsa-miR-200b-3p	CRKL	CRK like proto-oncogene, adaptor protein
hsa-miR-200b-3p	TENT4B	terminal nucleotidyltransferase 4B
hsa-miR-200b-3p	MPRIP	myosin phosphatase Rho interacting protein
hsa-miR-200b-3p	PPP4R2	protein phosphatase 4 regulatory subunit 2
hsa-miR-200b-3p	MGAT2	mannosyl (alpha-1,6-)-glycoprotein beta-1,2-N-acetylglucosaminyltransferase
hsa-miR-200b-3p	FAM122C	family with sequence similarity 122C
hsa-miR-200b-3p	NFIA	nuclear factor I A
hsa-miR-200b-3p	NOVA2	NOVA alternative splicing regulator 2
hsa-miR-200b-3p	SUSD5	sushi domain containing 5
hsa-miR-200b-3p	FEZ2	fasciculation and elongation protein zeta 2
hsa-miR-200b-3p	PRDM16	PR/SET domain 16
hsa-miR-200b-3p	LRP1B	LDL receptor related protein 1B
hsa-miR-200b-3p	PPP2R5E	protein phosphatase 2 regulatory subunit B'epsilon
hsa-miR-200b-3p	PTPN14	protein tyrosine phosphatase, non-receptor type 14
hsa-miR-200b-3p	OSTM1	osteoclastogenesis associated transmembrane protein 1
hsa-miR-200b-3p	FAM126B	family with sequence similarity 126 member B
hsa-miR-200b-3p	CDK17	cyclin dependent kinase 17
hsa-miR-200b-3p	FRMD6	FERM domain containing 6
hsa-miR-200b-3p	RBSN	rabenosyn, RAB effector
hsa-miR-200b-3p	JAKMIP2	janus kinase and microtubule interacting protein 2
hsa-miR-200b-3p	PIK3CA	phosphatidylinositol-4,5-bisphosphate 3-kinase catalytic subunit alpha
hsa-miR-200b-3p	CCDC177	coiled-coil domain containing 177
hsa-miR-200b-3p	ARIH1	ariadne RBR E3 ubiquitin protein ligase 1
hsa-miR-200b-3p	ATP11C	ATPase phospholipid transporting 11C

hsa-miR-200b-3p	RTF1	RTF1 homolog, Paf1/RNA polymerase II complex component
hsa-miR-200b-3p	PPM1F	protein phosphatase, Mg2+/Mn2+ dependent 1F
hsa-miR-200b-3p	RAP2C	RAP2C, member of RAS oncogene family
hsa-miR-200b-3p	MMD	monocyte to macrophage differentiation associated
hsa-miR-200b-3p	CHN2	chimerin 2
hsa-miR-200b-3p	PHF21B	PHD finger protein 21B
hsa-miR-200b-3p	SULF1	sulfatase 1
hsa-miR-200b-3p	ARL2BP	ADP ribosylation factor like GTPase 2 binding protein
hsa-miR-200b-3p	ADIPOR2	adiponectin receptor 2
hsa-miR-200b-3p	ZNF532	zinc finger protein 532
hsa-miR-200b-3p	PHACTR3	phosphatase and actin regulator 3
hsa-miR-200b-3p	RASA2	RAS p21 protein activator 2
hsa-miR-200b-3p	ZNF711	zinc finger protein 711
hsa-miR-200b-3p	SEMA6D	semaphorin 6D
hsa-miR-200b-3p	VLDLR	very low density lipoprotein receptor
hsa-miR-200b-3p	IGSF10	immunoglobulin superfamily member 10
hsa-miR-200b-3p	WNT16	Wnt family member 16
hsa-miR-200b-3p	CBL	Cbl proto-oncogene
hsa-miR-200b-3p	EPS8	epidermal growth factor receptor pathway substrate 8
hsa-miR-200b-3p	NANOS1	nanos C2HC-type zinc finger 1
hsa-miR-200b-3p	TMOD3	tropomodulin 3
hsa-miR-200b-3p	SGIP1	SH3 domain GRB2 like endophilin interacting protein 1
hsa-miR-200b-3p	COL4A3BP	collagen type IV alpha 3 binding protein
hsa-miR-200b-3p	KDELC1	KDEL motif containing 1
hsa-miR-200b-3p	MINDY2	MINDY lysine 48 deubiquitinase 2
hsa-miR-200b-3p	RND3	Rho family GTPase 3
hsa-miR-200b-3p	KDR	kinase insert domain receptor
hsa-miR-200b-3p	OTUD4	OTU deubiquitinase 4
hsa-miR-200b-3p	NCOA2	nuclear receptor coactivator 2
hsa-miR-200b-3p	HMBOX1	homeobox containing 1
hsa-miR-200b-3p	DUSP1	dual specificity phosphatase 1
hsa-miR-200b-3p	ELOC	elongin C
hsa-miR-200b-3p	C16orf72	chromosome 16 open reading frame 72
hsa-miR-200b-3p	EGLN1	egl-9 family hypoxia inducible factor 1
hsa-miR-200b-3p	CLASP1	cytoplasmic linker associated protein 1
hsa-miR-200b-3p	ZFAND6	zinc finger AN1-type containing 6
hsa-miR-200b-3p	FOXG1	forkhead box G1
hsa-miR-200b-3p	KCNQ3	potassium voltage-gated channel subfamily Q member 3
hsa-miR-200b-3p	MBOAT2	membrane bound O-acyltransferase domain containing 2
hsa-miR-200b-3p	PHTF2	putative homeodomain transcription factor 2
hsa-miR-200b-3p	BRWD3	bromodomain and WD repeat domain containing 3
hsa-miR-200b-3p	NBR1	NBR1, autophagy cargo receptor
hsa-miR-200b-3p	ZNF131	zinc finger protein 131
hsa-miR-200b-3p	AFF3	AF4/FMR2 family member 3

hsa-miR-200b-3p	ARL5A	ADP ribosylation factor like GTPase 5A
hsa-miR-200b-3p	TMX4	thioredoxin related transmembrane protein 4
hsa-miR-200b-3p	IMMP2L	inner mitochondrial membrane peptidase subunit 2
hsa-miR-200b-3p	MAP3K1	mitogen-activated protein kinase kinase kinase 1
hsa-miR-200b-3p	IPO7	importin 7
hsa-miR-200b-3p	GOLGA7	golgin A7
hsa-miR-200b-3p	DLC1	DLC1 Rho GTPase activating protein
hsa-miR-200b-3p	XKR8	XK related 8
hsa-miR-200b-3p	ELK4	ELK4, ETS transcription factor
hsa-miR-200b-3p	FN1	fibronectin 1
hsa-miR-200b-3p	SESN1	sestrin 1
hsa-miR-200b-3p	JUN	Jun proto-oncogene, AP-1 transcription factor subunit
hsa-miR-200b-3p	GPR158	G protein-coupled receptor 158
hsa-miR-200b-3p	DGKH	diacylglycerol kinase eta
hsa-miR-200b-3p	SYDE1	synapse defective Rho GTPase homolog 1
hsa-miR-200b-3p	PPHLN1	periphilin 1
hsa-miR-200b-3p	ZC3H6	zinc finger CCCH-type containing 6
hsa-miR-200b-3p	PRKG1	protein kinase cGMP-dependent 1
hsa-miR-200b-3p	SLC1A2	solute carrier family 1 member 2
hsa-miR-200b-3p	RANBP9	RAN binding protein 9
hsa-miR-200b-3p	GOLGA1	golgin A1
hsa-miR-200b-3p	LOX	lysyl oxidase
hsa-miR-200b-3p	GIT2	GIT ArfGAP 2
hsa-miR-200b-3p	ARMCX5-GPRASP2	ARMCX5-GPRASP2 readthrough
hsa-miR-200b-3p	MPDZ	multiple PDZ domain crumbs cell polarity complex component
hsa-miR-200b-3p	PGM2L1	phosphoglucomutase 2 like 1
hsa-miR-200b-3p	PRKACB	protein kinase cAMP-activated catalytic subunit beta
hsa-miR-200b-3p	CDH20	cadherin 20
hsa-miR-200b-3p	NTF3	neurotrophin 3
hsa-miR-200b-3p	B3GNT2	UDP-GlcNAc:betaGal beta-1,3-N-acetylglucosaminyltransferase 2
hsa-miR-200b-3p	PSAT1	phosphoserine aminotransferase 1
hsa-miR-200b-3p	REEP1	receptor accessory protein 1
hsa-miR-200b-3p	MED13	mediator complex subunit 13
hsa-miR-200b-3p	TBK1	TANK binding kinase 1
hsa-miR-200b-3p	TBC1D12	TBC1 domain family member 12
hsa-miR-200b-3p	MAPK7	mitogen-activated protein kinase 7
hsa-miR-200b-3p	WAPL	WAPL cohesin release factor
hsa-miR-200b-3p	VEGFA	vascular endothelial growth factor A
hsa-miR-200b-3p	RUSC2	RUN and SH3 domain containing 2
hsa-miR-200b-3p	SFXN1	sideroflexin 1
hsa-miR-200b-3p	ZFX	zinc finger protein X-linked
hsa-miR-200b-3p	HS2ST1	heparan sulfate 2-O-sulfotransferase 1

hsa-miR-200b-3p	BLCAP	BLCAP, apoptosis inducing factor
hsa-miR-200b-3p	RAB21	RAB21, member RAS oncogene family
hsa-miR-200b-3p	GPRASP2	G protein-coupled receptor associated sorting protein 2
hsa-miR-200b-3p	DESI1	desumoylating isopeptidase 1
hsa-miR-200b-3p	FOXF1	forkhead box F1
hsa-miR-200b-3p	PTPRZ1	protein tyrosine phosphatase, receptor type Z1
hsa-miR-200b-3p	TMEM17	transmembrane protein 17
hsa-miR-200b-3p	FSCN1	fascin actin-bundling protein 1
hsa-miR-200b-3p	PPP1R18	protein phosphatase 1 regulatory subunit 18
hsa-miR-200b-3p	RNF2	ring finger protein 2
hsa-miR-200b-3p	THAP1	THAP domain containing 1
hsa-miR-200b-3p	CLIC4	chloride intracellular channel 4
hsa-miR-200b-3p	CECR2	CECR2, histone acetyl-lysine reader
hsa-miR-200b-3p	HOOK1	hook microtubule tethering protein 1
hsa-miR-200b-3p	PPM1E	protein phosphatase, Mg2+/Mn2+ dependent 1E
hsa-miR-200b-3p	CHRDL1	chordin like 1
hsa-miR-200b-3p	DTNA	dystrobrevin alpha
hsa-miR-200b-3p	ZBTB10	zinc finger and BTB domain containing 10
hsa-miR-200b-3p	B3GLCT	beta 3-glucosyltransferase
hsa-miR-200b-3p	DNAJB9	DnaJ heat shock protein family (Hsp40) member B9
hsa-miR-200b-3p	FAT3	FAT atypical cadherin 3
hsa-miR-200b-3p	NR3C1	nuclear receptor subfamily 3 group C member 1
hsa-miR-200b-3p	FAM227B	family with sequence similarity 227 member B
hsa-miR-200b-3p	CSMD3	CUB and Sushi multiple domains 3
hsa-miR-200b-3p	PPFIA1	PTPRF interacting protein alpha 1
hsa-miR-200b-3p	KCTD8	potassium channel tetramerization domain containing 8
hsa-miR-200b-3p	PI4K2B	phosphatidylinositol 4-kinase type 2 beta
hsa-miR-200b-3p	ANKRD40	ankyrin repeat domain 40
hsa-miR-200b-3p	VASH1	vasohibin 1
hsa-miR-200b-3p	PDS5B	PDS5 cohesin associated factor B
hsa-miR-200b-3p	SLK	STE20 like kinase
hsa-miR-200b-3p	RTKN2	rhotekin 2
hsa-miR-200b-3p	RNF19A	ring finger protein 19A, RBR E3 ubiquitin protein ligase
hsa-miR-200b-3p	FHL1	four and a half LIM domains 1
hsa-miR-200b-3p	SPAG9	sperm associated antigen 9
hsa-miR-200b-3p	DPY19L1	dpy-19 like C-mannosyltransferase 1
hsa-miR-200b-3p	AMFR	autocrine motility factor receptor
hsa-miR-200b-3p	ELAVL2	ELAV like RNA binding protein 2
hsa-miR-200b-3p	LBR	lamin B receptor
hsa-miR-200b-3p	ZYG11B	zyg-11 family member B, cell cycle regulator
hsa-miR-200b-3p	CLIP1	CAP-Gly domain containing linker protein 1
hsa-miR-200b-3p	MOSMO	modulator of smoothened
hsa-miR-200b-3p	WDR82	WD repeat domain 82
hsa-miR-200b-3p	SERPINI1	serpin family I member 1
hsa-miR-200b-3p	RASSF8	Ras association domain family member 8

hsa-miR-200b-3p	JAZF1	JAZF zinc finger 1
hsa-miR-200b-3p	TOB1	transducer of ERBB2, 1
hsa-miR-200b-3p	EVI5	ecotropic viral integration site 5
hsa-miR-200b-3p	B4GAT1	beta-1,4-glucuronyltransferase 1
hsa-miR-200b-3p	POLK	DNA polymerase kappa
hsa-miR-200b-3p	CHRM2	cholinergic receptor muscarinic 2
hsa-miR-200b-3p	TRAPPC8	trafficking protein particle complex 8
hsa-miR-200b-3p	PUM2	pumilio RNA binding family member 2
hsa-miR-200b-3p	CDYL	chromodomain Y like
hsa-miR-200b-3p	YWHAG	tyrosine 3-monooxygenase/tryptophan 5-monooxygenase activation protein gamma
hsa-miR-200b-3p	SLC6A11	solute carrier family 6 member 11
hsa-miR-200b-3p	TCAIM	T cell activation inhibitor, mitochondrial
hsa-miR-200b-3p	PKD1	polycystin 1, transient receptor potential channel interacting
hsa-miR-200b-3p	FBXO30	F-box protein 30
hsa-miR-200b-3p	DNMT3B	DNA methyltransferase 3 beta
hsa-miR-200b-3p	FBXO33	F-box protein 33
hsa-miR-200b-3p	ITGA1	integrin subunit alpha 1
hsa-miR-200b-3p	OCLN	occludin
hsa-miR-200b-3p	CEP85L	centrosomal protein 85 like
hsa-miR-200b-3p	VAT1L	vesicle amine transport 1 like
hsa-miR-200b-3p	SCAMP1	secretory carrier membrane protein 1
hsa-miR-200b-3p	GABBR2	gamma-aminobutyric acid type B receptor subunit 2
hsa-miR-200b-3p	CAB39	calcium binding protein 39
hsa-miR-200b-3p	ADAMTS3	ADAM metallopeptidase with thrombospondin type 1 motif 3
hsa-miR-200b-3p	SCAI	suppressor of cancer cell invasion
hsa-miR-200b-3p	NEDD1	neural precursor cell expressed, developmentally down-regulated 1
hsa-miR-200b-3p	LRRC8A	leucine rich repeat containing 8 VRAC subunit A
hsa-miR-200b-3p	ANKRD44	ankyrin repeat domain 44
hsa-miR-200b-3p	NRBP1	nuclear receptor binding protein 1
hsa-miR-200b-3p	S100PBP	S100P binding protein
hsa-miR-200b-3p	USP25	ubiquitin specific peptidase 25
hsa-miR-200b-3p	MAP4K5	mitogen-activated protein kinase kinase kinase kinase 5
hsa-miR-200b-3p	CAMSAP2	calmodulin regulated spectrin associated protein family member 2
hsa-miR-200b-3p	HDAC9	histone deacetylase 9
hsa-miR-200b-3p	PPP1R9B	protein phosphatase 1 regulatory subunit 9B
hsa-miR-200b-3p	PMAIP1	phorbol-12-myristate-13-acetate-induced protein 1
hsa-miR-200b-3p	MAP4K4	mitogen-activated protein kinase kinase kinase kinase 4
hsa-miR-200b-3p	FSD1L	fibronectin type III and SPRY domain containing 1 like
hsa-miR-200b-3p	STK4	serine/threonine kinase 4
hsa-miR-200b-3p	TSC22D1	TSC22 domain family member 1
hsa-miR-200b-3p	PSIP1	PC4 and SFRS1 interacting protein 1

hsa-miR-200b-3p	MFAP5	microfibril associated protein 5
hsa-miR-200b-3p	SLC4A7	solute carrier family 4 member 7
hsa-miR-200b-3p	NOG	noggin
hsa-miR-200b-3p	SLC6A1	solute carrier family 6 member 1
hsa-miR-200b-3p	MIB1	mindbomb E3 ubiquitin protein ligase 1
hsa-miR-200b-3p	DCBLD2	discoidin, CUB and LCCL domain containing 2
hsa-miR-200b-3p	DENND5B	DENN domain containing 5B
hsa-miR-200b-3p	CCNYL1	cyclin Y like 1
hsa-miR-200b-3p	CKAP4	cytoskeleton associated protein 4
hsa-miR-200b-3p	STRN	striatin
hsa-miR-200b-3p	MARCH6	membrane associated ring-CH-type finger 6
hsa-miR-200b-3p	TMEFF2	transmembrane protein with EGF like and two follistatin like domains 2
hsa-miR-200b-3p	GAS2L3	growth arrest specific 2 like 3
hsa-miR-200b-3p	TLN2	talin 2
hsa-miR-200b-3p	HSPA13	heat shock protein family A (Hsp70) member 13
hsa-miR-200b-3p	SLC14A1	solute carrier family 14 member 1 (Kidd blood group)
hsa-miR-200b-3p	PAG1	phosphoprotein membrane anchor with glycosphingolipid microdomains 1
hsa-miR-200b-3p	NTRK2	neurotrophic receptor tyrosine kinase 2
hsa-miR-200b-3p	PIKFYVE	phosphoinositide kinase, FYVE-type zinc finger containing
hsa-miR-200b-3p	COPS8	COP9 signalosome subunit 8
hsa-miR-200b-3p	USP27X	ubiquitin specific peptidase 27 X-linked
hsa-miR-200b-3p	KYNU	kynureninase
hsa-miR-200b-3p	TSSK1B	testis specific serine kinase 1B
hsa-miR-200b-3p	PTPN12	protein tyrosine phosphatase, non-receptor type 12
hsa-miR-200b-3p	MARCKS	myristoylated alanine rich protein kinase C substrate
hsa-miR-200b-3p	AGFG1	ArfGAP with FG repeats 1
hsa-miR-200b-3p	CBX4	chromobox 4
hsa-miR-200b-3p	CNOT9	CCR4-NOT transcription complex subunit 9
hsa-miR-200b-3p	ATP5F1B	ATP synthase F1 subunit beta
hsa-miR-200b-3p	ZKSCAN8	zinc finger with KRAB and SCAN domains 8
hsa-miR-200b-3p	THAP2	THAP domain containing 2
hsa-miR-200b-3p	TRMT9B	tRNA methyltransferase 9B (putative)
hsa-miR-200b-3p	DIXDC1	DIX domain containing 1
hsa-miR-200b-3p	NOVA1	NOVA alternative splicing regulator 1
hsa-miR-200b-3p	DGKA	diacylglycerol kinase alpha
hsa-miR-200b-3p	ZNF217	zinc finger protein 217
hsa-miR-200b-3p	PCDH19	protocadherin 19
hsa-miR-200b-3p	SCN5A	sodium voltage-gated channel alpha subunit 5
hsa-miR-200b-3p	HS3ST1	heparan sulfate-glucosamine 3-sulfotransferase 1
hsa-miR-200b-3p	PITPNM3	PITPNM family member 3
hsa-miR-200b-3p	GXYLT1	glucoside xylosyltransferase 1
hsa-miR-200b-3p	PIM2	Pim-2 proto-oncogene, serine/threonine kinase
hsa-miR-200b-3p	INTS8	integrator complex subunit 8

hsa-miR-200b-3p	CRH	corticotropin releasing hormone
hsa-miR-200b-3p	RIMS2	regulating synaptic membrane exocytosis 2
hsa-miR-200b-3p	GLI3	GLI family zinc finger 3
hsa-miR-200b-3p	TOGARAM1	TOG array regulator of axonemal microtubules 1
hsa-miR-200b-3p	YPEL2	yippee like 2
hsa-miR-200b-3p	JCAD	junctional cadherin 5 associated
hsa-miR-200b-3p	HDHD2	haloacid dehalogenase like hydrolase domain containing 2
hsa-miR-200b-3p	ENO4	enolase 4
hsa-miR-200b-3p	DENND5A	DENN domain containing 5A
hsa-miR-200b-3p	TIMP2	TIMP metallopeptidase inhibitor 2
hsa-miR-200b-3p	SESN3	sestrin 3
hsa-miR-200b-3p	USP6NL	USP6 N-terminal like
hsa-miR-200b-3p	ERG	ETS transcription factor ERG
hsa-miR-200b-3p	CEP350	centrosomal protein 350
hsa-miR-200b-3p	ZNF326	zinc finger protein 326
hsa-miR-200b-3p	OSBPL11	oxysterol binding protein like 11
hsa-miR-200b-3p	NAP1L5	nucleosome assembly protein 1 like 5
hsa-miR-200b-3p	RBFOX2	RNA binding fox-1 homolog 2
hsa-miR-200b-3p	CYTH3	cytohesin 3
hsa-miR-200b-3p	DPH6	diphthamine biosynthesis 6
hsa-miR-200b-3p	MATR3	matrin 3
hsa-miR-200b-3p	DNA2	DNA replication helicase/nuclease 2
hsa-miR-200b-3p	ATL2	atlastin GTPase 2
hsa-miR-200b-3p	MAPRE1	microtubule associated protein RP/EB family member 1
hsa-miR-200b-3p	ROCK2	Rho associated coiled-coil containing protein kinase 2
hsa-miR-200b-3p	PROK2	prokineticin 2
hsa-miR-200b-3p	DNAJB5	DnaJ heat shock protein family (Hsp40) member B5
hsa-miR-200b-3p	ESRRG	estrogen related receptor gamma
hsa-miR-200b-3p	GNAQ	G protein subunit alpha q
hsa-miR-200b-3p	MAP4K3	mitogen-activated protein kinase kinase kinase kinase 3
hsa-miR-200b-3p	CNEP1R1	CTD nuclear envelope phosphatase 1 regulatory subunit 1
hsa-miR-200b-3p	RIPK2	receptor interacting serine/threonine kinase 2
hsa-miR-200b-3p	ZBTB38	zinc finger and BTB domain containing 38
hsa-miR-200b-3p	FIGNL2	fidgetin like 2
hsa-miR-200b-3p	ATAD2B	ATPase family, AAA domain containing 2B
hsa-miR-200b-3p	FOXN2	forkhead box N2
hsa-miR-200b-3p	ETS1	ETS proto-oncogene 1, transcription factor
hsa-miR-200b-3p	AKAP7	A-kinase anchoring protein 7
hsa-miR-200b-3p	PLPP3	phospholipid phosphatase 3
hsa-miR-200b-3p	SRGAP1	SLIT-ROBO Rho GTPase activating protein 1
hsa-miR-200b-3p	HECTD2	HECT domain E3 ubiquitin protein ligase 2
hsa-miR-200b-3p	ELK3	ELK3, ETS transcription factor
hsa-miR-200b-3p	CEP41	centrosomal protein 41

hsa-miR-200b-3p	CNKSR3	CNKSR family member 3
hsa-miR-200b-3p	ULK2	unc-51 like autophagy activating kinase 2
hsa-miR-200b-3p	ZFPM2	zinc finger protein, FOG family member 2
hsa-miR-200b-3p	ZMAT3	zinc finger matrin-type 3
hsa-miR-200b-3p	RDH10	retinol dehydrogenase 10
hsa-miR-200b-3p	SLC25A36	solute carrier family 25 member 36
hsa-miR-200b-3p	CCDC82	coiled-coil domain containing 82
hsa-miR-200b-3p	SLC30A7	solute carrier family 30 member 7
hsa-miR-200b-3p	SCD	stearoyl-CoA desaturase
hsa-miR-200b-3p	XKR4	XK related 4
hsa-miR-200b-3p	ZCCHC24	zinc finger CCHC-type containing 24
hsa-miR-200b-3p	CNTN1	contactin 1
hsa-miR-200b-3p	SMARCD1	SWI/SNF related, matrix associated, actin dependent regulator of chromatin, subfamily d, member 1
hsa-miR-200b-3p	NPM1	nucleophosmin 1
hsa-miR-200b-3p	ZSWIM4	zinc finger SWIM-type containing 4
hsa-miR-200b-3p	NCS1	neuronal calcium sensor 1
hsa-miR-200b-3p	RO60	Ro60, Y RNA binding protein
hsa-miR-200b-3p	PTAR1	protein prenyltransferase alpha subunit repeat containing 1
hsa-miR-200b-3p	PLCL1	phospholipase C like 1 (inactive)
hsa-miR-200b-3p	CYTH1	cytohesin 1
hsa-miR-200b-3p	TRHDE	thyrotropin releasing hormone degrading enzyme
hsa-miR-200b-3p	BCL11B	BCL11B, BAF complex component
hsa-miR-200b-3p	EDEM3	ER degradation enhancing alpha-mannosidase like protein 3
hsa-miR-200b-3p	FLII	FLII, actin remodeling protein
hsa-miR-200b-3p	KRT80	keratin 80
hsa-miR-200b-3p	EIF2B5	eukaryotic translation initiation factor 2B subunit epsilon
hsa-miR-200b-3p	FERMT2	fermitin family member 2
hsa-miR-200b-3p	SERINC1	serine incorporator 1
hsa-miR-200b-3p	ASAP1	ArfGAP with SH3 domain, ankyrin repeat and PH domain 1
hsa-miR-200b-3p	RANBP10	RAN binding protein 10
hsa-miR-200b-3p	RBFOX3	RNA binding fox-1 homolog 3
hsa-miR-200b-3p	HMGB3	high mobility group box 3
hsa-miR-200b-3p	GJC1	gap junction protein gamma 1
hsa-miR-200b-3p	CORO1C	coronin 1C
hsa-miR-200b-3p	HOOK3	hook microtubule tethering protein 3
hsa-miR-200b-3p	TAF12	TATA-box binding protein associated factor 12
hsa-miR-200b-3p	MARF1	meiosis regulator and mRNA stability factor 1
hsa-miR-200b-3p	NCK2	NCK adaptor protein 2
hsa-miR-200b-3p	TSC22D2	TSC22 domain family member 2
hsa-miR-200b-3p	CPED1	cadherin like and PC-esterase domain containing 1
hsa-miR-200b-3p	TBL1XR1	transducin beta like 1 X-linked receptor 1
hsa-miR-200b-3p	LRP1	LDL receptor related protein 1

hsa-miR-200b-3p	FARP1	FERM, ARH/RhoGEF and pleckstrin domain protein 1
hsa-miR-200b-3p	WWC3	WWC family member 3
hsa-miR-200b-3p	BNC2	basonuclin 2
hsa-miR-200b-3p	PHEX	phosphate regulating endopeptidase homolog X-linked
hsa-miR-200b-3p	PKIA	cAMP-dependent protein kinase inhibitor alpha
hsa-miR-200b-3p	RAP1B	RAP1B, member of RAS oncogene family
hsa-miR-200b-3p	PTHLH	parathyroid hormone like hormone
hsa-miR-200b-3p	UHRF1BP1	UHRF1 binding protein 1
hsa-miR-200b-3p	GPR180	G protein-coupled receptor 180
hsa-miR-200b-3p	C11orf87	chromosome 11 open reading frame 87
hsa-miR-200b-3p	CLASP2	cytoplasmic linker associated protein 2
hsa-miR-200b-3p	KLF4	Kruppel like factor 4
hsa-miR-200b-3p	SCRT2	scratch family transcriptional repressor 2
hsa-miR-200b-3p	RPS6KA3	ribosomal protein S6 kinase A3
hsa-miR-200b-3p	GPATCH8	G-patch domain containing 8
hsa-miR-200b-3p	GEM	GTP binding protein overexpressed in skeletal muscle
hsa-miR-200b-3p	ZDHHC21	zinc finger DHHC-type containing 21
hsa-miR-200b-3p	EXOG	exo/endonuclease G
hsa-miR-200b-3p	SGCE	sarcoglycan epsilon
hsa-miR-200b-3p	SLITRK1	SLIT and NTRK like family member 1
hsa-miR-200b-3p	WASF1	WAS protein family member 1
hsa-miR-200b-3p	INSM2	INSM transcriptional repressor 2
hsa-miR-200b-3p	FLI1	Fli-1 proto-oncogene, ETS transcription factor
hsa-miR-200b-3p	FAM118B	family with sequence similarity 118 member B
hsa-miR-200b-3p	ERICH4	glutamate rich 4
hsa-miR-200b-3p	SIX1	SIX homeobox 1
hsa-miR-200b-3p	TMEM136	transmembrane protein 136
hsa-miR-200b-3p	GTPBP10	GTP binding protein 10
hsa-miR-200b-3p	CHRNA6	cholinergic receptor nicotinic alpha 6 subunit
hsa-miR-200b-3p	EXD2	exonuclease 3'-5' domain containing 2
hsa-miR-200b-3p	TLN1	talin 1
hsa-miR-200b-3p	SNRPB2	small nuclear ribonucleoprotein polypeptide B2
hsa-miR-200b-3p	ZC3H4	zinc finger CCCH-type containing 4
hsa-miR-200b-3p	PI4KB	phosphatidylinositol 4-kinase beta
hsa-miR-200b-3p	SLC39A14	solute carrier family 39 member 14
hsa-miR-200b-3p	PPP2R1B	protein phosphatase 2 scaffold subunit Abeta
hsa-miR-200b-3p	NUDT4	nudix hydrolase 4
hsa-miR-200b-3p	PDIK1L	PDLIM1 interacting kinase 1 like
hsa-miR-200b-3p	UBE2R2	ubiquitin conjugating enzyme E2 R2
hsa-miR-200b-3p	CDYL2	chromodomain Y like 2
hsa-miR-200b-3p	FIGN	fidgetin, microtubule severing factor
hsa-miR-200b-3p	LIN7A	lin-7 homolog A, crumbs cell polarity complex component
hsa-miR-200b-3p	RRP15	ribosomal RNA processing 15 homolog
hsa-miR-200b-3p	HIPK1	homeodomain interacting protein kinase 1
hsa-miR-200b-3p	SRI	sorcin

hsa-miR-200b-3p	JKAMP	JNK1/MAPK8 associated membrane protein
hsa-miR-200b-3p	SDC2	syndecan 2
hsa-miR-200b-3p	PTBP3	polypyrimidine tract binding protein 3
hsa-miR-200b-3p	CUX1	cut like homeobox 1
hsa-miR-200b-3p	DACH1	dachshund family transcription factor 1
hsa-miR-200b-3p	PPP1R10	protein phosphatase 1 regulatory subunit 10
hsa-miR-200b-3p	RDX	radixin
hsa-miR-200b-3p	SLC35E2B	solute carrier family 35 member E2B
hsa-miR-200b-3p	YWHAB	tyrosine 3-monooxygenase/tryptophan 5-monooxygenase activation protein beta
hsa-miR-200b-3p	DDIT4L	DNA damage inducible transcript 4 like
hsa-miR-200b-3p	SYVN1	synoviolin 1
hsa-miR-200b-3p	DZIP1	DAZ interacting zinc finger protein 1
hsa-miR-200b-3p	PPM1B	protein phosphatase, Mg2+/Mn2+ dependent 1B
hsa-miR-200b-3p	SOX2	SRY-box 2
hsa-miR-200b-3p	GLCCI1	glucocorticoid induced 1
hsa-miR-200b-3p	BDP1	B double prime 1, subunit of RNA polymerase III transcription initiation factor IIIB
hsa-miR-200b-3p	STRADB	STE20 related adaptor beta
hsa-miR-200b-3p	HS3ST3A1	heparan sulfate-glucosamine 3-sulfotransferase 3A1
hsa-miR-200b-3p	KIAA0355	KIAA0355
hsa-miR-200b-3p	SLF2	SMC5-SMC6 complex localization factor 2
hsa-miR-200b-3p	ARHGAP20	Rho GTPase activating protein 20
hsa-miR-200b-3p	IKZF2	IKAROS family zinc finger 2
hsa-miR-200b-3p	SLC35E2A	solute carrier family 35 member E2A
hsa-miR-200b-3p	APOO	apolipoprotein O
hsa-miR-200b-3p	SLC24A4	solute carrier family 24 member 4
hsa-miR-200b-3p	FSTL1	follistatin like 1
hsa-miR-200b-3p	CASZ1	castor zinc finger 1
hsa-miR-200b-3p	CTDSPL2	CTD small phosphatase like 2
hsa-miR-200b-3p	PCLAF	PCNA clamp associated factor
hsa-miR-200b-3p	FNDC3B	fibronectin type III domain containing 3B
hsa-miR-200b-3p	A1CF	APOBEC1 complementation factor
hsa-miR-200b-3p	EIF4E2	eukaryotic translation initiation factor 4E family member 2
hsa-miR-200b-3p	NECTIN4	nectin cell adhesion molecule 4
hsa-miR-200b-3p	TMEM164	transmembrane protein 164
hsa-miR-200b-3p	PPP1R9A	protein phosphatase 1 regulatory subunit 9A
hsa-miR-200b-3p	SNX16	sorting nexin 16
hsa-miR-200b-3p	GTF3C4	general transcription factor IIIC subunit 4
hsa-miR-200b-3p	RGL1	ral guanine nucleotide dissociation stimulator like 1
hsa-miR-200b-3p	SUZ12	SUZ12, polycomb repressive complex 2 subunit
hsa-miR-200b-3p	CHSY1	chondroitin sulfate synthase 1
hsa-miR-200b-3p	KANK2	KN motif and ankyrin repeat domains 2
hsa-miR-200b-3p	GSTA4	glutathione S-transferase alpha 4
hsa-miR-200b-3p	CLVS2	clavesin 2

hsa-miR-200b-3p	NPC1	NPC intracellular cholesterol transporter 1
hsa-miR-200b-3p	PLXNC1	plexin C1
hsa-miR-200b-3p	ARID4B	AT-rich interaction domain 4B
hsa-miR-200b-3p	TUBB	tubulin beta class I
hsa-miR-200b-3p	NAB1	NGFI-A binding protein 1
hsa-miR-200b-3p	MBNL2	muscleblind like splicing regulator 2
hsa-miR-200b-3p	BICC1	BicC family RNA binding protein 1
hsa-miR-200b-3p	CEP97	centrosomal protein 97
hsa-miR-200b-3p	ANK3	ankyrin 3
hsa-miR-200b-3p	MYZAP	myocardial zonula adherens protein
hsa-miR-200b-3p	DPY19L3	dpy-19 like C-mannosyltransferase 3
hsa-miR-200b-3p	SEMA3F	semaphorin 3F
hsa-miR-200b-3p	IGF2R	insulin like growth factor 2 receptor
hsa-miR-200b-3p	TAP2	transporter 2, ATP binding cassette subfamily B member
hsa-miR-200b-3p	LEPR	leptin receptor
hsa-miR-200b-3p	CASR	calcium sensing receptor
hsa-miR-200b-3p	DCUN1D5	defective in cullin neddylation 1 domain containing 5
hsa-miR-200b-3p	HNRNPD	heterogeneous nuclear ribonucleoprotein D
hsa-miR-200b-3p	DENND1B	DENN domain containing 1B
hsa-miR-200b-3p	MTF2	metal response element binding transcription factor 2
hsa-miR-200b-3p	UBE2W	ubiquitin conjugating enzyme E2 W
hsa-miR-200b-3p	TBCA	tubulin folding cofactor A
hsa-miR-200b-3p	MYB	MYB proto-oncogene, transcription factor
hsa-miR-200b-3p	PLPPR4	phospholipid phosphatase related 4
hsa-miR-200b-3p	TRIM23	tripartite motif containing 23
hsa-miR-200b-3p	IER5	immediate early response 5
hsa-miR-200b-3p	CCNE2	cyclin E2
hsa-miR-200b-3p	CRTAP	cartilage associated protein
hsa-miR-200b-3p	NUFIP2	nuclear FMR1 interacting protein 2
hsa-miR-200b-3p	SECISBP2L	SECIS binding protein 2 like
hsa-miR-200b-3p	NOTCH1	notch 1
hsa-miR-200b-3p	PRDM1	PR/SET domain 1
hsa-miR-200b-3p	ALDH1A1	aldehyde dehydrogenase 1 family member A1
hsa-miR-200b-3p	DNAJB14	DnaJ heat shock protein family (Hsp40) member B14
hsa-miR-200b-3p	GATA4	GATA binding protein 4
hsa-miR-200b-3p	GPATCH2L	G-patch domain containing 2 like
hsa-miR-200b-3p	KIF14	kinesin family member 14
hsa-miR-200b-3p	NYAP1	neuronal tyrosine phosphorylated phosphoinositide-3-kinase adaptor 1
hsa-miR-200b-3p	SCN8A	sodium voltage-gated channel alpha subunit 8
hsa-miR-200b-3p	ATP6V0A2	ATPase H+ transporting V0 subunit a2
hsa-miR-200b-3p	NRG1	neuregulin 1
hsa-miR-200b-3p	ACVR1C	activin A receptor type 1C
hsa-miR-200b-3p	MTSS1L	MTSS1L, I-BAR domain containing
hsa-miR-200b-3p	ADH1B	alcohol dehydrogenase 1B (class I), beta polypeptide

hsa-miR-200b-3p	GUCY1A1	guanylate cyclase 1 soluble subunit alpha 1
hsa-miR-200b-3p	SBSPON	somatomedin B and thrombospondin type 1 domain containing
hsa-miR-200b-3p	PICALM	phosphatidylinositol binding clathrin assembly protein
hsa-miR-200b-3p	KLF6	Kruppel like factor 6
hsa-miR-200b-3p	CHMP5	charged multivesicular body protein 5
hsa-miR-200b-3p	EFNB2	ephrin B2
hsa-miR-200b-3p	MEX3D	mex-3 RNA binding family member D
hsa-miR-200b-3p	KCND2	potassium voltage-gated channel subfamily D member 2
hsa-miR-200b-3p	SIKE1	suppressor of IKBKE 1
hsa-miR-200b-3p	MSL2	MSL complex subunit 2
hsa-miR-200b-3p	ZNF224	zinc finger protein 224
hsa-miR-200b-3p	FRMD4B	FERM domain containing 4B
hsa-miR-200b-3p	OXR1	oxidation resistance 1
hsa-miR-200b-3p	CDH11	cadherin 11
hsa-miR-200b-3p	IPO8	importin 8
hsa-miR-200b-3p	BPTF	bromodomain PHD finger transcription factor
hsa-miR-200b-3p	RSPRY1	ring finger and SPRY domain containing 1
hsa-miR-200b-3p	NDN	necdin, MAGE family member
hsa-miR-200b-3p	THSD7A	thrombospondin type 1 domain containing 7A
hsa-miR-200b-3p	MMGT1	membrane magnesium transporter 1
hsa-miR-200b-3p	STX17	syntaxin 17
hsa-miR-200b-3p	PHF6	PHD finger protein 6
hsa-miR-200b-3p	ARHGEF17	Rho guanine nucleotide exchange factor 17
hsa-miR-200b-3p	RABIF	RAB interacting factor
hsa-miR-200b-3p	ETV5	ETS variant 5
hsa-miR-200b-3p	CADM1	cell adhesion molecule 1
hsa-miR-200b-3p	TMBIM4	transmembrane BAX inhibitor motif containing 4
hsa-miR-200b-3p	ANLN	anillin actin binding protein
hsa-miR-200b-3p	NIN	ninein
hsa-miR-200b-3p	TAF9B	TATA-box binding protein associated factor 9b
hsa-miR-200b-3p	BAG5	BCL2 associated athanogene 5
hsa-miR-200b-3p	PAPOLA	poly(A) polymerase alpha
hsa-miR-200b-3p	NSD2	nuclear receptor binding SET domain protein 2
hsa-miR-200b-3p	UBQLN1	ubiquilin 1
hsa-miR-200b-3p	SHROOM2	shroom family member 2
hsa-miR-200b-3p	LAMC1	laminin subunit gamma 1
hsa-miR-200b-3p	SYNCRIP	synaptotagmin binding cytoplasmic RNA interacting protein
hsa-miR-200b-3p	CPEB3	cytoplasmic polyadenylation element binding protein 3
hsa-miR-200b-3p	DCT	dopachrome tautomerase
hsa-miR-200b-3p	CYP11B1	cytochrome P450 family 11 subfamily B member 1
hsa-miR-200b-3p	ROBO2	roundabout guidance receptor 2
hsa-miR-200b-3p	XKR6	XK related 6
hsa-miR-200b-3p	STXBP6	syntaxin binding protein 6

hsa-miR-200b-3p	SWAP70	switching B cell complex subunit SWAP70
hsa-miR-200b-3p	LATS2	large tumor suppressor kinase 2
hsa-miR-200b-3p	FYN	FYN proto-oncogene, Src family tyrosine kinase
hsa-miR-200b-3p	PPP1CB	protein phosphatase 1 catalytic subunit beta
hsa-miR-200b-3p	RIMKLB	ribosomal modification protein rimK like family member B
hsa-miR-200b-3p	SASH1	SAM and SH3 domain containing 1
hsa-miR-200b-3p	NUP160	nucleoporin 160
hsa-miR-200b-3p	SHOX2	short stature homeobox 2
hsa-miR-200b-3p	HOXA5	homeobox A5
hsa-miR-200b-3p	GABPA	GA binding protein transcription factor subunit alpha
hsa-miR-200b-3p	SLC15A5	solute carrier family 15 member 5
hsa-miR-200b-3p	KANK1	KN motif and ankyrin repeat domains 1
hsa-miR-200b-3p	LMO7	LIM domain 7
hsa-miR-200b-3p	TMA16	translation machinery associated 16 homolog
hsa-miR-200b-3p	PCSK2	proprotein convertase subtilisin/kexin type 2
hsa-miR-200b-3p	SH3GL1	SH3 domain containing GRB2 like 1, endophilin A2
hsa-miR-200b-3p	SLC38A2	solute carrier family 38 member 2
hsa-miR-200b-3p	CNN3	calponin 3
hsa-miR-200b-3p	ZNF302	zinc finger protein 302
hsa-miR-200b-3p	RNF5	ring finger protein 5
hsa-miR-200b-3p	FSIP1	fibrous sheath interacting protein 1
hsa-miR-200b-3p	LRRC58	leucine rich repeat containing 58
hsa-miR-200b-3p	CNTFR	ciliary neurotrophic factor receptor
hsa-miR-200b-3p	ZFHX4	zinc finger homeobox 4
hsa-miR-200b-3p	ELMOD1	ELMO domain containing 1
hsa-miR-200b-3p	CASP2	caspase 2
hsa-miR-200b-3p	LCA5	LCA5, lebercilin
hsa-miR-200b-3p	RAPGEF2	Rap guanine nucleotide exchange factor 2
hsa-miR-200b-3p	IKBKB	inhibitor of nuclear factor kappa B kinase subunit beta
hsa-miR-200b-3p	SPTSSA	serine palmitoyltransferase small subunit A
hsa-miR-200b-3p	FUNDC1	FUN14 domain containing 1
hsa-miR-200b-3p	UBXN8	UBX domain protein 8
hsa-miR-200b-3p	SRP72	signal recognition particle 72
hsa-miR-200b-3p	NFIB	nuclear factor I B
hsa-miR-200b-3p	USP18	ubiquitin specific peptidase 18
hsa-miR-200b-3p	TBCK	TBC1 domain containing kinase
hsa-miR-200b-3p	LARP1B	La ribonucleoprotein domain family member 1B
hsa-miR-200b-3p	RNF180	ring finger protein 180
hsa-miR-200b-3p	UBE2D1	ubiquitin conjugating enzyme E2 D1
hsa-miR-200b-3p	GTF2E1	general transcription factor IIE subunit 1
hsa-miR-200b-3p	SBF1	SET binding factor 1
hsa-miR-200b-3p	SIX3	SIX homeobox 3
hsa-miR-200b-3p	UBA6	ubiquitin like modifier activating enzyme 6
hsa-miR-200b-3p	PLK2	polo like kinase 2
hsa-miR-200b-3p	SUPT20H	SPT20 homolog, SAGA complex component

hsa-miR-200b-3p	YAP1	Yes associated protein 1
hsa-miR-200b-3p	KLHL29	kelch like family member 29
hsa-miR-200b-3p	ZBTB20	zinc finger and BTB domain containing 20
hsa-miR-200b-3p	C6orf120	chromosome 6 open reading frame 120
hsa-miR-200b-3p	TRIM71	tripartite motif containing 71
hsa-miR-200b-3p	SLC16A2	solute carrier family 16 member 2
hsa-miR-200b-3p	GNAI3	G protein subunit alpha i3
hsa-miR-200b-3p	EFNA1	ephrin A1
hsa-miR-200b-3p	USP43	ubiquitin specific peptidase 43
hsa-miR-200b-3p	PAK6	p21 (RAC1) activated kinase 6
hsa-miR-200b-3p	KMT2C	lysine methyltransferase 2C
hsa-miR-200b-3p	AMBRA1	autophagy and beclin 1 regulator 1
hsa-miR-200b-3p	RASSF6	Ras association domain family member 6
hsa-miR-200b-3p	EPHA5	EPH receptor A5
hsa-miR-200b-3p	KDSR	3-ketodihydrosphingosine reductase
hsa-miR-200b-3p	MSANTD2	Myb/SANT DNA binding domain containing 2
hsa-miR-200b-3p	ZNF697	zinc finger protein 697
hsa-miR-200b-3p	PRKN	parkin RBR E3 ubiquitin protein ligase
hsa-miR-200b-3p	CDR2	cerebellar degeneration related protein 2
hsa-miR-200b-3p	ASH1L	ASH1 like histone lysine methyltransferase
hsa-miR-200b-3p	TIFA	TRAF interacting protein with forkhead associated domain
hsa-miR-200b-3p	SLC4A4	solute carrier family 4 member 4
hsa-miR-200b-3p	ANO5	anoctamin 5
hsa-miR-200b-3p	MXD4	MAX dimerization protein 4
hsa-miR-200b-3p	NRXN1	neurexin 1
hsa-miR-200b-3p	TMX1	thioredoxin related transmembrane protein 1
hsa-miR-200b-3p	SLC39A8	solute carrier family 39 member 8
hsa-miR-200b-3p	B4GALT6	beta-1,4-galactosyltransferase 6
hsa-miR-200b-3p	GAB1	GRB2 associated binding protein 1
hsa-miR-200b-3p	AAK1	AP2 associated kinase 1
hsa-miR-200b-3p	DNAJB6	DnaJ heat shock protein family (Hsp40) member B6
hsa-miR-200b-3p	STYX	serine/threonine/tyrosine interacting protein
hsa-miR-200b-3p	TAF4	TATA-box binding protein associated factor 4
hsa-miR-200b-3p	KCNMB1	potassium calcium-activated channel subfamily M regulatory beta subunit 1
hsa-miR-200b-3p	RNF169	ring finger protein 169
hsa-miR-200b-3p	C4orf46	chromosome 4 open reading frame 46
hsa-miR-200b-3p	FAM227A	family with sequence similarity 227 member A
hsa-miR-200b-3p	CPPED1	calcineurin like phosphoesterase domain containing 1
hsa-miR-200b-3p	ICK	intestinal cell kinase
hsa-miR-200b-3p	TMEM135	transmembrane protein 135
hsa-miR-200b-3p	TBC1D22B	TBC1 domain family member 22B
hsa-miR-200b-3p	CCDC186	coiled-coil domain containing 186
hsa-miR-200b-3p	SLC35A2	solute carrier family 35 member A2

hsa-miR-200b-3p	SEC24A	SEC24 homolog A, COPII coat complex component
hsa-miR-200b-3p	DNAJC10	DnaJ heat shock protein family (Hsp40) member C10
hsa-miR-200b-3p	GRAP2	GRB2 related adaptor protein 2
hsa-miR-200b-3p	KPNA4	karyopherin subunit alpha 4
hsa-miR-200b-3p	YTHDF3	YTH N6-methyladenosine RNA binding protein 3
hsa-miR-200b-3p	NCOA7	nuclear receptor coactivator 7
hsa-miR-200b-3p	FBXO22	F-box protein 22
hsa-miR-200b-3p	UBE2I	ubiquitin conjugating enzyme E2 I
hsa-miR-200b-3p	ADRB2	adrenoceptor beta 2
hsa-miR-200b-3p	UBAC2	UBA domain containing 2
hsa-miR-200b-3p	RAB8B	RAB8B, member RAS oncogene family
hsa-miR-200b-3p	RFTN2	raftlin family member 2
hsa-miR-200b-3p	ADCY9	adenylate cyclase 9
hsa-miR-200b-3p	PCDH7	protocadherin 7
hsa-miR-200b-3p	MIER3	MIER family member 3
hsa-miR-200b-3p	PHACTR2	phosphatase and actin regulator 2
hsa-miR-200b-3p	CACUL1	CDK2 associated cullin domain 1
hsa-miR-200b-3p	BLOC1S6	biogenesis of lysosomal organelles complex 1 subunit 6
hsa-miR-200b-3p	SOX1	SRY-box 1
hsa-miR-200b-3p	KIAA1841	KIAA1841
hsa-miR-200b-3p	PRKAR1A	protein kinase cAMP-dependent type I regulatory subunit alpha
hsa-miR-200b-3p	HTR2A	5-hydroxytryptamine receptor 2A
hsa-miR-200b-3p	FUT9	fucosyltransferase 9
hsa-miR-200b-3p	ACVR2B	activin A receptor type 2B
hsa-miR-200b-3p	LONRF2	LON peptidase N-terminal domain and ring finger 2
hsa-miR-200b-3p	RRAS2	RAS related 2
hsa-miR-200b-3p	ENOX2	ecto-NOX disulfide-thiol exchanger 2
hsa-miR-200b-3p	ZFY	zinc finger protein Y-linked
hsa-miR-200b-3p	TM7SF3	transmembrane 7 superfamily member 3
hsa-miR-200b-3p	MTMR6	myotubularin related protein 6
hsa-miR-200b-3p	MAGEC2	MAGE family member C2
hsa-miR-200b-3p	ZFR	zinc finger RNA binding protein
hsa-miR-200b-3p	AP1AR	adaptor related protein complex 1 associated regulatory protein
hsa-miR-200b-3p	NCOA4	nuclear receptor coactivator 4
hsa-miR-200b-3p	PSTPIP2	proline-serine-threonine phosphatase interacting protein 2
hsa-miR-200b-3p	FUBP3	far upstream element binding protein 3
hsa-miR-200b-3p	SHCBP1	SHC binding and spindle associated 1
hsa-miR-200b-3p	TIAL1	TIA1 cytotoxic granule associated RNA binding protein like 1
hsa-miR-200b-3p	MEX3B	mex-3 RNA binding family member B
hsa-miR-200b-3p	GPR173	G protein-coupled receptor 173
hsa-miR-200b-3p	SYNC	syncoilin, intermediate filament protein
hsa-miR-200b-3p	GIGYF1	GRB10 interacting GYF protein 1

hsa-miR-200b-3p	IQCJ-SCHIP1	IQCJ-SCHIP1 readthrough
hsa-miR-200b-3p	XG	Xg glycoprotein (Xg blood group)
hsa-miR-200b-3p	ZBTB8A	zinc finger and BTB domain containing 8A
hsa-miR-200b-3p	TMEM170B	transmembrane protein 170B
hsa-miR-200b-3p	UTY	ubiquitously transcribed tetratricopeptide repeat containing, Y-linked
hsa-miR-200b-3p	EIF5B	eukaryotic translation initiation factor 5B
hsa-miR-200b-3p	NCAM1	neural cell adhesion molecule 1
hsa-miR-200b-3p	PAIP2	poly(A) binding protein interacting protein 2
hsa-miR-200b-3p	RBFOX1	RNA binding fox-1 homolog 1
hsa-miR-200b-3p	SNAP25	synaptosome associated protein 25
hsa-miR-200b-3p	EP300	E1A binding protein p300
hsa-miR-200b-3p	CERS6	ceramide synthase 6
hsa-miR-200b-3p	GMFB	glia maturation factor beta
hsa-miR-200b-3p	NFYA	nuclear transcription factor Y subunit alpha
hsa-miR-200b-3p	PARD3B	par-3 family cell polarity regulator beta
hsa-miR-200b-3p	HNF1B	HNF1 homeobox B
hsa-miR-200b-3p	MARCH8	membrane associated ring-CH-type finger 8
hsa-miR-200b-3p	ZDHHC17	zinc finger DHHC-type containing 17
hsa-miR-200b-3p	C5orf24	chromosome 5 open reading frame 24
hsa-miR-200b-3p	WDR91	WD repeat domain 91
hsa-miR-200b-3p	COG6	component of oligomeric golgi complex 6
hsa-miR-200b-3p	MSTN	myostatin
hsa-miR-200b-3p	CREBBP	CREB binding protein
hsa-miR-200b-3p	NDNF	neuron derived neurotrophic factor
hsa-miR-200b-3p	ACACA	acetyl-CoA carboxylase alpha
hsa-miR-200b-3p	OPRM1	opioid receptor mu 1
hsa-miR-200b-3p	CALU	calumenin
hsa-miR-200b-3p	RSRC2	arginine and serine rich coiled-coil 2
hsa-miR-200b-3p	LPAR1	lysophosphatidic acid receptor 1
hsa-miR-200b-3p	PTPN22	protein tyrosine phosphatase, non-receptor type 22
hsa-miR-200b-3p	RHOT1	ras homolog family member T1
hsa-miR-200b-3p	N4BP2	NEDD4 binding protein 2
hsa-miR-200b-3p	RNF38	ring finger protein 38
hsa-miR-200b-3p	RBM26	RNA binding motif protein 26
hsa-miR-200b-3p	SLTM	SAFB like transcription modulator
hsa-miR-200b-3p	CLOCK	clock circadian regulator
hsa-miR-200b-3p	CDC73	cell division cycle 73
hsa-miR-200b-3p	PLS3	plastin 3
hsa-miR-200b-3p	ADCY2	adenylate cyclase 2
hsa-miR-200b-3p	CALN1	calneuron 1
hsa-miR-200b-3p	C15orf40	chromosome 15 open reading frame 40
hsa-miR-200b-3p	TP73	tumor protein p73
hsa-miR-200b-3p	FRMD4A	FERM domain containing 4A
hsa-miR-200b-3p	TMEM170A	transmembrane protein 170A

hsa-miR-200b-3p	DCAF17	DDB1 and CUL4 associated factor 17
hsa-miR-200b-3p	ANGEL2	angel homolog 2
hsa-miR-200b-3p	RPRD1A	regulation of nuclear pre-mRNA domain containing 1A
hsa-miR-200b-3p	EHD1	EH domain containing 1
hsa-miR-200b-3p	ACADSB	acyl-CoA dehydrogenase short/branched chain
hsa-miR-200b-3p	ARL15	ADP ribosylation factor like GTPase 15
hsa-miR-200b-3p	ALG2	ALG2, alpha-1,3/1,6-mannosyltransferase
hsa-miR-200b-3p	PALM2	paralemmin 2
hsa-miR-200b-3p	PPWD1	peptidylprolyl isomerase domain and WD repeat containing 1
hsa-miR-200b-3p	ARGLU1	arginine and glutamate rich 1
hsa-miR-200b-3p	TMEM33	transmembrane protein 33
hsa-miR-200b-3p	PPARA	peroxisome proliferator activated receptor alpha
hsa-miR-200b-3p	PTGER2	prostaglandin E receptor 2
hsa-miR-200b-3p	C19orf12	chromosome 19 open reading frame 12
hsa-miR-200b-3p	BAG6	BCL2 associated athanogene 6
hsa-miR-200b-3p	TLL2	tolloid like 2
hsa-miR-200b-3p	ANKRD33B	ankyrin repeat domain 33B
hsa-miR-200b-3p	PNISR	PNN interacting serine and arginine rich protein
hsa-miR-200b-3p	DOCK4	dedicator of cytokinesis 4
hsa-miR-200b-3p	MTMR9	myotubularin related protein 9
hsa-miR-200b-3p	ZNF557	zinc finger protein 557
hsa-miR-200b-3p	C2CD6	C2 calcium dependent domain containing 6
hsa-miR-200b-3p	FAM81A	family with sequence similarity 81 member A
hsa-miR-200b-3p	FASTKD3	FAST kinase domains 3
hsa-miR-200b-3p	TMEM245	transmembrane protein 245
hsa-miR-200b-3p	MON2	MON2 homolog, regulator of endosome-to-Golgi trafficking
hsa-miR-200b-3p	MXD3	MAX dimerization protein 3
hsa-miR-200b-3p	KIF11	kinesin family member 11
hsa-miR-200b-3p	AFF4	AF4/FMR2 family member 4
hsa-miR-200b-3p	SLC6A15	solute carrier family 6 member 15
hsa-miR-200b-3p	CNST	consortin, connexin sorting protein
hsa-miR-200b-3p	PPP2CA	protein phosphatase 2 catalytic subunit alpha
hsa-miR-200b-3p	CTNND2	catenin delta 2
hsa-miR-200b-3p	PHC3	polyhomeotic homolog 3
hsa-miR-200b-3p	ORMDL3	ORMDL sphingolipid biosynthesis regulator 3
hsa-miR-200b-3p	SRF	serum response factor
hsa-miR-200b-3p	CASK	calcium/calmodulin dependent serine protein kinase
hsa-miR-200b-3p	LCORL	ligand dependent nuclear receptor corepressor like
hsa-miR-200b-3p	YPEL1	yippee like 1
hsa-miR-200b-3p	GABRB3	gamma-aminobutyric acid type A receptor beta3 subunit
hsa-miR-200b-3p	TFPI	tissue factor pathway inhibitor
hsa-miR-200b-3p	UNC80	unc-80 homolog, NALCN channel complex subunit
hsa-miR-200b-3p	FLT4	fms related tyrosine kinase 4

hsa-miR-200b-3p	MKLN1	muskelin 1
hsa-miR-200b-3p	STARD13	StAR related lipid transfer domain containing 13
hsa-miR-200b-3p	BPY2C	basic charge Y-linked 2C
hsa-miR-200b-3p	MAMDC2	MAM domain containing 2
hsa-miR-200b-3p	BPY2B	basic charge Y-linked 2B
hsa-miR-200b-3p	SSR3	signal sequence receptor subunit 3
hsa-miR-200b-3p	SLC25A27	solute carrier family 25 member 27
hsa-miR-200b-3p	FUT4	fucosyltransferase 4
hsa-miR-200b-3p	LFNG	LFNG O-fucosylpeptide 3-beta-N-acetylglucosaminyltransferase
hsa-miR-200b-3p	UBE2V1	ubiquitin conjugating enzyme E2 V1
hsa-miR-200b-3p	FHOD1	formin homology 2 domain containing 1
hsa-miR-200b-3p	SYT1	synaptotagmin 1
hsa-miR-200b-3p	TIGAR	TP53 induced glycolysis regulatory phosphatase
hsa-miR-200b-3p	FBXW11	F-box and WD repeat domain containing 11
hsa-miR-200b-3p	DSG1	desmoglein 1
hsa-miR-200b-3p	LETM2	leucine zipper and EF-hand containing transmembrane protein 2
hsa-miR-200b-3p	MYT1	myelin transcription factor 1
hsa-miR-200b-3p	TAB3	TGF-beta activated kinase 1 (MAP3K7) binding protein 3
hsa-miR-200b-3p	PCNX1	pecanex 1
hsa-miR-200b-3p	ZNF292	zinc finger protein 292
hsa-miR-200b-3p	TBX5	T-box 5
hsa-miR-200b-3p	BPY2	basic charge Y-linked 2
hsa-miR-200b-3p	MMD2	monocyte to macrophage differentiation associated 2
hsa-miR-200b-3p	RAC1	Rac family small GTPase 1
hsa-miR-200b-3p	TMEM189-UBE2V1	TMEM189-UBE2V1 readthrough
hsa-miR-200b-3p	STAM2	signal transducing adaptor molecule 2
hsa-miR-200b-3p	CHST2	carbohydrate sulfotransferase 2
hsa-miR-200b-3p	GLRX3	glutaredoxin 3
hsa-miR-200b-3p	NLGN4X	neuroligin 4 X-linked
hsa-miR-200b-3p	BTF3L4	basic transcription factor 3 like 4
hsa-miR-200b-3p	SOWAHC	sosondowah ankyrin repeat domain family member C
hsa-miR-200b-3p	SGPP1	sphingosine-1-phosphate phosphatase 1
hsa-miR-200b-3p	PRRG4	proline rich and Gla domain 4
hsa-miR-200b-3p	ZC3H12B	zinc finger CCCH-type containing 12B
hsa-miR-200b-3p	NPTX1	neuronal pentraxin 1
hsa-miR-200b-3p	TMEM167A	transmembrane protein 167A
hsa-miR-200b-3p	HNRNPH3	heterogeneous nuclear ribonucleoprotein H3
hsa-miR-200b-3p	MTFR1	mitochondrial fission regulator 1
hsa-miR-200b-3p	RBL1	RB transcriptional corepressor like 1
hsa-miR-200b-3p	ELL	elongation factor for RNA polymerase II
hsa-miR-200b-3p	TBX22	T-box 22
hsa-miR-200b-3p	RLF	rearranged L-myc fusion

hsa-miR-200b-3p	CNTN4	contactin 4
hsa-miR-200b-3p	C6orf62	chromosome 6 open reading frame 62
hsa-miR-200b-3p	CADM2	cell adhesion molecule 2
hsa-miR-200b-3p	CNOT6L	CCR4-NOT transcription complex subunit 6 like
hsa-miR-200b-3p	PMM1	phosphomannomutase 1
hsa-miR-200b-3p	DIRAS2	DIRAS family GTPase 2
hsa-miR-200b-3p	SHC4	SHC adaptor protein 4
hsa-miR-200b-3p	TJAP1	tight junction associated protein 1
hsa-miR-200b-3p	RBM12B	RNA binding motif protein 12B
hsa-miR-200b-3p	LATS1	large tumor suppressor kinase 1
hsa-miR-200b-3p	EIF5A2	eukaryotic translation initiation factor 5A2
hsa-miR-200b-3p	UGCG	UDP-glucose ceramide glucosyltransferase
hsa-miR-200b-3p	RAB33B	RAB33B, member RAS oncogene family
hsa-miR-200b-3p	ZNF605	zinc finger protein 605
hsa-miR-200b-3p	PLOD2	procollagen-lysine,2-oxoglutarate 5-dioxygenase 2
hsa-miR-200b-3p	PHLDB1	pleckstrin homology like domain family B member 1
hsa-miR-200b-3p	SRSF1	serine and arginine rich splicing factor 1
hsa-miR-200b-3p	RPL22L1	ribosomal protein L22 like 1
hsa-miR-200b-3p	ARFGEF3	ARFGEF family member 3
hsa-miR-200b-3p	CYP1B1	cytochrome P450 family 1 subfamily B member 1
hsa-miR-200b-3p	NGEF	neuronal guanine nucleotide exchange factor
hsa-miR-200b-3p	TSTD3	thiosulfate sulfurtransferase like domain containing 3
hsa-miR-200b-3p	EFHC2	EF-hand domain containing 2
hsa-miR-200b-3p	PAK3	p21 (RAC1) activated kinase 3
hsa-miR-200b-3p	STON2	stonin 2
hsa-miR-200b-3p	SLC23A2	solute carrier family 23 member 2
hsa-miR-200b-3p	KLRF1	killer cell lectin like receptor F1
hsa-miR-200b-3p	ESM1	endothelial cell specific molecule 1
hsa-miR-200b-3p	MED6	mediator complex subunit 6
hsa-miR-200b-3p	SLC6A17	solute carrier family 6 member 17
hsa-miR-200b-3p	PPP2R2C	protein phosphatase 2 regulatory subunit Bgamma
hsa-miR-200b-3p	FAR2	fatty acyl-CoA reductase 2
hsa-miR-200b-3p	ZNF652	zinc finger protein 652
hsa-miR-200b-3p	LIN28B	lin-28 homolog B
hsa-miR-200b-3p	LCOR	ligand dependent nuclear receptor corepressor
hsa-miR-200b-3p	CALCR	calcitonin receptor
hsa-miR-200b-3p	ALDH1A3	aldehyde dehydrogenase 1 family member A3
hsa-miR-200b-3p	UBN2	ubinuclein 2
hsa-miR-200b-3p	BTBD18	BTB domain containing 18
hsa-miR-200b-3p	PDLIM5	PDZ and LIM domain 5
hsa-miR-200b-3p	PAK5	p21 (RAC1) activated kinase 5
hsa-miR-200b-3p	MSR1	macrophage scavenger receptor 1
hsa-miR-200b-3p	GPX6	glutathione peroxidase 6
hsa-miR-200b-3p	HIPK2	homeodomain interacting protein kinase 2
hsa-miR-200b-3p	SIRPA	signal regulatory protein alpha

hsa-miR-200b-3p	SLC35F4	solute carrier family 35 member F4
hsa-miR-200b-3p	JMY	junction mediating and regulatory protein, p53 cofactor
hsa-miR-200b-3p	ASF1A	anti-silencing function 1A histone chaperone
hsa-miR-200b-3p	INPP4A	inositol polyphosphate-4-phosphatase type I A
hsa-miR-200b-3p	SNAI2	snail family transcriptional repressor 2
hsa-miR-200b-3p	LPIN1	lipin 1
hsa-miR-200b-3p	TERF1	telomeric repeat binding factor 1
hsa-miR-200b-3p	RUNX1T1	RUNX1 translocation partner 1
hsa-miR-200b-3p	TAOK1	TAO kinase 1
hsa-miR-200b-3p	TXLNG	taxilin gamma
hsa-miR-200b-3p	NPNT	nephronectin
hsa-miR-200b-3p	MAPK1IP1L	mitogen-activated protein kinase 1 interacting protein 1 like
hsa-miR-200b-3p	SH3PXD2A	SH3 and PX domains 2A
hsa-miR-200b-3p	ZNF555	zinc finger protein 555
hsa-miR-200b-3p	ASXL3	ASXL transcriptional regulator 3
hsa-miR-200b-3p	PCNP	PEST proteolytic signal containing nuclear protein
hsa-miR-200b-3p	SHOC1	shortage in chiasmata 1
hsa-miR-200b-3p	ZNF181	zinc finger protein 181
hsa-miR-200b-3p	PIP4K2B	phosphatidylinositol-5-phosphate 4-kinase type 2 beta
hsa-miR-200b-3p	PLEKHM3	pleckstrin homology domain containing M3
hsa-miR-200b-3p	NCOA3	nuclear receptor coactivator 3
hsa-miR-200b-3p	ARHGAP19	Rho GTPase activating protein 19
hsa-miR-200b-3p	FXR2	FMR1 autosomal homolog 2
hsa-miR-200b-3p	SNX13	sorting nexin 13
hsa-miR-200b-3p	PLCG1	phospholipase C gamma 1
hsa-miR-200b-3p	PHF21A	PHD finger protein 21A
hsa-miR-200b-3p	GIN1	gypsy retrotransposon integrase 1
hsa-miR-200b-3p	TRAM1L1	translocation associated membrane protein 1 like 1
hsa-miR-200b-3p	THAP5	THAP domain containing 5
hsa-miR-200b-3p	SNAPC1	small nuclear RNA activating complex polypeptide 1
hsa-miR-200b-3p	C2orf49	chromosome 2 open reading frame 49
hsa-miR-200b-3p	TPCN1	two pore segment channel 1
hsa-miR-200b-3p	PTS	6-pyruvoyltetrahydropterin synthase
hsa-miR-200b-3p	PARD6B	par-6 family cell polarity regulator beta
hsa-miR-200b-3p	CDK15	cyclin dependent kinase 15
hsa-miR-200b-3p	THRB	thyroid hormone receptor beta
hsa-miR-200b-3p	MPP4	membrane palmitoylated protein 4
hsa-miR-200b-3p	RELN	reelin
hsa-miR-200b-3p	ZNF516	zinc finger protein 516
hsa-miR-200b-3p	KLK7	kallikrein related peptidase 7
hsa-miR-200b-3p	PAX6	paired box 6
hsa-miR-200b-3p	KLHL31	kelch like family member 31
hsa-miR-200b-3p	NCOR2	nuclear receptor corepressor 2
hsa-miR-200b-3p	KCTD15	potassium channel tetramerization domain containing 15

hsa-miR-200b-3p	PSPH	phosphoserine phosphatase
hsa-miR-200b-3p	PIN1	peptidylprolyl cis/trans isomerase, NIMA-interacting 1
hsa-miR-200b-3p	CHD2	chromodomain helicase DNA binding protein 2
hsa-miR-200b-3p	MAP1LC3B	microtubule associated protein 1 light chain 3 beta
hsa-miR-200b-3p	HPS5	HPS5, biogenesis of lysosomal organelles complex 2 subunit 2
hsa-miR-200b-3p	TMCC1	transmembrane and coiled-coil domain family 1
hsa-miR-200b-3p	PLPPR1	phospholipid phosphatase related 1
hsa-miR-200b-3p	LYRM2	LYR motif containing 2
hsa-miR-200b-3p	FAM169A	family with sequence similarity 169 member A
hsa-miR-200b-3p	ITPR1	inositol 1,4,5-trisphosphate receptor type 1
hsa-miR-200b-3p	HEG1	heart development protein with EGF like domains 1
hsa-miR-200b-3p	CHST9	carbohydrate sulfotransferase 9
hsa-miR-200b-3p	DMRT2	doublesex and mab-3 related transcription factor 2
hsa-miR-200b-3p	PRKAR2B	protein kinase cAMP-dependent type II regulatory subunit beta
hsa-miR-200b-3p	TJP1	tight junction protein 1
hsa-miR-200b-3p	FAM218A	family with sequence similarity 218 member A
hsa-miR-200b-3p	IQSEC1	IQ motif and Sec7 domain 1
hsa-miR-200b-3p	HS6ST2	heparan sulfate 6-O-sulfotransferase 2
hsa-miR-200b-3p	MYCN	MYCN proto-oncogene, bHLH transcription factor
hsa-miR-200b-3p	LY75	lymphocyte antigen 75
hsa-miR-200b-3p	REV3L	REV3 like, DNA directed polymerase zeta catalytic subunit
hsa-miR-200b-3p	ARIH2	ariadne RBR E3 ubiquitin protein ligase 2
hsa-miR-200b-3p	CHM	CHM, Rab escort protein 1
hsa-miR-200b-3p	CSRNP3	cysteine and serine rich nuclear protein 3
hsa-miR-200b-3p	KCNK2	potassium two pore domain channel subfamily K member 2
hsa-miR-200b-3p	MARCH1	membrane associated ring-CH-type finger 1
hsa-miR-200b-3p	ZNF274	zinc finger protein 274
hsa-miR-200b-3p	NEGR1	neuronal growth regulator 1
hsa-miR-200b-3p	FCAR	Fc fragment of IgA receptor
hsa-miR-200b-3p	FAM3B	family with sequence similarity 3 member B
hsa-miR-200b-3p	CFLAR	CASP8 and FADD like apoptosis regulator
hsa-miR-200b-3p	PTBP1	polypyrimidine tract binding protein 1
hsa-miR-200b-3p	FAM107B	family with sequence similarity 107 member B
hsa-miR-200b-3p	LRP4	LDL receptor related protein 4
hsa-miR-200b-3p	F2RL2	coagulation factor II thrombin receptor like 2
hsa-miR-200b-3p	MTCP1	mature T cell proliferation 1
hsa-miR-200b-3p	OTUD6B	OTU domain containing 6B
hsa-miR-200b-3p	ZNF566	zinc finger protein 566
hsa-miR-200b-3p	CA5B	carbonic anhydrase 5B
hsa-miR-200b-3p	TRPV3	transient receptor potential cation channel subfamily V member 3
hsa-miR-200b-3p	DCAF5	DDB1 and CUL4 associated factor 5

hsa-miR-200b-3p	BASP1	brain abundant membrane attached signal protein 1
hsa-miR-200b-3p	GSKIP	GSK3B interacting protein
hsa-miR-200b-3p	TRPC3	transient receptor potential cation channel subfamily C member 3
hsa-miR-200b-3p	PRRC2C	proline rich coiled-coil 2C
hsa-miR-200b-3p	VPS13A	vacuolar protein sorting 13 homolog A
hsa-miR-200b-3p	AP1M1	adaptor related protein complex 1 subunit mu 1
hsa-miR-200b-3p	CNTD1	cyclin N-terminal domain containing 1
hsa-miR-200b-3p	SCOC	short coiled-coil protein
hsa-miR-200b-3p	BCLAF3	BCLAF1 and THRAP3 family member 3
hsa-miR-200b-3p	TM4SF18	transmembrane 4 L six family member 18
hsa-miR-200b-3p	SMAD5	SMAD family member 5
hsa-miR-200b-3p	GFI1	growth factor independent 1 transcriptional repressor
hsa-miR-200b-3p	ZNF365	zinc finger protein 365
hsa-miR-200b-3p	ATP11A	ATPase phospholipid transporting 11A
hsa-miR-200b-3p	NLRP11	NLR family pyrin domain containing 11
hsa-miR-200b-3p	CDK12	cyclin dependent kinase 12
hsa-miR-200b-3p	MUL1	mitochondrial E3 ubiquitin protein ligase 1
hsa-miR-200b-3p	WSB1	WD repeat and SOCS box containing 1
hsa-miR-200b-3p	PSMF1	proteasome inhibitor subunit 1
hsa-miR-200b-3p	CDHR1	cadherin related family member 1
hsa-miR-200b-3p	RIF1	replication timing regulatory factor 1
hsa-miR-200b-3p	HNRNPH2	heterogeneous nuclear ribonucleoprotein H2
hsa-miR-200b-3p	ZNF366	zinc finger protein 366
hsa-miR-200b-3p	NAA50	N(alpha)-acetyltransferase 50, NatE catalytic subunit
hsa-miR-200b-3p	SUB1	SUB1 homolog, transcriptional regulator
hsa-miR-200b-3p	C22orf39	chromosome 22 open reading frame 39
hsa-miR-200b-3p	SPIDR	scaffold protein involved in DNA repair
hsa-miR-200b-3p	NIP7	NIP7, nucleolar pre-rRNA processing protein
hsa-miR-200b-3p	TGFBR3	transforming growth factor beta receptor 3
hsa-miR-200b-3p	TMEM229B	transmembrane protein 229B
hsa-miR-200b-3p	UTP25	UTP25, small subunit processor component
hsa-miR-200b-3p	PI15	peptidase inhibitor 15
hsa-miR-200b-3p	IRS2	insulin receptor substrate 2
hsa-miR-200b-3p	ANKRD28	ankyrin repeat domain 28
hsa-miR-200b-3p	WDR45B	WD repeat domain 45B
hsa-miR-200b-3p	C21orf91	chromosome 21 open reading frame 91
hsa-miR-200b-3p	FGD1	FYVE, RhoGEF and PH domain containing 1
hsa-miR-200b-3p	FMR1	fragile X mental retardation 1
hsa-miR-200b-3p	NFASC	neurofascin
hsa-miR-200b-3p	RFC3	replication factor C subunit 3
hsa-miR-200b-3p	RNASEL	ribonuclease L
hsa-miR-200b-3p	OSR1	odd-skipped related transcription factor 1
hsa-miR-200b-3p	FRS2	fibroblast growth factor receptor substrate 2
hsa-miR-200b-3p	C2orf15	chromosome 2 open reading frame 15

hsa-miR-200b-3p	NR2C1	nuclear receptor subfamily 2 group C member 1
hsa-miR-200b-3p	ING5	inhibitor of growth family member 5
hsa-miR-200b-3p	PENK	proenkephalin
hsa-miR-200b-3p	SOCS6	suppressor of cytokine signaling 6
hsa-miR-200b-3p	SEPHS1	selenophosphate synthetase 1
hsa-miR-200b-3p	NEO1	neogenin 1
hsa-miR-200b-3p	TP53INP1	tumor protein p53 inducible nuclear protein 1
hsa-miR-200b-3p	TMEM43	transmembrane protein 43
hsa-miR-200b-3p	RBM8A	RNA binding motif protein 8A
hsa-miR-200b-3p	PLXNA4	plexin A4
hsa-miR-200b-3p	RBM46	RNA binding motif protein 46
hsa-miR-200b-3p	GRIP1	glutamate receptor interacting protein 1
hsa-miR-200b-3p	MTAP	methylthioadenosine phosphorylase
hsa-miR-200b-3p	NOC3L	NOC3 like DNA replication regulator
hsa-miR-200b-3p	GATA2	GATA binding protein 2
hsa-miR-200b-3p	EGLN3	egl-9 family hypoxia inducible factor 3
hsa-miR-200b-3p	RASGEF1B	RasGEF domain family member 1B
hsa-miR-200b-3p	HBS1L	HBS1 like translational GTPase
hsa-miR-200b-3p	RAB30	RAB30, member RAS oncogene family
hsa-miR-200b-3p	NR2C2	nuclear receptor subfamily 2 group C member 2
hsa-miR-200b-3p	SOD2	superoxide dismutase 2
hsa-miR-200b-3p	ZNF330	zinc finger protein 330
hsa-miR-200b-3p	TAF3	TATA-box binding protein associated factor 3
hsa-miR-200b-3p	ZNF260	zinc finger protein 260
hsa-miR-200b-3p	MLLT10	MLLT10, histone lysine methyltransferase DOT1L cofactor
hsa-miR-200b-3p	ITPRID2	ITPR interacting domain containing 2
hsa-miR-200b-3p	NPR3	natriuretic peptide receptor 3
hsa-miR-200b-3p	CRYZ	crystallin zeta
hsa-miR-200b-3p	ONECUT2	one cut homeobox 2
hsa-miR-200b-3p	YOD1	YOD1 deubiquitinase
hsa-miR-200b-3p	LRRTM3	leucine rich repeat transmembrane neuronal 3
hsa-miR-200b-3p	ARPP21	cAMP regulated phosphoprotein 21
hsa-miR-200b-3p	CDK1	cyclin dependent kinase 1
hsa-miR-200b-3p	NAA16	N(alpha)-acetyltransferase 16, NatA auxiliary subunit
hsa-miR-200b-3p	TMEM123	transmembrane protein 123
hsa-miR-200b-3p	DYRK2	dual specificity tyrosine phosphorylation regulated kinase 2
hsa-miR-200b-3p	ACTC1	actin, alpha, cardiac muscle 1
hsa-miR-200b-3p	FAM160B1	family with sequence similarity 160 member B1
hsa-miR-200b-3p	UFSP2	UFM1 specific peptidase 2
hsa-miR-200b-3p	ASPH	aspartate beta-hydroxylase
hsa-miR-200b-3p	FUBP1	far upstream element binding protein 1
hsa-miR-200b-3p	HIF1AN	hypoxia inducible factor 1 subunit alpha inhibitor
hsa-miR-200b-3p	IL24	interleukin 24

hsa-miR-200b-3p	DCX	doublecortin
hsa-miR-200b-3p	CGGBP1	CGG triplet repeat binding protein 1
hsa-miR-200b-3p	ST3GAL5	ST3 beta-galactoside alpha-2,3-sialyltransferase 5
hsa-miR-200b-3p	CLIP2	CAP-Gly domain containing linker protein 2
hsa-miR-200b-3p	CD58	CD58 molecule
hsa-miR-200b-3p	ZNF550	zinc finger protein 550
hsa-miR-200b-3p	CDK2	cyclin dependent kinase 2
hsa-miR-200b-3p	TADA2B	transcriptional adaptor 2B
hsa-miR-200b-3p	FBXL16	F-box and leucine rich repeat protein 16
hsa-miR-200b-3p	TPD52L1	TPD52 like 1
hsa-miR-200b-3p	GLS	glutaminase
hsa-miR-200b-3p	SIRT1	sirtuin 1
hsa-miR-200b-3p	SPART	spartin
hsa-miR-200b-3p	ATMIN	ATM interactor
hsa-miR-200b-3p	C2orf72	chromosome 2 open reading frame 72
hsa-miR-200b-3p	C2orf68	chromosome 2 open reading frame 68
hsa-miR-200b-3p	BUB1B-PAK6	BUB1B-PAK6 readthrough
hsa-miR-200b-3p	CLCF1	cardiotrophin like cytokine factor 1
hsa-miR-200b-3p	CSTF3	cleavage stimulation factor subunit 3
hsa-miR-200b-3p	ADIPOQ	adiponectin, C1Q and collagen domain containing
hsa-miR-200b-3p	CLCC1	chloride channel CLIC like 1
hsa-miR-200b-3p	BORCS7	BLOC-1 related complex subunit 7
hsa-miR-200b-3p	SERF2	small EDRK-rich factor 2
hsa-miR-200b-3p	NPAP1	nuclear pore associated protein 1
hsa-miR-200b-3p	TREM1	triggering receptor expressed on myeloid cells 1
hsa-miR-200b-3p	ASXL1	ASXL transcriptional regulator 1
hsa-miR-200b-3p	TRIM2	tripartite motif containing 2
hsa-miR-200b-3p	RPL36A-HNRNPH2	RPL36A-HNRNPH2 readthrough
hsa-miR-200b-3p	PRPF38A	pre-mRNA processing factor 38A
hsa-miR-200b-3p	KLHL14	kelch like family member 14
hsa-miR-200b-3p	RALGPS2	Ral GEF with PH domain and SH3 binding motif 2
hsa-miR-200b-3p	LGSN	lengsin, lens protein with glutamine synthetase domain
hsa-miR-200b-3p	PAN3	poly(A) specific ribonuclease subunit PAN3
hsa-miR-200b-3p	SGCB	sarcoglycan beta
hsa-miR-200b-3p	MEGF10	multiple EGF like domains 10
hsa-miR-200b-3p	SLC16A7	solute carrier family 16 member 7
hsa-miR-200b-3p	CCSER1	coiled-coil serine rich protein 1
hsa-miR-200b-3p	BBS12	Bardet-Biedl syndrome 12
hsa-miR-200b-3p	LRRC34	leucine rich repeat containing 34
hsa-miR-200b-3p	USF3	upstream transcription factor family member 3
hsa-miR-200b-3p	CASC4	cancer susceptibility 4
hsa-miR-200b-3p	ITSN1	intersectin 1
hsa-miR-200b-3p	ERBIN	erbb2 interacting protein
hsa-miR-200b-3p	XRN2	5'-3' exoribonuclease 2

hsa-miR-200b-3p	CTNNA3	catenin alpha 3
hsa-miR-200b-3p	CREB5	cAMP responsive element binding protein 5
hsa-miR-200b-3p	PPP1R12B	protein phosphatase 1 regulatory subunit 12B
hsa-miR-200b-3p	MDM4	MDM4, p53 regulator
hsa-miR-200b-3p	XKRY2	XK related, Y-linked 2
hsa-miR-200b-3p	RBMXL2	RBMX like 2
hsa-miR-200b-3p	TENM1	teneurin transmembrane protein 1
hsa-miR-200b-3p	BHLHE41	basic helix-loop-helix family member e41
hsa-miR-200b-3p	SNTB2	syntrophin beta 2
hsa-miR-200b-3p	ATAT1	alpha tubulin acetyltransferase 1
hsa-miR-200b-3p	SSH2	slingshot protein phosphatase 2
hsa-miR-200b-3p	XKRY	XK related, Y-linked
hsa-miR-200b-3p	STAM	signal transducing adaptor molecule
hsa-miR-200b-3p	KIF13A	kinesin family member 13A
hsa-miR-200b-3p	PIGM	phosphatidylinositol glycan anchor biosynthesis class M
hsa-miR-200b-3p	LSM8	LSM8 homolog, U6 small nuclear RNA associated
hsa-miR-200b-3p	ANKRD34B	ankyrin repeat domain 34B
hsa-miR-200b-3p	CFAP97	cilia and flagella associated protein 97
hsa-miR-200b-3p	CKLF	chemokine like factor
hsa-miR-200b-3p	RBBP4	RB binding protein 4, chromatin remodeling factor
hsa-miR-200b-3p	ALPI	alkaline phosphatase, intestinal
hsa-miR-200b-3p	TMEM26	transmembrane protein 26
hsa-miR-200b-3p	DARS	aspartyl-tRNA synthetase
hsa-miR-200b-3p	GLIS2	GLIS family zinc finger 2
hsa-miR-200b-3p	COL4A3	collagen type IV alpha 3 chain
hsa-miR-200b-3p	MOSPD2	motile sperm domain containing 2
hsa-miR-200b-3p	CA3	carbonic anhydrase 3
hsa-miR-200b-3p	TOR1AIP2	torsin 1A interacting protein 2
hsa-miR-200b-3p	TARDBP	TAR DNA binding protein
hsa-miR-200b-3p	LRIG1	leucine rich repeats and immunoglobulin like domains 1
hsa-miR-200b-3p	FAM217B	family with sequence similarity 217 member B
hsa-miR-200b-3p	WWP2	WW domain containing E3 ubiquitin protein ligase 2
hsa-miR-200b-3p	PM20D2	peptidase M20 domain containing 2
hsa-miR-200b-3p	CEBPD	CCAAT enhancer binding protein delta
hsa-miR-200b-3p	LRAT	lecithin retinol acyltransferase
hsa-miR-200b-3p	NALCN	sodium leak channel, non-selective
hsa-miR-200b-3p	NEK9	NIMA related kinase 9
hsa-miR-200b-3p	TNFSF8	TNF superfamily member 8
hsa-miR-200b-3p	RNGTT	RNA guanylyltransferase and 5'-phosphatase
hsa-miR-200b-3p	IGSF3	immunoglobulin superfamily member 3
hsa-miR-200b-3p	LDB3	LIM domain binding 3
hsa-miR-200b-3p	SNX1	sorting nexin 1
hsa-miR-200b-3p	KAT2B	lysine acetyltransferase 2B
hsa-miR-200b-3p	SCN9A	sodium voltage-gated channel alpha subunit 9

hsa-miR-200b-3p	SMARCAD1	SWI/SNF-related, matrix-associated actin-dependent regulator of chromatin, subfamily a, containing DEAD/H box 1
hsa-miR-200b-3p	GABPB1	GA binding protein transcription factor subunit beta 1
hsa-miR-200b-3p	NUP153	nucleoporin 153
hsa-miR-200b-3p	ABRA	actin binding Rho activating protein
hsa-miR-200b-3p	ERI1	exoribonuclease 1
hsa-miR-200b-3p	GABRA6	gamma-aminobutyric acid type A receptor alpha6 subunit
hsa-miR-200b-3p	TUBB3	tubulin beta 3 class III
hsa-miR-200b-3p	CPXCR1	CPX chromosome region, candidate 1
hsa-miR-200b-3p	EDNRA	endothelin receptor type A
hsa-miR-200b-3p	BDKRB2	bradykinin receptor B2
hsa-miR-200b-3p	SHISA2	shisa family member 2
hsa-miR-200b-3p	GPR85	G protein-coupled receptor 85
hsa-miR-200b-3p	TNFRSF11B	TNF receptor superfamily member 11b
hsa-miR-200b-3p	ZNF674	zinc finger protein 674
hsa-miR-200b-3p	PDPK1	3-phosphoinositide dependent protein kinase 1
hsa-miR-200b-3p	VTI1A	vesicle transport through interaction with t-SNAREs 1A
hsa-miR-200b-3p	SLC25A30	solute carrier family 25 member 30
hsa-miR-200b-3p	PARG	poly(ADP-ribose) glycohydrolase
hsa-miR-200b-3p	TMEM38B	transmembrane protein 38B
hsa-miR-200b-3p	TMEM14A	transmembrane protein 14A
hsa-miR-200b-3p	PIK3CB	phosphatidylinositol-4,5-bisphosphate 3-kinase catalytic subunit beta
hsa-miR-200b-3p	TMED4	transmembrane p24 trafficking protein 4
hsa-miR-200b-3p	HLF	HLF, PAR bZIP transcription factor
hsa-miR-200b-3p	SLC15A2	solute carrier family 15 member 2
hsa-miR-200b-3p	COL9A2	collagen type IX alpha 2 chain
hsa-miR-200b-3p	CREG1	cellular repressor of E1A stimulated genes 1
hsa-miR-200b-3p	TENT5D	terminal nucleotidyltransferase 5D
hsa-miR-200b-3p	AMACR	alpha-methylacyl-CoA racemase
hsa-miR-200b-3p	MMS22L	MMS22 like, DNA repair protein
hsa-miR-200b-3p	RWDD2A	RWD domain containing 2A
hsa-miR-200b-3p	RFX7	regulatory factor X7
hsa-miR-200b-3p	AMOTL2	angiomotin like 2
hsa-miR-200b-3p	DDX3Y	DEAD-box helicase 3 Y-linked
hsa-miR-200b-3p	KBTBD6	kelch repeat and BTB domain containing 6
hsa-miR-200b-3p	ZNF692	zinc finger protein 692
hsa-miR-200b-3p	ZNF831	zinc finger protein 831
hsa-miR-200b-3p	PJA2	praja ring finger ubiquitin ligase 2
hsa-miR-200b-3p	GPR63	G protein-coupled receptor 63
hsa-miR-200b-3p	PLEKHA8	pleckstrin homology domain containing A8
hsa-miR-200b-3p	TRAT1	T cell receptor associated transmembrane adaptor 1
hsa-miR-200b-3p	ARL6IP6	ADP ribosylation factor like GTPase 6 interacting protein 6

hsa-miR-200b-3p	STX16	syntaxin 16
hsa-miR-200b-3p	RELCH	RAB11 binding and LisH domain, coiled-coil and HEAT repeat containing
hsa-miR-200b-3p	DDX1	DEAD-box helicase 1
hsa-miR-200b-3p	SELENOK	selenoprotein K
hsa-miR-200b-3p	SNX30	sorting nexin family member 30
hsa-miR-200b-3p	DNAJC18	DnaJ heat shock protein family (Hsp40) member C18
hsa-miR-200b-3p	SHROOM4	shroom family member 4
hsa-miR-200b-3p	CDH6	cadherin 6
hsa-miR-200b-3p	ZNF568	zinc finger protein 568
hsa-miR-200b-3p	ARHGEF3	Rho guanine nucleotide exchange factor 3
hsa-miR-200b-3p	NAPB	NSF attachment protein beta
hsa-miR-200b-3p	RET	ret proto-oncogene
hsa-miR-200b-3p	RAD18	RAD18, E3 ubiquitin protein ligase
hsa-miR-200b-3p	HLA-DPA1	major histocompatibility complex, class II, DP alpha 1
hsa-miR-200b-3p	ADGRL2	adhesion G protein-coupled receptor L2
hsa-miR-200b-3p	SLC17A4	solute carrier family 17 member 4
hsa-miR-200b-3p	ELAVL4	ELAV like RNA binding protein 4
hsa-miR-200b-3p	ZBTB5	zinc finger and BTB domain containing 5
hsa-miR-200b-3p	ENAH	ENAH, actin regulator
hsa-miR-200b-3p	SLC25A24	solute carrier family 25 member 24
hsa-miR-200b-3p	ZMYM4	zinc finger MYM-type containing 4
hsa-miR-200b-3p	HNRNPU	heterogeneous nuclear ribonucleoprotein U
hsa-miR-200b-3p	CALHM5	calcium homeostasis modulator family member 5
hsa-miR-200b-3p	GDPD1	glycerophosphodiester phosphodiesterase domain containing 1
hsa-miR-200b-3p	CRHBP	corticotropin releasing hormone binding protein
hsa-miR-200b-3p	RBFA	ribosome binding factor A
hsa-miR-200b-3p	EPM2AIP1	EPM2A interacting protein 1
hsa-miR-200b-3p	MPPED1	metallophosphoesterase domain containing 1
hsa-miR-200b-3p	RCN1	reticulocalbin 1
hsa-miR-200b-3p	AK9	adenylate kinase 9
hsa-miR-200b-3p	UXS1	UDP-glucuronate decarboxylase 1
hsa-miR-200b-3p	LMAN1	lectin, mannose binding 1
hsa-miR-200b-3p	CCSAP	centriole, cilia and spindle associated protein
hsa-miR-200b-3p	KLF10	Kruppel like factor 10
hsa-miR-200b-3p	COPS2	COP9 signalosome subunit 2
hsa-miR-200b-3p	SLAIN1	SLAIN motif family member 1
hsa-miR-200b-3p	ZDHHC15	zinc finger DHHC-type containing 15
hsa-miR-200b-3p	SCHIP1	schwannomin interacting protein 1
hsa-miR-200b-3p	MRPS35	mitochondrial ribosomal protein S35
hsa-miR-200b-3p	CDC27	cell division cycle 27
hsa-miR-200b-3p	GOSR2	golgi SNAP receptor complex member 2
hsa-miR-200b-3p	IPMK	inositol polyphosphate multikinase
hsa-miR-200b-3p	STAC	SH3 and cysteine rich domain

hsa-miR-200b-3p	KLHL3	kelch like family member 3
hsa-miR-200b-3p	USP47	ubiquitin specific peptidase 47
hsa-miR-200b-3p	TP53TG3C	TP53 target 3C
hsa-miR-200b-3p	TTC5	tetratricopeptide repeat domain 5
hsa-miR-200b-3p	EIF2S1	eukaryotic translation initiation factor 2 subunit alpha
hsa-miR-200b-3p	NOL12	nucleolar protein 12
hsa-miR-200b-3p	GPC6	glypican 6
hsa-miR-200b-3p	GPX8	glutathione peroxidase 8 (putative)
hsa-miR-200b-3p	EXOSC3	exosome component 3
hsa-miR-200b-3p	GREM2	gremlin 2, DAN family BMP antagonist
hsa-miR-200b-3p	NSMCE3	NSE3 homolog, SMC5-SMC6 complex component
hsa-miR-200b-3p	HMOX1	heme oxygenase 1
hsa-miR-200b-3p	APLP2	amyloid beta precursor like protein 2
hsa-miR-200b-3p	ATF7IP	activating transcription factor 7 interacting protein
hsa-miR-200b-3p	CALHM1	calcium homeostasis modulator 1
hsa-miR-200b-3p	CLDND1	claudin domain containing 1
hsa-miR-200b-3p	TTC33	tetratricopeptide repeat domain 33
hsa-miR-200b-3p	AVPR1A	arginine vasopressin receptor 1A
hsa-miR-200b-3p	ASB13	ankyrin repeat and SOCS box containing 13
hsa-miR-200b-3p	SAMD12	sterile alpha motif domain containing 12
hsa-miR-200b-3p	ADD3	adducin 3
hsa-miR-200b-3p	IFIT5	interferon induced protein with tetratricopeptide repeats 5
hsa-miR-200b-3p	ENTPD5	ectonucleoside triphosphate diphosphohydrolase 5
hsa-miR-200b-3p	CCDC144A	coiled-coil domain containing 144A
hsa-miR-200b-3p	TTC21B	tetratricopeptide repeat domain 21B
hsa-miR-200b-3p	DGKE	diacylglycerol kinase epsilon
hsa-miR-200b-3p	JADE3	jade family PHD finger 3
hsa-miR-200b-3p	PTPN13	protein tyrosine phosphatase, non-receptor type 13
hsa-miR-200b-3p	NCBP3	nuclear cap binding subunit 3
hsa-miR-200b-3p	MYEOV	myeloma overexpressed
hsa-miR-200b-3p	RBM48	RNA binding motif protein 48
hsa-miR-200b-3p	UCHL5	ubiquitin C-terminal hydrolase L5
hsa-miR-200b-3p	ABI2	abl interactor 2
hsa-miR-200b-3p	DR1	down-regulator of transcription 1
hsa-miR-200b-3p	TAF5L	TATA-box binding protein associated factor 5 like
hsa-miR-200b-3p	NECTIN1	nectin cell adhesion molecule 1
hsa-miR-200b-3p	MAP2K5	mitogen-activated protein kinase kinase 5
hsa-miR-200b-3p	FGF9	fibroblast growth factor 9
hsa-miR-200b-3p	DNMT3A	DNA methyltransferase 3 alpha
hsa-miR-200b-3p	ZDBF2	zinc finger DBF-type containing 2
hsa-miR-200b-3p	CNDP2	carnosine dipeptidase 2
hsa-miR-200b-3p	RALB	RAS like proto-oncogene B
hsa-miR-200b-3p	RUFY2	RUN and FYVE domain containing 2
hsa-miR-200b-3p	REL	REL proto-oncogene, NF-kB subunit

hsa-miR-200b-3p	RHOA	ras homolog family member A
hsa-miR-200b-3p	SH3TC2	SH3 domain and tetratricopeptide repeats 2
hsa-miR-200b-3p	MPP5	membrane palmitoylated protein 5
hsa-miR-200b-3p	CCDC62	coiled-coil domain containing 62
hsa-miR-200b-3p	SLC5A3	solute carrier family 5 member 3
hsa-miR-200b-3p	PRKAA2	protein kinase AMP-activated catalytic subunit alpha 2
hsa-miR-200b-3p	HPF1	histone PARylation factor 1
hsa-miR-200b-3p	TC2N	tandem C2 domains, nuclear
hsa-miR-200b-3p	SMPX	small muscle protein X-linked
hsa-miR-200b-3p	NRK	Nik related kinase
hsa-miR-200b-3p	SLC16A1	solute carrier family 16 member 1
hsa-miR-200b-3p	MGA	MGA, MAX dimerization protein
hsa-miR-200c-3p	VASH2	vasohibin 2
hsa-miR-200c-3p	HIPK3	homeodomain interacting protein kinase 3
hsa-miR-200c-3p	MAP2	microtubule associated protein 2
hsa-miR-200c-3p	ERRFI1	ERBB receptor feedback inhibitor 1
hsa-miR-200c-3p	ZEB1	zinc finger E-box binding homeobox 1
hsa-miR-200c-3p	NR5A2	nuclear receptor subfamily 5 group A member 2
hsa-miR-200c-3p	ZEB2	zinc finger E-box binding homeobox 2
hsa-miR-200c-3p	RECK	reversion inducing cysteine rich protein with kazal motifs
hsa-miR-200c-3p	SLIT2	slit guidance ligand 2
hsa-miR-200c-3p	WIPF1	WAS/WASL interacting protein family member 1
hsa-miR-200c-3p	C11orf95	chromosome 11 open reading frame 95
hsa-miR-200c-3p	FAM8A1	family with sequence similarity 8 member A1
hsa-miR-200c-3p	LHFPL6	LHFPL tetraspan subfamily member 6
hsa-miR-200c-3p	AP1S2	adaptor related protein complex 1 subunit sigma 2
hsa-miR-200c-3p	SEC23A	Sec23 homolog A, coat complex II component
hsa-miR-200c-3p	TBX18	T-box 18
hsa-miR-200c-3p	PTPN21	protein tyrosine phosphatase, non-receptor type 21
hsa-miR-200c-3p	DNAJC3	DnaJ heat shock protein family (Hsp40) member C3
hsa-miR-200c-3p	QKI	QKI, KH domain containing RNA binding
hsa-miR-200c-3p	RIC1	RIC1 homolog, RAB6A GEF complex partner 1
hsa-miR-200c-3p	CCNJ	cyclin J
hsa-miR-200c-3p	PCMTD1	protein-L-isoaspartate (D-aspartate) O-methyltransferase domain containing 1
hsa-miR-200c-3p	CFL2	cofilin 2
hsa-miR-200c-3p	GPM6A	glycoprotein M6A
hsa-miR-200c-3p	ARHGAP6	Rho GTPase activating protein 6
hsa-miR-200c-3p	TFAP2A	transcription factor AP-2 alpha
hsa-miR-200c-3p	SLC35B4	solute carrier family 35 member B4
hsa-miR-200c-3p	BAP1	BRCA1 associated protein 1
hsa-miR-200c-3p	RAB11FIP2	RAB11 family interacting protein 2
hsa-miR-200c-3p	MSN	moesin
hsa-miR-200c-3p	FBXW7	F-box and WD repeat domain containing 7
hsa-miR-200c-3p	TRIM33	tripartite motif containing 33

hsa-miR-200c-3p	CNOT6	CCR4-NOT transcription complex subunit 6
hsa-miR-200c-3p	PRTG	protogenin
hsa-miR-200c-3p	RPS6KB1	ribosomal protein S6 kinase B1
hsa-miR-200c-3p	NOVA2	NOVA alternative splicing regulator 2
hsa-miR-200c-3p	WASF3	WAS protein family member 3
hsa-miR-200c-3p	KDM7A	lysine demethylase 7A
hsa-miR-200c-3p	ELL2	elongation factor for RNA polymerase II 2
hsa-miR-200c-3p	ATXN1	ataxin 1
hsa-miR-200c-3p	SYNJ1	synaptojanin 1
hsa-miR-200c-3p	MIEF1	mitochondrial elongation factor 1
hsa-miR-200c-3p	MBNL3	muscleblind like splicing regulator 3
hsa-miR-200c-3p	CSNK1G3	casein kinase 1 gamma 3
hsa-miR-200c-3p	KHDRBS1	KH RNA binding domain containing, signal transduction associated 1
hsa-miR-200c-3p	ELMOD2	ELMO domain containing 2
hsa-miR-200c-3p	MCFD2	multiple coagulation factor deficiency 2
hsa-miR-200c-3p	CRKL	CRK like proto-oncogene, adaptor protein
hsa-miR-200c-3p	TENT4B	terminal nucleotidyltransferase 4B
hsa-miR-200c-3p	MPRIP	myosin phosphatase Rho interacting protein
hsa-miR-200c-3p	PPP4R2	protein phosphatase 4 regulatory subunit 2
hsa-miR-200c-3p	MGAT2	mannosyl (alpha-1,6-)-glycoprotein beta-1,2-N-acetylglucosaminyltransferase
hsa-miR-200c-3p	FAM122C	family with sequence similarity 122C
hsa-miR-200c-3p	NFIA	nuclear factor I A
hsa-miR-200c-3p	SUSD5	sushi domain containing 5
hsa-miR-200c-3p	FEZ2	fasciculation and elongation protein zeta 2
hsa-miR-200c-3p	PRDM16	PR/SET domain 16
hsa-miR-200c-3p	LRP1B	LDL receptor related protein 1B
hsa-miR-200c-3p	PPP2R5E	protein phosphatase 2 regulatory subunit B'epsilon
hsa-miR-200c-3p	PTPN14	protein tyrosine phosphatase, non-receptor type 14
hsa-miR-200c-3p	OSTM1	osteoclastogenesis associated transmembrane protein 1
hsa-miR-200c-3p	FAM126B	family with sequence similarity 126 member B
hsa-miR-200c-3p	CDK17	cyclin dependent kinase 17
hsa-miR-200c-3p	FRMD6	FERM domain containing 6
hsa-miR-200c-3p	RBSN	rabenosyn, RAB effector
hsa-miR-200c-3p	JAKMIP2	janus kinase and microtubule interacting protein 2
hsa-miR-200c-3p	PIK3CA	phosphatidylinositol-4,5-bisphosphate 3-kinase catalytic subunit alpha
hsa-miR-200c-3p	CCDC177	coiled-coil domain containing 177
hsa-miR-200c-3p	ARIH1	ariadne RBR E3 ubiquitin protein ligase 1
hsa-miR-200c-3p	ATP11C	ATPase phospholipid transporting 11C
hsa-miR-200c-3p	RTF1	RTF1 homolog, Paf1/RNA polymerase II complex component
hsa-miR-200c-3p	PPM1F	protein phosphatase, Mg2+/Mn2+ dependent 1F
hsa-miR-200c-3p	RAP2C	RAP2C, member of RAS oncogene family
hsa-miR-200c-3p	MMD	monocyte to macrophage differentiation associated

hsa-miR-200c-3p	CHN2	chimerin 2
hsa-miR-200c-3p	PHF21B	PHD finger protein 21B
hsa-miR-200c-3p	SULF1	sulfatase 1
hsa-miR-200c-3p	ARL2BP	ADP ribosylation factor like GTPase 2 binding protein
hsa-miR-200c-3p	ADIPOR2	adiponectin receptor 2
hsa-miR-200c-3p	ZNF532	zinc finger protein 532
hsa-miR-200c-3p	PHACTR3	phosphatase and actin regulator 3
hsa-miR-200c-3p	RASA2	RAS p21 protein activator 2
hsa-miR-200c-3p	ZNF711	zinc finger protein 711
hsa-miR-200c-3p	SEMA6D	semaphorin 6D
hsa-miR-200c-3p	VLDLR	very low density lipoprotein receptor
hsa-miR-200c-3p	IGSF10	immunoglobulin superfamily member 10
hsa-miR-200c-3p	WNT16	Wnt family member 16
hsa-miR-200c-3p	CBL	Cbl proto-oncogene
hsa-miR-200c-3p	EPS8	epidermal growth factor receptor pathway substrate 8
hsa-miR-200c-3p	NANOS1	nanos C2HC-type zinc finger 1
hsa-miR-200c-3p	TMOD3	tropomodulin 3
hsa-miR-200c-3p	COL4A3BP	collagen type IV alpha 3 binding protein
hsa-miR-200c-3p	KDELC1	KDEL motif containing 1
hsa-miR-200c-3p	MINDY2	MINDY lysine 48 deubiquitinase 2
hsa-miR-200c-3p	RND3	Rho family GTPase 3
hsa-miR-200c-3p	KDR	kinase insert domain receptor
hsa-miR-200c-3p	OTUD4	OTU deubiquitinase 4
hsa-miR-200c-3p	NCOA2	nuclear receptor coactivator 2
hsa-miR-200c-3p	HMBOX1	homeobox containing 1
hsa-miR-200c-3p	DUSP1	dual specificity phosphatase 1
hsa-miR-200c-3p	ELOC	elongin C
hsa-miR-200c-3p	C16orf72	chromosome 16 open reading frame 72
hsa-miR-200c-3p	EGLN1	egl-9 family hypoxia inducible factor 1
hsa-miR-200c-3p	CLASP1	cytoplasmic linker associated protein 1
hsa-miR-200c-3p	ZFAND6	zinc finger AN1-type containing 6
hsa-miR-200c-3p	FOXG1	forkhead box G1
hsa-miR-200c-3p	KCNQ3	potassium voltage-gated channel subfamily Q member 3
hsa-miR-200c-3p	MBOAT2	membrane bound O-acyltransferase domain containing 2
hsa-miR-200c-3p	PHTF2	putative homeodomain transcription factor 2
hsa-miR-200c-3p	BRWD3	bromodomain and WD repeat domain containing 3
hsa-miR-200c-3p	NBR1	NBR1, autophagy cargo receptor
hsa-miR-200c-3p	ZNF131	zinc finger protein 131
hsa-miR-200c-3p	AFF3	AF4/FMR2 family member 3
hsa-miR-200c-3p	ARL5A	ADP ribosylation factor like GTPase 5A
hsa-miR-200c-3p	TMX4	thioredoxin related transmembrane protein 4
hsa-miR-200c-3p	IMMP2L	inner mitochondrial membrane peptidase subunit 2
hsa-miR-200c-3p	MAP3K1	mitogen-activated protein kinase kinase kinase 1
hsa-miR-200c-3p	SGIP1	SH3 domain GRB2 like endophilin interacting protein 1
hsa-miR-200c-3p	IPO7	importin 7

hsa-miR-200c-3p	GOLGA7	golgin A7
hsa-miR-200c-3p	DLC1	DLC1 Rho GTPase activating protein
hsa-miR-200c-3p	XKR8	XK related 8
hsa-miR-200c-3p	ELK4	ELK4, ETS transcription factor
hsa-miR-200c-3p	FN1	fibronectin 1
hsa-miR-200c-3p	SESN1	sestrin 1
hsa-miR-200c-3p	JUN	Jun proto-oncogene, AP-1 transcription factor subunit
hsa-miR-200c-3p	GPR158	G protein-coupled receptor 158
hsa-miR-200c-3p	DGKH	diacylglycerol kinase eta
hsa-miR-200c-3p	SYDE1	synapse defective Rho GTPase homolog 1
hsa-miR-200c-3p	PPHLN1	periphilin 1
hsa-miR-200c-3p	ZC3H6	zinc finger CCCH-type containing 6
hsa-miR-200c-3p	PRKG1	protein kinase cGMP-dependent 1
hsa-miR-200c-3p	SLC1A2	solute carrier family 1 member 2
hsa-miR-200c-3p	RANBP9	RAN binding protein 9
hsa-miR-200c-3p	GOLGA1	golgin A1
hsa-miR-200c-3p	LOX	lysyl oxidase
hsa-miR-200c-3p	GIT2	GIT ArfGAP 2
hsa-miR-200c-3p	ARMCX5-GPRASP2	ARMCX5-GPRASP2 readthrough
hsa-miR-200c-3p	MPDZ	multiple PDZ domain crumbs cell polarity complex component
hsa-miR-200c-3p	PGM2L1	phosphoglucomutase 2 like 1
hsa-miR-200c-3p	PRKACB	protein kinase cAMP-activated catalytic subunit beta
hsa-miR-200c-3p	CDH20	cadherin 20
hsa-miR-200c-3p	NTF3	neurotrophin 3
hsa-miR-200c-3p	B3GNT2	UDP-GlcNAc:betaGal beta-1,3-N-acetylglucosaminyltransferase 2
hsa-miR-200c-3p	PSAT1	phosphoserine aminotransferase 1
hsa-miR-200c-3p	REEP1	receptor accessory protein 1
hsa-miR-200c-3p	MED13	mediator complex subunit 13
hsa-miR-200c-3p	TBK1	TANK binding kinase 1
hsa-miR-200c-3p	TBC1D12	TBC1 domain family member 12
hsa-miR-200c-3p	MAPK7	mitogen-activated protein kinase 7
hsa-miR-200c-3p	WAPL	WAPL cohesin release factor
hsa-miR-200c-3p	VEGFA	vascular endothelial growth factor A
hsa-miR-200c-3p	RUSC2	RUN and SH3 domain containing 2
hsa-miR-200c-3p	SFXN1	sideroflexin 1
hsa-miR-200c-3p	ZFX	zinc finger protein X-linked
hsa-miR-200c-3p	HS2ST1	heparan sulfate 2-O-sulfotransferase 1
hsa-miR-200c-3p	BLCAP	BLCAP, apoptosis inducing factor
hsa-miR-200c-3p	RAB21	RAB21, member RAS oncogene family
hsa-miR-200c-3p	GPRASP2	G protein-coupled receptor associated sorting protein 2
hsa-miR-200c-3p	DESI1	desumoylating isopeptidase 1
hsa-miR-200c-3p	FOXF1	forkhead box F1

hsa-miR-200c-3p	PTPRZ1	protein tyrosine phosphatase, receptor type Z1
hsa-miR-200c-3p	TMEM17	transmembrane protein 17
hsa-miR-200c-3p	FSCN1	fascin actin-bundling protein 1
hsa-miR-200c-3p	PPP1R18	protein phosphatase 1 regulatory subunit 18
hsa-miR-200c-3p	RNF2	ring finger protein 2
hsa-miR-200c-3p	THAP1	THAP domain containing 1
hsa-miR-200c-3p	CLIC4	chloride intracellular channel 4
hsa-miR-200c-3p	CECR2	CECR2, histone acetyl-lysine reader
hsa-miR-200c-3p	HOOK1	hook microtubule tethering protein 1
hsa-miR-200c-3p	PPM1E	protein phosphatase, Mg2+/Mn2+ dependent 1E
hsa-miR-200c-3p	CHRDL1	chordin like 1
hsa-miR-200c-3p	DTNA	dystrobrevin alpha
hsa-miR-200c-3p	ZBTB10	zinc finger and BTB domain containing 10
hsa-miR-200c-3p	B3GLCT	beta 3-glucosyltransferase
hsa-miR-200c-3p	DNAJB9	DnaJ heat shock protein family (Hsp40) member B9
hsa-miR-200c-3p	FAT3	FAT atypical cadherin 3
hsa-miR-200c-3p	NR3C1	nuclear receptor subfamily 3 group C member 1
hsa-miR-200c-3p	FAM227B	family with sequence similarity 227 member B
hsa-miR-200c-3p	CSMD3	CUB and Sushi multiple domains 3
hsa-miR-200c-3p	PPFIA1	PTPRF interacting protein alpha 1
hsa-miR-200c-3p	KCTD8	potassium channel tetramerization domain containing 8
hsa-miR-200c-3p	PI4K2B	phosphatidylinositol 4-kinase type 2 beta
hsa-miR-200c-3p	ANKRD40	ankyrin repeat domain 40
hsa-miR-200c-3p	VASH1	vasohibin 1
hsa-miR-200c-3p	PDS5B	PDS5 cohesin associated factor B
hsa-miR-200c-3p	SLK	STE20 like kinase
hsa-miR-200c-3p	RTKN2	rhotekin 2
hsa-miR-200c-3p	RNF19A	ring finger protein 19A, RBR E3 ubiquitin protein ligase
hsa-miR-200c-3p	FHL1	four and a half LIM domains 1
hsa-miR-200c-3p	SPAG9	sperm associated antigen 9
hsa-miR-200c-3p	DPY19L1	dpy-19 like C-mannosyltransferase 1
hsa-miR-200c-3p	AMFR	autocrine motility factor receptor
hsa-miR-200c-3p	ELAVL2	ELAV like RNA binding protein 2
hsa-miR-200c-3p	LBR	lamin B receptor
hsa-miR-200c-3p	ZYG11B	zyg-11 family member B, cell cycle regulator
hsa-miR-200c-3p	CLIP1	CAP-Gly domain containing linker protein 1
hsa-miR-200c-3p	MOSMO	modulator of smoothened
hsa-miR-200c-3p	WDR82	WD repeat domain 82
hsa-miR-200c-3p	SERPINI1	serpin family I member 1
hsa-miR-200c-3p	RASSF8	Ras association domain family member 8
hsa-miR-200c-3p	JAZF1	JAZF zinc finger 1
hsa-miR-200c-3p	TOB1	transducer of ERBB2, 1
hsa-miR-200c-3p	EVI5	ecotropic viral integration site 5
hsa-miR-200c-3p	B4GAT1	beta-1,4-glucuronyltransferase 1
hsa-miR-200c-3p	POLK	DNA polymerase kappa

hsa-miR-200c-3p	CHRM2	cholinergic receptor muscarinic 2
hsa-miR-200c-3p	TRAPPC8	trafficking protein particle complex 8
hsa-miR-200c-3p	PUM2	pumilio RNA binding family member 2
hsa-miR-200c-3p	CDYL	chromodomain Y like
hsa-miR-200c-3p	YWHAG	tyrosine 3-monooxygenase/tryptophan 5-monooxygenase activation protein gamma
hsa-miR-200c-3p	SLC6A11	solute carrier family 6 member 11
hsa-miR-200c-3p	TCAIM	T cell activation inhibitor, mitochondrial
hsa-miR-200c-3p	PKD1	polycystin 1, transient receptor potential channel interacting
hsa-miR-200c-3p	FBXO30	F-box protein 30
hsa-miR-200c-3p	DNMT3B	DNA methyltransferase 3 beta
hsa-miR-200c-3p	FBXO33	F-box protein 33
hsa-miR-200c-3p	ITGA1	integrin subunit alpha 1
hsa-miR-200c-3p	OCLN	occludin
hsa-miR-200c-3p	CEP85L	centrosomal protein 85 like
hsa-miR-200c-3p	VAT1L	vesicle amine transport 1 like
hsa-miR-200c-3p	SCAMP1	secretory carrier membrane protein 1
hsa-miR-200c-3p	GABBR2	gamma-aminobutyric acid type B receptor subunit 2
hsa-miR-200c-3p	CAB39	calcium binding protein 39
hsa-miR-200c-3p	ADAMTS3	ADAM metallopeptidase with thrombospondin type 1 motif 3
hsa-miR-200c-3p	SCAI	suppressor of cancer cell invasion
hsa-miR-200c-3p	NEDD1	neural precursor cell expressed, developmentally down-regulated 1
hsa-miR-200c-3p	LRRC8A	leucine rich repeat containing 8 VRAC subunit A
hsa-miR-200c-3p	ANKRD44	ankyrin repeat domain 44
hsa-miR-200c-3p	NRBP1	nuclear receptor binding protein 1
hsa-miR-200c-3p	S100PBP	S100P binding protein
hsa-miR-200c-3p	USP25	ubiquitin specific peptidase 25
hsa-miR-200c-3p	MAP4K5	mitogen-activated protein kinase kinase kinase kinase 5
hsa-miR-200c-3p	CAMSAP2	calmodulin regulated spectrin associated protein family member 2
hsa-miR-200c-3p	HDAC9	histone deacetylase 9
hsa-miR-200c-3p	PPP1R9B	protein phosphatase 1 regulatory subunit 9B
hsa-miR-200c-3p	PMAIP1	phorbol-12-myristate-13-acetate-induced protein 1
hsa-miR-200c-3p	MAP4K4	mitogen-activated protein kinase kinase kinase kinase 4
hsa-miR-200c-3p	FSD1L	fibronectin type III and SPRY domain containing 1 like
hsa-miR-200c-3p	STK4	serine/threonine kinase 4
hsa-miR-200c-3p	TSC22D1	TSC22 domain family member 1
hsa-miR-200c-3p	PSIP1	PC4 and SFRS1 interacting protein 1
hsa-miR-200c-3p	MFAP5	microfibril associated protein 5
hsa-miR-200c-3p	SLC4A7	solute carrier family 4 member 7
hsa-miR-200c-3p	NOG	noggin
hsa-miR-200c-3p	SLC6A1	solute carrier family 6 member 1
hsa-miR-200c-3p	MIB1	mindbomb E3 ubiquitin protein ligase 1

hsa-miR-200c-3p	DCBLD2	discoidin, CUB and LCCL domain containing 2
hsa-miR-200c-3p	DENND5B	DENN domain containing 5B
hsa-miR-200c-3p	CCNYL1	cyclin Y like 1
hsa-miR-200c-3p	CKAP4	cytoskeleton associated protein 4
hsa-miR-200c-3p	STRN	striatin
hsa-miR-200c-3p	MARCH6	membrane associated ring-CH-type finger 6
hsa-miR-200c-3p	TMEFF2	transmembrane protein with EGF like and two follistatin like domains 2
hsa-miR-200c-3p	GAS2L3	growth arrest specific 2 like 3
hsa-miR-200c-3p	TLN2	talin 2
hsa-miR-200c-3p	HSPA13	heat shock protein family A (Hsp70) member 13
hsa-miR-200c-3p	SLC14A1	solute carrier family 14 member 1 (Kidd blood group)
hsa-miR-200c-3p	PAG1	phosphoprotein membrane anchor with glycosphingolipid microdomains 1
hsa-miR-200c-3p	NTRK2	neurotrophic receptor tyrosine kinase 2
hsa-miR-200c-3p	PIKFYVE	phosphoinositide kinase, FYVE-type zinc finger containing
hsa-miR-200c-3p	COPS8	COP9 signalosome subunit 8
hsa-miR-200c-3p	USP27X	ubiquitin specific peptidase 27 X-linked
hsa-miR-200c-3p	KYNU	kynureninase
hsa-miR-200c-3p	TSSK1B	testis specific serine kinase 1B
hsa-miR-200c-3p	PTPN12	protein tyrosine phosphatase, non-receptor type 12
hsa-miR-200c-3p	MARCKS	myristoylated alanine rich protein kinase C substrate
hsa-miR-200c-3p	AGFG1	ArfGAP with FG repeats 1
hsa-miR-200c-3p	CBX4	chromobox 4
hsa-miR-200c-3p	CNOT9	CCR4-NOT transcription complex subunit 9
hsa-miR-200c-3p	ATP5F1B	ATP synthase F1 subunit beta
hsa-miR-200c-3p	ZKSCAN8	zinc finger with KRAB and SCAN domains 8
hsa-miR-200c-3p	THAP2	THAP domain containing 2
hsa-miR-200c-3p	TRMT9B	tRNA methyltransferase 9B (putative)
hsa-miR-200c-3p	DIXDC1	DIX domain containing 1
hsa-miR-200c-3p	NOVA1	NOVA alternative splicing regulator 1
hsa-miR-200c-3p	DGKA	diacylglycerol kinase alpha
hsa-miR-200c-3p	ZNF217	zinc finger protein 217
hsa-miR-200c-3p	PCDH19	protocadherin 19
hsa-miR-200c-3p	SCN5A	sodium voltage-gated channel alpha subunit 5
hsa-miR-200c-3p	HS3ST1	heparan sulfate-glucosamine 3-sulfotransferase 1
hsa-miR-200c-3p	PITPNM3	PITPNM family member 3
hsa-miR-200c-3p	GXYLT1	glucoside xylosyltransferase 1
hsa-miR-200c-3p	PIM2	Pim-2 proto-oncogene, serine/threonine kinase
hsa-miR-200c-3p	INTS8	integrator complex subunit 8
hsa-miR-200c-3p	CRH	corticotropin releasing hormone
hsa-miR-200c-3p	RIMS2	regulating synaptic membrane exocytosis 2
hsa-miR-200c-3p	GLI3	GLI family zinc finger 3
hsa-miR-200c-3p	TOGARAM1	TOG array regulator of axonemal microtubules 1
hsa-miR-200c-3p	YPEL2	yippee like 2

hsa-miR-200c-3p	JCAD	junctional cadherin 5 associated
hsa-miR-200c-3p	HDHD2	haloacid dehalogenase like hydrolase domain containing 2
hsa-miR-200c-3p	ENO4	enolase 4
hsa-miR-200c-3p	DENND5A	DENN domain containing 5A
hsa-miR-200c-3p	TIMP2	TIMP metallopeptidase inhibitor 2
hsa-miR-200c-3p	SESN3	sestrin 3
hsa-miR-200c-3p	USP6NL	USP6 N-terminal like
hsa-miR-200c-3p	ERG	ETS transcription factor ERG
hsa-miR-200c-3p	CEP350	centrosomal protein 350
hsa-miR-200c-3p	ZNF326	zinc finger protein 326
hsa-miR-200c-3p	OSBPL11	oxysterol binding protein like 11
hsa-miR-200c-3p	NAP1L5	nucleosome assembly protein 1 like 5
hsa-miR-200c-3p	RBFOX2	RNA binding fox-1 homolog 2
hsa-miR-200c-3p	CYTH3	cytohesin 3
hsa-miR-200c-3p	DPH6	diphthamine biosynthesis 6
hsa-miR-200c-3p	MATR3	matrin 3
hsa-miR-200c-3p	DNA2	DNA replication helicase/nuclease 2
hsa-miR-200c-3p	ATL2	atlastin GTPase 2
hsa-miR-200c-3p	MAPRE1	microtubule associated protein RP/EB family member 1
hsa-miR-200c-3p	ROCK2	Rho associated coiled-coil containing protein kinase 2
hsa-miR-200c-3p	PROK2	prokineticin 2
hsa-miR-200c-3p	DNAJB5	DnaJ heat shock protein family (Hsp40) member B5
hsa-miR-200c-3p	ESRRG	estrogen related receptor gamma
hsa-miR-200c-3p	GNAQ	G protein subunit alpha q
hsa-miR-200c-3p	MAP4K3	mitogen-activated protein kinase kinase kinase kinase 3
hsa-miR-200c-3p	CNEP1R1	CTD nuclear envelope phosphatase 1 regulatory subunit 1
hsa-miR-200c-3p	RIPK2	receptor interacting serine/threonine kinase 2
hsa-miR-200c-3p	ZBTB38	zinc finger and BTB domain containing 38
hsa-miR-200c-3p	FIGNL2	fidgetin like 2
hsa-miR-200c-3p	ATAD2B	ATPase family, AAA domain containing 2B
hsa-miR-200c-3p	FOXN2	forkhead box N2
hsa-miR-200c-3p	ETS1	ETS proto-oncogene 1, transcription factor
hsa-miR-200c-3p	AKAP7	A-kinase anchoring protein 7
hsa-miR-200c-3p	PLPP3	phospholipid phosphatase 3
hsa-miR-200c-3p	SRGAP1	SLIT-ROBO Rho GTPase activating protein 1
hsa-miR-200c-3p	HECTD2	HECT domain E3 ubiquitin protein ligase 2
hsa-miR-200c-3p	ELK3	ELK3, ETS transcription factor
hsa-miR-200c-3p	CEP41	centrosomal protein 41
hsa-miR-200c-3p	CNKSR3	CNKSR family member 3
hsa-miR-200c-3p	ULK2	unc-51 like autophagy activating kinase 2
hsa-miR-200c-3p	ZFPM2	zinc finger protein, FOG family member 2
hsa-miR-200c-3p	ZMAT3	zinc finger matrin-type 3
hsa-miR-200c-3p	RDH10	retinol dehydrogenase 10

hsa-miR-200c-3p	SLC25A36	solute carrier family 25 member 36
hsa-miR-200c-3p	CCDC82	coiled-coil domain containing 82
hsa-miR-200c-3p	SLC30A7	solute carrier family 30 member 7
hsa-miR-200c-3p	SCD	stearoyl-CoA desaturase
hsa-miR-200c-3p	XKR4	XK related 4
hsa-miR-200c-3p	ZCCHC24	zinc finger CCHC-type containing 24
hsa-miR-200c-3p	CNTN1	contactin 1
hsa-miR-200c-3p	SMARCD1	SWI/SNF related, matrix associated, actin dependent regulator of chromatin, subfamily d, member 1
hsa-miR-200c-3p	NPM1	nucleophosmin 1
hsa-miR-200c-3p	ZSWIM4	zinc finger SWIM-type containing 4
hsa-miR-200c-3p	NCS1	neuronal calcium sensor 1
hsa-miR-200c-3p	RO60	Ro60, Y RNA binding protein
hsa-miR-200c-3p	PTAR1	protein prenyltransferase alpha subunit repeat containing 1
hsa-miR-200c-3p	PLCL1	phospholipase C like 1 (inactive)
hsa-miR-200c-3p	CYTH1	cytohesin 1
hsa-miR-200c-3p	TRHDE	thyrotropin releasing hormone degrading enzyme
hsa-miR-200c-3p	BCL11B	BCL11B, BAF complex component
hsa-miR-200c-3p	EDEM3	ER degradation enhancing alpha-mannosidase like protein 3
hsa-miR-200c-3p	FLII	FLII, actin remodeling protein
hsa-miR-200c-3p	KRT80	keratin 80
hsa-miR-200c-3p	EIF2B5	eukaryotic translation initiation factor 2B subunit epsilon
hsa-miR-200c-3p	FERMT2	fermitin family member 2
hsa-miR-200c-3p	SERINC1	serine incorporator 1
hsa-miR-200c-3p	ASAP1	ArfGAP with SH3 domain, ankyrin repeat and PH domain 1
hsa-miR-200c-3p	RANBP10	RAN binding protein 10
hsa-miR-200c-3p	RBFOX3	RNA binding fox-1 homolog 3
hsa-miR-200c-3p	HMGB3	high mobility group box 3
hsa-miR-200c-3p	GJC1	gap junction protein gamma 1
hsa-miR-200c-3p	CORO1C	coronin 1C
hsa-miR-200c-3p	HOOK3	hook microtubule tethering protein 3
hsa-miR-200c-3p	TAF12	TATA-box binding protein associated factor 12
hsa-miR-200c-3p	MARF1	meiosis regulator and mRNA stability factor 1
hsa-miR-200c-3p	NCK2	NCK adaptor protein 2
hsa-miR-200c-3p	TSC22D2	TSC22 domain family member 2
hsa-miR-200c-3p	CPED1	cadherin like and PC-esterase domain containing 1
hsa-miR-200c-3p	TBL1XR1	transducin beta like 1 X-linked receptor 1
hsa-miR-200c-3p	LRP1	LDL receptor related protein 1
hsa-miR-200c-3p	FARP1	FERM, ARH/RhoGEF and pleckstrin domain protein 1
hsa-miR-200c-3p	WWC3	WWC family member 3
hsa-miR-200c-3p	BNC2	basonuclin 2
hsa-miR-200c-3p	PHEX	phosphate regulating endopeptidase homolog X-linked
hsa-miR-200c-3p	PKIA	cAMP-dependent protein kinase inhibitor alpha

hsa-miR-200c-3p	RAP1B	RAP1B, member of RAS oncogene family
hsa-miR-200c-3p	PTHLH	parathyroid hormone like hormone
hsa-miR-200c-3p	UHRF1BP1	UHRF1 binding protein 1
hsa-miR-200c-3p	GPR180	G protein-coupled receptor 180
hsa-miR-200c-3p	C11orf87	chromosome 11 open reading frame 87
hsa-miR-200c-3p	CLASP2	cytoplasmic linker associated protein 2
hsa-miR-200c-3p	KLF4	Kruppel like factor 4
hsa-miR-200c-3p	SCRT2	scratch family transcriptional repressor 2
hsa-miR-200c-3p	RPS6KA3	ribosomal protein S6 kinase A3
hsa-miR-200c-3p	GPATCH8	G-patch domain containing 8
hsa-miR-200c-3p	GEM	GTP binding protein overexpressed in skeletal muscle
hsa-miR-200c-3p	ZDHHC21	zinc finger DHHC-type containing 21
hsa-miR-200c-3p	EXOG	exo/endonuclease G
hsa-miR-200c-3p	SGCE	sarcoglycan epsilon
hsa-miR-200c-3p	SLITRK1	SLIT and NTRK like family member 1
hsa-miR-200c-3p	WASF1	WAS protein family member 1
hsa-miR-200c-3p	INSM2	INSM transcriptional repressor 2
hsa-miR-200c-3p	FLI1	Fli-1 proto-oncogene, ETS transcription factor
hsa-miR-200c-3p	FAM118B	family with sequence similarity 118 member B
hsa-miR-200c-3p	ERICH4	glutamate rich 4
hsa-miR-200c-3p	SIX1	SIX homeobox 1
hsa-miR-200c-3p	TMEM136	transmembrane protein 136
hsa-miR-200c-3p	GTPBP10	GTP binding protein 10
hsa-miR-200c-3p	CHRNA6	cholinergic receptor nicotinic alpha 6 subunit
hsa-miR-200c-3p	EXD2	exonuclease 3'-5' domain containing 2
hsa-miR-200c-3p	TLN1	talin 1
hsa-miR-200c-3p	SNRPB2	small nuclear ribonucleoprotein polypeptide B2
hsa-miR-200c-3p	ZC3H4	zinc finger CCCH-type containing 4
hsa-miR-200c-3p	PI4KB	phosphatidylinositol 4-kinase beta
hsa-miR-200c-3p	SLC39A14	solute carrier family 39 member 14
hsa-miR-200c-3p	PPP2R1B	protein phosphatase 2 scaffold subunit Abeta
hsa-miR-200c-3p	NUDT4	nudix hydrolase 4
hsa-miR-200c-3p	PDIK1L	PDLIM1 interacting kinase 1 like
hsa-miR-200c-3p	UBE2R2	ubiquitin conjugating enzyme E2 R2
hsa-miR-200c-3p	CDYL2	chromodomain Y like 2
hsa-miR-200c-3p	FIGN	fidgetin, microtubule severing factor
hsa-miR-200c-3p	LIN7A	lin-7 homolog A, crumbs cell polarity complex component
hsa-miR-200c-3p	RRP15	ribosomal RNA processing 15 homolog
hsa-miR-200c-3p	HIPK1	homeodomain interacting protein kinase 1
hsa-miR-200c-3p	SRI	sorcin
hsa-miR-200c-3p	JKAMP	JNK1/MAPK8 associated membrane protein
hsa-miR-200c-3p	SDC2	syndecan 2
hsa-miR-200c-3p	PTBP3	polypyrimidine tract binding protein 3
hsa-miR-200c-3p	CUX1	cut like homeobox 1
hsa-miR-200c-3p	DACH1	dachshund family transcription factor 1

hsa-miR-200c-3p	PPP1R10	protein phosphatase 1 regulatory subunit 10
hsa-miR-200c-3p	RDX	radixin
hsa-miR-200c-3p	SLC35E2B	solute carrier family 35 member E2B
hsa-miR-200c-3p	YWHAB	tyrosine 3-monooxygenase/tryptophan 5-monooxygenase activation protein beta
hsa-miR-200c-3p	DDIT4L	DNA damage inducible transcript 4 like
hsa-miR-200c-3p	SYVN1	synoviolin 1
hsa-miR-200c-3p	DZIP1	DAZ interacting zinc finger protein 1
hsa-miR-200c-3p	PPM1B	protein phosphatase, Mg2+/Mn2+ dependent 1B
hsa-miR-200c-3p	SOX2	SRY-box 2
hsa-miR-200c-3p	GLCCI1	glucocorticoid induced 1
hsa-miR-200c-3p	BDP1	B double prime 1, subunit of RNA polymerase III transcription initiation factor IIIB
hsa-miR-200c-3p	STRADB	STE20 related adaptor beta
hsa-miR-200c-3p	HS3ST3A1	heparan sulfate-glucosamine 3-sulfotransferase 3A1
hsa-miR-200c-3p	KIAA0355	KIAA0355
hsa-miR-200c-3p	SLF2	SMC5-SMC6 complex localization factor 2
hsa-miR-200c-3p	ARHGAP20	Rho GTPase activating protein 20
hsa-miR-200c-3p	IKZF2	IKAROS family zinc finger 2
hsa-miR-200c-3p	SLC35E2A	solute carrier family 35 member E2A
hsa-miR-200c-3p	APOO	apolipoprotein O
hsa-miR-200c-3p	SLC24A4	solute carrier family 24 member 4
hsa-miR-200c-3p	FSTL1	follistatin like 1
hsa-miR-200c-3p	CASZ1	castor zinc finger 1
hsa-miR-200c-3p	CTDSPL2	CTD small phosphatase like 2
hsa-miR-200c-3p	PCLAF	PCNA clamp associated factor
hsa-miR-200c-3p	FNDC3B	fibronectin type III domain containing 3B
hsa-miR-200c-3p	A1CF	APOBEC1 complementation factor
hsa-miR-200c-3p	EIF4E2	eukaryotic translation initiation factor 4E family member 2
hsa-miR-200c-3p	NECTIN4	nectin cell adhesion molecule 4
hsa-miR-200c-3p	PPP1R9A	protein phosphatase 1 regulatory subunit 9A
hsa-miR-200c-3p	SNX16	sorting nexin 16
hsa-miR-200c-3p	GTF3C4	general transcription factor IIIC subunit 4
hsa-miR-200c-3p	RGL1	ral guanine nucleotide dissociation stimulator like 1
hsa-miR-200c-3p	SUZ12	SUZ12, polycomb repressive complex 2 subunit
hsa-miR-200c-3p	CHSY1	chondroitin sulfate synthase 1
hsa-miR-200c-3p	KANK2	KN motif and ankyrin repeat domains 2
hsa-miR-200c-3p	GSTA4	glutathione S-transferase alpha 4
hsa-miR-200c-3p	CLVS2	clavesin 2
hsa-miR-200c-3p	NPC1	NPC intracellular cholesterol transporter 1
hsa-miR-200c-3p	PLXNC1	plexin C1
hsa-miR-200c-3p	ARID4B	AT-rich interaction domain 4B
hsa-miR-200c-3p	TUBB	tubulin beta class I
hsa-miR-200c-3p	NAB1	NGFI-A binding protein 1
hsa-miR-200c-3p	MBNL2	muscleblind like splicing regulator 2

hsa-miR-200c-3p	BICC1	BicC family RNA binding protein 1
hsa-miR-200c-3p	CEP97	centrosomal protein 97
hsa-miR-200c-3p	ANK3	ankyrin 3
hsa-miR-200c-3p	MYZAP	myocardial zonula adherens protein
hsa-miR-200c-3p	DPY19L3	dpy-19 like C-mannosyltransferase 3
hsa-miR-200c-3p	SEMA3F	semaphorin 3F
hsa-miR-200c-3p	IGF2R	insulin like growth factor 2 receptor
hsa-miR-200c-3p	TAP2	transporter 2, ATP binding cassette subfamily B member
hsa-miR-200c-3p	LEPR	leptin receptor
hsa-miR-200c-3p	CASR	calcium sensing receptor
hsa-miR-200c-3p	DCUN1D5	defective in cullin neddylation 1 domain containing 5
hsa-miR-200c-3p	HNRNPD	heterogeneous nuclear ribonucleoprotein D
hsa-miR-200c-3p	DENND1B	DENN domain containing 1B
hsa-miR-200c-3p	MTF2	metal response element binding transcription factor 2
hsa-miR-200c-3p	UBE2W	ubiquitin conjugating enzyme E2 W
hsa-miR-200c-3p	TBCA	tubulin folding cofactor A
hsa-miR-200c-3p	MYB	MYB proto-oncogene, transcription factor
hsa-miR-200c-3p	PLPPR4	phospholipid phosphatase related 4
hsa-miR-200c-3p	TRIM23	tripartite motif containing 23
hsa-miR-200c-3p	IER5	immediate early response 5
hsa-miR-200c-3p	CCNE2	cyclin E2
hsa-miR-200c-3p	CRTAP	cartilage associated protein
hsa-miR-200c-3p	NUFIP2	nuclear FMR1 interacting protein 2
hsa-miR-200c-3p	SECISBP2L	SECIS binding protein 2 like
hsa-miR-200c-3p	NOTCH1	notch 1
hsa-miR-200c-3p	PRDM1	PR/SET domain 1
hsa-miR-200c-3p	ALDH1A1	aldehyde dehydrogenase 1 family member A1
hsa-miR-200c-3p	DNAJB14	DnaJ heat shock protein family (Hsp40) member B14
hsa-miR-200c-3p	GATA4	GATA binding protein 4
hsa-miR-200c-3p	KIF14	kinesin family member 14
hsa-miR-200c-3p	NYAP1	neuronal tyrosine phosphorylated phosphoinositide-3-kinase adaptor 1
hsa-miR-200c-3p	SCN8A	sodium voltage-gated channel alpha subunit 8
hsa-miR-200c-3p	ATP6V0A2	ATPase H+ transporting V0 subunit a2
hsa-miR-200c-3p	NRG1	neuregulin 1
hsa-miR-200c-3p	ACVR1C	activin A receptor type 1C
hsa-miR-200c-3p	MTSS1L	MTSS1L, I-BAR domain containing
hsa-miR-200c-3p	ADH1B	alcohol dehydrogenase 1B (class I), beta polypeptide
hsa-miR-200c-3p	GUCY1A1	guanylate cyclase 1 soluble subunit alpha 1
hsa-miR-200c-3p	SBSPON	somatomedin B and thrombospondin type 1 domain containing
hsa-miR-200c-3p	PICALM	phosphatidylinositol binding clathrin assembly protein
hsa-miR-200c-3p	KLF6	Kruppel like factor 6
hsa-miR-200c-3p	CHMP5	charged multivesicular body protein 5
hsa-miR-200c-3p	EFNB2	ephrin B2

hsa-miR-200c-3p	MEX3D	mex-3 RNA binding family member D
hsa-miR-200c-3p	KCND2	potassium voltage-gated channel subfamily D member 2
hsa-miR-200c-3p	SIKE1	suppressor of IKBKE 1
hsa-miR-200c-3p	MSL2	MSL complex subunit 2
hsa-miR-200c-3p	ZNF224	zinc finger protein 224
hsa-miR-200c-3p	FRMD4B	FERM domain containing 4B
hsa-miR-200c-3p	OXR1	oxidation resistance 1
hsa-miR-200c-3p	CDH11	cadherin 11
hsa-miR-200c-3p	IPO8	importin 8
hsa-miR-200c-3p	BPTF	bromodomain PHD finger transcription factor
hsa-miR-200c-3p	RSPRY1	ring finger and SPRY domain containing 1
hsa-miR-200c-3p	NDN	necdin, MAGE family member
hsa-miR-200c-3p	THSD7A	thrombospondin type 1 domain containing 7A
hsa-miR-200c-3p	MMGT1	membrane magnesium transporter 1
hsa-miR-200c-3p	STX17	syntaxin 17
hsa-miR-200c-3p	PHF6	PHD finger protein 6
hsa-miR-200c-3p	ARHGEF17	Rho guanine nucleotide exchange factor 17
hsa-miR-200c-3p	RABIF	RAB interacting factor
hsa-miR-200c-3p	ETV5	ETS variant 5
hsa-miR-200c-3p	CADM1	cell adhesion molecule 1
hsa-miR-200c-3p	TMBIM4	transmembrane BAX inhibitor motif containing 4
hsa-miR-200c-3p	ANLN	anillin actin binding protein
hsa-miR-200c-3p	NIN	ninein
hsa-miR-200c-3p	TAF9B	TATA-box binding protein associated factor 9b
hsa-miR-200c-3p	BAG5	BCL2 associated athanogene 5
hsa-miR-200c-3p	PAPOLA	poly(A) polymerase alpha
hsa-miR-200c-3p	UBQLN1	ubiquilin 1
hsa-miR-200c-3p	SHROOM2	shroom family member 2
hsa-miR-200c-3p	LAMC1	laminin subunit gamma 1
hsa-miR-200c-3p	SYNCRIP	synaptotagmin binding cytoplasmic RNA interacting protein
hsa-miR-200c-3p	CPEB3	cytoplasmic polyadenylation element binding protein 3
hsa-miR-200c-3p	DCT	dopachrome tautomerase
hsa-miR-200c-3p	CYP11B1	cytochrome P450 family 11 subfamily B member 1
hsa-miR-200c-3p	GPATCH2L	G-patch domain containing 2 like
hsa-miR-200c-3p	ROBO2	roundabout guidance receptor 2
hsa-miR-200c-3p	XKR6	XK related 6
hsa-miR-200c-3p	STXBP6	syntaxin binding protein 6
hsa-miR-200c-3p	SWAP70	switching B cell complex subunit SWAP70
hsa-miR-200c-3p	LATS2	large tumor suppressor kinase 2
hsa-miR-200c-3p	FYN	FYN proto-oncogene, Src family tyrosine kinase
hsa-miR-200c-3p	PPP1CB	protein phosphatase 1 catalytic subunit beta
hsa-miR-200c-3p	RIMKLB	ribosomal modification protein rimK like family member B
hsa-miR-200c-3p	SASH1	SAM and SH3 domain containing 1
hsa-miR-200c-3p	NUP160	nucleoporin 160

hsa-miR-200c-3p	SHOX2	short stature homeobox 2
hsa-miR-200c-3p	HOXA5	homeobox A5
hsa-miR-200c-3p	GABPA	GA binding protein transcription factor subunit alpha
hsa-miR-200c-3p	SLC15A5	solute carrier family 15 member 5
hsa-miR-200c-3p	KANK1	KN motif and ankyrin repeat domains 1
hsa-miR-200c-3p	LMO7	LIM domain 7
hsa-miR-200c-3p	TMA16	translation machinery associated 16 homolog
hsa-miR-200c-3p	PCSK2	proprotein convertase subtilisin/kexin type 2
hsa-miR-200c-3p	SH3GL1	SH3 domain containing GRB2 like 1, endophilin A2
hsa-miR-200c-3p	SLC38A2	solute carrier family 38 member 2
hsa-miR-200c-3p	CNN3	calponin 3
hsa-miR-200c-3p	ZNF302	zinc finger protein 302
hsa-miR-200c-3p	RNF5	ring finger protein 5
hsa-miR-200c-3p	FSIP1	fibrous sheath interacting protein 1
hsa-miR-200c-3p	LRRC58	leucine rich repeat containing 58
hsa-miR-200c-3p	CNTFR	ciliary neurotrophic factor receptor
hsa-miR-200c-3p	ZFHX4	zinc finger homeobox 4
hsa-miR-200c-3p	ACACA	acetyl-CoA carboxylase alpha
hsa-miR-200c-3p	ELMOD1	ELMO domain containing 1
hsa-miR-200c-3p	CASP2	caspase 2
hsa-miR-200c-3p	LCA5	LCA5, lebercilin
hsa-miR-200c-3p	RAPGEF2	Rap guanine nucleotide exchange factor 2
hsa-miR-200c-3p	IKBKB	inhibitor of nuclear factor kappa B kinase subunit beta
hsa-miR-200c-3p	SPTSSA	serine palmitoyltransferase small subunit A
hsa-miR-200c-3p	FUNDC1	FUN14 domain containing 1
hsa-miR-200c-3p	UBXN8	UBX domain protein 8
hsa-miR-200c-3p	NFIB	nuclear factor I B
hsa-miR-200c-3p	USP18	ubiquitin specific peptidase 18
hsa-miR-200c-3p	TBCK	TBC1 domain containing kinase
hsa-miR-200c-3p	LARP1B	La ribonucleoprotein domain family member 1B
hsa-miR-200c-3p	RNF180	ring finger protein 180
hsa-miR-200c-3p	UBE2D1	ubiquitin conjugating enzyme E2 D1
hsa-miR-200c-3p	GTF2E1	general transcription factor IIE subunit 1
hsa-miR-200c-3p	SBF1	SET binding factor 1
hsa-miR-200c-3p	SIX3	SIX homeobox 3
hsa-miR-200c-3p	UBA6	ubiquitin like modifier activating enzyme 6
hsa-miR-200c-3p	PLK2	polo like kinase 2
hsa-miR-200c-3p	SUPT20H	SPT20 homolog, SAGA complex component
hsa-miR-200c-3p	YAP1	Yes associated protein 1
hsa-miR-200c-3p	KLHL29	kelch like family member 29
hsa-miR-200c-3p	ZBTB20	zinc finger and BTB domain containing 20
hsa-miR-200c-3p	C6orf120	chromosome 6 open reading frame 120
hsa-miR-200c-3p	TRIM71	tripartite motif containing 71
hsa-miR-200c-3p	SLC16A2	solute carrier family 16 member 2
hsa-miR-200c-3p	GNAI3	G protein subunit alpha i3

hsa-miR-200c-3p	EFNA1	ephrin A1
hsa-miR-200c-3p	USP43	ubiquitin specific peptidase 43
hsa-miR-200c-3p	PAK6	p21 (RAC1) activated kinase 6
hsa-miR-200c-3p	KMT2C	lysine methyltransferase 2C
hsa-miR-200c-3p	AMBRA1	autophagy and beclin 1 regulator 1
hsa-miR-200c-3p	RASSF6	Ras association domain family member 6
hsa-miR-200c-3p	EPHA5	EPH receptor A5
hsa-miR-200c-3p	KDSR	3-ketodihydrosphingosine reductase
hsa-miR-200c-3p	MSANTD2	Myb/SANT DNA binding domain containing 2
hsa-miR-200c-3p	ZNF697	zinc finger protein 697
hsa-miR-200c-3p	PRKN	parkin RBR E3 ubiquitin protein ligase
hsa-miR-200c-3p	CDR2	cerebellar degeneration related protein 2
hsa-miR-200c-3p	ASH1L	ASH1 like histone lysine methyltransferase
hsa-miR-200c-3p	TIFA	TRAF interacting protein with forkhead associated domain
hsa-miR-200c-3p	SLC4A4	solute carrier family 4 member 4
hsa-miR-200c-3p	TMEM164	transmembrane protein 164
hsa-miR-200c-3p	ANO5	anoctamin 5
hsa-miR-200c-3p	MXD4	MAX dimerization protein 4
hsa-miR-200c-3p	NRXN1	neurexin 1
hsa-miR-200c-3p	TMX1	thioredoxin related transmembrane protein 1
hsa-miR-200c-3p	SLC39A8	solute carrier family 39 member 8
hsa-miR-200c-3p	B4GALT6	beta-1,4-galactosyltransferase 6
hsa-miR-200c-3p	GAB1	GRB2 associated binding protein 1
hsa-miR-200c-3p	AAK1	AP2 associated kinase 1
hsa-miR-200c-3p	DNAJB6	DnaJ heat shock protein family (Hsp40) member B6
hsa-miR-200c-3p	STYX	serine/threonine/tyrosine interacting protein
hsa-miR-200c-3p	TAF4	TATA-box binding protein associated factor 4
hsa-miR-200c-3p	KCNMB1	potassium calcium-activated channel subfamily M regulatory beta subunit 1
hsa-miR-200c-3p	RNF169	ring finger protein 169
hsa-miR-200c-3p	C4orf46	chromosome 4 open reading frame 46
hsa-miR-200c-3p	FAM227A	family with sequence similarity 227 member A
hsa-miR-200c-3p	CPPED1	calcineurin like phosphoesterase domain containing 1
hsa-miR-200c-3p	ICK	intestinal cell kinase
hsa-miR-200c-3p	TMEM135	transmembrane protein 135
hsa-miR-200c-3p	TBC1D22B	TBC1 domain family member 22B
hsa-miR-200c-3p	CCDC186	coiled-coil domain containing 186
hsa-miR-200c-3p	SLC35A2	solute carrier family 35 member A2
hsa-miR-200c-3p	SEC24A	SEC24 homolog A, COPII coat complex component
hsa-miR-200c-3p	DNAJC10	DnaJ heat shock protein family (Hsp40) member C10
hsa-miR-200c-3p	GRAP2	GRB2 related adaptor protein 2
hsa-miR-200c-3p	KPNA4	karyopherin subunit alpha 4
hsa-miR-200c-3p	YTHDF3	YTH N6-methyladenosine RNA binding protein 3
hsa-miR-200c-3p	NCOA7	nuclear receptor coactivator 7

hsa-miR-200c-3p	FBXO22	F-box protein 22
hsa-miR-200c-3p	UBE2I	ubiquitin conjugating enzyme E2 I
hsa-miR-200c-3p	ADRB2	adrenoceptor beta 2
hsa-miR-200c-3p	MUL1	mitochondrial E3 ubiquitin protein ligase 1
hsa-miR-200c-3p	UBAC2	UBA domain containing 2
hsa-miR-200c-3p	RAB8B	RAB8B, member RAS oncogene family
hsa-miR-200c-3p	RFTN2	raftlin family member 2
hsa-miR-200c-3p	ADCY9	adenylate cyclase 9
hsa-miR-200c-3p	PCDH7	protocadherin 7
hsa-miR-200c-3p	MIER3	MIER family member 3
hsa-miR-200c-3p	PHACTR2	phosphatase and actin regulator 2
hsa-miR-200c-3p	CACUL1	CDK2 associated cullin domain 1
hsa-miR-200c-3p	BLOC1S6	biogenesis of lysosomal organelles complex 1 subunit 6
hsa-miR-200c-3p	SOX1	SRY-box 1
hsa-miR-200c-3p	KIAA1841	KIAA1841
hsa-miR-200c-3p	PRKAR1A	protein kinase cAMP-dependent type I regulatory subunit alpha
hsa-miR-200c-3p	HTR2A	5-hydroxytryptamine receptor 2A
hsa-miR-200c-3p	FUT9	fucosyltransferase 9
hsa-miR-200c-3p	ACVR2B	activin A receptor type 2B
hsa-miR-200c-3p	LONRF2	LON peptidase N-terminal domain and ring finger 2
hsa-miR-200c-3p	RRAS2	RAS related 2
hsa-miR-200c-3p	ENOX2	ecto-NOX disulfide-thiol exchanger 2
hsa-miR-200c-3p	ZFY	zinc finger protein Y-linked
hsa-miR-200c-3p	TM7SF3	transmembrane 7 superfamily member 3
hsa-miR-200c-3p	MTMR6	myotubularin related protein 6
hsa-miR-200c-3p	MAGEC2	MAGE family member C2
hsa-miR-200c-3p	ZFR	zinc finger RNA binding protein
hsa-miR-200c-3p	AP1AR	adaptor related protein complex 1 associated regulatory protein
hsa-miR-200c-3p	NCOA4	nuclear receptor coactivator 4
hsa-miR-200c-3p	PSTPIP2	proline-serine-threonine phosphatase interacting protein 2
hsa-miR-200c-3p	FUBP3	far upstream element binding protein 3
hsa-miR-200c-3p	SHCBP1	SHC binding and spindle associated 1
hsa-miR-200c-3p	TIAL1	TIA1 cytotoxic granule associated RNA binding protein like 1
hsa-miR-200c-3p	MEX3B	mex-3 RNA binding family member B
hsa-miR-200c-3p	GPR173	G protein-coupled receptor 173
hsa-miR-200c-3p	SYNC	syncoilin, intermediate filament protein
hsa-miR-200c-3p	GIGYF1	GRB10 interacting GYF protein 1
hsa-miR-200c-3p	IQCJ-SCHIP1	IQCJ-SCHIP1 readthrough
hsa-miR-200c-3p	XG	Xg glycoprotein (Xg blood group)
hsa-miR-200c-3p	ZBTB8A	zinc finger and BTB domain containing 8A
hsa-miR-200c-3p	TMEM170B	transmembrane protein 170B

hsa-miR-200c-3p	UTY	ubiquitously transcribed tetratricopeptide repeat containing, Y-linked
hsa-miR-200c-3p	EIF5B	eukaryotic translation initiation factor 5B
hsa-miR-200c-3p	NCAM1	neural cell adhesion molecule 1
hsa-miR-200c-3p	PAIP2	poly(A) binding protein interacting protein 2
hsa-miR-200c-3p	RBFOX1	RNA binding fox-1 homolog 1
hsa-miR-200c-3p	SNAP25	synaptosome associated protein 25
hsa-miR-200c-3p	EP300	E1A binding protein p300
hsa-miR-200c-3p	CERS6	ceramide synthase 6
hsa-miR-200c-3p	GMFB	glia maturation factor beta
hsa-miR-200c-3p	NFYA	nuclear transcription factor Y subunit alpha
hsa-miR-200c-3p	PARD3B	par-3 family cell polarity regulator beta
hsa-miR-200c-3p	HNF1B	HNF1 homeobox B
hsa-miR-200c-3p	MARCH8	membrane associated ring-CH-type finger 8
hsa-miR-200c-3p	ZDHHC17	zinc finger DHHC-type containing 17
hsa-miR-200c-3p	C5orf24	chromosome 5 open reading frame 24
hsa-miR-200c-3p	WDR91	WD repeat domain 91
hsa-miR-200c-3p	COG6	component of oligomeric golgi complex 6
hsa-miR-200c-3p	MSTN	myostatin
hsa-miR-200c-3p	CREBBP	CREB binding protein
hsa-miR-200c-3p	NDNF	neuron derived neurotrophic factor
hsa-miR-200c-3p	OPRM1	opioid receptor mu 1
hsa-miR-200c-3p	CALU	calumenin
hsa-miR-200c-3p	RSRC2	arginine and serine rich coiled-coil 2
hsa-miR-200c-3p	LPAR1	lysophosphatidic acid receptor 1
hsa-miR-200c-3p	PTPN22	protein tyrosine phosphatase, non-receptor type 22
hsa-miR-200c-3p	RHOT1	ras homolog family member T1
hsa-miR-200c-3p	N4BP2	NEDD4 binding protein 2
hsa-miR-200c-3p	RNF38	ring finger protein 38
hsa-miR-200c-3p	RBM26	RNA binding motif protein 26
hsa-miR-200c-3p	SLTM	SAFB like transcription modulator
hsa-miR-200c-3p	CLOCK	clock circadian regulator
hsa-miR-200c-3p	CDC73	cell division cycle 73
hsa-miR-200c-3p	PLS3	plastin 3
hsa-miR-200c-3p	ADCY2	adenylate cyclase 2
hsa-miR-200c-3p	CALN1	calneuron 1
hsa-miR-200c-3p	C15orf40	chromosome 15 open reading frame 40
hsa-miR-200c-3p	TP73	tumor protein p73
hsa-miR-200c-3p	FRMD4A	FERM domain containing 4A
hsa-miR-200c-3p	TMEM170A	transmembrane protein 170A
hsa-miR-200c-3p	DCAF17	DDB1 and CUL4 associated factor 17
hsa-miR-200c-3p	ANGEL2	angel homolog 2
hsa-miR-200c-3p	RPRD1A	regulation of nuclear pre-mRNA domain containing 1A
hsa-miR-200c-3p	EHD1	EH domain containing 1
hsa-miR-200c-3p	ACADSB	acyl-CoA dehydrogenase short/branched chain

hsa-miR-200c-3p	ARL15	ADP ribosylation factor like GTPase 15
hsa-miR-200c-3p	ALG2	ALG2, alpha-1,3/1,6-mannosyltransferase
hsa-miR-200c-3p	PALM2	paralemmin 2
hsa-miR-200c-3p	PPWD1	peptidylprolyl isomerase domain and WD repeat containing 1
hsa-miR-200c-3p	SRP72	signal recognition particle 72
hsa-miR-200c-3p	ARGLU1	arginine and glutamate rich 1
hsa-miR-200c-3p	TMEM33	transmembrane protein 33
hsa-miR-200c-3p	PPARA	peroxisome proliferator activated receptor alpha
hsa-miR-200c-3p	PTGER2	prostaglandin E receptor 2
hsa-miR-200c-3p	C19orf12	chromosome 19 open reading frame 12
hsa-miR-200c-3p	BAG6	BCL2 associated athanogene 6
hsa-miR-200c-3p	TLL2	tolloid like 2
hsa-miR-200c-3p	ANKRD33B	ankyrin repeat domain 33B
hsa-miR-200c-3p	PNISR	PNN interacting serine and arginine rich protein
hsa-miR-200c-3p	DOCK4	dedicator of cytokinesis 4
hsa-miR-200c-3p	MTMR9	myotubularin related protein 9
hsa-miR-200c-3p	ZNF557	zinc finger protein 557
hsa-miR-200c-3p	C2CD6	C2 calcium dependent domain containing 6
hsa-miR-200c-3p	FAM81A	family with sequence similarity 81 member A
hsa-miR-200c-3p	FASTKD3	FAST kinase domains 3
hsa-miR-200c-3p	TMEM245	transmembrane protein 245
hsa-miR-200c-3p	MON2	MON2 homolog, regulator of endosome-to-Golgi trafficking
hsa-miR-200c-3p	MXD3	MAX dimerization protein 3
hsa-miR-200c-3p	KIF11	kinesin family member 11
hsa-miR-200c-3p	AFF4	AF4/FMR2 family member 4
hsa-miR-200c-3p	SLC6A15	solute carrier family 6 member 15
hsa-miR-200c-3p	CNST	consortin, connexin sorting protein
hsa-miR-200c-3p	PPP2CA	protein phosphatase 2 catalytic subunit alpha
hsa-miR-200c-3p	CTNND2	catenin delta 2
hsa-miR-200c-3p	PHC3	polyhomeotic homolog 3
hsa-miR-200c-3p	ORMDL3	ORMDL sphingolipid biosynthesis regulator 3
hsa-miR-200c-3p	SRF	serum response factor
hsa-miR-200c-3p	CASK	calcium/calmodulin dependent serine protein kinase
hsa-miR-200c-3p	LCORL	ligand dependent nuclear receptor corepressor like
hsa-miR-200c-3p	YPEL1	yippee like 1
hsa-miR-200c-3p	GABRB3	gamma-aminobutyric acid type A receptor beta3 subunit
hsa-miR-200c-3p	TFPI	tissue factor pathway inhibitor
hsa-miR-200c-3p	UNC80	unc-80 homolog, NALCN channel complex subunit
hsa-miR-200c-3p	MKLN1	muskelin 1
hsa-miR-200c-3p	STARD13	StAR related lipid transfer domain containing 13
hsa-miR-200c-3p	BPY2C	basic charge Y-linked 2C
hsa-miR-200c-3p	MAMDC2	MAM domain containing 2
hsa-miR-200c-3p	BPY2B	basic charge Y-linked 2B

hsa-miR-200c-3p	SSR3	signal sequence receptor subunit 3
hsa-miR-200c-3p	SLC25A27	solute carrier family 25 member 27
hsa-miR-200c-3p	FUT4	fucosyltransferase 4
hsa-miR-200c-3p	LFNG	LFNG O-fucosylpeptide 3-beta-N-acetylglucosaminyltransferase
hsa-miR-200c-3p	UBE2V1	ubiquitin conjugating enzyme E2 V1
hsa-miR-200c-3p	FHOD1	formin homology 2 domain containing 1
hsa-miR-200c-3p	SYT1	synaptotagmin 1
hsa-miR-200c-3p	TIGAR	TP53 induced glycolysis regulatory phosphatase
hsa-miR-200c-3p	NPAP1	nuclear pore associated protein 1
hsa-miR-200c-3p	FBXW11	F-box and WD repeat domain containing 11
hsa-miR-200c-3p	DSG1	desmoglein 1
hsa-miR-200c-3p	LETM2	leucine zipper and EF-hand containing transmembrane protein 2
hsa-miR-200c-3p	MYT1	myelin transcription factor 1
hsa-miR-200c-3p	TAB3	TGF-beta activated kinase 1 (MAP3K7) binding protein 3
hsa-miR-200c-3p	PCNX1	pecanex 1
hsa-miR-200c-3p	ZNF292	zinc finger protein 292
hsa-miR-200c-3p	TBX5	T-box 5
hsa-miR-200c-3p	BPY2	basic charge Y-linked 2
hsa-miR-200c-3p	MMD2	monocyte to macrophage differentiation associated 2
hsa-miR-200c-3p	RAC1	Rac family small GTPase 1
hsa-miR-200c-3p	TMEM189-UBE2V1	TMEM189-UBE2V1 readthrough
hsa-miR-200c-3p	STAM2	signal transducing adaptor molecule 2
hsa-miR-200c-3p	CHST2	carbohydrate sulfotransferase 2
hsa-miR-200c-3p	GLRX3	glutaredoxin 3
hsa-miR-200c-3p	NLGN4X	neuroligin 4 X-linked
hsa-miR-200c-3p	BTF3L4	basic transcription factor 3 like 4
hsa-miR-200c-3p	SOWAHC	sosondowah ankyrin repeat domain family member C
hsa-miR-200c-3p	SGPP1	sphingosine-1-phosphate phosphatase 1
hsa-miR-200c-3p	PRRG4	proline rich and Gla domain 4
hsa-miR-200c-3p	ZC3H12B	zinc finger CCCH-type containing 12B
hsa-miR-200c-3p	NPTX1	neuronal pentraxin 1
hsa-miR-200c-3p	TMEM167A	transmembrane protein 167A
hsa-miR-200c-3p	HNRNPH3	heterogeneous nuclear ribonucleoprotein H3
hsa-miR-200c-3p	MTFR1	mitochondrial fission regulator 1
hsa-miR-200c-3p	RBL1	RB transcriptional corepressor like 1
hsa-miR-200c-3p	ELL	elongation factor for RNA polymerase II
hsa-miR-200c-3p	TBX22	T-box 22
hsa-miR-200c-3p	RLF	rearranged L-myc fusion
hsa-miR-200c-3p	CNTN4	contactin 4
hsa-miR-200c-3p	C6orf62	chromosome 6 open reading frame 62
hsa-miR-200c-3p	CADM2	cell adhesion molecule 2
hsa-miR-200c-3p	CNOT6L	CCR4-NOT transcription complex subunit 6 like

hsa-miR-200c-3p	PMM1	phosphomannomutase 1
hsa-miR-200c-3p	DIRAS2	DIRAS family GTPase 2
hsa-miR-200c-3p	SHC4	SHC adaptor protein 4
hsa-miR-200c-3p	TJAP1	tight junction associated protein 1
hsa-miR-200c-3p	RBM12B	RNA binding motif protein 12B
hsa-miR-200c-3p	LATS1	large tumor suppressor kinase 1
hsa-miR-200c-3p	EIF5A2	eukaryotic translation initiation factor 5A2
hsa-miR-200c-3p	UGCG	UDP-glucose ceramide glucosyltransferase
hsa-miR-200c-3p	RAB33B	RAB33B, member RAS oncogene family
hsa-miR-200c-3p	ZNF605	zinc finger protein 605
hsa-miR-200c-3p	PLOD2	procollagen-lysine,2-oxoglutarate 5-dioxygenase 2
hsa-miR-200c-3p	NSD2	nuclear receptor binding SET domain protein 2
hsa-miR-200c-3p	PHLDB1	pleckstrin homology like domain family B member 1
hsa-miR-200c-3p	SRSF1	serine and arginine rich splicing factor 1
hsa-miR-200c-3p	RPL22L1	ribosomal protein L22 like 1
hsa-miR-200c-3p	ARFGEF3	ARFGEF family member 3
hsa-miR-200c-3p	CYP1B1	cytochrome P450 family 1 subfamily B member 1
hsa-miR-200c-3p	NGEF	neuronal guanine nucleotide exchange factor
hsa-miR-200c-3p	TSTD3	thiosulfate sulfurtransferase like domain containing 3
hsa-miR-200c-3p	EFHC2	EF-hand domain containing 2
hsa-miR-200c-3p	PAK3	p21 (RAC1) activated kinase 3
hsa-miR-200c-3p	SLC23A2	solute carrier family 23 member 2
hsa-miR-200c-3p	KLRF1	killer cell lectin like receptor F1
hsa-miR-200c-3p	ESM1	endothelial cell specific molecule 1
hsa-miR-200c-3p	MED6	mediator complex subunit 6
hsa-miR-200c-3p	SLC6A17	solute carrier family 6 member 17
hsa-miR-200c-3p	PPP2R2C	protein phosphatase 2 regulatory subunit Bgamma
hsa-miR-200c-3p	FAR2	fatty acyl-CoA reductase 2
hsa-miR-200c-3p	ZNF652	zinc finger protein 652
hsa-miR-200c-3p	LIN28B	lin-28 homolog B
hsa-miR-200c-3p	LCOR	ligand dependent nuclear receptor corepressor
hsa-miR-200c-3p	CALCR	calcitonin receptor
hsa-miR-200c-3p	ALDH1A3	aldehyde dehydrogenase 1 family member A3
hsa-miR-200c-3p	UBN2	ubinuclein 2
hsa-miR-200c-3p	BTBD18	BTB domain containing 18
hsa-miR-200c-3p	PDLIM5	PDZ and LIM domain 5
hsa-miR-200c-3p	PAK5	p21 (RAC1) activated kinase 5
hsa-miR-200c-3p	MSR1	macrophage scavenger receptor 1
hsa-miR-200c-3p	GPX6	glutathione peroxidase 6
hsa-miR-200c-3p	HIPK2	homeodomain interacting protein kinase 2
hsa-miR-200c-3p	SIRPA	signal regulatory protein alpha
hsa-miR-200c-3p	SLC35F4	solute carrier family 35 member F4
hsa-miR-200c-3p	JMY	junction mediating and regulatory protein, p53 cofactor
hsa-miR-200c-3p	ASF1A	anti-silencing function 1A histone chaperone
hsa-miR-200c-3p	INPP4A	inositol polyphosphate-4-phosphatase type I A

hsa-miR-200c-3p	SNAI2	snail family transcriptional repressor 2
hsa-miR-200c-3p	LPIN1	lipin 1
hsa-miR-200c-3p	TERF1	telomeric repeat binding factor 1
hsa-miR-200c-3p	RUNX1T1	RUNX1 translocation partner 1
hsa-miR-200c-3p	TAOK1	TAO kinase 1
hsa-miR-200c-3p	TXLNG	taxilin gamma
hsa-miR-200c-3p	NPNT	nephronectin
hsa-miR-200c-3p	MAPK1IP1L	mitogen-activated protein kinase 1 interacting protein 1 like
hsa-miR-200c-3p	SH3PXD2A	SH3 and PX domains 2A
hsa-miR-200c-3p	ZNF555	zinc finger protein 555
hsa-miR-200c-3p	ASXL3	ASXL transcriptional regulator 3
hsa-miR-200c-3p	PCNP	PEST proteolytic signal containing nuclear protein
hsa-miR-200c-3p	SHOC1	shortage in chiasmata 1
hsa-miR-200c-3p	ZNF181	zinc finger protein 181
hsa-miR-200c-3p	PIP4K2B	phosphatidylinositol-5-phosphate 4-kinase type 2 beta
hsa-miR-200c-3p	PLEKHM3	pleckstrin homology domain containing M3
hsa-miR-200c-3p	NCOA3	nuclear receptor coactivator 3
hsa-miR-200c-3p	ARHGAP19	Rho GTPase activating protein 19
hsa-miR-200c-3p	FXR2	FMR1 autosomal homolog 2
hsa-miR-200c-3p	SNX13	sorting nexin 13
hsa-miR-200c-3p	PLCG1	phospholipase C gamma 1
hsa-miR-200c-3p	PHF21A	PHD finger protein 21A
hsa-miR-200c-3p	GIN1	gypsy retrotransposon integrase 1
hsa-miR-200c-3p	TRAM1L1	translocation associated membrane protein 1 like 1
hsa-miR-200c-3p	THAP5	THAP domain containing 5
hsa-miR-200c-3p	SNAPC1	small nuclear RNA activating complex polypeptide 1
hsa-miR-200c-3p	C2orf49	chromosome 2 open reading frame 49
hsa-miR-200c-3p	TPCN1	two pore segment channel 1
hsa-miR-200c-3p	PTS	6-pyruvoyltetrahydropterin synthase
hsa-miR-200c-3p	PARD6B	par-6 family cell polarity regulator beta
hsa-miR-200c-3p	CDK15	cyclin dependent kinase 15
hsa-miR-200c-3p	THRB	thyroid hormone receptor beta
hsa-miR-200c-3p	MPP4	membrane palmitoylated protein 4
hsa-miR-200c-3p	RELN	reelin
hsa-miR-200c-3p	ZNF516	zinc finger protein 516
hsa-miR-200c-3p	KLK7	kallikrein related peptidase 7
hsa-miR-200c-3p	PAX6	paired box 6
hsa-miR-200c-3p	KLHL31	kelch like family member 31
hsa-miR-200c-3p	NCOR2	nuclear receptor corepressor 2
hsa-miR-200c-3p	KCTD15	potassium channel tetramerization domain containing 15
hsa-miR-200c-3p	PSPH	phosphoserine phosphatase
hsa-miR-200c-3p	PIN1	peptidylprolyl cis/trans isomerase, NIMA-interacting 1
hsa-miR-200c-3p	CHD2	chromodomain helicase DNA binding protein 2
hsa-miR-200c-3p	MAP1LC3B	microtubule associated protein 1 light chain 3 beta

hsa-miR-200c-3p	HPS5	HPS5, biogenesis of lysosomal organelles complex 2 subunit 2
hsa-miR-200c-3p	TMCC1	transmembrane and coiled-coil domain family 1
hsa-miR-200c-3p	PLPPR1	phospholipid phosphatase related 1
hsa-miR-200c-3p	LYRM2	LYR motif containing 2
hsa-miR-200c-3p	FAM169A	family with sequence similarity 169 member A
hsa-miR-200c-3p	ITPR1	inositol 1,4,5-trisphosphate receptor type 1
hsa-miR-200c-3p	HEG1	heart development protein with EGF like domains 1
hsa-miR-200c-3p	CHST9	carbohydrate sulfotransferase 9
hsa-miR-200c-3p	DMRT2	doublesex and mab-3 related transcription factor 2
hsa-miR-200c-3p	PRKAR2B	protein kinase cAMP-dependent type II regulatory subunit beta
hsa-miR-200c-3p	TJP1	tight junction protein 1
hsa-miR-200c-3p	FAM218A	family with sequence similarity 218 member A
hsa-miR-200c-3p	IQSEC1	IQ motif and Sec7 domain 1
hsa-miR-200c-3p	HS6ST2	heparan sulfate 6-O-sulfotransferase 2
hsa-miR-200c-3p	MYCN	MYCN proto-oncogene, bHLH transcription factor
hsa-miR-200c-3p	LY75	lymphocyte antigen 75
hsa-miR-200c-3p	REV3L	REV3 like, DNA directed polymerase zeta catalytic subunit
hsa-miR-200c-3p	ARIH2	ariadne RBR E3 ubiquitin protein ligase 2
hsa-miR-200c-3p	CHM	CHM, Rab escort protein 1
hsa-miR-200c-3p	CSRNP3	cysteine and serine rich nuclear protein 3
hsa-miR-200c-3p	KCNK2	potassium two pore domain channel subfamily K member 2
hsa-miR-200c-3p	MARCH1	membrane associated ring-CH-type finger 1
hsa-miR-200c-3p	ZNF274	zinc finger protein 274
hsa-miR-200c-3p	NEGR1	neuronal growth regulator 1
hsa-miR-200c-3p	STON2	stonin 2
hsa-miR-200c-3p	FCAR	Fc fragment of IgA receptor
hsa-miR-200c-3p	FAM3B	family with sequence similarity 3 member B
hsa-miR-200c-3p	CFLAR	CASP8 and FADD like apoptosis regulator
hsa-miR-200c-3p	PTBP1	polypyrimidine tract binding protein 1
hsa-miR-200c-3p	FAM107B	family with sequence similarity 107 member B
hsa-miR-200c-3p	LRP4	LDL receptor related protein 4
hsa-miR-200c-3p	F2RL2	coagulation factor II thrombin receptor like 2
hsa-miR-200c-3p	MTCP1	mature T cell proliferation 1
hsa-miR-200c-3p	OTUD6B	OTU domain containing 6B
hsa-miR-200c-3p	ZNF566	zinc finger protein 566
hsa-miR-200c-3p	CA5B	carbonic anhydrase 5B
hsa-miR-200c-3p	TRPV3	transient receptor potential cation channel subfamily V member 3
hsa-miR-200c-3p	DCAF5	DDB1 and CUL4 associated factor 5
hsa-miR-200c-3p	BASP1	brain abundant membrane attached signal protein 1
hsa-miR-200c-3p	GSKIP	GSK3B interacting protein
hsa-miR-200c-3p	TRPC3	transient receptor potential cation channel subfamily C member 3

hsa-miR-200c-3p	PRRC2C	proline rich coiled-coil 2C
hsa-miR-200c-3p	VPS13A	vacuolar protein sorting 13 homolog A
hsa-miR-200c-3p	AP1M1	adaptor related protein complex 1 subunit mu 1
hsa-miR-200c-3p	CNTD1	cyclin N-terminal domain containing 1
hsa-miR-200c-3p	SCOC	short coiled-coil protein
hsa-miR-200c-3p	BCLAF3	BCLAF1 and THRAP3 family member 3
hsa-miR-200c-3p	TM4SF18	transmembrane 4 L six family member 18
hsa-miR-200c-3p	SMAD5	SMAD family member 5
hsa-miR-200c-3p	GFI1	growth factor independent 1 transcriptional repressor
hsa-miR-200c-3p	ZNF365	zinc finger protein 365
hsa-miR-200c-3p	ATP11A	ATPase phospholipid transporting 11A
hsa-miR-200c-3p	NLRP11	NLR family pyrin domain containing 11
hsa-miR-200c-3p	CDK12	cyclin dependent kinase 12
hsa-miR-200c-3p	WSB1	WD repeat and SOCS box containing 1
hsa-miR-200c-3p	PSMF1	proteasome inhibitor subunit 1
hsa-miR-200c-3p	CDHR1	cadherin related family member 1
hsa-miR-200c-3p	RIF1	replication timing regulatory factor 1
hsa-miR-200c-3p	HNRNPH2	heterogeneous nuclear ribonucleoprotein H2
hsa-miR-200c-3p	ZNF366	zinc finger protein 366
hsa-miR-200c-3p	NAA50	N(alpha)-acetyltransferase 50, NatE catalytic subunit
hsa-miR-200c-3p	SUB1	SUB1 homolog, transcriptional regulator
hsa-miR-200c-3p	C22orf39	chromosome 22 open reading frame 39
hsa-miR-200c-3p	SPIDR	scaffold protein involved in DNA repair
hsa-miR-200c-3p	NIP7	NIP7, nucleolar pre-rRNA processing protein
hsa-miR-200c-3p	TGFBR3	transforming growth factor beta receptor 3
hsa-miR-200c-3p	TMEM229B	transmembrane protein 229B
hsa-miR-200c-3p	UTP25	UTP25, small subunit processor component
hsa-miR-200c-3p	PI15	peptidase inhibitor 15
hsa-miR-200c-3p	IRS2	insulin receptor substrate 2
hsa-miR-200c-3p	ANKRD28	ankyrin repeat domain 28
hsa-miR-200c-3p	WDR45B	WD repeat domain 45B
hsa-miR-200c-3p	C21orf91	chromosome 21 open reading frame 91
hsa-miR-200c-3p	FGD1	FYVE, RhoGEF and PH domain containing 1
hsa-miR-200c-3p	FMR1	fragile X mental retardation 1
hsa-miR-200c-3p	NFASC	neurofascin
hsa-miR-200c-3p	RFC3	replication factor C subunit 3
hsa-miR-200c-3p	RNASEL	ribonuclease L
hsa-miR-200c-3p	OSR1	odd-skipped related transcription factor 1
hsa-miR-200c-3p	FRS2	fibroblast growth factor receptor substrate 2
hsa-miR-200c-3p	C2orf15	chromosome 2 open reading frame 15
hsa-miR-200c-3p	NR2C1	nuclear receptor subfamily 2 group C member 1
hsa-miR-200c-3p	ING5	inhibitor of growth family member 5
hsa-miR-200c-3p	PENK	proenkephalin
hsa-miR-200c-3p	SOCS6	suppressor of cytokine signaling 6
hsa-miR-200c-3p	SEPHS1	selenophosphate synthetase 1

hsa-miR-200c-3p	NEO1	neogenin 1
hsa-miR-200c-3p	TP53INP1	tumor protein p53 inducible nuclear protein 1
hsa-miR-200c-3p	TMEM43	transmembrane protein 43
hsa-miR-200c-3p	RBM8A	RNA binding motif protein 8A
hsa-miR-200c-3p	PLXNA4	plexin A4
hsa-miR-200c-3p	GRIP1	glutamate receptor interacting protein 1
hsa-miR-200c-3p	MTAP	methylthioadenosine phosphorylase
hsa-miR-200c-3p	NOC3L	NOC3 like DNA replication regulator
hsa-miR-200c-3p	GATA2	GATA binding protein 2
hsa-miR-200c-3p	EGLN3	egl-9 family hypoxia inducible factor 3
hsa-miR-200c-3p	RASGEF1B	RasGEF domain family member 1B
hsa-miR-200c-3p	HBS1L	HBS1 like translational GTPase
hsa-miR-200c-3p	RAB30	RAB30, member RAS oncogene family
hsa-miR-200c-3p	NR2C2	nuclear receptor subfamily 2 group C member 2
hsa-miR-200c-3p	SOD2	superoxide dismutase 2
hsa-miR-200c-3p	ZNF330	zinc finger protein 330
hsa-miR-200c-3p	ZNF260	zinc finger protein 260
hsa-miR-200c-3p	MLLT10	MLLT10, histone lysine methyltransferase DOT1L cofactor
hsa-miR-200c-3p	ITPRID2	ITPR interacting domain containing 2
hsa-miR-200c-3p	NPR3	natriuretic peptide receptor 3
hsa-miR-200c-3p	CRYZ	crystallin zeta
hsa-miR-200c-3p	ONECUT2	one cut homeobox 2
hsa-miR-200c-3p	YOD1	YOD1 deubiquitinase
hsa-miR-200c-3p	LRRTM3	leucine rich repeat transmembrane neuronal 3
hsa-miR-200c-3p	ARPP21	cAMP regulated phosphoprotein 21
hsa-miR-200c-3p	CDK1	cyclin dependent kinase 1
hsa-miR-200c-3p	NAA16	N(alpha)-acetyltransferase 16, NatA auxiliary subunit
hsa-miR-200c-3p	TMEM123	transmembrane protein 123
hsa-miR-200c-3p	DYRK2	dual specificity tyrosine phosphorylation regulated kinase 2
hsa-miR-200c-3p	ACTC1	actin, alpha, cardiac muscle 1
hsa-miR-200c-3p	FAM160B1	family with sequence similarity 160 member B1
hsa-miR-200c-3p	UFSP2	UFM1 specific peptidase 2
hsa-miR-200c-3p	ASPH	aspartate beta-hydroxylase
hsa-miR-200c-3p	FUBP1	far upstream element binding protein 1
hsa-miR-200c-3p	HIF1AN	hypoxia inducible factor 1 subunit alpha inhibitor
hsa-miR-200c-3p	IL24	interleukin 24
hsa-miR-200c-3p	DCX	doublecortin
hsa-miR-200c-3p	CGGBP1	CGG triplet repeat binding protein 1
hsa-miR-200c-3p	ST3GAL5	ST3 beta-galactoside alpha-2,3-sialyltransferase 5
hsa-miR-200c-3p	CLIP2	CAP-Gly domain containing linker protein 2
hsa-miR-200c-3p	CD58	CD58 molecule
hsa-miR-200c-3p	ZNF550	zinc finger protein 550
hsa-miR-200c-3p	CDK2	cyclin dependent kinase 2

hsa-miR-200c-3p	TADA2B	transcriptional adaptor 2B
hsa-miR-200c-3p	FBXL16	F-box and leucine rich repeat protein 16
hsa-miR-200c-3p	TPD52L1	TPD52 like 1
hsa-miR-200c-3p	GLS	glutaminase
hsa-miR-200c-3p	SIRT1	sirtuin 1
hsa-miR-200c-3p	SPART	spartin
hsa-miR-200c-3p	ATMIN	ATM interactor
hsa-miR-200c-3p	C2orf72	chromosome 2 open reading frame 72
hsa-miR-200c-3p	C2orf68	chromosome 2 open reading frame 68
hsa-miR-200c-3p	BUB1B-PAK6	BUB1B-PAK6 readthrough
hsa-miR-200c-3p	CLCF1	cardiotrophin like cytokine factor 1
hsa-miR-200c-3p	CSTF3	cleavage stimulation factor subunit 3
hsa-miR-200c-3p	ADIPOQ	adiponectin, C1Q and collagen domain containing
hsa-miR-200c-3p	CLCC1	chloride channel CLIC like 1
hsa-miR-200c-3p	BORCS7	BLOC-1 related complex subunit 7
hsa-miR-200c-3p	SERF2	small EDRK-rich factor 2
hsa-miR-200c-3p	TREM1	triggering receptor expressed on myeloid cells 1
hsa-miR-200c-3p	ASXL1	ASXL transcriptional regulator 1
hsa-miR-200c-3p	TRIM2	tripartite motif containing 2
hsa-miR-200c-3p	RPL36A-HNRNPH2	RPL36A-HNRNPH2 readthrough
hsa-miR-200c-3p	PRPF38A	pre-mRNA processing factor 38A
hsa-miR-200c-3p	KLHL14	kelch like family member 14
hsa-miR-200c-3p	RALGPS2	Ral GEF with PH domain and SH3 binding motif 2
hsa-miR-200c-3p	LGSN	lengsin, lens protein with glutamine synthetase domain
hsa-miR-200c-3p	PAN3	poly(A) specific ribonuclease subunit PAN3
hsa-miR-200c-3p	SGCB	sarcoglycan beta
hsa-miR-200c-3p	MEGF10	multiple EGF like domains 10
hsa-miR-200c-3p	SLC16A7	solute carrier family 16 member 7
hsa-miR-200c-3p	CCSER1	coiled-coil serine rich protein 1
hsa-miR-200c-3p	BBS12	Bardet-Biedl syndrome 12
hsa-miR-200c-3p	LRRC34	leucine rich repeat containing 34
hsa-miR-200c-3p	USF3	upstream transcription factor family member 3
hsa-miR-200c-3p	CASC4	cancer susceptibility 4
hsa-miR-200c-3p	ITSN1	intersectin 1
hsa-miR-200c-3p	ERBIN	erbb2 interacting protein
hsa-miR-200c-3p	XRN2	5'-3' exoribonuclease 2
hsa-miR-200c-3p	CTNNA3	catenin alpha 3
hsa-miR-200c-3p	CREB5	cAMP responsive element binding protein 5
hsa-miR-200c-3p	PPP1R12B	protein phosphatase 1 regulatory subunit 12B
hsa-miR-200c-3p	MDM4	MDM4, p53 regulator
hsa-miR-200c-3p	XKRY2	XK related, Y-linked 2
hsa-miR-200c-3p	RBMXL2	RBMX like 2
hsa-miR-200c-3p	TENM1	teneurin transmembrane protein 1
hsa-miR-200c-3p	BHLHE41	basic helix-loop-helix family member e41

hsa-miR-200c-3p	SNTB2	syntrophin beta 2
hsa-miR-200c-3p	ATAT1	alpha tubulin acetyltransferase 1
hsa-miR-200c-3p	SSH2	slingshot protein phosphatase 2
hsa-miR-200c-3p	XKRY	XK related, Y-linked
hsa-miR-200c-3p	STAM	signal transducing adaptor molecule
hsa-miR-200c-3p	KIF13A	kinesin family member 13A
hsa-miR-200c-3p	PIGM	phosphatidylinositol glycan anchor biosynthesis class M
hsa-miR-200c-3p	LSM8	LSM8 homolog, U6 small nuclear RNA associated
hsa-miR-200c-3p	CFAP97	cilia and flagella associated protein 97
hsa-miR-200c-3p	CKLF	chemokine like factor
hsa-miR-200c-3p	RBBP4	RB binding protein 4, chromatin remodeling factor
hsa-miR-200c-3p	ALPI	alkaline phosphatase, intestinal
hsa-miR-200c-3p	TMEM26	transmembrane protein 26
hsa-miR-200c-3p	DARS	aspartyl-tRNA synthetase
hsa-miR-200c-3p	GLIS2	GLIS family zinc finger 2
hsa-miR-200c-3p	COL4A3	collagen type IV alpha 3 chain
hsa-miR-200c-3p	MOSPD2	motile sperm domain containing 2
hsa-miR-200c-3p	CA3	carbonic anhydrase 3
hsa-miR-200c-3p	TOR1AIP2	torsin 1A interacting protein 2
hsa-miR-200c-3p	TARDBP	TAR DNA binding protein
hsa-miR-200c-3p	LRIG1	leucine rich repeats and immunoglobulin like domains 1
hsa-miR-200c-3p	FAM217B	family with sequence similarity 217 member B
hsa-miR-200c-3p	WWP2	WW domain containing E3 ubiquitin protein ligase 2
hsa-miR-200c-3p	PM20D2	peptidase M20 domain containing 2
hsa-miR-200c-3p	CEBPD	CCAAT enhancer binding protein delta
hsa-miR-200c-3p	LRAT	lecithin retinol acyltransferase
hsa-miR-200c-3p	NALCN	sodium leak channel, non-selective
hsa-miR-200c-3p	NEK9	NIMA related kinase 9
hsa-miR-200c-3p	FLT4	fms related tyrosine kinase 4
hsa-miR-200c-3p	IGSF3	immunoglobulin superfamily member 3
hsa-miR-200c-3p	LDB3	LIM domain binding 3
hsa-miR-200c-3p	SNX1	sorting nexin 1
hsa-miR-200c-3p	KAT2B	lysine acetyltransferase 2B
hsa-miR-200c-3p	SCN9A	sodium voltage-gated channel alpha subunit 9
hsa-miR-200c-3p	SMARCAD1	SWI/SNF-related, matrix-associated actin-dependent regulator of chromatin, subfamily a, containing DEAD/H box 1
hsa-miR-200c-3p	GABPB1	GA binding protein transcription factor subunit beta 1
hsa-miR-200c-3p	NUP153	nucleoporin 153
hsa-miR-200c-3p	ABRA	actin binding Rho activating protein
hsa-miR-200c-3p	ERI1	exoribonuclease 1
hsa-miR-200c-3p	GABRA6	gamma-aminobutyric acid type A receptor alpha6 subunit
hsa-miR-200c-3p	TUBB3	tubulin beta 3 class III
hsa-miR-200c-3p	CPXCR1	CPX chromosome region, candidate 1

hsa-miR-200c-3p	EDNRA	endothelin receptor type A
hsa-miR-200c-3p	BDKRB2	bradykinin receptor B2
hsa-miR-200c-3p	SHISA2	shisa family member 2
hsa-miR-200c-3p	GPR85	G protein-coupled receptor 85
hsa-miR-200c-3p	TNFRSF11B	TNF receptor superfamily member 11b
hsa-miR-200c-3p	ZNF674	zinc finger protein 674
hsa-miR-200c-3p	PDPK1	3-phosphoinositide dependent protein kinase 1
hsa-miR-200c-3p	CFHR5	complement factor H related 5
hsa-miR-200c-3p	VTI1A	vesicle transport through interaction with t-SNAREs 1A
hsa-miR-200c-3p	SLC25A30	solute carrier family 25 member 30
hsa-miR-200c-3p	PARG	poly(ADP-ribose) glycohydrolase
hsa-miR-200c-3p	TMEM38B	transmembrane protein 38B
hsa-miR-200c-3p	TMEM14A	transmembrane protein 14A
hsa-miR-200c-3p	PIK3CB	phosphatidylinositol-4,5-bisphosphate 3-kinase catalytic subunit beta
hsa-miR-200c-3p	TMED4	transmembrane p24 trafficking protein 4
hsa-miR-200c-3p	HLF	HLF, PAR bZIP transcription factor
hsa-miR-200c-3p	SLC15A2	solute carrier family 15 member 2
hsa-miR-200c-3p	COL9A2	collagen type IX alpha 2 chain
hsa-miR-200c-3p	CREG1	cellular repressor of E1A stimulated genes 1
hsa-miR-200c-3p	TENT5D	terminal nucleotidyltransferase 5D
hsa-miR-200c-3p	AMACR	alpha-methylacyl-CoA racemase
hsa-miR-200c-3p	MMS22L	MMS22 like, DNA repair protein
hsa-miR-200c-3p	RWDD2A	RWD domain containing 2A
hsa-miR-200c-3p	RFX7	regulatory factor X7
hsa-miR-200c-3p	AMOTL2	angiomotin like 2
hsa-miR-200c-3p	DDX3Y	DEAD-box helicase 3 Y-linked
hsa-miR-200c-3p	KBTBD6	kelch repeat and BTB domain containing 6
hsa-miR-200c-3p	ZNF692	zinc finger protein 692
hsa-miR-200c-3p	ZNF831	zinc finger protein 831
hsa-miR-200c-3p	PJA2	praja ring finger ubiquitin ligase 2
hsa-miR-200c-3p	GPR63	G protein-coupled receptor 63
hsa-miR-200c-3p	PLEKHA8	pleckstrin homology domain containing A8
hsa-miR-200c-3p	TRAT1	T cell receptor associated transmembrane adaptor 1
hsa-miR-200c-3p	ARL6IP6	ADP ribosylation factor like GTPase 6 interacting protein 6
hsa-miR-200c-3p	STX16	syntaxin 16
hsa-miR-200c-3p	RELCH	RAB11 binding and LisH domain, coiled-coil and HEAT repeat containing
hsa-miR-200c-3p	DDX1	DEAD-box helicase 1
hsa-miR-200c-3p	SELENOK	selenoprotein K
hsa-miR-200c-3p	SNX30	sorting nexin family member 30
hsa-miR-200c-3p	DNAJC18	DnaJ heat shock protein family (Hsp40) member C18
hsa-miR-200c-3p	SHROOM4	shroom family member 4
hsa-miR-200c-3p	CDH6	cadherin 6
hsa-miR-200c-3p	ZNF568	zinc finger protein 568

hsa-miR-200c-3p	ARHGEF3	Rho guanine nucleotide exchange factor 3
hsa-miR-200c-3p	NAPB	NSF attachment protein beta
hsa-miR-200c-3p	RET	ret proto-oncogene
hsa-miR-200c-3p	RAD18	RAD18, E3 ubiquitin protein ligase
hsa-miR-200c-3p	HLA-DPA1	major histocompatibility complex, class II, DP alpha 1
hsa-miR-200c-3p	ADGRL2	adhesion G protein-coupled receptor L2
hsa-miR-200c-3p	SLC17A4	solute carrier family 17 member 4
hsa-miR-200c-3p	ELAVL4	ELAV like RNA binding protein 4
hsa-miR-200c-3p	ZBTB5	zinc finger and BTB domain containing 5
hsa-miR-200c-3p	ENAH	ENAH, actin regulator
hsa-miR-200c-3p	SLC25A24	solute carrier family 25 member 24
hsa-miR-200c-3p	ZMYM4	zinc finger MYM-type containing 4
hsa-miR-200c-3p	HNRNPU	heterogeneous nuclear ribonucleoprotein U
hsa-miR-200c-3p	CALHM5	calcium homeostasis modulator family member 5
hsa-miR-200c-3p	GDPD1	glycerophosphodiester phosphodiesterase domain containing 1
hsa-miR-200c-3p	RBFA	ribosome binding factor A
hsa-miR-200c-3p	EPM2AIP1	EPM2A interacting protein 1
hsa-miR-200c-3p	MPPED1	metallophosphoesterase domain containing 1
hsa-miR-200c-3p	RCN1	reticulocalbin 1
hsa-miR-200c-3p	AK9	adenylate kinase 9
hsa-miR-200c-3p	UXS1	UDP-glucuronate decarboxylase 1
hsa-miR-200c-3p	LMAN1	lectin, mannose binding 1
hsa-miR-200c-3p	CCSAP	centriole, cilia and spindle associated protein
hsa-miR-200c-3p	KLF10	Kruppel like factor 10
hsa-miR-200c-3p	COPS2	COP9 signalosome subunit 2
hsa-miR-200c-3p	SLAIN1	SLAIN motif family member 1
hsa-miR-200c-3p	ZDHHC15	zinc finger DHHC-type containing 15
hsa-miR-200c-3p	SCHIP1	schwannomin interacting protein 1
hsa-miR-200c-3p	MRPS35	mitochondrial ribosomal protein S35
hsa-miR-200c-3p	CDC27	cell division cycle 27
hsa-miR-200c-3p	GOSR2	golgi SNAP receptor complex member 2
hsa-miR-200c-3p	IPMK	inositol polyphosphate multikinase
hsa-miR-200c-3p	STAC	SH3 and cysteine rich domain
hsa-miR-200c-3p	KLHL3	kelch like family member 3
hsa-miR-200c-3p	USP47	ubiquitin specific peptidase 47
hsa-miR-200c-3p	TP53TG3C	TP53 target 3C
hsa-miR-200c-3p	TTC5	tetratricopeptide repeat domain 5
hsa-miR-200c-3p	EIF2S1	eukaryotic translation initiation factor 2 subunit alpha
hsa-miR-200c-3p	NOL12	nucleolar protein 12
hsa-miR-200c-3p	GPC6	glypican 6
hsa-miR-200c-3p	GPX8	glutathione peroxidase 8 (putative)
hsa-miR-200c-3p	EXOSC3	exosome component 3
hsa-miR-200c-3p	GREM2	gremlin 2, DAN family BMP antagonist
hsa-miR-200c-3p	NSMCE3	NSE3 homolog, SMC5-SMC6 complex component

hsa-miR-200c-3p	HMOX1	heme oxygenase 1
hsa-miR-200c-3p	APLP2	amyloid beta precursor like protein 2
hsa-miR-200c-3p	ATF7IP	activating transcription factor 7 interacting protein
hsa-miR-200c-3p	CALHM1	calcium homeostasis modulator 1
hsa-miR-200c-3p	CLDND1	claudin domain containing 1
hsa-miR-200c-3p	TTC33	tetratricopeptide repeat domain 33
hsa-miR-200c-3p	AVPR1A	arginine vasopressin receptor 1A
hsa-miR-200c-3p	ASB13	ankyrin repeat and SOCS box containing 13
hsa-miR-200c-3p	SAMD12	sterile alpha motif domain containing 12
hsa-miR-200c-3p	ADD3	adducin 3
hsa-miR-200c-3p	ENTPD5	ectonucleoside triphosphate diphosphohydrolase 5
hsa-miR-200c-3p	CCDC144A	coiled-coil domain containing 144A
hsa-miR-200c-3p	TTC21B	tetratricopeptide repeat domain 21B
hsa-miR-200c-3p	DGKE	diacylglycerol kinase epsilon
hsa-miR-200c-3p	JADE3	jade family PHD finger 3
hsa-miR-200c-3p	PTPN13	protein tyrosine phosphatase, non-receptor type 13
hsa-miR-200c-3p	NCBP3	nuclear cap binding subunit 3
hsa-miR-200c-3p	MYEOV	myeloma overexpressed
hsa-miR-200c-3p	RBM48	RNA binding motif protein 48
hsa-miR-200c-3p	UCHL5	ubiquitin C-terminal hydrolase L5
hsa-miR-200c-3p	ABI2	abl interactor 2
hsa-miR-200c-3p	DR1	down-regulator of transcription 1
hsa-miR-200c-3p	TAF5L	TATA-box binding protein associated factor 5 like
hsa-miR-200c-3p	NECTIN1	nectin cell adhesion molecule 1
hsa-miR-200c-3p	MAP2K5	mitogen-activated protein kinase kinase 5
hsa-miR-200c-3p	FGF9	fibroblast growth factor 9
hsa-miR-200c-3p	DNMT3A	DNA methyltransferase 3 alpha
hsa-miR-200c-3p	ZDBF2	zinc finger DBF-type containing 2
hsa-miR-200c-3p	CNDP2	carnosine dipeptidase 2
hsa-miR-200c-3p	RALB	RAS like proto-oncogene B
hsa-miR-200c-3p	RUFY2	RUN and FYVE domain containing 2
hsa-miR-200c-3p	REL	REL proto-oncogene, NF-kB subunit
hsa-miR-200c-3p	RHOA	ras homolog family member A
hsa-miR-200c-3p	RBM46	RNA binding motif protein 46
hsa-miR-200c-3p	SH3TC2	SH3 domain and tetratricopeptide repeats 2
hsa-miR-200c-3p	MPP5	membrane palmitoylated protein 5
hsa-miR-200c-3p	CCDC62	coiled-coil domain containing 62
hsa-miR-200c-3p	SLC5A3	solute carrier family 5 member 3
hsa-miR-200c-3p	PRKAA2	protein kinase AMP-activated catalytic subunit alpha 2
hsa-miR-200c-3p	HPF1	histone PARylation factor 1
hsa-miR-200c-3p	TC2N	tandem C2 domains, nuclear
hsa-miR-200c-3p	SMPX	small muscle protein X-linked
hsa-miR-200c-3p	NRK	Nik related kinase
hsa-miR-200c-3p	SLC16A1	solute carrier family 16 member 1
hsa-miR-200c-3p	MGA	MGA, MAX dimerization prote

hsa-miR-210-3p	IGF2	insulin like growth factor 2
hsa-miR-210-3p	ISCU	iron-sulfur cluster assembly enzyme
hsa-miR-210-3p	GALR2	galanin receptor 2
hsa-miR-210-3p	KMT2D	lysine methyltransferase 2D
hsa-miR-210-3p	AIFM3	apoptosis inducing factor, mitochondria associated 3
hsa-miR-210-3p	FGFRL1	fibroblast growth factor receptor like 1
hsa-miR-210-3p	NDUFA4	NDUFA4, mitochondrial complex associated
hsa-miR-210-3p	MID1IP1	MID1 interacting protein 1
hsa-miR-210-3p	BDNF	brain derived neurotrophic factor
hsa-miR-210-3p	RUNX3	runt related transcription factor 3
hsa-miR-210-3p	SHISAL2B	shisa like 2B
hsa-miR-210-3p	DENND6A	DENN domain containing 6A
hsa-miR-210-3p	GIT2	GIT ArfGAP 2
hsa-miR-210-3p	TTC13	tetratricopeptide repeat domain 13
hsa-miR-210-3p	SDF2	stromal cell derived factor 2
hsa-miR-210-3p	ACVR1B	activin A receptor type 1B
hsa-miR-210-3p	PCYT1B	phosphate cytidylyltransferase 1, choline, beta
hsa-miR-210-3p	THSD7A	thrombospondin type 1 domain containing 7A
hsa-miR-210-3p	B4GALT5	beta-1,4-galactosyltransferase 5
hsa-miR-210-3p	ARMC1	armadillo repeat containing 1
hsa-miR-210-3p	BARD1	BRCA1 associated RING domain 1
hsa-miR-210-3p	FAM222A	family with sequence similarity 222 member A
hsa-miR-210-3p	SIN3A	SIN3 transcription regulator family member A
hsa-miR-210-3p	VAMP7	vesicle associated membrane protein 7
hsa-miR-210-3p	DLX1	distal-less homeobox 1
hsa-miR-210-3p	GPD1L	glycerol-3-phosphate dehydrogenase 1 like
hsa-miR-210-3p	RRP1B	ribosomal RNA processing 1B
hsa-miR-210-3p	ZNF37A	zinc finger protein 37A
hsa-miR-210-3p	CYP4A22	cytochrome P450 family 4 subfamily A member 22
hsa-miR-210-3p	DIMT1	DIM1 dimethyladenosine transferase 1 homolog
hsa-miR-210-3p	ST6GAL2	ST6 beta-galactoside alpha-2,6-sialyltransferase 2
hsa-miR-210-3p	DCAF5	DDB1 and CUL4 associated factor 5
hsa-miR-210-3p	SLC16A14	solute carrier family 16 member 14
hsa-miR-210-3p	ATP11A	ATPase phospholipid transporting 11A
hsa-miR-210-3p	ALDH5A1	aldehyde dehydrogenase 5 family member A1
hsa-miR-210-3p	SH3BGRL	SH3 domain binding glutamate rich protein like
hsa-miR-210-3p	SLC24A2	solute carrier family 24 member 2
hsa-miR-210-3p	NPTX1	neuronal pentraxin 1
hsa-miR-210-3p	DELE1	DAP3 binding cell death enhancer 1
hsa-miR-210-3p	NCOR1	nuclear receptor corepressor 1
hsa-miR-210-3p	CORO2B	coronin 2B
hsa-miR-210-3p	ST3GAL3	ST3 beta-galactoside alpha-2,3-sialyltransferase 3
hsa-miR-210-3p	CAMTA1	calmodulin binding transcription activator 1
hsa-miR-210-3p	DHX58	DExH-box helicase 58
hsa-miR-210-3p	SCARA3	scavenger receptor class A member 3

hsa-miR-210-3p	TM4SF18	transmembrane 4 L six family member 18
hsa-miR-210-3p	WLS	Wnt ligand secretion mediator
hsa-miR-210-3p	SLC25A26	solute carrier family 25 member 26
hsa-miR-210-3p	KPNA1	karyopherin subunit alpha 1
hsa-miR-210-3p	NR1D2	nuclear receptor subfamily 1 group D member 2
hsa-miR-210-3p	PANX3	pannexin 3
hsa-miR-210-3p	FKBP1B	FKBP prolyl isomerase 1B
hsa-miR-210-3p	ELFN2	extracellular leucine rich repeat and fibronectin type III domain containing 2
hsa-miR-210-3p	MGRN1	mahogunin ring finger 1
hsa-miR-210-3p	KIAA1143	KIAA1143
hsa-miR-210-3p	PPM1K	protein phosphatase, Mg2+/Mn2+ dependent 1K
hsa-miR-210-3p	KCMF1	potassium channel modulatory factor 1
hsa-miR-210-3p	DSG1	desmoglein 1
hsa-miR-210-3p	E2F3	E2F transcription factor 3
hsa-miR-210-3p	TNPO3	transportin 3
hsa-miR-210-3p	INHBB	inhibin subunit beta B
hsa-miR-210-3p	NMNAT2	nicotinamide nucleotide adenylyltransferase 2
hsa-miR-210-3p	MIGA2	mitoguardin 2
hsa-miR-210-3p	CCDC32	coiled-coil domain containing 32
hsa-miR-210-3p	ATP2B3	ATPase plasma membrane Ca2+ transporting 3
hsa-miR-210-3p	MLC1	megalencephalic leukoencephalopathy with subcortical cysts 1
hsa-miR-210-3p	GGNBP2	gametogenetin binding protein 2
hsa-miR-210-3p	CNTNAP5	contactin associated protein like 5
hsa-miR-210-3p	SSX1	SSX family member 1
hsa-miR-210-3p	USH2A	usherin
hsa-miR-210-3p	HOXA9	homeobox A9
hsa-miR-210-3p	MYORG	myogenesis regulating glycosidase (putative)
hsa-miR-210-3p	SAMD12	sterile alpha motif domain containing 12
hsa-miR-210-3p	ESCO2	establishment of sister chromatid cohesion N-acetyltransferase 2
hsa-miR-210-3p	KLF12	Kruppel like factor 12
hsa-miR-210-3p	RAB7A	RAB7A, member RAS oncogene family
hsa-miR-210-3p	MZF1	myeloid zinc finger 1
hsa-miR-210-3p	C5orf38	chromosome 5 open reading frame 38
hsa-miR-210-3p	PIK3R5	phosphoinositide-3-kinase regulatory subunit 5
hsa-miR-210-3p	CD22	CD22 molecule
hsa-miR-210-3p	SDHD	succinate dehydrogenase complex subunit D
hsa-miR-210-3p	MPEG1	macrophage expressed 1
hsa-miR-210-3p	ADGRA1	adhesion G protein-coupled recepto
hsa-miR-221-3p	RIMS3	regulating synaptic membrane exocytosis 3
hsa-miR-221-3p	GABRA1	gamma-aminobutyric acid type A receptor alpha1 subunit
hsa-miR-221-3p	CDKN1B	cyclin dependent kinase inhibitor 1B
hsa-miR-221-3p	PAIP1	poly(A) binding protein interacting protein 1

hsa-miR-221-3p	PANK3	pantothenate kinase 3
hsa-miR-221-3p	TCF12	transcription factor 12
hsa-miR-221-3p	HECTD2	HECT domain E3 ubiquitin protein ligase 2
hsa-miR-221-3p	RFX7	regulatory factor X7
hsa-miR-221-3p	RGS6	regulator of G protein signaling 6
hsa-miR-221-3p	HMBOX1	homeobox containing 1
hsa-miR-221-3p	EIF5A2	eukaryotic translation initiation factor 5A2
hsa-miR-221-3p	KIT	KIT proto-oncogene receptor tyrosine kinase
hsa-miR-221-3p	MIDN	midnolin
hsa-miR-221-3p	EML6	EMAP like 6
hsa-miR-221-3p	CLVS2	clavesin 2
hsa-miR-221-3p	GNAI3	G protein subunit alpha i3
hsa-miR-221-3p	GRB10	growth factor receptor bound protein 10
hsa-miR-221-3p	ARHGEF38	Rho guanine nucleotide exchange factor 38
hsa-miR-221-3p	GRIK1	glutamate ionotropic receptor kainate type subunit 1
hsa-miR-221-3p	TGS1	trimethylguanosine synthase 1
hsa-miR-221-3p	RAB18	RAB18, member RAS oncogene family
hsa-miR-221-3p	ADAM22	ADAM metallopeptidase domain 22
hsa-miR-221-3p	DENND1B	DENN domain containing 1B
hsa-miR-221-3p	FNIP2	folliculin interacting protein 2
hsa-miR-221-3p	MYLIP	myosin regulatory light chain interacting protein
hsa-miR-221-3p	VAPB	VAMP associated protein B and C
hsa-miR-221-3p	FNDC3A	fibronectin type III domain containing 3A
hsa-miR-221-3p	LOC100506388	uncharacterized LOC100506388
hsa-miR-221-3p	ZNF91	zinc finger protein 91
hsa-miR-221-3p	WDR35	WD repeat domain 35
hsa-miR-221-3p	C3orf70	chromosome 3 open reading frame 70
hsa-miR-221-3p	MIER3	MIER family member 3
hsa-miR-221-3p	GALNT3	polypeptide N-acetylgalactosaminyltransferase 3
hsa-miR-221-3p	NRK	Nik related kinase
hsa-miR-221-3p	TFG	TRK-fused gene
hsa-miR-221-3p	PCMTD1	protein-L-isoaspartate (D-aspartate) O-methyltransferase domain containing 1
hsa-miR-221-3p	POGZ	pogo transposable element derived with ZNF domain
hsa-miR-221-3p	RSBN1L	round spermatid basic protein 1 like
hsa-miR-221-3p	DCUN1D1	defective in cullin neddylation 1 domain containing 1
hsa-miR-221-3p	CHSY1	chondroitin sulfate synthase 1
hsa-miR-221-3p	AGTPBP1	ATP/GTP binding protein 1
hsa-miR-221-3p	SEC62	SEC62 homolog, preprotein translocation factor
hsa-miR-221-3p	RIT2	Ras like without CAAX 2
hsa-miR-221-3p	ERBB4	erb-b2 receptor tyrosine kinase 4
hsa-miR-221-3p	DMRT3	doublesex and mab-3 related transcription factor 3
hsa-miR-221-3p	PPP3R1	protein phosphatase 3 regulatory subunit B, alpha
hsa-miR-221-3p	CCN1	cellular communication network factor 1
hsa-miR-221-3p	NAA25	N(alpha)-acetyltransferase 25, NatB auxiliary subunit

hsa-miR-221-3p	ARF4	ADP ribosylation factor 4
hsa-miR-221-3p	CDH2	cadherin 2
hsa-miR-221-3p	ZFPM2	zinc finger protein, FOG family member 2
hsa-miR-221-3p	HNRNPH3	heterogeneous nuclear ribonucleoprotein H3
hsa-miR-221-3p	KIF16B	kinesin family member 16B
hsa-miR-221-3p	KIF20A	kinesin family member 20A
hsa-miR-221-3p	FMR1	fragile X mental retardation 1
hsa-miR-221-3p	ATXN1	ataxin 1
hsa-miR-221-3p	FOXN2	forkhead box N2
hsa-miR-221-3p	MRAP2	melanocortin 2 receptor accessory protein 2
hsa-miR-221-3p	CBWD5	COBW domain containing 5
hsa-miR-221-3p	CASZ1	castor zinc finger 1
hsa-miR-221-3p	CBWD1	COBW domain containing 1
hsa-miR-221-3p	SUN2	Sad1 and UNC84 domain containing 2
hsa-miR-221-3p	CBWD6	COBW domain containing 6
hsa-miR-221-3p	DCAF12	DDB1 and CUL4 associated factor 12
hsa-miR-221-3p	VASH1	vasohibin 1
hsa-miR-221-3p	TMCC1	transmembrane and coiled-coil domain family 1
hsa-miR-221-3p	TP53BP2	tumor protein p53 binding protein 2
hsa-miR-221-3p	CBWD3	COBW domain containing 3
hsa-miR-221-3p	ETV3	ETS variant 3
hsa-miR-221-3p	MARK1	microtubule affinity regulating kinase 1
hsa-miR-221-3p	PHACTR4	phosphatase and actin regulator 4
hsa-miR-221-3p	SYT10	synaptotagmin 10
hsa-miR-221-3p	PIK3R1	phosphoinositide-3-kinase regulatory subunit 1
hsa-miR-221-3p	CBWD2	COBW domain containing 2
hsa-miR-221-3p	APOLD1	apolipoprotein L domain containing 1
hsa-miR-221-3p	LHFPL2	LHFPL tetraspan subfamily member 2
hsa-miR-221-3p	FERMT2	fermitin family member 2
hsa-miR-221-3p	KLF7	Kruppel like factor 7
hsa-miR-221-3p	CXCL12	C-X-C motif chemokine ligand 12
hsa-miR-221-3p	CCDC18	coiled-coil domain containing 18
hsa-miR-221-3p	NXPH1	neurexophilin 1
hsa-miR-221-3p	BICDL1	BICD family like cargo adaptor 1
hsa-miR-221-3p	BCL2L11	BCL2 like 11
hsa-miR-221-3p	BRWD1	bromodomain and WD repeat domain containing 1
hsa-miR-221-3p	C6	complement C6
hsa-miR-221-3p	RFX8	RFX family member 8, lacking RFX DNA binding domain
hsa-miR-221-3p	C6orf118	chromosome 6 open reading frame 118
hsa-miR-221-3p	PPP2R2A	protein phosphatase 2 regulatory subunit Balpha
hsa-miR-221-3p	MARF1	meiosis regulator and mRNA stability factor 1
hsa-miR-221-3p	FAM214A	family with sequence similarity 214 member A
hsa-miR-221-3p	PIEZO2	piezo type mechanosensitive ion channel component 2
hsa-miR-221-3p	DPH6	diphthamine biosynthesis 6
hsa-miR-221-3p	PLCL2	phospholipase C like 2

hsa-miR-221-3p	ZNF615	zinc finger protein 615
hsa-miR-221-3p	GABRG1	gamma-aminobutyric acid type A receptor gamma1 subunit
hsa-miR-221-3p	CDK19	cyclin dependent kinase 19
hsa-miR-221-3p	HIPK1	homeodomain interacting protein kinase 1
hsa-miR-221-3p	GUCY1A2	guanylate cyclase 1 soluble subunit alpha 2
hsa-miR-221-3p	WDR47	WD repeat domain 47
hsa-miR-221-3p	SESN3	sestrin 3
hsa-miR-221-3p	USP27X	ubiquitin specific peptidase 27 X-linked
hsa-miR-221-3p	AP3B2	adaptor related protein complex 3 subunit beta 2
hsa-miR-221-3p	ESR1	estrogen receptor 1
hsa-miR-221-3p	TUB	tubby bipartite transcription factor
hsa-miR-221-3p	ASPA	aspartoacylase
hsa-miR-221-3p	SYBU	syntabulin
hsa-miR-221-3p	CTDSPL2	CTD small phosphatase like 2
hsa-miR-221-3p	SBK1	SH3 domain binding kinase 1
hsa-miR-221-3p	DDIT4	DNA damage inducible transcript 4
hsa-miR-221-3p	CLRN1	clarin 1
hsa-miR-221-3p	SLC4A7	solute carrier family 4 member 7
hsa-miR-221-3p	TLE3	TLE family member 3, transcriptional corepressor
hsa-miR-221-3p	RFX3	regulatory factor X3
hsa-miR-221-3p	EIF3J	eukaryotic translation initiation factor 3 subunit J
hsa-miR-221-3p	ANKRD12	ankyrin repeat domain 12
hsa-miR-221-3p	CREBL2	cAMP responsive element binding protein like 2
hsa-miR-221-3p	TSPAN13	tetraspanin 13
hsa-miR-221-3p	ZMYM2	zinc finger MYM-type containing 2
hsa-miR-221-3p	DPP8	dipeptidyl peptidase 8
hsa-miR-221-3p	AQP3	aquaporin 3 (Gill blood group)
hsa-miR-221-3p	SHLD2	shieldin complex subunit 2
hsa-miR-221-3p	MIA3	MIA SH3 domain ER export factor 3
hsa-miR-221-3p	IRX5	iroquois homeobox 5
hsa-miR-221-3p	CLGN	calmegin
hsa-miR-221-3p	PGPEP1L	pyroglutamyl-peptidase I like
hsa-miR-221-3p	KIAA1841	KIAA1841
hsa-miR-221-3p	BEND4	BEN domain containing 4
hsa-miR-221-3p	LYPLA1	lysophospholipase 1
hsa-miR-221-3p	SEMA3C	semaphorin 3C
hsa-miR-221-3p	PAF1	PAF1 homolog, Paf1/RNA polymerase II complex component
hsa-miR-221-3p	TRPC3	transient receptor potential cation channel subfamily C member 3
hsa-miR-221-3p	RNPS1	RNA binding protein with serine rich domain 1
hsa-miR-221-3p	NIPAL4	NIPA like domain containing 4
hsa-miR-221-3p	UBE2J1	ubiquitin conjugating enzyme E2 J1
hsa-miR-221-3p	ZNF385A	zinc finger protein 385A
hsa-miR-221-3p	TSC22D3	TSC22 domain family member 3

hsa-miR-221-3p	ZNF181	zinc finger protein 181
hsa-miR-221-3p	BEAN1	brain expressed associated with NEDD4 1
hsa-miR-221-3p	KDR	kinase insert domain receptor
hsa-miR-221-3p	GPBP1	GC-rich promoter binding protein 1
hsa-miR-221-3p	SNX4	sorting nexin 4
hsa-miR-221-3p	L3MBTL1	L3MBTL1, histone methyl-lysine binding protein
hsa-miR-221-3p	AIDA	axin interactor, dorsalization associated
hsa-miR-221-3p	GDF9	growth differentiation factor 9
hsa-miR-221-3p	FGF14	fibroblast growth factor 14
hsa-miR-221-3p	NYAP2	neuronal tyrosine-phosphorylated phosphoinositide-3-kinase adaptor 2
hsa-miR-221-3p	MYBL1	MYB proto-oncogene like 1
hsa-miR-221-3p	BRWD3	bromodomain and WD repeat domain containing 3
hsa-miR-221-3p	POLR3E	RNA polymerase III subunit E
hsa-miR-221-3p	IRF2	interferon regulatory factor 2
hsa-miR-221-3p	ATP1B1	ATPase Na+/K+ transporting subunit beta 1
hsa-miR-221-3p	TUBA1A	tubulin alpha 1a
hsa-miR-221-3p	SNRNP48	small nuclear ribonucleoprotein U11/U12 subunit 48
hsa-miR-221-3p	RNF4	ring finger protein 4
hsa-miR-221-3p	DNAJC6	DnaJ heat shock protein family (Hsp40) member C6
hsa-miR-221-3p	FXN	frataxin
hsa-miR-221-3p	NAP1L5	nucleosome assembly protein 1 like 5
hsa-miR-221-3p	BBC3	BCL2 binding component 3
hsa-miR-221-3p	WNK3	WNK lysine deficient protein kinase 3
hsa-miR-221-3p	SLC2A13	solute carrier family 2 member 13
hsa-miR-221-3p	PLXNC1	plexin C1
hsa-miR-221-3p	LBR	lamin B receptor
hsa-miR-221-3p	RDX	radixin
hsa-miR-221-3p	SYNCRIP	synaptotagmin binding cytoplasmic RNA interacting protein
hsa-miR-221-3p	TDRP	testis development related protein
hsa-miR-221-3p	SNAP29	synaptosome associated protein 29
hsa-miR-221-3p	ATAD2B	ATPase family, AAA domain containing 2B
hsa-miR-221-3p	AKAP5	A-kinase anchoring protein 5
hsa-miR-221-3p	RBP2	retinol binding protein 2
hsa-miR-221-3p	INA	internexin neuronal intermediate filament protein alpha
hsa-miR-221-3p	LUZP2	leucine zipper protein 2
hsa-miR-221-3p	SMARCA5	SWI/SNF related, matrix associated, actin dependent regulator of chromatin, subfamily a, member 5
hsa-miR-221-3p	SVIP	small VCP interacting protein
hsa-miR-221-3p	ZFYVE16	zinc finger FYVE-type containing 16
hsa-miR-221-3p	PMEPA1	prostate transmembrane protein, androgen induced 1
hsa-miR-221-3p	ZFP36L2	ZFP36 ring finger protein like 2
hsa-miR-221-3p	ZNF624	zinc finger protein 624
hsa-miR-221-3p	SEC24C	SEC24 homolog C, COPII coat complex component
hsa-miR-221-3p	RALA	RAS like proto-oncogene A

hsa-miR-221-3p	SLC6A4	solute carrier family 6 member 4
hsa-miR-221-3p	RAB1A	RAB1A, member RAS oncogene family
hsa-miR-221-3p	TNRC6C	trinucleotide repeat containing 6C
hsa-miR-221-3p	HSPA8	heat shock protein family A (Hsp70) member 8
hsa-miR-221-3p	CASR	calcium sensing receptor
hsa-miR-221-3p	PRRC2B	proline rich coiled-coil 2B
hsa-miR-221-3p	NFYB	nuclear transcription factor Y subunit beta
hsa-miR-221-3p	NTF3	neurotrophin 3
hsa-miR-221-3p	CMTM4	CKLF like MARVEL transmembrane domain containing 4
hsa-miR-221-3p	PARP9	poly(ADP-ribose) polymerase family member 9
hsa-miR-221-3p	NANOS1	nanos C2HC-type zinc finger 1
hsa-miR-221-3p	ANGPTL2	angiopoietin like 2
hsa-miR-221-3p	AMMECR1	Alport syndrome, mental retardation, midface hypoplasia and elliptocytosis chromosomal region gene 1
hsa-miR-221-3p	OSTM1	osteoclastogenesis associated transmembrane protein 1
hsa-miR-221-3p	SOX10	SRY-box 10
hsa-miR-221-3p	DIRAS3	DIRAS family GTPase 3
hsa-miR-221-3p	ADAM17	ADAM metallopeptidase domain 17
hsa-miR-221-3p	LRFN2	leucine rich repeat and fibronectin type III domain containing 2
hsa-miR-221-3p	ZNF275	zinc finger protein 275
hsa-miR-221-3p	NFATC3	nuclear factor of activated T cells 3
hsa-miR-221-3p	PTPN4	protein tyrosine phosphatase, non-receptor type 4
hsa-miR-221-3p	ANKRD52	ankyrin repeat domain 52
hsa-miR-221-3p	NFATC2	nuclear factor of activated T cells 2
hsa-miR-221-3p	MAP4K5	mitogen-activated protein kinase kinase kinase kinase 5
hsa-miR-221-3p	KMT2A	lysine methyltransferase 2A
hsa-miR-221-3p	PLCXD3	phosphatidylinositol specific phospholipase C X domain containing 3
hsa-miR-221-3p	ZFAND5	zinc finger AN1-type containing 5
hsa-miR-221-3p	KCNH8	potassium voltage-gated channel subfamily H member 8
hsa-miR-221-3p	NDST3	N-deacetylase and N-sulfotransferase 3
hsa-miR-221-3p	SLC30A6	solute carrier family 30 member 6
hsa-miR-221-3p	NCKAP5	NCK associated protein 5
hsa-miR-221-3p	ANXA3	annexin A3
hsa-miR-221-3p	EOGT	EGF domain specific O-linked N-acetylglucosamine transferase
hsa-miR-221-3p	ZBTB5	zinc finger and BTB domain containing 5
hsa-miR-221-3p	BMF	Bcl2 modifying factor
hsa-miR-221-3p	CUX2	cut like homeobox 2
hsa-miR-221-3p	PRDM1	PR/SET domain 1
hsa-miR-221-3p	FRY	FRY microtubule binding protein
hsa-miR-221-3p	CLDN11	claudin 11
hsa-miR-221-3p	PLPPR1	phospholipid phosphatase related 1
hsa-miR-221-3p	PPDPFL	pancreatic progenitor cell differentiation and proliferation factor like

hsa-miR-221-3p	CNR1	cannabinoid receptor 1
hsa-miR-221-3p	TRAF3IP2	TRAF3 interacting protein 2
hsa-miR-221-3p	ITGB3	integrin subunit beta 3
hsa-miR-221-3p	ZNF629	zinc finger protein 629
hsa-miR-221-3p	ZFP30	ZFP30 zinc finger protein
hsa-miR-221-3p	SNCB	synuclein beta
hsa-miR-221-3p	CPEB3	cytoplasmic polyadenylation element binding protein 3
hsa-miR-221-3p	GALNT18	polypeptide N-acetylgalactosaminyltransferase 18
hsa-miR-221-3p	PRICKLE2	prickle planar cell polarity protein 2
hsa-miR-221-3p	MEGF9	multiple EGF like domains 9
hsa-miR-221-3p	PAIP2	poly(A) binding protein interacting protein 2
hsa-miR-221-3p	PCDHA1	protocadherin alpha 1
hsa-miR-221-3p	CBFB	core-binding factor subunit beta
hsa-miR-221-3p	C11orf87	chromosome 11 open reading frame 87
hsa-miR-221-3p	PCDHA4	protocadherin alpha 4
hsa-miR-221-3p	PCDHA7	protocadherin alpha 7
hsa-miR-221-3p	PCDHA3	protocadherin alpha 3
hsa-miR-221-3p	PCDHA12	protocadherin alpha 12
hsa-miR-221-3p	GBX2	gastrulation brain homeobox 2
hsa-miR-221-3p	ZNF704	zinc finger protein 704
hsa-miR-221-3p	C6orf120	chromosome 6 open reading frame 120
hsa-miR-221-3p	PCDHA11	protocadherin alpha 11
hsa-miR-221-3p	PCDHA2	protocadherin alpha 2
hsa-miR-221-3p	DCUN1D4	defective in cullin neddylation 1 domain containing 4
hsa-miR-221-3p	FBXO47	F-box protein 47
hsa-miR-221-3p	XIRP2	xin actin binding repeat containing 2
hsa-miR-221-3p	PCDHA6	protocadherin alpha 6
hsa-miR-221-3p	MAGI1	membrane associated guanylate kinase, WW and PDZ domain containing 1
hsa-miR-221-3p	PCDHA5	protocadherin alpha 5
hsa-miR-221-3p	ZKSCAN8	zinc finger with KRAB and SCAN domains 8
hsa-miR-221-3p	PCDHA10	protocadherin alpha 10
hsa-miR-221-3p	PCDHAC1	protocadherin alpha subfamily C, 1
hsa-miR-221-3p	PCDHA8	protocadherin alpha 8
hsa-miR-221-3p	PCDHA13	protocadherin alpha 13
hsa-miR-221-3p	PCDHAC2	protocadherin alpha subfamily C, 2
hsa-miR-221-3p	CPNE8	copine 8
hsa-miR-221-3p	PPARGC1A	PPARG coactivator 1 alpha
hsa-miR-221-3p	TMEM132C	transmembrane protein 132C
hsa-miR-221-3p	ABHD3	abhydrolase domain containing 3
hsa-miR-221-3p	MPZL1	myelin protein zero like 1
hsa-miR-221-3p	VGLL4	vestigial like family member 4
hsa-miR-221-3p	SRD5A3	steroid 5 alpha-reductase 3
hsa-miR-221-3p	RORB	RAR related orphan receptor B
hsa-miR-221-3p	SCD5	stearoyl-CoA desaturase 5

hsa-miR-221-3p	RALGAPA1	Ral GTPase activating protein catalytic alpha subunit 1
hsa-miR-221-3p	MRPS7	mitochondrial ribosomal protein S7
hsa-miR-221-3p	FANCD2	FA complementation group D2
hsa-miR-221-3p	CLDND1	claudin domain containing 1
hsa-miR-221-3p	SYCE2	synaptonemal complex central element protein 2
hsa-miR-221-3p	YWHAG	tyrosine 3-monooxygenase/tryptophan 5-monooxygenase activation protein gamma
hsa-miR-221-3p	TGOLN2	trans-golgi network protein 2
hsa-miR-221-3p	KLC1	kinesin light chain 1
hsa-miR-221-3p	ABCA8	ATP binding cassette subfamily A member 8
hsa-miR-221-3p	ZNF93	zinc finger protein 93
hsa-miR-221-3p	SGIP1	SH3 domain GRB2 like endophilin interacting protein 1
hsa-miR-221-3p	PDIK1L	PDLIM1 interacting kinase 1 like
hsa-miR-221-3p	ARAP2	ArfGAP with RhoGAP domain, ankyrin repeat and PH domain 2
hsa-miR-221-3p	WSB2	WD repeat and SOCS box containing 2
hsa-miR-221-3p	SPART	spartin
hsa-miR-221-3p	PTBP3	polypyrimidine tract binding protein 3
hsa-miR-221-3p	NIPBL	NIPBL, cohesin loading factor
hsa-miR-221-3p	NOVA1	NOVA alternative splicing regulator 1
hsa-miR-221-3p	ZNF74	zinc finger protein 74
hsa-miR-221-3p	GAB1	GRB2 associated binding protein 1
hsa-miR-221-3p	GTF2E1	general transcription factor IIE subunit 1
hsa-miR-221-3p	CAMTA1	calmodulin binding transcription activator 1
hsa-miR-221-3p	ONECUT2	one cut homeobox 2
hsa-miR-221-3p	PLSCR4	phospholipid scramblase 4
hsa-miR-221-3p	SERPINB2	serpin family B member 2
hsa-miR-221-3p	EVI2A	ecotropic viral integration site 2A
hsa-miR-221-3p	PDS5A	PDS5 cohesin associated factor A
hsa-miR-221-3p	TLNRD1	talin rod domain containing 1
hsa-miR-221-3p	DYNC1LI2	dynein cytoplasmic 1 light intermediate chain 2
hsa-miR-221-3p	MAN2A1	mannosidase alpha class 2A member 1
hsa-miR-221-3p	AGPS	alkylglycerone phosphate synthase
hsa-miR-221-3p	TBXT	T-box transcription factor T
hsa-miR-221-3p	DBT	dihydrolipoamide branched chain transacylase E2
hsa-miR-221-3p	ENAH	ENAH, actin regulator
hsa-miR-221-3p	LRRCC1	leucine rich repeat and coiled-coil centrosomal protein 1
hsa-miR-221-3p	CREBZF	CREB/ATF bZIP transcription factor
hsa-miR-221-3p	CDKN2AIP	CDKN2A interacting protein
hsa-miR-221-3p	CYLD	CYLD lysine 63 deubiquitinase
hsa-miR-221-3p	RBM24	RNA binding motif protein 24
hsa-miR-221-3p	KIAA0586	KIAA0586
hsa-miR-221-3p	ARHGEF7	Rho guanine nucleotide exchange factor 7
hsa-miR-221-3p	ITPR2	inositol 1,4,5-trisphosphate receptor type 2
hsa-miR-221-3p	KPNA2	karyopherin subunit alpha 2

hsa-miR-221-3p	ZNF25	zinc finger protein 25
hsa-miR-221-3p	PDLIM2	PDZ and LIM domain 2
hsa-miR-221-3p	TOX	thymocyte selection associated high mobility group box
hsa-miR-221-3p	PRKAB2	protein kinase AMP-activated non-catalytic subunit beta 2
hsa-miR-221-3p	NSMCE4A	NSE4 homolog A, SMC5-SMC6 complex component
hsa-miR-221-3p	SEC24B	SEC24 homolog B, COPII coat complex component
hsa-miR-221-3p	TRPS1	transcriptional repressor GATA binding 1
hsa-miR-221-3p	CLIC2	chloride intracellular channel 2
hsa-miR-221-3p	NAP1L1	nucleosome assembly protein 1 like 1
hsa-miR-221-3p	LRRTM2	leucine rich repeat transmembrane neuronal 2
hsa-miR-221-3p	IL17RB	interleukin 17 receptor B
hsa-miR-221-3p	SEMA6D	semaphorin 6D
hsa-miR-221-3p	HNRNPA0	heterogeneous nuclear ribonucleoprotein A0
hsa-miR-221-3p	GJC3	gap junction protein gamma 3
hsa-miR-221-3p	CYREN	cell cycle regulator of NHEJ
hsa-miR-221-3p	SOCS3	suppressor of cytokine signaling 3
hsa-miR-221-3p	CYP4X1	cytochrome P450 family 4 subfamily X member 1
hsa-miR-221-3p	KPNA1	karyopherin subunit alpha 1
hsa-miR-221-3p	ANKRD10	ankyrin repeat domain 10
hsa-miR-221-3p	NLK	nemo like kinase
hsa-miR-221-3p	MEX3A	mex-3 RNA binding family member A
hsa-miR-221-3p	HLTF	helicase like transcription factor
hsa-miR-221-3p	IFIT2	interferon induced protein with tetratricopeptide repeats 2
hsa-miR-221-3p	ANKS1B	ankyrin repeat and sterile alpha motif domain containing 1B
hsa-miR-221-3p	UGT2B15	UDP glucuronosyltransferase family 2 member B15
hsa-miR-221-3p	CHD7	chromodomain helicase DNA binding protein 7
hsa-miR-221-3p	IGF2BP2	insulin like growth factor 2 mRNA binding protein 2
hsa-miR-221-3p	YTHDF3	YTH N6-methyladenosine RNA binding protein 3
hsa-miR-221-3p	NGRN	neugrin, neurite outgrowth associated
hsa-miR-221-3p	COBLL1	cordon-bleu WH2 repeat protein like 1
hsa-miR-221-3p	CEP41	centrosomal protein 41
hsa-miR-221-3p	ASB4	ankyrin repeat and SOCS box containing 4
hsa-miR-221-3p	PDCD10	programmed cell death 10
hsa-miR-221-3p	MASTL	microtubule associated serine/threonine kinase like
hsa-miR-221-3p	RUNX2	runt related transcription factor 2
hsa-miR-221-3p	TRABD2B	TraB domain containing 2B
hsa-miR-221-3p	PTPRZ1	protein tyrosine phosphatase, receptor type Z1
hsa-miR-221-3p	FAT2	FAT atypical cadherin 2
hsa-miR-221-3p	FAM160B1	family with sequence similarity 160 member B1
hsa-miR-221-3p	KANK4	KN motif and ankyrin repeat domains 4
hsa-miR-221-3p	ZNF652	zinc finger protein 652
hsa-miR-221-3p	CD164	CD164 molecule
hsa-miR-221-3p	NRG1	neuregulin 1

hsa-miR-221-3p	GNB1	G protein subunit beta 1
hsa-miR-221-3p	CEP70	centrosomal protein 70
hsa-miR-221-3p	CCSAP	centriole, cilia and spindle associated protein
hsa-miR-221-3p	KHDRBS2	KH RNA binding domain containing, signal transduction associated 2
hsa-miR-221-3p	ERCC4	ERCC excision repair 4, endonuclease catalytic subunit
hsa-miR-221-3p	ACVR2B	activin A receptor type 2B
hsa-miR-221-3p	RASSF8	Ras association domain family member 8
hsa-miR-221-3p	RNF20	ring finger protein 20
hsa-miR-221-3p	ALDH16A1	aldehyde dehydrogenase 16 family member A1
hsa-miR-221-3p	SPTBN1	spectrin beta, non-erythrocytic 1
hsa-miR-221-3p	ANTXR2	ANTXR cell adhesion molecule 2
hsa-miR-221-3p	ANKHD1	ankyrin repeat and KH domain containing 1
hsa-miR-221-3p	SLC4A4	solute carrier family 4 member 4
hsa-miR-221-3p	ZNF3	zinc finger protein 3
hsa-miR-221-3p	CALM1	calmodulin 1
hsa-miR-221-3p	BCL2L14	BCL2 like 14
hsa-miR-221-3p	TYMSOS	TYMS opposite strand
hsa-miR-221-3p	CARF	calcium responsive transcription factor
hsa-miR-221-3p	USP6NL	USP6 N-terminal like
hsa-miR-221-3p	ZC3H13	zinc finger CCCH-type containing 13
hsa-miR-221-3p	DLD	dihydrolipoamide dehydrogenase
hsa-miR-221-3p	FBXO28	F-box protein 28
hsa-miR-221-3p	MON2	MON2 homolog, regulator of endosome-to-Golgi trafficking
hsa-miR-221-3p	STN1	STN1, CST complex subunit
hsa-miR-221-3p	ETS1	ETS proto-oncogene 1, transcription factor
hsa-miR-221-3p	CACNB4	calcium voltage-gated channel auxiliary subunit beta 4
hsa-miR-221-3p	PDCD6IP	programmed cell death 6 interacting protein
hsa-miR-221-3p	GOLGA1	golgin A1
hsa-miR-221-3p	ZFHX3	zinc finger homeobox 3
hsa-miR-221-3p	TMEM132B	transmembrane protein 132B
hsa-miR-221-3p	C22orf39	chromosome 22 open reading frame 39
hsa-miR-221-3p	CCT5	chaperonin containing TCP1 subunit 5
hsa-miR-221-3p	KSR1	kinase suppressor of ras 1
hsa-miR-221-3p	NUFIP2	nuclear FMR1 interacting protein 2
hsa-miR-221-3p	AGFG1	ArfGAP with FG repeats 1
hsa-miR-221-3p	TCF7L2	transcription factor 7 like 2
hsa-miR-221-3p	PPP4R2	protein phosphatase 4 regulatory subunit 2
hsa-miR-221-3p	STK24	serine/threonine kinase 24
hsa-miR-221-3p	CDON	cell adhesion associated, oncogene regulated
hsa-miR-221-3p	FAM222B	family with sequence similarity 222 member B
hsa-miR-221-3p	DGKH	diacylglycerol kinase eta
hsa-miR-221-3p	HDLBP	high density lipoprotein binding protein
hsa-miR-221-3p	SHANK2	SH3 and multiple ankyrin repeat domains 2

hsa-miR-221-3p	CFAP161	cilia and flagella associated protein 161
hsa-miR-221-3p	CRHBP	corticotropin releasing hormone binding protein
hsa-miR-221-3p	CDV3	CDV3 homolog
hsa-miR-221-3p	URI1	URI1, prefoldin like chaperone
hsa-miR-221-3p	CD47	CD47 molecule
hsa-miR-221-3p	SPPL3	signal peptide peptidase like 3
hsa-miR-221-3p	CD4	CD4 molecule
hsa-miR-221-3p	LRRC19	leucine rich repeat containing 19
hsa-miR-221-3p	EMX2	empty spiracles homeobox 2
hsa-miR-221-3p	PRDM11	PR/SET domain 11
hsa-miR-221-3p	PTPRR	protein tyrosine phosphatase, receptor type R
hsa-miR-221-3p	IGDCC4	immunoglobulin superfamily DCC subclass member 4
hsa-miR-221-3p	CDKN2B	cyclin dependent kinase inhibitor 2B
hsa-miR-221-3p	TMEM25	transmembrane protein 25
hsa-miR-221-3p	CAMK1D	calcium/calmodulin dependent protein kinase ID
hsa-miR-221-3p	MGAT4A	alpha-1,3-mannosyl-glycoprotein 4-beta-N-acetylglucosaminyltransferase A
hsa-miR-221-3p	ZFP90	ZFP90 zinc finger protein
hsa-miR-221-3p	ERMN	ermin
hsa-miR-221-3p	RPS3	ribosomal protein S3
hsa-miR-221-3p	CCP110	centriolar coiled-coil protein 110
hsa-miR-221-3p	NBPF3	NBPF member 3
hsa-miR-221-3p	CRKL	CRK like proto-oncogene, adaptor protein
hsa-miR-221-3p	REV3L	REV3 like, DNA directed polymerase zeta catalytic subunit
hsa-miR-221-3p	STYX	serine/threonine/tyrosine interacting protein
hsa-miR-221-3p	ZNF83	zinc finger protein 83
hsa-miR-221-3p	ST8SIA1	ST8 alpha-N-acetyl-neuraminide alpha-2,8-sialyltransferase 1
hsa-miR-221-3p	SOD2	superoxide dismutase 2
hsa-miR-221-3p	sept-14	septin 14
hsa-miR-221-3p	GPD2	glycerol-3-phosphate dehydrogenase 2
hsa-miR-221-3p	HIPK2	homeodomain interacting protein kinase 2
hsa-miR-221-3p	HOXC10	homeobox C10
hsa-miR-221-3p	STOX2	storkhead box 2
hsa-miR-221-3p	PSAP	prosaposin
hsa-miR-221-3p	DIRAS2	DIRAS family GTPase 2
hsa-miR-221-3p	C2CD4A	C2 calcium dependent domain containing 4A
hsa-miR-221-3p	SLC26A3	solute carrier family 26 member 3
hsa-miR-221-3p	DLG2	discs large MAGUK scaffold protein 2
hsa-miR-221-3p	PRDM2	PR/SET domain 2
hsa-miR-221-3p	C18orf54	chromosome 18 open reading frame 54
hsa-miR-221-3p	CRX	cone-rod homeobox
hsa-miR-221-3p	CAVIN3	caveolae associated protein 3
hsa-miR-221-3p	STK38L	serine/threonine kinase 38 like
hsa-miR-221-3p	CCDC126	coiled-coil domain containing 126

hsa-miR-221-3p	GLMN	glomulin, FKBP associated protein
hsa-miR-221-3p	LGI2	leucine rich repeat LGI family member 2
hsa-miR-221-3p	KBTBD11	kelch repeat and BTB domain containing 11
hsa-miR-221-3p	PLEKHA2	pleckstrin homology domain containing A2
hsa-miR-221-3p	SLITRK5	SLIT and NTRK like family member 5
hsa-miR-221-3p	THBS1	thrombospondin 1
hsa-miR-221-3p	PCDHA9	protocadherin alpha 9
hsa-miR-221-3p	GLS	glutaminase
hsa-miR-221-3p	CCDC148	coiled-coil domain containing 148
hsa-miR-221-3p	SLC10A7	solute carrier family 10 member 7
hsa-miR-221-3p	MSL2	MSL complex subunit 2
hsa-miR-221-3p	MBD2	methyl-CpG binding domain protein 2
hsa-miR-221-3p	PLPP3	phospholipid phosphatase 3
hsa-miR-221-3p	TMEM237	transmembrane protein 237
hsa-miR-221-3p	PROM2	prominin 2
hsa-miR-221-3p	UBE2V1	ubiquitin conjugating enzyme E2 V1
hsa-miR-221-3p	MAPK10	mitogen-activated protein kinase 10
hsa-miR-221-3p	ADIPOR1	adiponectin receptor 1
hsa-miR-221-3p	TMEM167A	transmembrane protein 167A
hsa-miR-221-3p	KCNQ3	potassium voltage-gated channel subfamily Q member 3
hsa-miR-221-3p	LDHAL6B	lactate dehydrogenase A like 6B
hsa-miR-221-3p	ANKIB1	ankyrin repeat and IBR domain containing 1
hsa-miR-221-3p	ECPAS	Ecm29 proteasome adaptor and scaffold
hsa-miR-221-3p	MRE11	MRE11 homolog, double strand break repair nuclease
hsa-miR-221-3p	DCAF7	DDB1 and CUL4 associated factor 7
hsa-miR-221-3p	ZNF547	zinc finger protein 547
hsa-miR-221-3p	GRM1	glutamate metabotropic receptor 1
hsa-miR-221-3p	GPM6A	glycoprotein M6A
hsa-miR-221-3p	BRD1	bromodomain containing 1
hsa-miR-221-3p	FBN2	fibrillin 2
hsa-miR-221-3p	SLC25A37	solute carrier family 25 member 37
hsa-miR-221-3p	TMSB15B	thymosin beta 15B
hsa-miR-221-3p	CSTF2T	cleavage stimulation factor subunit 2 tau variant
hsa-miR-221-3p	RECK	reversion inducing cysteine rich protein with kazal motifs
hsa-miR-221-3p	TBC1D22B	TBC1 domain family member 22B
hsa-miR-221-3p	SH3PXD2B	SH3 and PX domains 2B
hsa-miR-221-3p	MYO10	myosin X
hsa-miR-221-3p	ACADM	acyl-CoA dehydrogenase medium chain
hsa-miR-221-3p	PRUNE1	prune exopolyphosphatase 1
hsa-miR-221-3p	MRAP	melanocortin 2 receptor accessory protein
hsa-miR-221-3p	PALM2	paralemmin 2
hsa-miR-221-3p	IFRD1	interferon related developmental regulator 1
hsa-miR-221-3p	CDK8	cyclin dependent kinase 8
hsa-miR-221-3p	ACTC1	actin, alpha, cardiac muscle 1
hsa-miR-221-3p	CNPY2	canopy FGF signaling regulator 2

hsa-miR-221-3p	MAGEL2	MAGE family member L2
hsa-miR-221-3p	TMEM165	transmembrane protein 165
hsa-miR-221-3p	AADAC	arylacetamide deacetylase
hsa-miR-221-3p	PLXDC2	plexin domain containing 2
hsa-miR-221-3p	SKP1	S-phase kinase associated protein 1
hsa-miR-221-3p	NDFIP1	Nedd4 family interacting protein 1
hsa-miR-221-3p	CTTN	cortactin
hsa-miR-221-3p	RETREG1	reticulophagy regulator 1
hsa-miR-221-3p	CFHR5	complement factor H related 5
hsa-miR-221-3p	HLA-F	major histocompatibility complex, class I, F
hsa-miR-221-3p	C16orf45	chromosome 16 open reading frame 45
hsa-miR-221-3p	NDUFA1	NADH:ubiquinone oxidoreductase subunit A1
hsa-miR-221-3p	ENTPD7	ectonucleoside triphosphate diphosphohydrolase 7
hsa-miR-221-3p	PTPRM	protein tyrosine phosphatase, receptor type M
hsa-miR-221-3p	DEPDC4	DEP domain containing 4
hsa-miR-221-3p	LRRN1	leucine rich repeat neuronal 1
hsa-miR-221-3p	KCNK2	potassium two pore domain channel subfamily K member 2
hsa-miR-221-3p	CTCF	CCCTC-binding factor
hsa-miR-221-3p	KLF3	Kruppel like factor 3
hsa-miR-221-3p	HTT	huntingtin
hsa-miR-221-3p	HEXIM1	HEXIM P-TEFb complex subunit 1
hsa-miR-221-3p	SATB1	SATB homeobox 1
hsa-miR-221-3p	OLA1	Obg like ATPase 1
hsa-miR-221-3p	SLC25A46	solute carrier family 25 member 46
hsa-miR-221-3p	OSBPL3	oxysterol binding protein like 3
hsa-miR-221-3p	IPO5	importin 5
hsa-miR-221-3p	FKTN	fukutin
hsa-miR-221-3p	SOX11	SRY-box 11
hsa-miR-221-3p	SLC40A1	solute carrier family 40 member 1
hsa-miR-221-3p	SPATS2L	spermatogenesis associated serine rich 2 like
hsa-miR-221-3p	MAPK8	mitogen-activated protein kinase 8
hsa-miR-221-3p	GALC	galactosylceramidase
hsa-miR-221-3p	FIGNL2	fidgetin like 2
hsa-miR-221-3p	RREB1	ras responsive element binding protein 1
hsa-miR-221-3p	MCMDC2	minichromosome maintenance domain containing 2
hsa-miR-221-3p	BBS4	Bardet-Biedl syndrome 4
hsa-miR-221-3p	PGGT1B	protein geranylgeranyltransferase type I subunit beta
hsa-miR-221-3p	FOS	Fos proto-oncogene, AP-1 transcription factor subunit
hsa-miR-221-3p	TDRKH	tudor and KH domain containing
hsa-miR-221-3p	PRKAA2	protein kinase AMP-activated catalytic subunit alpha 2
hsa-miR-221-3p	SLITRK6	SLIT and NTRK like family member 6
hsa-miR-221-3p	RBM18	RNA binding motif protein 18
hsa-miR-221-3p	TAOK1	TAO kinase 1
hsa-miR-221-3p	CXCL11	C-X-C motif chemokine ligand 11

hsa-miR-221-3p	ETS2	ETS proto-oncogene 2, transcription factor
hsa-miR-221-3p	COX15	cytochrome c oxidase assembly homolog COX15
hsa-miR-221-3p	ATP2B3	ATPase plasma membrane Ca2+ transporting 3
hsa-miR-221-3p	ARID1A	AT-rich interaction domain 1A
hsa-miR-221-3p	RAB14	RAB14, member RAS oncogene family
hsa-miR-221-3p	NAV3	neuron navigator 3
hsa-miR-221-3p	ZNF595	zinc finger protein 595
hsa-miR-221-3p	ASB7	ankyrin repeat and SOCS box containing 7
hsa-miR-221-3p	ADHFE1	alcohol dehydrogenase, iron containing 1
hsa-miR-221-3p	KBTBD8	kelch repeat and BTB domain containing 8
hsa-miR-221-3p	PDZRN4	PDZ domain containing ring finger 4
hsa-miR-221-3p	STMN1	stathmin 1
hsa-miR-221-3p	PAFAH1B2	platelet activating factor acetylhydrolase 1b catalytic subunit 2
hsa-miR-221-3p	CASP3	caspase 3
hsa-miR-221-3p	MAT2A	methionine adenosyltransferase 2A
hsa-miR-221-3p	BCHE	butyrylcholinesterase
hsa-miR-221-3p	DDX42	DEAD-box helicase 42
hsa-miR-221-3p	GTF2B	general transcription factor IIB
hsa-miR-221-3p	PITPNM2	phosphatidylinositol transfer protein membrane associated 2
hsa-miR-221-3p	KLHL18	kelch like family member 18
hsa-miR-221-3p	RNF44	ring finger protein 44
hsa-miR-221-3p	SLC45A3	solute carrier family 45 member 3
hsa-miR-221-3p	SLC6A9	solute carrier family 6 member 9
hsa-miR-221-3p	LIFR	LIF receptor alpha
hsa-miR-221-3p	ARHGAP42	Rho GTPase activating protein 42
hsa-miR-221-3p	VEZF1	vascular endothelial zinc finger 1
hsa-miR-221-3p	RAB3GAP2	RAB3 GTPase activating non-catalytic protein subunit 2
hsa-miR-221-3p	MTMR6	myotubularin related protein 6
hsa-miR-221-3p	MED1	mediator complex subunit 1
hsa-miR-221-3p	COL6A5	collagen type VI alpha 5 chain
hsa-miR-221-3p	USP49	ubiquitin specific peptidase 49
hsa-miR-221-3p	RGS8	regulator of G protein signaling 8
hsa-miR-221-3p	CD8A	CD8a molecule
hsa-miR-221-3p	MPRIP	myosin phosphatase Rho interacting protein
hsa-miR-221-3p	KIAA1549L	KIAA1549 like
hsa-miR-221-3p	C12orf77	chromosome 12 open reading frame 77
hsa-miR-221-3p	PRAMEF13	PRAME family member 13
hsa-miR-221-3p	NIPA1	NIPA magnesium transporter 1
hsa-miR-221-3p	RANBP2	RAN binding protein 2
hsa-miR-221-3p	PRAMEF14	PRAME family member 14
hsa-miR-221-3p	KCNH1	potassium voltage-gated channel subfamily H member 1
hsa-miR-221-3p	SLC16A6	solute carrier family 16 member 6
hsa-miR-221-3p	PCDH11X	protocadherin 11 X-linked

hsa-miR-221-3p	CHORDC1	cysteine and histidine rich domain containing 1
hsa-miR-221-3p	PCDH11Y	protocadherin 11 Y-linked
hsa-miR-221-3p	BCL11B	BCL11B, BAF complex component
hsa-miR-221-3p	C4orf33	chromosome 4 open reading frame 33
hsa-miR-221-3p	PRSS37	serine protease 37
hsa-miR-221-3p	KCNA1	potassium voltage-gated channel subfamily A member 1
hsa-miR-221-3p	LRP10	LDL receptor related protein 10
hsa-miR-221-3p	DYRK1A	dual specificity tyrosine phosphorylation regulated kinase 1A
hsa-miR-221-3p	SLAIN2	SLAIN motif family member 2
hsa-miR-221-3p	DDX58	DExD/H-box helicase 58
hsa-miR-221-3p	TAF9B	TATA-box binding protein associated factor 9b
hsa-miR-221-3p	CYP1B1	cytochrome P450 family 1 subfamily B member 1
hsa-miR-221-3p	UBN2	ubinuclein 2
hsa-miR-221-3p	GPATCH2L	G-patch domain containing 2 like
hsa-miR-221-3p	SNCA	synuclein alpha
hsa-miR-221-3p	WDR77	WD repeat domain 77
hsa-miR-221-3p	IL1RAP	interleukin 1 receptor accessory protein
hsa-miR-221-3p	CHFR	checkpoint with forkhead and ring finger domains
hsa-miR-221-3p	C16orf82	chromosome 16 open reading frame 82
hsa-miR-221-3p	ZBTB20	zinc finger and BTB domain containing 20
hsa-miR-221-3p	ESYT1	extended synaptotagmin 1
hsa-miR-221-3p	KRT81	keratin 81
hsa-miR-221-3p	DICER1	dicer 1, ribonuclease III
hsa-miR-221-3p	MDM2	MDM2 proto-oncogene
hsa-miR-221-3p	ZBTB41	zinc finger and BTB domain containing 41
hsa-miR-221-3p	NCAM1	neural cell adhesion molecule 1
hsa-miR-221-3p	MAP4K4	mitogen-activated protein kinase kinase kinase kinase 4
hsa-miR-221-3p	MYOD1	myogenic differentiation 1
hsa-miR-221-3p	GNB3	G protein subunit beta 3
hsa-miR-221-3p	MMD2	monocyte to macrophage differentiation associated 2
hsa-miR-221-3p	SLC25A21	solute carrier family 25 member 21
hsa-miR-221-3p	ITPRID1	ITPR interacting domain containing 1
hsa-miR-221-3p	CAVIN4	caveolae associated protein 4
hsa-miR-221-3p	AGO4	argonaute RISC catalytic component 4
hsa-miR-221-3p	AJAP1	adherens junctions associated protein 1
hsa-miR-221-3p	ITIH5	inter-alpha-trypsin inhibitor heavy chain family member 5
hsa-miR-221-3p	CNKSR3	CNKSR family member 3
hsa-miR-221-3p	CT45A5	cancer/testis antigen family 45 member A5
hsa-miR-221-3p	PKDCC	protein kinase domain containing, cytoplasmic
hsa-miR-221-3p	STAT2	signal transducer and activator of transcription 2
hsa-miR-221-3p	A4GNT	alpha-1,4-N-acetylglucosaminyltransferase
hsa-miR-221-3p	CT45A10	cancer/testis antigen family 45 member A10
hsa-miR-221-3p	GCNT1	glucosaminyl (N-acetyl) transferase 1

hsa-miR-221-3p	ERI2	ERI1 exoribonuclease family member 2
hsa-miR-221-3p	APAF1	apoptotic peptidase activating factor 1
hsa-miR-221-3p	CLDN12	claudin 12
hsa-miR-221-3p	SAR1A	secretion associated Ras related GTPase 1A
hsa-miR-424-5p	PAPPA	pappalysin 1
hsa-miR-424-5p	FASN	fatty acid synthase
hsa-miR-424-5p	UNC80	unc-80 homolog, NALCN channel complex subunit
hsa-miR-424-5p	FGF2	fibroblast growth factor 2
hsa-miR-424-5p	TNRC6B	trinucleotide repeat containing 6B
hsa-miR-424-5p	PTPN4	protein tyrosine phosphatase, non-receptor type 4
hsa-miR-424-5p	PHF19	PHD finger protein 19
hsa-miR-424-5p	UBE2Q1	ubiquitin conjugating enzyme E2 Q1
hsa-miR-424-5p	LSM11	LSM11, U7 small nuclear RNA associated
hsa-miR-424-5p	ANKUB1	ankyrin repeat and ubiquitin domain containing 1
hsa-miR-424-5p	CCNE1	cyclin E1
hsa-miR-424-5p	ATG14	autophagy related 14
hsa-miR-424-5p	SLC13A3	solute carrier family 13 member 3
hsa-miR-424-5p	ARIH1	ariadne RBR E3 ubiquitin protein ligase 1
hsa-miR-424-5p	BTRC	beta-transducin repeat containing E3 ubiquitin protein ligase
hsa-miR-424-5p	SPRYD3	SPRY domain containing 3
hsa-miR-424-5p	ARL2	ADP ribosylation factor like GTPase 2
hsa-miR-424-5p	CASK	calcium/calmodulin dependent serine protein kinase
hsa-miR-424-5p	NUP50	nucleoporin 50
hsa-miR-424-5p	DCLK1	doublecortin like kinase 1
hsa-miR-424-5p	DESI1	desumoylating isopeptidase 1
hsa-miR-424-5p	ZBTB46	zinc finger and BTB domain containing 46
hsa-miR-424-5p	FGF7	fibroblast growth factor 7
hsa-miR-424-5p	RECK	reversion inducing cysteine rich protein with kazal motifs
hsa-miR-424-5p	AXIN2	axin 2
hsa-miR-424-5p	GPR63	G protein-coupled receptor 63
hsa-miR-424-5p	SYNJ1	synaptojanin 1
hsa-miR-424-5p	NECTIN1	nectin cell adhesion molecule 1
hsa-miR-424-5p	GAREM1	GRB2 associated regulator of MAPK1 subtype 1
hsa-miR-424-5p	ABL2	ABL proto-oncogene 2, non-receptor tyrosine kinase
hsa-miR-424-5p	FBXO21	F-box protein 21
hsa-miR-424-5p	C2orf42	chromosome 2 open reading frame 42
hsa-miR-424-5p	KIF1B	kinesin family member 1B
hsa-miR-424-5p	LUZP1	leucine zipper protein 1
hsa-miR-424-5p	TBL1XR1	transducin beta like 1 X-linked receptor 1
hsa-miR-424-5p	SLC11A2	solute carrier family 11 member 2
hsa-miR-424-5p	MOB3B	MOB kinase activator 3B
hsa-miR-424-5p	ZBTB44	zinc finger and BTB domain containing 44
hsa-miR-424-5p	DMPK	DM1 protein kinase

hsa-miR-424-5p	MGAT4A	alpha-1,3-mannosyl-glycoprotein 4-beta-N-acetylglucosaminyltransferase A
hsa-miR-424-5p	EPHB2	EPH receptor B2
hsa-miR-424-5p	ANO3	anoctamin 3
hsa-miR-424-5p	SLC9A6	solute carrier family 9 member A6
hsa-miR-424-5p	APLN	apelin
hsa-miR-424-5p	IPO7	importin 7
hsa-miR-424-5p	RASGEF1B	RasGEF domain family member 1B
hsa-miR-424-5p	ATG9A	autophagy related 9A
hsa-miR-424-5p	CPEB2	cytoplasmic polyadenylation element binding protein 2
hsa-miR-424-5p	AHCYL2	adenosylhomocysteinase like 2
hsa-miR-424-5p	UBE4B	ubiquitination factor E4B
hsa-miR-424-5p	CYB561A3	cytochrome b561 family member A3
hsa-miR-424-5p	ARL3	ADP ribosylation factor like GTPase 3
hsa-miR-424-5p	PLAG1	PLAG1 zinc finger
hsa-miR-424-5p	CDCA4	cell division cycle associated 4
hsa-miR-424-5p	CACNA1E	calcium voltage-gated channel subunit alpha1 E
hsa-miR-424-5p	PISD	phosphatidylserine decarboxylase
hsa-miR-424-5p	KCNJ2	potassium voltage-gated channel subfamily J member 2
hsa-miR-424-5p	UBE2V1	ubiquitin conjugating enzyme E2 V1
hsa-miR-424-5p	SPRED1	sprouty related EVH1 domain containing 1
hsa-miR-424-5p	PPM1E	protein phosphatase, Mg2+/Mn2+ dependent 1E
hsa-miR-424-5p	MTMR3	myotubularin related protein 3
hsa-miR-424-5p	SHOC2	SHOC2, leucine rich repeat scaffold protein
hsa-miR-424-5p	TLK1	tousled like kinase 1
hsa-miR-424-5p	LURAP1L	leucine rich adaptor protein 1 like
hsa-miR-424-5p	PTPN3	protein tyrosine phosphatase, non-receptor type 3
hsa-miR-424-5p	TMEM100	transmembrane protein 100
hsa-miR-424-5p	RASSF8	Ras association domain family member 8
hsa-miR-424-5p	FBXW7	F-box and WD repeat domain containing 7
hsa-miR-424-5p	AKT3	AKT serine/threonine kinase 3
hsa-miR-424-5p	MYB	MYB proto-oncogene, transcription factor
hsa-miR-424-5p	GRM7	glutamate metabotropic receptor 7
hsa-miR-424-5p	CCND2	cyclin D2
hsa-miR-424-5p	WEE1	WEE1 G2 checkpoint kinase
hsa-miR-424-5p	STOX2	storkhead box 2
hsa-miR-424-5p	UBN2	ubinuclein 2
hsa-miR-424-5p	CYP26B1	cytochrome P450 family 26 subfamily B member 1
hsa-miR-424-5p	ZCCHC3	zinc finger CCHC-type containing 3
hsa-miR-424-5p	RNF144B	ring finger protein 144B
hsa-miR-424-5p	SALL4	spalt like transcription factor 4
hsa-miR-424-5p	TBPL1	TATA-box binding protein like 1
hsa-miR-424-5p	KDSR	3-ketodihydrosphingosine reductase
hsa-miR-424-5p	SCN8A	sodium voltage-gated channel alpha subunit 8
hsa-miR-424-5p	SEMA6D	semaphorin 6D

hsa-miR-424-5p	SREK1	splicing regulatory glutamic acid and lysine rich protein 1
hsa-miR-424-5p	HTR2A	5-hydroxytryptamine receptor 2A
hsa-miR-424-5p	RAB11FIP2	RAB11 family interacting protein 2
hsa-miR-424-5p	DNAJB4	DnaJ heat shock protein family (Hsp40) member B4
hsa-miR-424-5p	KIF5C	kinesin family member 5C
hsa-miR-424-5p	GPATCH8	G-patch domain containing 8
hsa-miR-424-5p	N4BP1	NEDD4 binding protein 1
hsa-miR-424-5p	ZNF691	zinc finger protein 691
hsa-miR-424-5p	MAMSTR	MEF2 activating motif and SAP domain containing transcriptional regulator
hsa-miR-424-5p	ZMAT3	zinc finger matrin-type 3
hsa-miR-424-5p	UBFD1	ubiquitin family domain containing 1
hsa-miR-424-5p	ARMH4	armadillo-like helical domain containing 4
hsa-miR-424-5p	HIPK2	homeodomain interacting protein kinase 2
hsa-miR-424-5p	CEP55	centrosomal protein 55
hsa-miR-424-5p	MKX	mohawk homeobox
hsa-miR-424-5p	KIF23	kinesin family member 23
hsa-miR-424-5p	CFAP45	cilia and flagella associated protein 45
hsa-miR-424-5p	CNOT6L	CCR4-NOT transcription complex subunit 6 like
hsa-miR-424-5p	RFX3	regulatory factor X3
hsa-miR-424-5p	TFAP2A	transcription factor AP-2 alpha
hsa-miR-424-5p	PCMT1	protein-L-isoaspartate (D-aspartate) O-methyltransferase
hsa-miR-424-5p	EDA	ectodysplasin A
hsa-miR-424-5p	SEC24A	SEC24 homolog A, COPII coat complex component
hsa-miR-424-5p	PAFAH1B1	platelet activating factor acetylhydrolase 1b regulatory subunit 1
hsa-miR-424-5p	SLC25A37	solute carrier family 25 member 37
hsa-miR-424-5p	DDX3X	DEAD-box helicase 3 X-linked
hsa-miR-424-5p	MYLK	myosin light chain kinase
hsa-miR-424-5p	SETD3	SET domain containing 3, actin histidine methyltransferase
hsa-miR-424-5p	CCDC6	coiled-coil domain containing 6
hsa-miR-424-5p	ARHGDIA	Rho GDP dissociation inhibitor alpha
hsa-miR-424-5p	NAPG	NSF attachment protein gamma
hsa-miR-424-5p	GABARAPL1	GABA type A receptor associated protein like 1
hsa-miR-424-5p	FGFR1	fibroblast growth factor receptor 1
hsa-miR-424-5p	JPH3	junctophilin 3
hsa-miR-424-5p	USP42	ubiquitin specific peptidase 42
hsa-miR-424-5p	STRADB	STE20 related adaptor beta
hsa-miR-424-5p	LRRN3	leucine rich repeat neuronal 3
hsa-miR-424-5p	STXBP5	syntaxin binding protein 5
hsa-miR-424-5p	ZFHX4	zinc finger homeobox 4
hsa-miR-424-5p	ZNF622	zinc finger protein 622
hsa-miR-424-5p	OOEP	oocyte expressed protein
hsa-miR-424-5p	CBX2	chromobox 2

hsa-miR-424-5p	SRPRA	SRP receptor subunit alpha
hsa-miR-424-5p	KLHL2	kelch like family member 2
hsa-miR-424-5p	VEGFA	vascular endothelial growth factor A
hsa-miR-424-5p	ZBTB34	zinc finger and BTB domain containing 34
hsa-miR-424-5p	KIF5B	kinesin family member 5B
hsa-miR-424-5p	MYO5A	myosin VA
hsa-miR-424-5p	CASR	calcium sensing receptor
hsa-miR-424-5p	LRIG2	leucine rich repeats and immunoglobulin like domains 2
hsa-miR-424-5p	SMURF1	SMAD specific E3 ubiquitin protein ligase 1
hsa-miR-424-5p	ZNF367	zinc finger protein 367
hsa-miR-424-5p	USP25	ubiquitin specific peptidase 25
hsa-miR-424-5p	ACTR2	ARP2 actin related protein 2 homolog
hsa-miR-424-5p	PLPP1	phospholipid phosphatase 1
hsa-miR-424-5p	RNF217	ring finger protein 217
hsa-miR-424-5p	BTAF1	B-TFIID TATA-box binding protein associated factor 1
hsa-miR-424-5p	TRANK1	tetratricopeptide repeat and ankyrin repeat containing 1
hsa-miR-424-5p	CHAC1	ChaC glutathione specific gamma-glutamylcyclotransferase 1
hsa-miR-424-5p	PTPRR	protein tyrosine phosphatase, receptor type R
hsa-miR-424-5p	TNFSF13B	TNF superfamily member 13b
hsa-miR-424-5p	IPPK	inositol-pentakisphosphate 2-kinase
hsa-miR-424-5p	ZBTB39	zinc finger and BTB domain containing 39
hsa-miR-424-5p	WNK3	WNK lysine deficient protein kinase 3
hsa-miR-424-5p	SUCO	SUN domain containing ossification factor
hsa-miR-424-5p	LATS1	large tumor suppressor kinase 1
hsa-miR-424-5p	MAP2K1	mitogen-activated protein kinase kinase 1
hsa-miR-424-5p	AMOTL1	angiomotin like 1
hsa-miR-424-5p	RBPJ	recombination signal binding protein for immunoglobulin kappa J region
hsa-miR-424-5p	TMEM183A	transmembrane protein 183A
hsa-miR-424-5p	STXBP3	syntaxin binding protein 3
hsa-miR-424-5p	DCP1A	decapping mRNA 1A
hsa-miR-424-5p	MYBL1	MYB proto-oncogene like 1
hsa-miR-424-5p	KIF21A	kinesin family member 21A
hsa-miR-424-5p	GHR	growth hormone receptor
hsa-miR-424-5p	SUMO3	small ubiquitin-like modifier 3
hsa-miR-424-5p	AVL9	AVL9 cell migration associated
hsa-miR-424-5p	PIAS2	protein inhibitor of activated STAT 2
hsa-miR-424-5p	FBXL20	F-box and leucine rich repeat protein 20
hsa-miR-424-5p	UBE4A	ubiquitination factor E4A
hsa-miR-424-5p	SLIT2	slit guidance ligand 2
hsa-miR-424-5p	XPO7	exportin 7
hsa-miR-424-5p	MFN2	mitofusin 2
hsa-miR-424-5p	CHD2	chromodomain helicase DNA binding protein 2
hsa-miR-424-5p	POU2F1	POU class 2 homeobox 1

hsa-miR-424-5p	HSPA4L	heat shock protein family A (Hsp70) member 4 like
hsa-miR-424-5p	COP1	COP1, E3 ubiquitin ligase
hsa-miR-424-5p	ADAMTS3	ADAM metallopeptidase with thrombospondin type 1 motif 3
hsa-miR-424-5p	P3H2	prolyl 3-hydroxylase 2
hsa-miR-424-5p	RAD23B	RAD23 homolog B, nucleotide excision repair protein
hsa-miR-424-5p	FOXK1	forkhead box K1
hsa-miR-424-5p	RNF10	ring finger protein 10
hsa-miR-424-5p	PLXNA4	plexin A4
hsa-miR-424-5p	PPM1A	protein phosphatase, Mg2+/Mn2+ dependent 1A
hsa-miR-424-5p	LITAF	lipopolysaccharide induced TNF factor
hsa-miR-424-5p	RETREG2	reticulophagy regulator family member 2
hsa-miR-424-5p	KANK1	KN motif and ankyrin repeat domains 1
hsa-miR-424-5p	PPP2R1B	protein phosphatase 2 scaffold subunit Abeta
hsa-miR-424-5p	SYDE2	synapse defective Rho GTPase homolog 2
hsa-miR-424-5p	RPS6KA3	ribosomal protein S6 kinase A3
hsa-miR-424-5p	CHEK1	checkpoint kinase 1
hsa-miR-424-5p	OMG	oligodendrocyte myelin glycoprotein
hsa-miR-424-5p	PCDH17	protocadherin 17
hsa-miR-424-5p	PLXNC1	plexin C1
hsa-miR-424-5p	GALNT13	polypeptide N-acetylgalactosaminyltransferase 13
hsa-miR-424-5p	CBX4	chromobox 4
hsa-miR-424-5p	ACVR2A	activin A receptor type 2A
hsa-miR-424-5p	ATXN2	ataxin 2
hsa-miR-424-5p	SIK1	salt inducible kinase 1
hsa-miR-424-5p	SMAD7	SMAD family member 7
hsa-miR-424-5p	SALL1	spalt like transcription factor 1
hsa-miR-424-5p	YTHDC1	YTH domain containing 1
hsa-miR-424-5p	ZNRF3	zinc and ring finger 3
hsa-miR-424-5p	CLOCK	clock circadian regulator
hsa-miR-424-5p	KRTAP4-6	keratin associated protein 4-6
hsa-miR-424-5p	ADGRL1	adhesion G protein-coupled receptor L1
hsa-miR-424-5p	AGO4	argonaute RISC catalytic component 4
hsa-miR-424-5p	ASH1L	ASH1 like histone lysine methyltransferase
hsa-miR-424-5p	ZFHX3	zinc finger homeobox 3
hsa-miR-424-5p	USP31	ubiquitin specific peptidase 31
hsa-miR-424-5p	HSPG2	heparan sulfate proteoglycan 2
hsa-miR-424-5p	CD2AP	CD2 associated protein
hsa-miR-424-5p	CCNT1	cyclin T1
hsa-miR-424-5p	PPP1R11	protein phosphatase 1 regulatory inhibitor subunit 11
hsa-miR-424-5p	COL12A1	collagen type XII alpha 1 chain
hsa-miR-424-5p	GATAD2A	GATA zinc finger domain containing 2A
hsa-miR-424-5p	RARB	retinoic acid receptor beta
hsa-miR-424-5p	LAMP3	lysosomal associated membrane protein 3
hsa-miR-424-5p	TMEM245	transmembrane protein 245

hsa-miR-424-5p	MEOX2	mesenchyme homeobox 2
hsa-miR-424-5p	DIXDC1	DIX domain containing 1
hsa-miR-424-5p	TGFBR3	transforming growth factor beta receptor 3
hsa-miR-424-5p	CCND1	cyclin D1
hsa-miR-424-5p	FAM133B	family with sequence similarity 133 member B
hsa-miR-424-5p	ELMSAN1	ELM2 and Myb/SANT domain containing 1
hsa-miR-424-5p	STK33	serine/threonine kinase 33
hsa-miR-424-5p	AK4	adenylate kinase 4
hsa-miR-424-5p	C1orf21	chromosome 1 open reading frame 21
hsa-miR-424-5p	SOCS6	suppressor of cytokine signaling 6
hsa-miR-424-5p	SLC12A2	solute carrier family 12 member 2
hsa-miR-424-5p	INSR	insulin receptor
hsa-miR-424-5p	TMCC1	transmembrane and coiled-coil domain family 1
hsa-miR-424-5p	SEL1L3	SEL1L family member 3
hsa-miR-424-5p	UNC5D	unc-5 netrin receptor D
hsa-miR-424-5p	JARID2	jumonji and AT-rich interaction domain containing 2
hsa-miR-424-5p	SIRT4	sirtuin 4
hsa-miR-424-5p	TMEM178B	transmembrane protein 178B
hsa-miR-424-5p	SEMA3A	semaphorin 3A
hsa-miR-424-5p	LRIG1	leucine rich repeats and immunoglobulin like domains 1
hsa-miR-424-5p	TMC7	transmembrane channel like 7
hsa-miR-424-5p	RPS6KA6	ribosomal protein S6 kinase A6
hsa-miR-424-5p	EZH1	enhancer of zeste 1 polycomb repressive complex 2 subunit
hsa-miR-424-5p	PTH	parathyroid hormone
hsa-miR-424-5p	PAFAH1B2	platelet activating factor acetylhydrolase 1b catalytic subunit 2
hsa-miR-424-5p	SPTLC1	serine palmitoyltransferase long chain base subunit 1
hsa-miR-424-5p	KRTAP11-1	keratin associated protein 11-1
hsa-miR-424-5p	DENND1B	DENN domain containing 1B
hsa-miR-424-5p	ZNF449	zinc finger protein 449
hsa-miR-424-5p	AMOT	angiomotin
hsa-miR-424-5p	DLL1	delta like canonical Notch ligand 1
hsa-miR-424-5p	SLC20A2	solute carrier family 20 member 2
hsa-miR-424-5p	FERMT2	fermitin family member 2
hsa-miR-424-5p	ILDR2	immunoglobulin like domain containing receptor 2
hsa-miR-424-5p	CSRNP1	cysteine and serine rich nuclear protein 1
hsa-miR-424-5p	NAA25	N(alpha)-acetyltransferase 25, NatB auxiliary subunit
hsa-miR-424-5p	ELL	elongation factor for RNA polymerase II
hsa-miR-424-5p	ZC3H13	zinc finger CCCH-type containing 13
hsa-miR-424-5p	CSDE1	cold shock domain containing E1
hsa-miR-424-5p	CPEB3	cytoplasmic polyadenylation element binding protein 3
hsa-miR-424-5p	MEX3C	mex-3 RNA binding family member C
hsa-miR-424-5p	BAG4	BCL2 associated athanogene 4
hsa-miR-424-5p	VPS33B	VPS33B, late endosome and lysosome associated

hsa-miR-424-5p	CACUL1	CDK2 associated cullin domain 1
hsa-miR-424-5p	TBP	TATA-box binding protein
hsa-miR-424-5p	ARFGAP2	ADP ribosylation factor GTPase activating protein 2
hsa-miR-424-5p	TRIM66	tripartite motif containing 66
hsa-miR-424-5p	E2F3	E2F transcription factor 3
hsa-miR-424-5p	WNT3A	Wnt family member 3A
hsa-miR-424-5p	ABHD2	abhydrolase domain containing 2
hsa-miR-424-5p	ZNRF2	zinc and ring finger 2
hsa-miR-424-5p	ATXN1L	ataxin 1 like
hsa-miR-424-5p	CC2D1B	coiled-coil and C2 domain containing 1B
hsa-miR-424-5p	KCNK10	potassium two pore domain channel subfamily K member 10
hsa-miR-424-5p	SIK1B	salt inducible kinase 1B (putative)
hsa-miR-424-5p	SYNRG	synergin gamma
hsa-miR-424-5p	CBX6	chromobox 6
hsa-miR-424-5p	CD47	CD47 molecule
hsa-miR-424-5p	TAB3	TGF-beta activated kinase 1 (MAP3K7) binding protein 3
hsa-miR-424-5p	NOS1	nitric oxide synthase 1
hsa-miR-424-5p	IFT74	intraflagellar transport 74
hsa-miR-424-5p	SERBP1	SERPINE1 mRNA binding protein 1
hsa-miR-424-5p	ETNK1	ethanolamine kinase 1
hsa-miR-424-5p	G2E3	G2/M-phase specific E3 ubiquitin protein ligase
hsa-miR-424-5p	ATXN7L2	ataxin 7 like 2
hsa-miR-424-5p	AMER1	APC membrane recruitment protein 1
hsa-miR-424-5p	TRABD2B	TraB domain containing 2B
hsa-miR-424-5p	SEMA5B	semaphorin 5B
hsa-miR-424-5p	NHLRC2	NHL repeat containing 2
hsa-miR-424-5p	NR2C2	nuclear receptor subfamily 2 group C member 2
hsa-miR-424-5p	SMIM13	small integral membrane protein 13
hsa-miR-424-5p	CDC25A	cell division cycle 25A
hsa-miR-424-5p	DDX3Y	DEAD-box helicase 3 Y-linked
hsa-miR-424-5p	TCAIM	T cell activation inhibitor, mitochondrial
hsa-miR-424-5p	WIPI2	WD repeat domain, phosphoinositide interacting 2
hsa-miR-424-5p	TMEM268	transmembrane protein 268
hsa-miR-424-5p	ZMYM2	zinc finger MYM-type containing 2
hsa-miR-424-5p	RASEF	RAS and EF-hand domain containing
hsa-miR-424-5p	ATXN7L1	ataxin 7 like 1
hsa-miR-424-5p	PDZD8	PDZ domain containing 8
hsa-miR-424-5p	EGLN1	egl-9 family hypoxia inducible factor 1
hsa-miR-424-5p	sept-02	septin 2
hsa-miR-424-5p	MAP3K13	mitogen-activated protein kinase kinase kinase 13
hsa-miR-424-5p	RBM6	RNA binding motif protein 6
hsa-miR-424-5p	CDC37L1	cell division cycle 37 like 1
hsa-miR-424-5p	MED26	mediator complex subunit 26

hsa-miR-424-5p	HERC6	HECT and RLD domain containing E3 ubiquitin protein ligase family member 6
hsa-miR-424-5p	DRD1	dopamine receptor D1
hsa-miR-424-5p	CXCR5	C-X-C motif chemokine receptor 5
hsa-miR-424-5p	PDIA6	protein disulfide isomerase family A member 6
hsa-miR-424-5p	RBBP6	RB binding protein 6, ubiquitin ligase
hsa-miR-424-5p	GSTCD	glutathione S-transferase C-terminal domain containing
hsa-miR-424-5p	USP15	ubiquitin specific peptidase 15
hsa-miR-424-5p	SYT3	synaptotagmin 3
hsa-miR-424-5p	PAG1	phosphoprotein membrane anchor with glycosphingolipid microdomains 1
hsa-miR-424-5p	MKNK1	MAP kinase interacting serine/threonine kinase 1
hsa-miR-424-5p	TLL1	tolloid like 1
hsa-miR-424-5p	WNT7A	Wnt family member 7A
hsa-miR-424-5p	QKI	QKI, KH domain containing RNA binding
hsa-miR-424-5p	RAB9B	RAB9B, member RAS oncogene family
hsa-miR-424-5p	BCL11B	BCL11B, BAF complex component
hsa-miR-424-5p	TMEM189-UBE2V1	TMEM189-UBE2V1 readthrough
hsa-miR-424-5p	LRP6	LDL receptor related protein 6
hsa-miR-424-5p	RS1	retinoschisin 1
hsa-miR-424-5p	SON	SON DNA binding protein
hsa-miR-424-5p	BZW1	basic leucine zipper and W2 domains 1
hsa-miR-424-5p	PIP4P2	phosphatidylinositol-4,5-bisphosphate 4-phosphatase 2
hsa-miR-424-5p	KCNG4	potassium voltage-gated channel modifier subfamily G member 4
hsa-miR-424-5p	TFCP2L1	transcription factor CP2 like 1
hsa-miR-424-5p	DYRK1B	dual specificity tyrosine phosphorylation regulated kinase 1B
hsa-miR-424-5p	CLUH	clustered mitochondria homolog
hsa-miR-424-5p	CACNA2D1	calcium voltage-gated channel auxiliary subunit alpha2delta 1
hsa-miR-424-5p	RREB1	ras responsive element binding protein 1
hsa-miR-424-5p	SLC36A1	solute carrier family 36 member 1
hsa-miR-424-5p	ZSCAN31	zinc finger and SCAN domain containing 31
hsa-miR-424-5p	TENM2	teneurin transmembrane protein 2
hsa-miR-424-5p	PDE3B	phosphodiesterase 3B
hsa-miR-424-5p	ATXN7L3	ataxin 7 like 3
hsa-miR-424-5p	UBQLNL	ubiquilin like
hsa-miR-424-5p	CHUK	conserved helix-loop-helix ubiquitous kinase
hsa-miR-424-5p	WWC1	WW and C2 domain containing 1
hsa-miR-424-5p	CDC42SE2	CDC42 small effector 2
hsa-miR-424-5p	CUX1	cut like homeobox 1
hsa-miR-424-5p	SNX16	sorting nexin 16
hsa-miR-424-5p	PARVA	parvin alpha
hsa-miR-424-5p	VPS4A	vacuolar protein sorting 4 homolog A

hsa-miR-424-5p	IVNS1ABP	influenza virus NS1A binding protein
hsa-miR-424-5p	SNRPB2	small nuclear ribonucleoprotein polypeptide B2
hsa-miR-424-5p	MASP1	mannan binding lectin serine peptidase 1
hsa-miR-424-5p	NAV1	neuron navigator 1
hsa-miR-424-5p	LARGE2	LARGE xylosyl- and glucuronyltransferase 2
hsa-miR-424-5p	RET	ret proto-oncogene
hsa-miR-424-5p	MTFR1L	mitochondrial fission regulator 1 like
hsa-miR-424-5p	C12orf76	chromosome 12 open reading frame 76
hsa-miR-424-5p	CLCN4	chloride voltage-gated channel 4
hsa-miR-424-5p	DEPDC4	DEP domain containing 4
hsa-miR-424-5p	CDK5R1	cyclin dependent kinase 5 regulatory subunit 1
hsa-miR-424-5p	TARBP2	TARBP2, RISC loading complex RNA binding subunit
hsa-miR-424-5p	IKBKB	inhibitor of nuclear factor kappa B kinase subunit beta
hsa-miR-424-5p	USP3	ubiquitin specific peptidase 3
hsa-miR-424-5p	GALNT7	polypeptide N-acetylgalactosaminyltransferase 7
hsa-miR-424-5p	CDK17	cyclin dependent kinase 17
hsa-miR-424-5p	ROCK2	Rho associated coiled-coil containing protein kinase 2
hsa-miR-424-5p	LGR5	leucine rich repeat containing G protein-coupled receptor 5
hsa-miR-424-5p	SCOC	short coiled-coil protein
hsa-miR-424-5p	ISLR	immunoglobulin superfamily containing leucine rich repeat
hsa-miR-424-5p	NRN1	neuritin 1
hsa-miR-424-5p	ARHGAP12	Rho GTPase activating protein 12
hsa-miR-424-5p	KIF3B	kinesin family member 3B
hsa-miR-424-5p	SYPL1	synaptophysin like 1
hsa-miR-424-5p	OGT	O-linked N-acetylglucosamine (GlcNAc) transferase
hsa-miR-424-5p	MNT	MAX network transcriptional repressor
hsa-miR-424-5p	YWHAH	tyrosine 3-monooxygenase/tryptophan 5-monooxygenase activation protein eta
hsa-miR-424-5p	LRRK1	leucine rich repeat kinase 1
hsa-miR-424-5p	IARS	isoleucyl-tRNA synthetase
hsa-miR-424-5p	TUBA4A	tubulin alpha 4a
hsa-miR-424-5p	ANKS1A	ankyrin repeat and sterile alpha motif domain containing 1A
hsa-miR-424-5p	DPY19L4	dpy-19 like 4
hsa-miR-424-5p	COPS7B	COP9 signalosome subunit 7B
hsa-miR-424-5p	ARHGAP32	Rho GTPase activating protein 32
hsa-miR-424-5p	FAM91A1	family with sequence similarity 91 member A1
hsa-miR-424-5p	SGK1	serum/glucocorticoid regulated kinase 1
hsa-miR-424-5p	NOB1	NIN1 (RPN12) binding protein 1 homolog
hsa-miR-424-5p	MOB4	MOB family member 4, phocein
hsa-miR-424-5p	MYO5B	myosin VB
hsa-miR-424-5p	PCDH9	protocadherin 9
hsa-miR-424-5p	NFATC3	nuclear factor of activated T cells 3
hsa-miR-424-5p	RAB9A	RAB9A, member RAS oncogene family

hsa-miR-424-5p	RBM12	RNA binding motif protein 12
hsa-miR-424-5p	CEP85L	centrosomal protein 85 like
hsa-miR-424-5p	ZNHIT6	zinc finger HIT-type containing 6
hsa-miR-424-5p	SYT4	synaptotagmin 4
hsa-miR-424-5p	PDK4	pyruvate dehydrogenase kinase 4
hsa-miR-424-5p	PRDM4	PR/SET domain 4
hsa-miR-424-5p	CPD	carboxypeptidase D
hsa-miR-424-5p	RUNDC3B	RUN domain containing 3B
hsa-miR-424-5p	SKI	SKI proto-oncogene
hsa-miR-424-5p	ADRB2	adrenoceptor beta 2
hsa-miR-424-5p	SSTR3	somatostatin receptor 3
hsa-miR-424-5p	SEH1L	SEH1 like nucleoporin
hsa-miR-424-5p	FAM81A	family with sequence similarity 81 member A
hsa-miR-424-5p	MAP3K9	mitogen-activated protein kinase kinase kinase 9
hsa-miR-424-5p	CAPRIN1	cell cycle associated protein 1
hsa-miR-424-5p	RBM24	RNA binding motif protein 24
hsa-miR-424-5p	BTG2	BTG anti-proliferation factor 2
hsa-miR-424-5p	PTPRD	protein tyrosine phosphatase, receptor type D
hsa-miR-424-5p	PRRC2C	proline rich coiled-coil 2C
hsa-miR-424-5p	EXOC3L2	exocyst complex component 3 like 2
hsa-miR-424-5p	CD80	CD80 molecule
hsa-miR-424-5p	NSG1	neuronal vesicle trafficking associated 1
hsa-miR-424-5p	FLT3	fms related tyrosine kinase 3
hsa-miR-424-5p	GNAT1	G protein subunit alpha transducin 1
hsa-miR-424-5p	SSR1	signal sequence receptor subunit 1
hsa-miR-424-5p	SAV1	salvador family WW domain containing protein 1
hsa-miR-424-5p	PEX13	peroxisomal biogenesis factor 13
hsa-miR-424-5p	UNC13A	unc-13 homolog A
hsa-miR-424-5p	SOBP	sine oculis binding protein homolog
hsa-miR-424-5p	HECTD1	HECT domain E3 ubiquitin protein ligase 1
hsa-miR-424-5p	GFAP	glial fibrillary acidic protein
hsa-miR-424-5p	CMPK1	cytidine/uridine monophosphate kinase 1
hsa-miR-424-5p	ZDHHC15	zinc finger DHHC-type containing 15
hsa-miR-424-5p	BCL2L2	BCL2 like 2
hsa-miR-424-5p	CCDC88C	coiled-coil domain containing 88C
hsa-miR-424-5p	PELI2	pellino E3 ubiquitin protein ligase family member 2
hsa-miR-424-5p	CHPT1	choline phosphotransferase 1
hsa-miR-424-5p	TMEM154	transmembrane protein 154
hsa-miR-424-5p	ARMCX2	armadillo repeat containing X-linked 2
hsa-miR-424-5p	HMBOX1	homeobox containing 1
hsa-miR-424-5p	EPHA7	EPH receptor A7
hsa-miR-424-5p	KCTD8	potassium channel tetramerization domain containing 8
hsa-miR-424-5p	PPT2	palmitoyl-protein thioesterase 2
hsa-miR-424-5p	CARM1	coactivator associated arginine methyltransferase 1
hsa-miR-424-5p	RFK	riboflavin kinase

hsa-miR-424-5p	ST8SIA3	ST8 alpha-N-acetyl-neuraminide alpha-2,8-sialyltransferase 3
hsa-miR-424-5p	AGO1	argonaute RISC catalytic component 1
hsa-miR-424-5p	RICTOR	RPTOR independent companion of MTOR complex 2
hsa-miR-424-5p	PNPLA6	patatin like phospholipase domain containing 6
hsa-miR-424-5p	ATXN7L3B	ataxin 7 like 3B
hsa-miR-424-5p	EPC1	enhancer of polycomb homolog 1
hsa-miR-424-5p	SIPA1L2	signal induced proliferation associated 1 like 2
hsa-miR-424-5p	CHIC1	cysteine rich hydrophobic domain 1
hsa-miR-424-5p	AMMECR1	Alport syndrome, mental retardation, midface hypoplasia and elliptocytosis chromosomal region gene 1
hsa-miR-424-5p	SLC39A10	solute carrier family 39 member 10
hsa-miR-424-5p	HEPHL1	hephaestin like 1
hsa-miR-424-5p	PIK3R1	phosphoinositide-3-kinase regulatory subunit 1
hsa-miR-424-5p	RUNX1T1	RUNX1 translocation partner 1
hsa-miR-424-5p	HIGD1A	HIG1 hypoxia inducible domain family member 1A
hsa-miR-424-5p	KCNN4	potassium calcium-activated channel subfamily N member 4
hsa-miR-424-5p	SESN1	sestrin 1
hsa-miR-424-5p	DENND4A	DENN domain containing 4A
hsa-miR-424-5p	DYNC1LI2	dynein cytoplasmic 1 light intermediate chain 2
hsa-miR-424-5p	WBP11	WW domain binding protein 11
hsa-miR-424-5p	DENND2C	DENN domain containing 2C
hsa-miR-424-5p	GLS2	glutaminase 2
hsa-miR-424-5p	SOX6	SRY-box 6
hsa-miR-424-5p	HMGA1	high mobility group AT-hook 1
hsa-miR-424-5p	NUFIP2	nuclear FMR1 interacting protein 2
hsa-miR-424-5p	LDLRAD2	low density lipoprotein receptor class A domain containing 2
hsa-miR-424-5p	FAM122B	family with sequence similarity 122B
hsa-miR-424-5p	ANKRD46	ankyrin repeat domain 46
hsa-miR-424-5p	ATF6	activating transcription factor 6
hsa-miR-424-5p	COBLL1	cordon-bleu WH2 repeat protein like 1
hsa-miR-424-5p	ELMOD1	ELMO domain containing 1
hsa-miR-424-5p	HTR4	5-hydroxytryptamine receptor 4
hsa-miR-424-5p	TSC22D2	TSC22 domain family member 2
hsa-miR-424-5p	UTP25	UTP25, small subunit processor component
hsa-miR-424-5p	USP44	ubiquitin specific peptidase 44
hsa-miR-424-5p	EYA1	EYA transcriptional coactivator and phosphatase 1
hsa-miR-424-5p	MYEF2	myelin expression factor 2
hsa-miR-424-5p	UBR3	ubiquitin protein ligase E3 component n-recognin 3
hsa-miR-424-5p	MCU	mitochondrial calcium uniporter
hsa-miR-424-5p	ATG13	autophagy related 13
hsa-miR-424-5p	PLRG1	pleiotropic regulator 1
hsa-miR-424-5p	FAM110C	family with sequence similarity 110 member C
hsa-miR-424-5p	IGF2R	insulin like growth factor 2 receptor

hsa-miR-424-5p	SLC4A4	solute carrier family 4 member 4
hsa-miR-424-5p	PIP4P1	phosphatidylinositol-4,5-bisphosphate 4-phosphatase 1
hsa-miR-424-5p	MAN2A2	mannosidase alpha class 2A member 2
hsa-miR-424-5p	CDK8	cyclin dependent kinase 8
hsa-miR-424-5p	CYP2S1	cytochrome P450 family 2 subfamily S member 1
hsa-miR-424-5p	ZCCHC2	zinc finger CCHC-type containing 2
hsa-miR-424-5p	OTX1	orthodenticle homeobox 1
hsa-miR-424-5p	NF1	neurofibromin 1
hsa-miR-424-5p	DMTF1	cyclin D binding myb like transcription factor 1
hsa-miR-424-5p	TMEM199	transmembrane protein 199
hsa-miR-424-5p	RELN	reelin
hsa-miR-424-5p	ZBTB20	zinc finger and BTB domain containing 20
hsa-miR-424-5p	FAM89A	family with sequence similarity 89 member A
hsa-miR-424-5p	NSMF	NMDA receptor synaptonuclear signaling and neuronal migration factor
hsa-miR-424-5p	ST7L	suppression of tumorigenicity 7 like
hsa-miR-424-5p	SPTBN2	spectrin beta, non-erythrocytic 2
hsa-miR-424-5p	TGIF2	TGFB induced factor homeobox 2
hsa-miR-424-5p	RAD50	RAD50 double strand break repair protein
hsa-miR-424-5p	CAPZA2	capping actin protein of muscle Z-line subunit alpha 2
hsa-miR-424-5p	CREBRF	CREB3 regulatory factor
hsa-miR-424-5p	CD3E	CD3e molecule
hsa-miR-424-5p	FAM135A	family with sequence similarity 135 member A
hsa-miR-424-5p	C1QL3	complement C1q like 3
hsa-miR-424-5p	TMEM135	transmembrane protein 135
hsa-miR-424-5p	NRP2	neuropilin 2
hsa-miR-424-5p	SLC2A14	solute carrier family 2 member 14
hsa-miR-424-5p	LRP2	LDL receptor related protein 2
hsa-miR-424-5p	ACOX1	acyl-CoA oxidase 1
hsa-miR-424-5p	SLC35G1	solute carrier family 35 member G1
hsa-miR-424-5p	GCC2	GRIP and coiled-coil domain containing 2
hsa-miR-424-5p	RSPO3	R-spondin 3
hsa-miR-424-5p	SH3GL2	SH3 domain containing GRB2 like 2, endophilin A1
hsa-miR-424-5p	TRAM1	translocation associated membrane protein 1
hsa-miR-424-5p	UROS	uroporphyrinogen III synthase
hsa-miR-424-5p	SNTB2	syntrophin beta 2
hsa-miR-424-5p	CMC4	C-X9-C motif containing 4
hsa-miR-424-5p	SLC6A11	solute carrier family 6 member 11
hsa-miR-424-5p	ENAH	ENAH, actin regulator
hsa-miR-424-5p	PPP6R3	protein phosphatase 6 regulatory subunit 3
hsa-miR-424-5p	HELZ	helicase with zinc finger
hsa-miR-424-5p	ELAC1	elaC ribonuclease Z 1
hsa-miR-424-5p	ARHGAP20	Rho GTPase activating protein 20
hsa-miR-424-5p	PLEKHA1	pleckstrin homology domain containing A1
hsa-miR-424-5p	ZC2HC1A	zinc finger C2HC-type containing 1A

hsa-miR-424-5p	SAMD10	sterile alpha motif domain containing 10
hsa-miR-424-5p	NCS1	neuronal calcium sensor 1
hsa-miR-424-5p	ACVR2B	activin A receptor type 2B
hsa-miR-424-5p	SVIP	small VCP interacting protein
hsa-miR-424-5p	GGA3	golgi associated, gamma adaptin ear containing, ARF binding protein 3
hsa-miR-424-5p	CPSF7	cleavage and polyadenylation specific factor 7
hsa-miR-424-5p	MYRIP	myosin VIIA and Rab interacting protein
hsa-miR-424-5p	NRBP1	nuclear receptor binding protein 1
hsa-miR-424-5p	SLC2A3	solute carrier family 2 member 3
hsa-miR-424-5p	RAB30	RAB30, member RAS oncogene family
hsa-miR-424-5p	RSBN1	round spermatid basic protein 1
hsa-miR-424-5p	AREL1	apoptosis resistant E3 ubiquitin protein ligase 1
hsa-miR-424-5p	LCOR	ligand dependent nuclear receptor corepressor
hsa-miR-424-5p	ABCF3	ATP binding cassette subfamily F member 3
hsa-miR-424-5p	WDTC1	WD and tetratricopeptide repeats 1
hsa-miR-424-5p	LAMC1	laminin subunit gamma 1
hsa-miR-424-5p	VTI1B	vesicle transport through interaction with t-SNAREs 1B
hsa-miR-424-5p	ANXA11	annexin A11
hsa-miR-424-5p	SLC35B2	solute carrier family 35 member B2
hsa-miR-424-5p	MOV10	Mov10 RISC complex RNA helicase
hsa-miR-424-5p	PLXNA2	plexin A2
hsa-miR-424-5p	KPNA3	karyopherin subunit alpha 3
hsa-miR-424-5p	IHH	Indian hedgehog signaling molecule
hsa-miR-424-5p	LOC100130451	uncharacterized LOC100130451
hsa-miR-424-5p	ADAMTS6	ADAM metallopeptidase with thrombospondin type 1 motif 6
hsa-miR-424-5p	MDN1	midasin AAA ATPase 1
hsa-miR-424-5p	SLC15A4	solute carrier family 15 member 4
hsa-miR-424-5p	YRDC	yrdC N6-threonylcarbamoyltransferase domain containing
hsa-miR-424-5p	IGF1R	insulin like growth factor 1 receptor
hsa-miR-424-5p	RIMKLB	ribosomal modification protein rimK like family member B
hsa-miR-424-5p	GPN1	GPN-loop GTPase 1
hsa-miR-424-5p	LYPLA2	lysophospholipase 2
hsa-miR-424-5p	CDC27	cell division cycle 27
hsa-miR-424-5p	PTCH1	patched 1
hsa-miR-424-5p	SUZ12	SUZ12, polycomb repressive complex 2 subunit
hsa-miR-424-5p	CUL2	cullin 2
hsa-miR-424-5p	EXT2	exostosin glycosyltransferase 2
hsa-miR-424-5p	NCBP3	nuclear cap binding subunit 3
hsa-miR-424-5p	HOXA10	homeobox A10
hsa-miR-424-5p	CCNJL	cyclin J like
hsa-miR-424-5p	SEMA3D	semaphorin 3D
hsa-miR-424-5p	MAP7	microtubule associated protein 7
hsa-miR-424-5p	ATP13A3	ATPase 13A3

hsa-miR-424-5p	LOC390877	adenylate kinase isoenzyme 1-like
hsa-miR-424-5p	ZNF548	zinc finger protein 548
hsa-miR-424-5p	BCAP29	B cell receptor associated protein 29
hsa-miR-424-5p	SLC1A2	solute carrier family 1 member 2
hsa-miR-424-5p	MAPRE1	microtubule associated protein RP/EB family member 1
hsa-miR-424-5p	FKBP1A	FKBP prolyl isomerase 1A
hsa-miR-424-5p	PDCD1	programmed cell death 1
hsa-miR-424-5p	TMEM121B	transmembrane protein 121B
hsa-miR-424-5p	RTF1	RTF1 homolog, Paf1/RNA polymerase II complex component
hsa-miR-424-5p	PRDM11	PR/SET domain 11
hsa-miR-424-5p	CNTNAP1	contactin associated protein 1
hsa-miR-424-5p	CAMSAP1	calmodulin regulated spectrin associated protein 1
hsa-miR-424-5p	RGMA	repulsive guidance molecule BMP co-receptor a
hsa-miR-424-5p	NEDD9	neural precursor cell expressed, developmentally down-regulated 9
hsa-miR-424-5p	SPAG7	sperm associated antigen 7
hsa-miR-424-5p	RAB40AL	RAB40A like
hsa-miR-424-5p	PTAR1	protein prenyltransferase alpha subunit repeat containing 1
hsa-miR-424-5p	SUSD6	sushi domain containing 6
hsa-miR-424-5p	PID1	phosphotyrosine interaction domain containing 1
hsa-miR-424-5p	MMD	monocyte to macrophage differentiation associated
hsa-miR-424-5p	CCDC85C	coiled-coil domain containing 85C
hsa-miR-424-5p	DNAJA2	DnaJ heat shock protein family (Hsp40) member A2
hsa-miR-424-5p	ZSWIM3	zinc finger SWIM-type containing 3
hsa-miR-424-5p	PDIK1L	PDLIM1 interacting kinase 1 like
hsa-miR-424-5p	SESTD1	SEC14 and spectrin domain containing 1
hsa-miR-424-5p	IL7R	interleukin 7 receptor
hsa-miR-424-5p	SMURF2	SMAD specific E3 ubiquitin protein ligase 2
hsa-miR-424-5p	CCDC83	coiled-coil domain containing 83
hsa-miR-424-5p	SLC39A9	solute carrier family 39 member 9
hsa-miR-424-5p	DLEU7	deleted in lymphocytic leukemia 7
hsa-miR-424-5p	GNAQ	G protein subunit alpha q
hsa-miR-424-5p	TMEM74B	transmembrane protein 74B
hsa-miR-424-5p	CLDN2	claudin 2
hsa-miR-424-5p	TTC14	tetratricopeptide repeat domain 14
hsa-miR-424-5p	MIGA1	mitoguardin 1
hsa-miR-424-5p	ACSL4	acyl-CoA synthetase long chain family member 4
hsa-miR-424-5p	BCL2	BCL2, apoptosis regulator
hsa-miR-424-5p	CARD10	caspase recruitment domain family member 10
hsa-miR-424-5p	FNTA	farnesyltransferase, CAAX box, alpha
hsa-miR-424-5p	SIAH1	siah E3 ubiquitin protein ligase 1
hsa-miR-424-5p	BMPR1A	bone morphogenetic protein receptor type 1A
hsa-miR-424-5p	HECTD4	HECT domain E3 ubiquitin protein ligase 4
hsa-miR-424-5p	MLYCD	malonyl-CoA decarboxylase

hsa-miR-424-5p	GNAI3	G protein subunit alpha i3
hsa-miR-424-5p	PHACTR2	phosphatase and actin regulator 2
hsa-miR-424-5p	GLRX	glutaredoxin
hsa-miR-424-5p	KBTBD2	kelch repeat and BTB domain containing 2
hsa-miR-424-5p	PTPRJ	protein tyrosine phosphatase, receptor type J
hsa-miR-424-5p	EIF3A	eukaryotic translation initiation factor 3 subunit A
hsa-miR-424-5p	GOLGA1	golgin A1
hsa-miR-424-5p	HSPE1-MOB4	HSPE1-MOB4 readthrough
hsa-miR-424-5p	C16orf72	chromosome 16 open reading frame 72
hsa-miR-424-5p	MBNL2	muscleblind like splicing regulator 2
hsa-miR-424-5p	FGF9	fibroblast growth factor 9
hsa-miR-424-5p	MIB1	mindbomb E3 ubiquitin protein ligase 1
hsa-miR-424-5p	PRKAR2A	protein kinase cAMP-dependent type II regulatory subunit alpha
hsa-miR-424-5p	MYT1L	myelin transcription factor 1 like
hsa-miR-424-5p	KCNU1	potassium calcium-activated channel subfamily U member 1
hsa-miR-424-5p	IST1	IST1, ESCRT-III associated factor
hsa-miR-424-5p	PSKH1	protein serine kinase H1
hsa-miR-424-5p	SLC13A1	solute carrier family 13 member 1
hsa-miR-424-5p	ZNF117	zinc finger protein 117
hsa-miR-424-5p	ESRRA	estrogen related receptor alpha
hsa-miR-424-5p	KL	klotho
hsa-miR-424-5p	PPFIA2	PTPRF interacting protein alpha 2
hsa-miR-424-5p	BCL7A	BCL7A, BAF complex component
hsa-miR-424-5p	USP12	ubiquitin specific peptidase 12
hsa-miR-424-5p	SCAI	suppressor of cancer cell invasion
hsa-miR-424-5p	ARPP19	cAMP regulated phosphoprotein 19
hsa-miR-424-5p	KPNA1	karyopherin subunit alpha 1
hsa-miR-424-5p	ZNF704	zinc finger protein 704
hsa-miR-424-5p	E2F7	E2F transcription factor 7
hsa-miR-424-5p	GOLT1B	golgi transport 1B
hsa-miR-424-5p	EPB41L4B	erythrocyte membrane protein band 4.1 like 4B
hsa-miR-424-5p	NF2	neurofibromin 2
hsa-miR-424-5p	NUAK2	NUAK family kinase 2
hsa-miR-424-5p	CCNT2	cyclin T2
hsa-miR-424-5p	SCUBE3	signal peptide, CUB domain and EGF like domain containing 3
hsa-miR-424-5p	TRIP11	thyroid hormone receptor interactor 11
hsa-miR-424-5p	WNT4	Wnt family member 4
hsa-miR-424-5p	G0S2	G0/G1 switch 2
hsa-miR-424-5p	DLL4	delta like canonical Notch ligand 4
hsa-miR-424-5p	CDHR1	cadherin related family member 1
hsa-miR-424-5p	HPSE2	heparanase 2 (inactive)
hsa-miR-424-5p	ASNSD1	asparagine synthetase domain containing 1
hsa-miR-424-5p	OTUD4	OTU deubiquitinase 4

hsa-miR-424-5p	ENTPD7	ectonucleoside triphosphate diphosphohydrolase 7
hsa-miR-424-5p	RORA	RAR related orphan receptor A
hsa-miR-424-5p	ELL2	elongation factor for RNA polymerase II 2
hsa-miR-424-5p	SALL3	spalt like transcription factor 3
hsa-miR-424-5p	RNF43	ring finger protein 43
hsa-miR-424-5p	CLDN12	claudin 12
hsa-miR-424-5p	RAB10	RAB10, member RAS oncogene family
hsa-miR-424-5p	FAM122A	family with sequence similarity 122A
hsa-miR-424-5p	TLR1	toll like receptor 1
hsa-miR-424-5p	ISM2	isthmin 2
hsa-miR-424-5p	USP9X	ubiquitin specific peptidase 9 X-linked
hsa-miR-424-5p	CARNMT1	carnosine N-methyltransferase 1
hsa-miR-424-5p	ERC2	ELKS/RAB6-interacting/CAST family member 2
hsa-miR-424-5p	PPIF	peptidylprolyl isomerase F
hsa-miR-424-5p	NUCKS1	nuclear casein kinase and cyclin dependent kinase substrate 1
hsa-miR-424-5p	CRIM1	cysteine rich transmembrane BMP regulator 1
hsa-miR-424-5p	ANLN	anillin actin binding protein
hsa-miR-424-5p	BOLA3	bolA family member 3
hsa-miR-424-5p	KCNQ5	potassium voltage-gated channel subfamily Q member 5
hsa-miR-424-5p	ATG4B	autophagy related 4B cysteine peptidase
hsa-miR-424-5p	TRIM35	tripartite motif containing 35
hsa-miR-424-5p	MXD3	MAX dimerization protein 3
hsa-miR-424-5p	CLCN3	chloride voltage-gated channel 3
hsa-miR-424-5p	LY9	lymphocyte antigen 9
hsa-miR-424-5p	IP6K1	inositol hexakisphosphate kinase 1
hsa-miR-424-5p	LRRFIP2	LRR binding FLII interacting protein 2
hsa-miR-424-5p	DNAJC16	DnaJ heat shock protein family (Hsp40) member C16
hsa-miR-424-5p	ACSS2	acyl-CoA synthetase short chain family member 2
hsa-miR-424-5p	CASZ1	castor zinc finger 1
hsa-miR-424-5p	LHX3	LIM homeobox 3
hsa-miR-424-5p	STXBP1	syntaxin binding protein 1
hsa-miR-424-5p	CBFA2T3	CBFA2/RUNX1 translocation partner 3
hsa-miR-424-5p	FAM160B1	family with sequence similarity 160 member B1
hsa-miR-424-5p	TTC1	tetratricopeptide repeat domain 1
hsa-miR-424-5p	PNISR	PNN interacting serine and arginine rich protein
hsa-miR-424-5p	KIF1C	kinesin family member 1C
hsa-miR-424-5p	SNRK	SNF related kinase
hsa-miR-424-5p	FAT3	FAT atypical cadherin 3
hsa-miR-424-5p	IRAK2	interleukin 1 receptor associated kinase 2
hsa-miR-424-5p	EML6	EMAP like 6
hsa-miR-424-5p	NOTCH2	notch 2
hsa-miR-424-5p	SRSF11	serine and arginine rich splicing factor 11
hsa-miR-424-5p	CREBL2	cAMP responsive element binding protein like 2
hsa-miR-424-5p	ADSS	adenylosuccinate synthase

hsa-miR-424-5p	ATP7A	ATPase copper transporting alpha
hsa-miR-424-5p	KLHL18	kelch like family member 18
hsa-miR-424-5p	INSYN2	inhibitory synaptic factor 2A
hsa-miR-424-5p	RAPH1	Ras association (RalGDS/AF-6) and pleckstrin homology domains 1
hsa-miR-424-5p	TRMT9B	tRNA methyltransferase 9B (putative)
hsa-miR-424-5p	SLC6A4	solute carrier family 6 member 4
hsa-miR-424-5p	ADAMTSL3	ADAMTS like 3
hsa-miR-424-5p	NAA15	N(alpha)-acetyltransferase 15, NatA auxiliary subunit
hsa-miR-424-5p	ENTPD1	ectonucleoside triphosphate diphosphohydrolase 1
hsa-miR-424-5p	POLR3F	RNA polymerase III subunit F
hsa-miR-424-5p	TFAP2D	transcription factor AP-2 delta
hsa-miR-424-5p	ZNF264	zinc finger protein 264
hsa-miR-424-5p	DOLPP1	dolichyldiphosphatase 1
hsa-miR-424-5p	ARHGAP5	Rho GTPase activating protein 5
hsa-miR-424-5p	OSCP1	organic solute carrier partner 1
hsa-miR-424-5p	ZNF423	zinc finger protein 423
hsa-miR-424-5p	RAF1	Raf-1 proto-oncogene, serine/threonine kinase
hsa-miR-424-5p	PRTG	protogenin
hsa-miR-424-5p	RIC1	RIC1 homolog, RAB6A GEF complex partner 1
hsa-miR-424-5p	HPCAL4	hippocalcin like 4
hsa-miR-424-5p	ESPN	espin
hsa-miR-424-5p	IL10RA	interleukin 10 receptor subunit alpha
hsa-miR-424-5p	SATB2	SATB homeobox 2
hsa-miR-424-5p	YAP1	Yes associated protein 1
hsa-miR-424-5p	RIF1	replication timing regulatory factor 1
hsa-miR-424-5p	EN2	engrailed homeobox 2
hsa-miR-424-5p	MAPK9	mitogen-activated protein kinase 9
hsa-miR-424-5p	RTN4	reticulon 4
hsa-miR-424-5p	MARCH4	membrane associated ring-CH-type finger 4
hsa-miR-424-5p	HMGA2	high mobility group AT-hook 2
hsa-miR-424-5p	ZDHHC23	zinc finger DHHC-type containing 23
hsa-miR-424-5p	RNF41	ring finger protein 41
hsa-miR-424-5p	SLC35A4	solute carrier family 35 member A4
hsa-miR-424-5p	YWHAQ	tyrosine 3-monooxygenase/tryptophan 5-monooxygenase activation protein theta
hsa-miR-424-5p	AFF4	AF4/FMR2 family member 4
hsa-miR-424-5p	C2orf72	chromosome 2 open reading frame 72
hsa-miR-424-5p	ABCB5	ATP binding cassette subfamily B member 5
hsa-miR-424-5p	TMEM43	transmembrane protein 43
hsa-miR-424-5p	SLC25A22	solute carrier family 25 member 22
hsa-miR-424-5p	DEPDC5	DEP domain containing 5
hsa-miR-424-5p	UHMK1	U2AF homology motif kinase 1
hsa-miR-424-5p	TACC1	transforming acidic coiled-coil containing protein 1
hsa-miR-424-5p	TMEM255A	transmembrane protein 255A

hsa-miR-424-5p	SOS2	SOS Ras/Rho guanine nucleotide exchange factor 2
hsa-miR-424-5p	PANK2	pantothenate kinase 2
hsa-miR-424-5p	ZNF275	zinc finger protein 275
hsa-miR-424-5p	FZD6	frizzled class receptor 6
hsa-miR-424-5p	MTHFR	methylenetetrahydrofolate reductase
hsa-miR-424-5p	NPAS3	neuronal PAS domain protein 3
hsa-miR-424-5p	ALDH1A3	aldehyde dehydrogenase 1 family member A3
hsa-miR-424-5p	ABCC5	ATP binding cassette subfamily C member 5
hsa-miR-424-5p	GLYATL3	glycine-N-acyltransferase like 3
hsa-miR-424-5p	ZDHHC16	zinc finger DHHC-type containing 16
hsa-miR-424-5p	PDK3	pyruvate dehydrogenase kinase 3
hsa-miR-424-5p	PRKG1	protein kinase cGMP-dependent 1
hsa-miR-424-5p	MFAP5	microfibril associated protein 5
hsa-miR-424-5p	MTMR4	myotubularin related protein 4
hsa-miR-424-5p	ATP1B4	ATPase Na+/K+ transporting family member beta 4
hsa-miR-424-5p	CALU	calumenin
hsa-miR-424-5p	ABTB2	ankyrin repeat and BTB domain containing 2
hsa-miR-424-5p	FURIN	furin, paired basic amino acid cleaving enzyme
hsa-miR-424-5p	KIF5A	kinesin family member 5A
hsa-miR-424-5p	YOD1	YOD1 deubiquitinase
hsa-miR-424-5p	TSPAN5	tetraspanin 5
hsa-miR-424-5p	PSAT1	phosphoserine aminotransferase 1
hsa-miR-424-5p	SPRTN	SprT-like N-terminal domain
hsa-miR-424-5p	LATS2	large tumor suppressor kinase 2
hsa-miR-424-5p	KLHDC8B	kelch domain containing 8B
hsa-miR-424-5p	TSPYL2	TSPY like 2
hsa-miR-424-5p	NISCH	nischarin
hsa-miR-424-5p	CAAP1	caspase activity and apoptosis inhibitor 1
hsa-miR-424-5p	ANKRD33B	ankyrin repeat domain 33B
hsa-miR-424-5p	ZBTB33	zinc finger and BTB domain containing 33
hsa-miR-424-5p	NTRK2	neurotrophic receptor tyrosine kinase 2
hsa-miR-424-5p	TMEM221	transmembrane protein 221
hsa-miR-424-5p	CREG1	cellular repressor of E1A stimulated genes 1
hsa-miR-424-5p	DEF8	differentially expressed in FDCP 8 homolog
hsa-miR-424-5p	WWP1	WW domain containing E3 ubiquitin protein ligase 1
hsa-miR-424-5p	RTN3	reticulon 3
hsa-miR-424-5p	PAQR3	progestin and adipoQ receptor family member 3
hsa-miR-424-5p	LRRC27	leucine rich repeat containing 27
hsa-miR-424-5p	PARP12	poly(ADP-ribose) polymerase family member 12
hsa-miR-424-5p	SPRY4	sprouty RTK signaling antagonist 4
hsa-miR-424-5p	DENND6A	DENN domain containing 6A
hsa-miR-424-5p	EXTL3	exostosin like glycosyltransferase 3
hsa-miR-424-5p	TOX3	TOX high mobility group box family member 3
hsa-miR-424-5p	TXNDC17	thioredoxin domain containing 17
hsa-miR-424-5p	ZFYVE1	zinc finger FYVE-type containing 1

hsa-miR-424-5p	ELK4	ELK4, ETS transcription factor
hsa-miR-424-5p	LDLRAD4	low density lipoprotein receptor class A domain containing 4
hsa-miR-424-5p	FAM189B	family with sequence similarity 189 member B
hsa-miR-424-5p	ANK2	ankyrin 2
hsa-miR-424-5p	DCAF8	DDB1 and CUL4 associated factor 8
hsa-miR-424-5p	CDC42EP2	CDC42 effector protein 2
hsa-miR-424-5p	TPD52L3	TPD52 like 3
hsa-miR-424-5p	ADAMTSL1	ADAMTS like 1
hsa-miR-424-5p	SCAF11	SR-related CTD associated factor 11
hsa-miR-424-5p	PPM1D	protein phosphatase, Mg2+/Mn2+ dependent 1D
hsa-miR-424-5p	RAPGEFL1	Rap guanine nucleotide exchange factor like 1
hsa-miR-424-5p	METTL8	methyltransferase like 8
hsa-miR-424-5p	PPP2R5C	protein phosphatase 2 regulatory subunit B'gamma
hsa-miR-424-5p	ZNF705E	zinc finger protein 705E
hsa-miR-424-5p	SPSB4	splA/ryanodine receptor domain and SOCS box containing 4
hsa-miR-424-5p	APP	amyloid beta precursor protein
hsa-miR-424-5p	NOL4L	nucleolar protein 4 like
hsa-miR-424-5p	PHKA1	phosphorylase kinase regulatory subunit alpha 1
hsa-miR-424-5p	MMS19	MMS19 homolog, cytosolic iron-sulfur assembly component
hsa-miR-424-5p	CCND3	cyclin D3
hsa-miR-424-5p	RAB40B	RAB40B, member RAS oncogene family
hsa-miR-424-5p	PLBD2	phospholipase B domain containing 2
hsa-miR-424-5p	ADAMTS5	ADAM metallopeptidase with thrombospondin type 1 motif 5
hsa-miR-424-5p	FRMPD1	FERM and PDZ domain containing 1
hsa-miR-424-5p	PIEZO1	piezo type mechanosensitive ion channel component 1
hsa-miR-424-5p	FAT4	FAT atypical cadherin 4
hsa-miR-424-5p	OTUD5	OTU deubiquitinase 5
hsa-miR-424-5p	CLSPN	claspin
hsa-miR-424-5p	RIMS3	regulating synaptic membrane exocytosis 3
hsa-miR-424-5p	COPS7A	COP9 signalosome subunit 7A
hsa-miR-424-5p	KMT2A	lysine methyltransferase 2A
hsa-miR-424-5p	TCIM	transcriptional and immune response regulator
hsa-miR-424-5p	RBM20	RNA binding motif protein 20
hsa-miR-424-5p	BTLA	B and T lymphocyte associated
hsa-miR-424-5p	TPM2	tropomyosin 2
hsa-miR-424-5p	TNFAIP8L3	TNF alpha induced protein 8 like 3
hsa-miR-424-5p	DLGAP1	DLG associated protein 1
hsa-miR-424-5p	DEPTOR	DEP domain containing MTOR interacting protein
hsa-miR-424-5p	LIPE	lipase E, hormone sensitive type
hsa-miR-424-5p	YIF1B	Yip1 interacting factor homolog B, membrane trafficking protein
hsa-miR-424-5p	ZBTB10	zinc finger and BTB domain containing 10

hsa-miR-424-5p	NEBL	nebulette
hsa-miR-424-5p	ETFRF1	electron transfer flavoprotein regulatory factor 1
hsa-miR-424-5p	PWWP2B	PWWP domain containing 2B
hsa-miR-424-5p	LRRC7	leucine rich repeat containing 7
hsa-miR-424-5p	KCNAB1	potassium voltage-gated channel subfamily A member regulatory beta subunit 1
hsa-miR-424-5p	SINHCAF	SIN3-HDAC complex associated factor
hsa-miR-424-5p	C5orf63	chromosome 5 open reading frame 63
hsa-miR-424-5p	DYNC1I1	dynein cytoplasmic 1 intermediate chain 1
hsa-miR-424-5p	SRPRB	SRP receptor subunit beta
hsa-miR-424-5p	PLPP3	phospholipid phosphatase 3
hsa-miR-424-5p	CCNJ	cyclin J
hsa-miR-424-5p	RNF125	ring finger protein 125
hsa-miR-424-5p	PHF20	PHD finger protein 20
hsa-miR-424-5p	PLSCR4	phospholipid scramblase 4
hsa-miR-424-5p	MINDY2	MINDY lysine 48 deubiquitinase 2
hsa-miR-424-5p	BDNF	brain derived neurotrophic factor
hsa-miR-424-5p	PPM1K	protein phosphatase, Mg2+/Mn2+ dependent 1K
hsa-miR-424-5p	PCBP4	poly(rC) binding protein 4
hsa-miR-424-5p	SLITRK1	SLIT and NTRK like family member 1
hsa-miR-424-5p	DCUN1D1	defective in cullin neddylation 1 domain containing 1
hsa-miR-424-5p	ADCY5	adenylate cyclase 5
hsa-miR-424-5p	ZNF267	zinc finger protein 267
hsa-miR-424-5p	PACRG	parkin coregulated
hsa-miR-424-5p	TSC1	TSC complex subunit 1
hsa-miR-424-5p	RGS8	regulator of G protein signaling 8
hsa-miR-424-5p	USP49	ubiquitin specific peptidase 49
hsa-miR-424-5p	ASB1	ankyrin repeat and SOCS box containing 1
hsa-miR-424-5p	ZNF559-ZNF177	ZNF559-ZNF177 readthrough
hsa-miR-424-5p	ZNF177	zinc finger protein 177
hsa-miR-424-5p	CRKL	CRK like proto-oncogene, adaptor protein
hsa-miR-424-5p	POM121C	POM121 transmembrane nucleoporin C
hsa-miR-424-5p	RAB4B	RAB4B, member RAS oncogene family
hsa-miR-424-5p	TTC25	tetratricopeptide repeat domain 25
hsa-miR-424-5p	ELAC2	elaC ribonuclease Z 2
hsa-miR-424-5p	RAB11FIP1	RAB11 family interacting protein 1
hsa-miR-424-5p	WSB1	WD repeat and SOCS box containing 1
hsa-miR-424-5p	ZBTB43	zinc finger and BTB domain containing 43
hsa-miR-424-5p	PPM1H	protein phosphatase, Mg2+/Mn2+ dependent 1H
hsa-miR-424-5p	CD40	CD40 molecule
hsa-miR-424-5p	OSCAR	osteoclast associated, immunoglobulin-like receptor
hsa-miR-424-5p	HSPA1B	heat shock protein family A (Hsp70) member 1B
hsa-miR-424-5p	SLC38A4	solute carrier family 38 member 4
hsa-miR-424-5p	PCDHA8	protocadherin alpha 8
hsa-miR-424-5p	PCDHAC1	protocadherin alpha subfamily C, 1

hsa-miR-424-5p	C8orf58	chromosome 8 open reading frame 58
hsa-miR-424-5p	PANK1	pantothenate kinase 1
hsa-miR-424-5p	PAM	peptidylglycine alpha-amidating monooxygenase
hsa-miR-424-5p	SIDT2	SID1 transmembrane family member 2
hsa-miR-424-5p	PCDHAC2	protocadherin alpha subfamily C, 2
hsa-miR-424-5p	PCDHA13	protocadherin alpha 13
hsa-miR-424-5p	ESRRG	estrogen related receptor gamma
hsa-miR-424-5p	FGF18	fibroblast growth factor 18
hsa-miR-424-5p	PCDHA10	protocadherin alpha 10
hsa-miR-424-5p	PCDHA5	protocadherin alpha 5
hsa-miR-424-5p	BORCS6	BLOC-1 related complex subunit 6
hsa-miR-424-5p	PRR13	proline rich 13
hsa-miR-424-5p	FNDC3B	fibronectin type III domain containing 3B
hsa-miR-424-5p	UNC5B	unc-5 netrin receptor B
hsa-miR-424-5p	PCDHA6	protocadherin alpha 6
hsa-miR-424-5p	PCDHA11	protocadherin alpha 11
hsa-miR-424-5p	RAB23	RAB23, member RAS oncogene family
hsa-miR-424-5p	PCDHA3	protocadherin alpha 3
hsa-miR-424-5p	RNF183	ring finger protein 183
hsa-miR-424-5p	UBE2J1	ubiquitin conjugating enzyme E2 J1
hsa-miR-424-5p	KY	kyphoscoliosis peptidase
hsa-miR-424-5p	PDPR	pyruvate dehydrogenase phosphatase regulatory subunit
hsa-miR-424-5p	MAPK8	mitogen-activated protein kinase 8
hsa-miR-424-5p	COL4A3BP	collagen type IV alpha 3 binding protein
hsa-miR-424-5p	MAP3K21	mitogen-activated protein kinase kinase kinase 21
hsa-miR-424-5p	PCDHA4	protocadherin alpha 4
hsa-miR-424-5p	ABCG4	ATP binding cassette subfamily G member 4
hsa-miR-424-5p	LYPLAL1	lysophospholipase like 1
hsa-miR-424-5p	CDS2	CDP-diacylglycerol synthase 2
hsa-miR-424-5p	HOXC8	homeobox C8
hsa-miR-424-5p	PCDHA1	protocadherin alpha 1
hsa-miR-424-5p	NFS1	NFS1, cysteine desulfurase
hsa-miR-424-5p	PCDHA7	protocadherin alpha 7
hsa-miR-424-5p	PCDHA2	protocadherin alpha 2
hsa-miR-424-5p	PSMA5	proteasome subunit alpha 5
hsa-miR-424-5p	PCDHA12	protocadherin alpha 12
hsa-miR-424-5p	UBE3C	ubiquitin protein ligase E3C
hsa-miR-424-5p	ZNF705A	zinc finger protein 705A
hsa-miR-424-5p	FCHSD2	FCH and double SH3 domains 2
hsa-miR-424-5p	CHORDC1	cysteine and histidine rich domain containing 1
hsa-miR-424-5p	UBAC1	UBA domain containing 1
hsa-miR-424-5p	SNIP1	Smad nuclear interacting protein 1
hsa-miR-424-5p	GRK3	G protein-coupled receptor kinase 3
hsa-miR-424-5p	DHRS7	dehydrogenase/reductase 7

hsa-miR-424-5p	XDH	xanthine dehydrogenase
hsa-miR-424-5p	GEN1	GEN1, Holliday junction 5' flap endonuclease
hsa-miR-424-5p	NLRX1	NLR family member X1
hsa-miR-424-5p	DACH1	dachshund family transcription factor 1
hsa-miR-424-5p	ATP5MC3	ATP synthase membrane subunit c locus 3
hsa-miR-424-5p	TVP23C	trans-golgi network vesicle protein 23 homolog C
hsa-miR-424-5p	RUBCNL	rubicon like autophagy enhancer
hsa-miR-424-5p	WAPL	WAPL cohesin release factor
hsa-miR-424-5p	BACE1	beta-secretase 1
hsa-miR-424-5p	BMX	BMX non-receptor tyrosine kinase
hsa-miR-424-5p	RHPN2	rhophilin Rho GTPase binding protein 2
hsa-miR-424-5p	VSIR	V-set immunoregulatory receptor
hsa-miR-424-5p	IRS1	insulin receptor substrate 1
hsa-miR-424-5p	SGCA	sarcoglycan alpha
hsa-miR-424-5p	CCDC18	coiled-coil domain containing 18
hsa-miR-424-5p	PFKFB4	6-phosphofructo-2-kinase/fructose-2,6-biphosphatase 4
hsa-miR-424-5p	NAT8L	N-acetyltransferase 8 like
hsa-miR-424-5p	NAPEPLD	N-acyl phosphatidylethanolamine phospholipase D
hsa-miR-424-5p	DCAF7	DDB1 and CUL4 associated factor 7
hsa-miR-424-5p	KCNIP1	potassium voltage-gated channel interacting protein 1
hsa-miR-424-5p	RYBP	RING1 and YY1 binding protein
hsa-miR-424-5p	PEX12	peroxisomal biogenesis factor 12
hsa-miR-424-5p	GRB10	growth factor receptor bound protein 10
hsa-miR-424-5p	MAP2K3	mitogen-activated protein kinase kinase 3
hsa-miR-424-5p	EMC4	ER membrane protein complex subunit 4
hsa-miR-424-5p	AP1S2	adaptor related protein complex 1 subunit sigma 2
hsa-miR-424-5p	HARS2	histidyl-tRNA synthetase 2, mitochondrial
hsa-miR-424-5p	TREM1	triggering receptor expressed on myeloid cells 1
hsa-miR-424-5p	RAP2C	RAP2C, member of RAS oncogene family
hsa-miR-424-5p	CRACR2B	calcium release activated channel regulator 2B
hsa-miR-424-5p	SLC30A8	solute carrier family 30 member 8
hsa-miR-424-5p	HAS2	hyaluronan synthase 2
hsa-miR-424-5p	LRP1B	LDL receptor related protein 1B
hsa-miR-424-5p	SEC61A1	Sec61 translocon alpha 1 subunit
hsa-miR-424-5p	MIEF2	mitochondrial elongation factor 2
hsa-miR-424-5p	GRB2	growth factor receptor bound protein 2
hsa-miR-424-5p	PDLIM5	PDZ and LIM domain 5
hsa-miR-424-5p	ANOS1	anosmin 1
hsa-miR-424-5p	CDV3	CDV3 homolog
hsa-miR-424-5p	SMPD1	sphingomyelin phosphodiesterase 1
hsa-miR-424-5p	CPSF6	cleavage and polyadenylation specific factor 6
hsa-miR-424-5p	FRYL	FRY like transcription coactivator
hsa-miR-424-5p	EIF4E	eukaryotic translation initiation factor 4E
hsa-miR-424-5p	SLC24A3	solute carrier family 24 member 3

hsa-miR-424-5p	CLU	clusterin
hsa-miR-424-5p	IPO9	importin 9
hsa-miR-424-5p	COL24A1	collagen type XXIV alpha 1 chain
hsa-miR-424-5p	TEX19	testis expressed 19
hsa-miR-424-5p	OCRL	OCRL, inositol polyphosphate-5-phosphatase
hsa-miR-424-5p	MTAP	methylthioadenosine phosphorylase
hsa-miR-424-5p	ZNF705D	zinc finger protein 705D
hsa-miR-424-5p	UMOD	uromodulin
hsa-miR-424-5p	PARD6B	par-6 family cell polarity regulator beta
hsa-miR-424-5p	VSX1	visual system homeobox 1
hsa-miR-424-5p	EFCAB5	EF-hand calcium binding domain 5
hsa-miR-424-5p	TATDN3	TatD DNase domain containing 3
hsa-miR-424-5p	PLS1	plastin 1
hsa-miR-424-5p	CCDC179	coiled-coil domain containing 179
hsa-miR-424-5p	AJUBA	ajuba LIM protein
hsa-miR-424-5p	TUBA1A	tubulin alpha 1a
hsa-miR-424-5p	ELMO2	engulfment and cell motility 2
hsa-miR-424-5p	CACNA2D4	calcium voltage-gated channel auxiliary subunit alpha2delta 4
hsa-miR-424-5p	CDC23	cell division cycle 23
hsa-miR-424-5p	AP5B1	adaptor related protein complex 5 subunit beta 1
hsa-miR-424-5p	FRY	FRY microtubule binding protein
hsa-miR-424-5p	MED14OS	MED14 opposite strand
hsa-miR-424-5p	TARBP1	TAR (HIV-1) RNA binding protein 1
hsa-miR-424-5p	KCNC2	potassium voltage-gated channel subfamily C member 2
hsa-miR-424-5p	NFRKB	nuclear factor related to kappaB binding protein
hsa-miR-424-5p	RBSN	rabenosyn, RAB effector
hsa-miR-424-5p	ITGA10	integrin subunit alpha 10
hsa-miR-424-5p	DRAM1	DNA damage regulated autophagy modulator 1
hsa-miR-424-5p	AQP11	aquaporin 11
hsa-miR-424-5p	TBCK	TBC1 domain containing kinase
hsa-miR-424-5p	SSRP1	structure specific recognition protein 1
hsa-miR-424-5p	TASP1	taspase 1
hsa-miR-424-5p	SARM1	sterile alpha and TIR motif containing 1
hsa-miR-424-5p	RNF138	ring finger protein 138
hsa-miR-424-5p	B4GALT1	beta-1,4-galactosyltransferase 1
hsa-miR-424-5p	ZC3H11A	zinc finger CCCH-type containing 11A
hsa-miR-424-5p	CYB561D1	cytochrome b561 family member D1
hsa-miR-424-5p	ITPR1	inositol 1,4,5-trisphosphate receptor type 1
hsa-miR-424-5p	UBE2B	ubiquitin conjugating enzyme E2 B
hsa-miR-424-5p	TFPI2	tissue factor pathway inhibitor 2
hsa-miR-424-5p	RBMS1	RNA binding motif single stranded interacting protein 1
hsa-miR-424-5p	CYB5B	cytochrome b5 type B
hsa-miR-424-5p	SENP5	SUMO specific peptidase 5
hsa-miR-424-5p	GRM1	glutamate metabotropic receptor 1

hsa-miR-424-5p	FCHSD1	FCH and double SH3 domains 1
hsa-miR-424-5p	SIDT1	SID1 transmembrane family member 1
hsa-miR-424-5p	SYNDIG1	synapse differentiation inducing 1
hsa-miR-424-5p	SLITRK6	SLIT and NTRK like family member 6
hsa-miR-424-5p	ARRDC4	arrestin domain containing 4
hsa-miR-424-5p	SAMD4A	sterile alpha motif domain containing 4A
hsa-miR-424-5p	MYLK4	myosin light chain kinase family member 4
hsa-miR-424-5p	CYB561	cytochrome b561
hsa-miR-424-5p	CCDC28A	coiled-coil domain containing 28A
hsa-miR-424-5p	KLF7	Kruppel like factor 7
hsa-miR-424-5p	TMEM143	transmembrane protein 143
hsa-miR-424-5p	EPHA1	EPH receptor A1
hsa-miR-424-5p	CCDC149	coiled-coil domain containing 149
hsa-miR-424-5p	ACACB	acetyl-CoA carboxylase beta
hsa-miR-424-5p	LYNX1	Ly6/neurotoxin 1
hsa-miR-424-5p	HSPA8	heat shock protein family A (Hsp70) member 8
hsa-miR-424-5p	BACE2	beta-secretase 2
hsa-miR-424-5p	C15orf40	chromosome 15 open reading frame 40
hsa-miR-424-5p	SRPK1	SRSF protein kinase 1
hsa-miR-424-5p	RPS6KB1	ribosomal protein S6 kinase B1
hsa-miR-424-5p	NHSL1	NHS like 1
hsa-miR-424-5p	MAP3K4	mitogen-activated protein kinase kinase kinase 4
hsa-miR-424-5p	VEGFD	vascular endothelial growth factor D
hsa-miR-424-5p	LGI2	leucine rich repeat LGI family member 2
hsa-miR-424-5p	FAM189A1	family with sequence similarity 189 member A1
hsa-miR-424-5p	BRK1	BRICK1, SCAR/WAVE actin nucleating complex subunit
hsa-miR-424-5p	TXNRD2	thioredoxin reductase 2
hsa-miR-424-5p	ERLIN2	ER lipid raft associated 2
hsa-miR-424-5p	PLEKHB2	pleckstrin homology domain containing B2
hsa-miR-424-5p	GATAD2B	GATA zinc finger domain containing 2B
hsa-miR-424-5p	SNX29	sorting nexin 29
hsa-miR-424-5p	CSMD1	CUB and Sushi multiple domains 1
hsa-miR-424-5p	ESYT3	extended synaptotagmin 3
hsa-miR-424-5p	SKIL	SKI like proto-oncogene
hsa-miR-424-5p	SLC4A8	solute carrier family 4 member 8
hsa-miR-424-5p	OTUB2	OTU deubiquitinase, ubiquitin aldehyde binding 2
hsa-miR-424-5p	PHIP	pleckstrin homology domain interacting protein
hsa-miR-424-5p	ISOC1	isochorismatase domain containing 1
hsa-miR-424-5p	TTPAL	alpha tocopherol transfer protein like
hsa-miR-424-5p	HS3ST5	heparan sulfate-glucosamine 3-sulfotransferase 5
hsa-miR-424-5p	CNNM2	cyclin and CBS domain divalent metal cation transport mediator 2
hsa-miR-424-5p	C11orf24	chromosome 11 open reading frame 24
hsa-miR-424-5p	ASTN1	astrotactin 1
hsa-miR-424-5p	SF3B3	splicing factor 3b subunit 3

hsa-miR-424-5p	SLC16A6	solute carrier family 16 member 6
hsa-miR-424-5p	KCNMA1	potassium calcium-activated channel subfamily M alpha 1
hsa-miR-424-5p	CXorf40B	chromosome X open reading frame 40B
hsa-miR-424-5p	DSEL	dermatan sulfate epimerase like
hsa-miR-424-5p	CACNB4	calcium voltage-gated channel auxiliary subunit beta 4
hsa-miR-424-5p	ELAVL4	ELAV like RNA binding protein 4
hsa-miR-424-5p	GLUD1	glutamate dehydrogenase 1
hsa-miR-424-5p	PTPN14	protein tyrosine phosphatase, non-receptor type 14
hsa-miR-424-5p	DNAJC24	DnaJ heat shock protein family (Hsp40) member C24
hsa-miR-424-5p	PSMD7	proteasome 26S subunit, non-ATPase 7
hsa-miR-424-5p	PKDCC	protein kinase domain containing, cytoplasmic
hsa-miR-424-5p	ZBTB7A	zinc finger and BTB domain containing 7A
hsa-miR-424-5p	PLEKHH1	pleckstrin homology, MyTH4 and FERM domain containing H1
hsa-miR-424-5p	RNF111	ring finger protein 111
hsa-miR-424-5p	GABRA1	gamma-aminobutyric acid type A receptor alpha1 subunit
hsa-miR-424-5p	LAMTOR4	late endosomal/lysosomal adaptor, MAPK and MTOR activator 4
hsa-miR-424-5p	MAMLD1	mastermind like domain containing 1
hsa-miR-424-5p	IL17RE	interleukin 17 receptor E
hsa-miR-424-5p	NACC2	NACC family member 2
hsa-miR-424-5p	DPP8	dipeptidyl peptidase 8
hsa-miR-424-5p	FCRL2	Fc receptor like 2
hsa-miR-424-5p	NRARP	NOTCH regulated ankyrin repeat protein
hsa-miR-424-5p	RAB3IP	RAB3A interacting protein
hsa-miR-424-5p	ZBTB2	zinc finger and BTB domain containing 2
hsa-miR-424-5p	PCDHA9	protocadherin alpha 9
hsa-miR-424-5p	BTN1A1	butyrophilin subfamily 1 member A1
hsa-miR-424-5p	XPR1	xenotropic and polytropic retrovirus receptor 1
hsa-miR-424-5p	NDP	NDP, norrin cystine knot growth factor
hsa-miR-424-5p	LHFPL4	LHFPL tetraspan subfamily member 4
hsa-miR-424-5p	GABRP	gamma-aminobutyric acid type A receptor pi subunit
hsa-miR-424-5p	RAB40A	RAB40A, member RAS oncogene family
hsa-miR-424-5p	MAFK	MAF bZIP transcription factor K
hsa-miR-424-5p	BACH2	BTB domain and CNC homolog 2
hsa-miR-424-5p	SGPL1	sphingosine-1-phosphate lyase 1
hsa-miR-424-5p	VSTM2A	V-set and transmembrane domain containing 2A
hsa-miR-424-5p	FDFT1	farnesyl-diphosphate farnesyltransferase 1
hsa-miR-424-5p	RANBP3	RAN binding protein 3
hsa-miR-424-5p	TBC1D9	TBC1 domain family member 9
hsa-miR-424-5p	KLHL15	kelch like family member 15
hsa-miR-424-5p	COMT	catechol-O-methyltransferase
hsa-miR-424-5p	IRF2BP2	interferon regulatory factor 2 binding protein 2
hsa-miR-424-5p	ANKIB1	ankyrin repeat and IBR domain containing 1

hsa-miR-424-5p	ODAPH	odontogenesis associated phosphoprotein
hsa-miR-424-5p	STX1A	syntaxin 1A
hsa-miR-424-5p	TTC39A	tetratricopeptide repeat domain 39A
hsa-miR-424-5p	MICAL2	microtubule associated monooxygenase, calponin and LIM domain containing 2
hsa-miR-424-5p	MAP3K7	mitogen-activated protein kinase kinase kinase 7
hsa-miR-424-5p	LHPP	phospholysine phosphohistidine inorganic pyrophosphate phosphatase
hsa-miR-424-5p	SYDE1	synapse defective Rho GTPase homolog 1
hsa-miR-424-5p	ARHGEF12	Rho guanine nucleotide exchange factor 12
hsa-miR-424-5p	RELT	RELT, TNF receptor
hsa-miR-424-5p	USP14	ubiquitin specific peptidase 14
hsa-miR-424-5p	CD28	CD28 molecule
hsa-miR-424-5p	PIP4K2C	phosphatidylinositol-5-phosphate 4-kinase type 2 gamma
hsa-miR-424-5p	ELP1	elongator complex protein 1
hsa-miR-424-5p	GNA12	G protein subunit alpha 12
hsa-miR-424-5p	NMD3	NMD3 ribosome export adaptor
hsa-miR-424-5p	CAB39	calcium binding protein 39
hsa-miR-424-5p	PTPRM	protein tyrosine phosphatase, receptor type M
hsa-miR-424-5p	MYO1C	myosin IC
hsa-miR-424-5p	CXCR3	C-X-C motif chemokine receptor 3
hsa-miR-424-5p	CNIH2	cornichon family AMPA receptor auxiliary protein 2
hsa-miR-424-5p	PAFAH2	platelet activating factor acetylhydrolase 2
hsa-miR-424-5p	BPIFA1	BPI fold containing family A member 1
hsa-miR-424-5p	TAOK1	TAO kinase 1
hsa-miR-424-5p	LHFPL5	LHFPL tetraspan subfamily member 5
hsa-miR-424-5p	GLCE	glucuronic acid epimerase
hsa-miR-424-5p	TNIK	TRAF2 and NCK interacting kinase
hsa-miR-424-5p	SSU72	SSU72 homolog, RNA polymerase II CTD phosphatase
hsa-miR-424-5p	M6PR	mannose-6-phosphate receptor, cation dependent
hsa-miR-424-5p	SVEP1	sushi, von Willebrand factor type A, EGF and pentraxin domain containing 1
hsa-miR-424-5p	DCN	decorin
hsa-miR-424-5p	SEC14L4	SEC14 like lipid binding 4
hsa-miR-424-5p	C6orf222	chromosome 6 open reading frame 222
hsa-miR-424-5p	RBMS2	RNA binding motif single stranded interacting protein 2
hsa-miR-424-5p	VAMP1	vesicle associated membrane protein 1
hsa-miR-424-5p	NIM1K	NIM1 serine/threonine protein kinase
hsa-miR-424-5p	CMTM4	CKLF like MARVEL transmembrane domain containing 4
hsa-miR-424-5p	GHSR	growth hormone secretagogue receptor
hsa-miR-424-5p	IGSF23	immunoglobulin superfamily member 23
hsa-miR-424-5p	KCTD1	potassium channel tetramerization domain containing 1
hsa-miR-424-5p	TAF5	TATA-box binding protein associated factor 5
hsa-miR-424-5p	MYADM	myeloid associated differentiation marker
hsa-miR-424-5p	LCP1	lymphocyte cytosolic protein 1

hsa-miR-424-5p	TRIM37	tripartite motif containing 37
hsa-miR-424-5p	FSTL4	follistatin like 4
hsa-miR-424-5p	TRAF6	TNF receptor associated factor 6
hsa-miR-424-5p	VWA8	von Willebrand factor A domain containing 8
hsa-miR-424-5p	RAB3D	RAB3D, member RAS oncogene family
hsa-miR-424-5p	CDC42	cell division cycle 42
hsa-miR-424-5p	TTL	tubulin tyrosine ligase
hsa-miR-424-5p	PIAS1	protein inhibitor of activated STAT 1
hsa-miR-424-5p	GRAMD2B	GRAM domain containing 2B
hsa-miR-424-5p	TBC1D16	TBC1 domain family member 16
hsa-miR-424-5p	ZNF662	zinc finger protein 662
hsa-miR-424-5p	MLLT6	MLLT6, PHD finger containing
hsa-miR-424-5p	DEDD	death effector domain containing
hsa-miR-424-5p	KLHL26	kelch like family member 26
hsa-miR-424-5p	AP2B1	adaptor related protein complex 2 subunit beta 1
hsa-miR-424-5p	ASB7	ankyrin repeat and SOCS box containing 7
hsa-miR-424-5p	SLC22A17	solute carrier family 22 member 17
hsa-miR-424-5p	ZER1	zyg-11 related cell cycle regulator
hsa-miR-424-5p	NFE2L1	nuclear factor, erythroid 2 like 1
hsa-miR-424-5p	PPP1R2	protein phosphatase 1 regulatory inhibitor subunit 2
hsa-miR-424-5p	SOGA1	suppressor of glucose, autophagy associated 1
hsa-miR-424-5p	CWC15	CWC15 spliceosome associated protein homolog
hsa-miR-424-5p	PPIL4	peptidylprolyl isomerase like 4
hsa-miR-424-5p	CHD6	chromodomain helicase DNA binding protein 6
hsa-miR-424-5p	ARMC8	armadillo repeat containing 8
hsa-miR-424-5p	CABLES2	Cdk5 and Abl enzyme substrate 2
hsa-miR-424-5p	GRPR	gastrin releasing peptide receptor
hsa-miR-424-5p	RAB35	RAB35, member RAS oncogene family
hsa-miR-424-5p	FAM174B	family with sequence similarity 174 member B
hsa-miR-424-5p	ACTR1A	ARP1 actin related protein 1 homolog A
hsa-miR-424-5p	C5orf64	chromosome 5 open reading frame 64
hsa-miR-424-5p	TMPRSS15	transmembrane serine protease 15
hsa-miR-424-5p	TMEM138	transmembrane protein 138
hsa-miR-424-5p	PITPNA	phosphatidylinositol transfer protein alpha
hsa-miR-424-5p	ESRP1	epithelial splicing regulatory protein 1
hsa-miR-424-5p	LOC100996842	uncharacterized LOC100996842
hsa-miR-424-5p	VAPB	VAMP associated protein B and C
hsa-miR-424-5p	PTCD3	pentatricopeptide repeat domain 3
hsa-miR-424-5p	PAGR1	PAXIP1 associated glutamate rich protein 1
hsa-miR-424-5p	ZDHHC21	zinc finger DHHC-type containing 21
hsa-miR-424-5p	HIPK3	homeodomain interacting protein kinase 3
hsa-miR-424-5p	FBXO33	F-box protein 33
hsa-miR-424-5p	ARMC1	armadillo repeat containing 1
hsa-miR-424-5p	PDCD4	programmed cell death 4
hsa-miR-424-5p	AK9	adenylate kinase 9

hsa-miR-424-5p	ANKRD13B	ankyrin repeat domain 13B
hsa-miR-424-5p	ADAM10	ADAM metallopeptidase domain 10
hsa-miR-424-5p	VAMP8	vesicle associated membrane protein 8
hsa-miR-424-5p	TMEM33	transmembrane protein 33
hsa-miR-424-5p	SCN4B	sodium voltage-gated channel beta subunit 4
hsa-miR-424-5p	GOLGA4	golgin A4
hsa-miR-424-5p	RAD9A	RAD9 checkpoint clamp component A
hsa-miR-424-5p	DTNA	dystrobrevin alpha
hsa-miR-424-5p	BHLHE41	basic helix-loop-helix family member e41
hsa-miR-424-5p	PRR15L	proline rich 15 like
hsa-miR-424-5p	CADM1	cell adhesion molecule 1
hsa-miR-424-5p	PLPP6	phospholipid phosphatase 6
hsa-miR-424-5p	PEX5	peroxisomal biogenesis factor 5
hsa-miR-424-5p	SH2D2A	SH2 domain containing 2A
hsa-miR-424-5p	TPM3	tropomyosin 3
hsa-miR-424-5p	C8orf86	chromosome 8 open reading frame 86
hsa-miR-424-5p	EYA4	EYA transcriptional coactivator and phosphatase 4
hsa-miR-424-5p	POM121	POM121 transmembrane nucleoporin
hsa-miR-424-5p	HOXA3	homeobox A3
hsa-miR-424-5p	NXPH1	neurexophilin 1
hsa-miR-424-5p	CHRNB2	cholinergic receptor nicotinic beta 2 subunit
hsa-miR-424-5p	GDI2	GDP dissociation inhibitor 2
hsa-miR-424-5p	WASL	Wiskott-Aldrich syndrome like
hsa-miR-424-5p	PGM2L1	phosphoglucomutase 2 like 1
hsa-miR-424-5p	CPNE1	copine 1
hsa-miR-424-5p	CDK6	cyclin dependent kinase 6
hsa-miR-424-5p	NECAP1	NECAP endocytosis associated 1
hsa-miR-424-5p	ESS2	ess-2 splicing factor homolog
hsa-miR-424-5p	SCN2A	sodium voltage-gated channel alpha subunit 2
hsa-miR-424-5p	ORC4	origin recognition complex subunit 4
hsa-miR-424-5p	FBXO22	F-box protein 22
hsa-miR-424-5p	sept-11	septin 11
hsa-miR-424-5p	AEBP2	AE binding protein 2
hsa-miR-424-5p	ZNF436	zinc finger protein 436
hsa-miR-424-5p	CAST	calpastatin
hsa-miR-424-5p	RASSF5	Ras association domain family member 5
hsa-miR-424-5p	THSD4	thrombospondin type 1 domain containing 4
hsa-miR-424-5p	CCDC81	coiled-coil domain containing 81
hsa-miR-424-5p	SLC4A7	solute carrier family 4 member 7
hsa-miR-424-5p	MRAS	muscle RAS oncogene homolog
hsa-miR-424-5p	SBNO1	strawberry notch homolog 1
hsa-miR-424-5p	CXorf40A	chromosome X open reading frame 40A
hsa-miR-424-5p	TFEC	transcription factor EC
hsa-miR-424-5p	AATK	apoptosis associated tyrosine kinase
hsa-miR-424-5p	SLC9B2	solute carrier family 9 member B2

hsa-miR-424-5p	DAZAP2	DAZ associated protein 2
hsa-miR-424-5p	PRKAA1	protein kinase AMP-activated catalytic subunit alpha 1
hsa-miR-424-5p	B3GNT2	UDP-GlcNAc:betaGal beta-1,3-N-acetylglucosaminyltransferase 2
hsa-miR-424-5p	ZNF609	zinc finger protein 609
hsa-miR-424-5p	GORASP1	golgi reassembly stacking protein 1
hsa-miR-424-5p	RDH13	retinol dehydrogenase 13
hsa-miR-424-5p	FSD1	fibronectin type III and SPRY domain containing 1
hsa-miR-424-5p	ADAP1	ArfGAP with dual PH domains 1
hsa-miR-424-5p	SWAP70	switching B cell complex subunit SWAP70
hsa-miR-424-5p	MIPOL1	mirror-image polydactyly 1
hsa-miR-424-5p	MBTPS2	membrane bound transcription factor peptidase, site 2
hsa-miR-424-5p	MYLK3	myosin light chain kinase 3
hsa-miR-424-5p	THUMPD1	THUMP domain containing 1
hsa-miR-424-5p	NEXMIF	neurite extension and migration factor
hsa-miR-424-5p	ZDHHC14	zinc finger DHHC-type containing 14
hsa-miR-424-5p	FLCN	folliculin
hsa-miR-424-5p	KLC4	kinesin light chain 4
hsa-miR-424-5p	PSME3	proteasome activator subunit 3
hsa-miR-424-5p	ZNF699	zinc finger protein 699
hsa-miR-424-5p	SLC9A8	solute carrier family 9 member A8
hsa-miR-424-5p	EFNB2	ephrin B2
hsa-miR-424-5p	PPP2R1A	protein phosphatase 2 scaffold subunit Aalpha
hsa-miR-424-5p	CDC14A	cell division cycle 14A
hsa-miR-424-5p	RPRD1B	regulation of nuclear pre-mRNA domain containing 1B
hsa-miR-424-5p	TLE4	TLE family member 4, transcriptional corepressor
hsa-miR-424-5p	CLCN5	chloride voltage-gated channel 5
hsa-miR-424-5p	SH3BP2	SH3 domain binding protein 2
hsa-miR-424-5p	VCL	vinculin
hsa-miR-424-5p	GNPDA2	glucosamine-6-phosphate deaminase 2
hsa-miR-424-5p	ABI2	abl interactor 2
hsa-miR-424-5p	TTC38	tetratricopeptide repeat domain 38
hsa-miR-424-5p	CNNM3	cyclin and CBS domain divalent metal cation transport mediator 3
hsa-miR-424-5p	BVES	blood vessel epicardial substance
hsa-miR-424-5p	PEAK1	pseudopodium enriched atypical kinase 1
hsa-miR-424-5p	TMEM68	transmembrane protein 68
hsa-miR-424-5p	AMMECR1L	AMMECR1 like
hsa-miR-424-5p	AHNAK2	AHNAK nucleoprotein 2
hsa-miR-424-5p	SRP72	signal recognition particle 72
hsa-miR-424-5p	DNAJC5	DnaJ heat shock protein family (Hsp40) member C5
hsa-miR-424-5p	PLAGL1	PLAG1 like zinc finger 1
hsa-miR-424-5p	C11orf53	chromosome 11 open reading frame 53
hsa-miR-424-5p	DNAJA1	DnaJ heat shock protein family (Hsp40) member A1
hsa-miR-424-5p	FGFR4	fibroblast growth factor receptor 4

hsa-miR-424-5p	PIM1	Pim-1 proto-oncogene, serine/threonine kinase
hsa-miR-424-5p	ZNF362	zinc finger protein 362
hsa-miR-424-5p	PURA	purine rich element binding protein A
hsa-miR-424-5p	MCRIP1	MAPK regulated corepressor interacting protein 1
hsa-miR-424-5p	RNMT	RNA guanine-7 methyltransferase
hsa-miR-424-5p	PSMF1	proteasome inhibitor subunit 1
hsa-miR-424-5p	MRPS2	mitochondrial ribosomal protein S2
hsa-miR-424-5p	TXN2	thioredoxin 2
hsa-miR-424-5p	SNRNP48	small nuclear ribonucleoprotein U11/U12 subunit 48
hsa-miR-424-5p	CNN1	calponin 1
hsa-miR-424-5p	A4GNT	alpha-1,4-N-acetylglucosaminyltransferase
hsa-miR-424-5p	IDH3A	isocitrate dehydrogenase 3 (NAD(+)) alpha
hsa-miR-424-5p	UBL3	ubiquitin like 3
hsa-miR-424-5p	GDPD1	glycerophosphodiester phosphodiesterase domain containing 1
hsa-miR-424-5p	EPB41L1	erythrocyte membrane protein band 4.1 like 1
hsa-miR-424-5p	FOSL1	FOS like 1, AP-1 transcription factor subunit
hsa-miR-424-5p	USP38	ubiquitin specific peptidase 38
hsa-miR-424-5p	GABRE	gamma-aminobutyric acid type A receptor epsilon subunit
hsa-miR-424-5p	PHF21A	PHD finger protein 21A
hsa-miR-424-5p	CA8	carbonic anhydrase 8
hsa-miR-424-5p	ZNF697	zinc finger protein 697
hsa-miR-424-5p	ATP4B	ATPase H+/K+ transporting subunit beta
hsa-miR-424-5p	LMAN2L	lectin, mannose binding 2 like
hsa-miR-424-5p	TECPR2	tectonin beta-propeller repeat containing 2
hsa-miR-424-5p	RNF213	ring finger protein 213
hsa-miR-424-5p	MCFD2	multiple coagulation factor deficiency 2
hsa-miR-424-5p	KCNJ11	potassium voltage-gated channel subfamily J member 11
hsa-miR-424-5p	FBXO10	F-box protein 10
hsa-miR-424-5p	SNCG	synuclein gamma
hsa-miR-424-5p	TRAF3	TNF receptor associated factor 3
hsa-miR-424-5p	TRIM36	tripartite motif containing 36
hsa-miR-424-5p	PDCD11	programmed cell death 11
hsa-miR-424-5p	RLIM	ring finger protein, LIM domain interacting
hsa-miR-424-5p	HIRA	histone cell cycle regulator
hsa-miR-424-5p	KIAA1549	KIAA1549
hsa-miR-424-5p	PARM1	prostate androgen-regulated mucin-like protein 1
hsa-miR-424-5p	BTF3	basic transcription factor 3
hsa-miR-424-5p	KRTAP4-4	keratin associated protein 4-4
hsa-miR-424-5p	TPRG1L	tumor protein p63 regulated 1 like
hsa-miR-424-5p	MTMR11	myotubularin related protein 11
hsa-miR-424-5p	WNT2B	Wnt family member 2B
hsa-miR-424-5p	DCAF17	DDB1 and CUL4 associated factor 17
hsa-miR-424-5p	ABHD13	abhydrolase domain containing 13

hsa-miR-424-5p	TUBGCP2	tubulin gamma complex associated protein 2
hsa-miR-424-5p	LRRTM2	leucine rich repeat transmembrane neuronal 2
hsa-miR-424-5p	ADNP2	ADNP homeobox 2
hsa-miR-424-5p	ODF2L	outer dense fiber of sperm tails 2 like
hsa-miR-424-5p	LYZL4	lysozyme like 4
hsa-miR-424-5p	SELENOI	selenoprotein I
hsa-miR-424-5p	PIK3C2A	phosphatidylinositol-4-phosphate 3-kinase catalytic subunit type 2 alpha
hsa-miR-424-5p	PLEKHA5	pleckstrin homology domain containing A5
hsa-miR-424-5p	FAM161A	FAM161A, centrosomal protein
hsa-miR-424-5p	PIGB	phosphatidylinositol glycan anchor biosynthesis class B
hsa-miR-424-5p	REEP1	receptor accessory protein 1
hsa-miR-424-5p	BCL9L	BCL9 like
hsa-miR-424-5p	SLC7A2	solute carrier family 7 member 2
hsa-miR-424-5p	DENR	density regulated re-initiation and release factor
hsa-miR-424-5p	TP53INP1	tumor protein p53 inducible nuclear protein 1
hsa-miR-424-5p	UBAP1	ubiquitin associated protein 1
hsa-miR-424-5p	MOCS3	molybdenum cofactor synthesis 3
hsa-miR-424-5p	XIRP2	xin actin binding repeat containing 2
hsa-miR-424-5p	CHFR	checkpoint with forkhead and ring finger domains
hsa-miR-424-5p	C11orf68	chromosome 11 open reading frame 68
hsa-miR-424-5p	GCNT3	glucosaminyl (N-acetyl) transferase 3, mucin type
hsa-miR-424-5p	MAP3K3	mitogen-activated protein kinase kinase kinase 3
hsa-miR-424-5p	FAM84B	family with sequence similarity 84 member B
hsa-miR-424-5p	KARS	lysyl-tRNA synthetase
hsa-miR-424-5p	FRS2	fibroblast growth factor receptor substrate 2
hsa-miR-424-5p	USP32	ubiquitin specific peptidase 32
hsa-miR-424-5p	PPP6C	protein phosphatase 6 catalytic subunit
hsa-miR-424-5p	TIMM10B	translocase of inner mitochondrial membrane 10B
hsa-miR-424-5p	ADGRD1	adhesion G protein-coupled receptor D1
hsa-miR-424-5p	C17orf51	chromosome 17 open reading frame 51
hsa-miR-424-5p	CNTN3	contactin 3
hsa-miR-424-5p	NSMCE3	NSE3 homolog, SMC5-SMC6 complex component
hsa-miR-424-5p	PRR11	proline rich 11
hsa-miR-424-5p	ALDH6A1	aldehyde dehydrogenase 6 family member A1
hsa-miR-424-5p	ONECUT2	one cut homeobox 2
hsa-miR-424-5p	C1orf226	chromosome 1 open reading frame 226
hsa-miR-424-5p	DICER1	dicer 1, ribonuclease III
hsa-miR-424-5p	RRAGA	Ras related GTP binding A
hsa-miR-424-5p	SELENOO	selenoprotein O
hsa-miR-424-5p	PMM1	phosphomannomutase 1
hsa-miR-424-5p	SENP2	SUMO specific peptidase 2
hsa-miR-424-5p	TIGAR	TP53 induced glycolysis regulatory phosphatase
hsa-miR-424-5p	PCNX1	pecanex 1

hsa-miR-424-5p	TRPC1	transient receptor potential cation channel subfamily C member 1
hsa-miR-424-5p	ADORA2A	adenosine A2a receptor
hsa-miR-424-5p	TM7SF3	transmembrane 7 superfamily member 3
hsa-miR-424-5p	CASC1	cancer susceptibility 1
hsa-miR-424-5p	RGS5	regulator of G protein signaling 5
hsa-miR-424-5p	OTUD6A	OTU deubiquitinase 6A
hsa-miR-424-5p	ANKRD11	ankyrin repeat domain 11
hsa-miR-424-5p	KLC1	kinesin light chain 1
hsa-miR-424-5p	NSD2	nuclear receptor binding SET domain protein 2
hsa-miR-424-5p	ZHX1	zinc fingers and homeoboxes 1
hsa-miR-424-5p	PPM1L	protein phosphatase, Mg2+/Mn2+ dependent 1L
hsa-miR-424-5p	AKAP12	A-kinase anchoring protein 12
hsa-miR-424-5p	PXMP4	peroxisomal membrane protein 4
hsa-miR-451-5p	OSR1	odd-skipped related transcription factor 1
hsa-miR-451-5p	CUX2	cut like homeobox 2
hsa-miR-451-5p	PSMB8	proteasome subunit beta 8
hsa-miR-451-5p	CXCL16	C-X-C motif chemokine ligand 16
hsa-miR-451-5p	TARP	TCR gamma alternate reading frame protein
hsa-miR-451-5p	ST8SIA4	ST8 alpha-N-acetyl-neuraminide alpha-2,8-sialyltransferase 4
hsa-miR-451-5p	CDKN2D	cyclin dependent kinase inhibitor 2D
hsa-miR-451-5p	MIF	macrophage migration inhibitory factor
hsa-miR-451-5p	FBLN5	fibulin 5
hsa-miR-451-5p	SAMD4B	sterile alpha motif domain containing 4B
hsa-miR-451-5p	CERK	ceramide kinase
hsa-miR-451-5p	CAB39	calcium binding protein 39
hsa-miR-451-5p	VAPA	VAMP associated protein A
hsa-miR-451-5p	LETM2	leucine zipper and EF-hand containing transmembrane protein 2
hsa-miR-451-5p	CMTM6	CKLF like MARVEL transmembrane domain containing 6
hsa-miR-451-5p	USP46	ubiquitin specific peptidase 46
hsa-miR-451-5p	MEX3C	mex-3 RNA binding family member C
hsa-miR-451-5p	PMM2	phosphomannomutase 2
hsa-miR-451-5p	TBC1D9B	TBC1 domain family member 9B
hsa-miR-451-5p	KIAA1217	KIAA1217
hsa-miR-451-5p	MAU2	MAU2 sister chromatid cohesion factor
hsa-miR-451-5p	RNF217	ring finger protein 217
hsa-miR-451-5p	S1PR2	sphingosine-1-phosphate receptor 2
hsa-miR-451-5p	MEGF6	multiple EGF like domains 6
hsa-miR-451-5p	EVL	Enah/Vasp-like
hsa-miR-451-5p	FBXO33	F-box protein 33
hsa-miR-451-5p	CDKN2B	cyclin dependent kinase inhibitor 2B
hsa-miR-451-5p	UCK1	uridine-cytidine kinase 1
hsa-miR-451-5p	ATF2	activating transcription factor 2
hsa-miR-451-5p	CAV1	caveolin 1

hsa-miR-451-5p	C16orf72	chromosome 16 open reading frame 72
hsa-miR-451-5p	DCAF5	DDB1 and CUL4 associated factor 5
hsa-miR-451-5p	RAB5A	RAB5A, member RAS oncogene family
hsa-miR-451-5p	CACHD1	cache domain containing 1
hsa-miR-451-5p	LUZP2	leucine zipper protein 2
hsa-miR-451-5p	EIF2AK3	eukaryotic translation initiation factor 2 alpha kinase 3
hsa-miR-451-5p	AKTIP	AKT interacting protein
hsa-miR-451-5p	FAM171A1	family with sequence similarity 171 member A1
hsa-miR-451-5p	TTN	titin
hsa-miR-451-5p	NEDD9	neural precursor cell expressed, developmentally down-regulated 9

Table S3: delta-CT detail for each of pro-angiogenic and/or pro-hypoxic microRNA of 8 non tumoral brain tissues from patients without glioma who underwent surgery to for epilepsy

	deltaCT (CT^{mirX}- CT^{RNU48})									
**Normal brain tissue**	**mir-100-5p**	**mir-126-5p**	**mir-128-3p**	**mir-132-3p**	**mir-200b-3p**	**mir-200c-3p**	**mir-210-3p**	**mir-221-3p**	**mir-424-5p**	**mir-451-5p**
**1**	-1.36	6.71	-4.16	0.05	8.07	7.14	5.17	-1.83	7.98	-1.89
**2**	-1.02	6.23	-3.86	-0.25	7.88	8.07	4.92	-1.69	6.66	-0.40
**3**	-1.32	5.01	-5.09	-0.43	8.47	7.96	6.04	-2.19	6.74	-0.26
**4**	-0.78	6.23	-3.88	0.13	8.11	8.45	4.41	-1.45	7.73	0.05
**5**	-0.69	6.52	-3.87	-0.53	8.09	8.25	3.81	-1.64	7.87	0.80
**6**	-1.04	6.63	-3.41	0.26	8.37	8.40	4.32	-1.31	8.54	0.25
**7**	-2.29	4.73	-5.13	-0.71	7.79	6.96	4.05	-2.87	6.45	-1.86
**8**	-0.60	6.42	-3.61	-0.23	8.59	8.58	4.99	-1.56	7.00	-1.14
**deltaCT** (mean ±SD)	-1.14 ±0.54	6.06 ±0.76	-4.12 ±0.64	-0.22 ±0.34	8.17 ±0.28	7.97 ±0.61	4.71 ±0.72	-1.82 ±0.50	7.37 ±0.76	-0.66 ±0.98

CT: cycle threshold. SD: standard deviation
RNU48: internal control

Table S4. Comparison of pro-angiogenic and/or pro-hypoxic miRNA effects between WHO 2016 classes.

	**OS**	**PFS**
	p interaction*	p interaction*
**mir-200b-3p**	0.19	0.15
**mir-200c-3p**	NC†	0.21
**mir-210-3p**	0.26	NC†
**mir-100-5p**	0.17	**0.013**
**mir-126-5p**	0.49	0.46
**mir-132-3p**	0.72	0.72
**mir-221-3p**	0.71	0.87
**mir-424-5p**	0.30	0.061
**mir-128-3p**	0.72	**0.033**
**mir-451-5p**	NC†	0.26

* comparison of the Hazard Ratios of the 6 classes, † not calculable (too few people), OS: Overall survival; PFS: Progression Free Survival; WHO: World Health Organization
